# Enantioselective Organocatalytic Desymmetric Acylation as an Access to Orthogonally Protected *myo*-Inositols

**DOI:** 10.1021/acs.joc.5c02735

**Published:** 2025-12-23

**Authors:** Ondřej Hladík, Vojtěch Dočekal, Ivana Císařová, Jan Veselý

**Affiliations:** 1 Department of Organic Chemistry, 112302 Faculty of Science, Charles University, Hlavova 2030/8, 128 00 Prague 2, Czech Republic; 2 Department of Inorganic Chemistry, Faculty of Science, Charles University, Hlavova 2030/8, 128 00 Prague 2, Czech Republic

## Abstract

Chiral cyclitols represent an important class of naturally occurring compounds. In particular, *myo*-inositol and its derivatives are essential for phosphorus storage and cell-signaling pathways in living organisms. Not surprisingly, these compounds constitute an emerging class of molecules with significant potential in both medicinal and synthetic chemistry. However, efficient catalytic methodologies for accessing chiral *myo*-inositol derivatives remain scarce. Herein, we report a metal-free, organocatalytic protocol for the desymmetric acylation of a readily available *meso*-*myo*-inositol diol. The reaction proceeds with high enantioselectivity, moderate to high yields, and broad tolerance to various functional groups. The developed methodology enables the efficient synthesis of chiral *myo*-inositol derivatives. Furthermore, its scalability and subsequent transformations into orthogonally protected building blocks for synthesizing *myo*-inositol phosphates underscore the practical utility of this approach.

## Graphical abstract

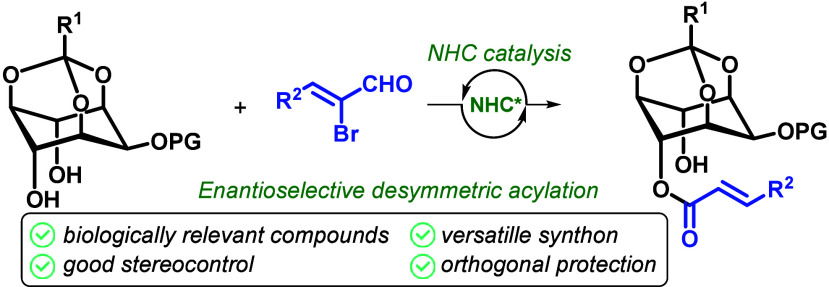

## Introduction

Cyclitols are cycloalkanes with hydroxyl groups attached to at least three carbon atoms.^1^ Among them, cyclohexane-derived polyols constitute a class of particular importance for the function and survival of living cells.^2^ The most important members are cyclohexanehexols, known as inositols. Of the nine stereoisomeric forms, *myo*-inositol with five equatorial and one axial hydroxy group is the most abundant isomer. *Myo*-inositol serves as a key structural unit in naturally occurring molecules (Figure 1A), widely distributed in plants and mammalian cells, and plays multiple roles in cell function and survival across both eukaryotic and prokaryotic systems.^3^ For instance, phosphatidylinositols (PIs) and their phosphorylated derivatives, such as inositol hexakisphosphate (phytic acid, IP_6_) and inositol lipids, are involved in diverse physiological functions, including regulation of ion-channel permeability, phosphate homeostasis, metabolic flux, gene expression, insulin signaling, embryonic development, and stress response.^4^ Additionally, *myo*-inositol and its chiral derivatives have been successfully utilized as a versatile starting material for the synthesis of natural products^5,6^ or biologically relevant inositol derivatives (Figure 1A).^7^

**Figure 1:**
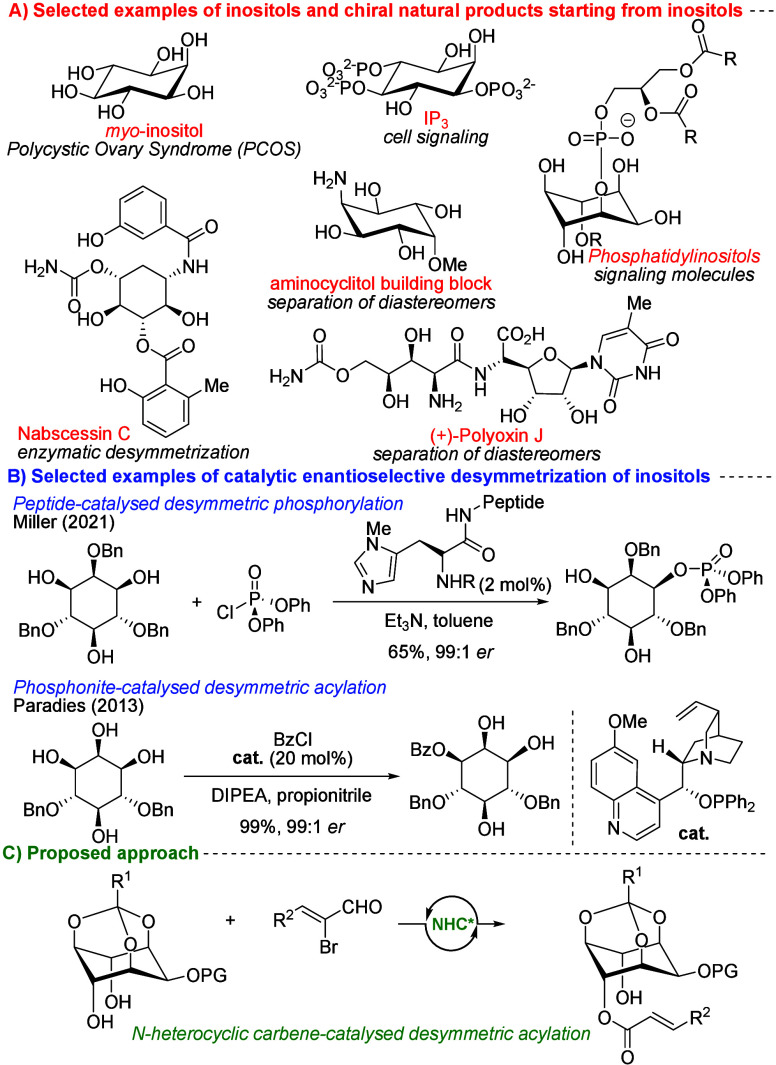
A) Selected examples of biologically relevant inositol and total synthesis products starting from inositols. B) Selected desymmetric approaches toward chiral inositol derivatives. C) Our proposed approach.

*Myo*-inositol is a *meso* compound that possesses a plane of symmetry. In contrast, unsymmetrical substitution breaks this symmetry, affording derivatives of high importance. Thus, developing efficient methodologies to access such *myo*-inositol units remains a central goal in modern synthetic chemistry.^8^ Several well-established approaches have been reported, including the separation of diastereomeric mixtures (via chiral auxiliaries), synthesis from enantiopure synthons such as the Ferrier carbocyclization of methylglucoside, or benzoin condensation.^9^ However, these methods are often hampered by low yields or demanding reaction conditions.^10^ These limitations can be overcome by adopting the concept of catalytic enantioselective desymmetrization. This strategy has been extensively studied by Scott J. Miller, who developed direct desymmetric sulfonylation^11^ and phosphorylations of inositols using peptide catalysis (Figure 1B).^12^ Another efficient approach was reported by J. Paradies in 2013, employing phosphinite catalysis with benzoyl chloride as the acylating agent (Figure 1B).^13^ Alternatively, enzymatic desymmetric transformations were reported.^14^ Despite these advances, the development of efficient methodologies to access highly substituted and orthogonally protected inositol derivatives remains highly desirable.

To address this challenge, *N*-heterocyclic carbene (NHC) catalysis could represent a promising strategy. Nowadays, NHC-catalyzed desymmetrizations of prochiral or *meso* compounds provide access to a variety of enantioenriched products, including selective functionalization of sugars.^15,16^ However, efficient carbene-catalyzed desymmetrizations of diols remain rare.^17^ Motivated by the broad utility of precisely substituted chiral *myo*-inositol derivatives, we developed a novel enantioselective strategy: a desymmetric, nonoxidative acylation reaction catalyzed by chiral NHCs (Figure 1C).

## Results and Discussion

At the outset of the study, we proposed *myo*-inositol **1c** as an easily accessible prochiral starting material, considering the accessibility and versatility of both protecting groups.^18^ To our delight, simple mixing of this diol (**1c**) with α-bromocinnamic aldehyde **2a**,^19^ a chiral NHC precursor (*pre*-**C1**, Bode catalyst), and an excess of base (cesium carbonate) resulted in the formation of the expected chiral product (**3a**). Compound **3a** was obtained in 65% yield as an inseparable mixture of *E*/*Z*-isomers (6:1, based on NMR and HPLC) with moderate enantioselectivity (60:40 *er*, Table 1, entry 1). Building on this proof-of-concept experiment, we aimed to improve the yield and stereoselectivity of the model reaction by varying chiral precursors, bases, additives, and other reaction parameters (for a full optimization survey, please refer to the Supporting Information).

**Table 1:**
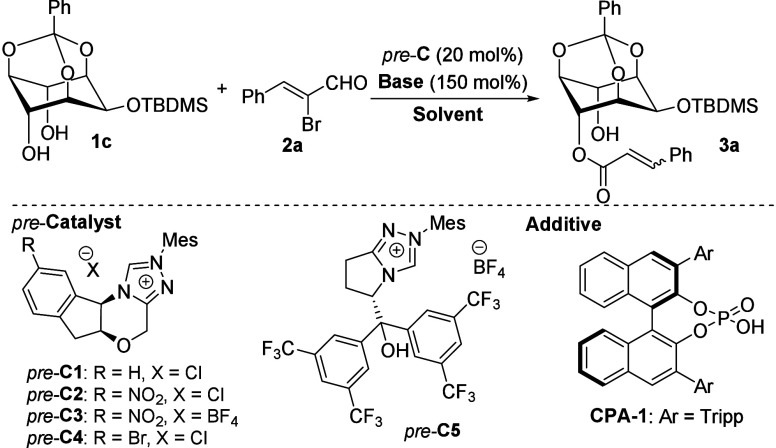
Optimization Studies

Entry^a^	*pre*-**C**	Base	Sol.	Time (h)	Yield^b^ (%)	*E*/*Z* ^c^	*er* ^d^
1	*pre*-**C1**	Cs_2_CO_3_	DCM	18	65	6:1	60:40
2	*pre*-**C2**	Cs_2_CO_3_	DCM	2	61	11:1	68:32
3	*pre*-**C3**	Cs_2_CO_3_	DCM	18	90	13:1	79:21
4	*pre*-**C4**	Cs_2_CO_3_	DCM	18	59	5:1	69:31
5	*pre*-**C5**	Cs_2_CO_3_	DCM	18	96	3:1	70:30
6	*pre*-**C3**	Na_2_CO_3_	DCM	18	98	20:1	79:21
7	*pre*-**C3**	DABCO	DCM	18	53	20:1	83:17
8	*pre*-**C3**	DABCO	CHCl_3_	18	47	20:1	88:12
9	*pre*-**C3**	DABCO	CCl_4_	18	57	20:1	91:9
10	*pre*-**C3**	Na_2_CO_3_	CCl_4_	18	45	20:1	87:13
11	*pre*-**C3**	DABCO	PhCl	1	61	20:1	85:15
12^e^	*pre*-**C3**	DABCO	PhCl	2	54	20:1	84:16
13^f^	*pre*-**C3**	DABCO	PhCl	18	65	20:1	85:15
14^g^	*pre*-**C3**	DABCO	PhCl	42	53	20:1	86:14

a Reactions were conducted with **1c** (0.10 mmol), **2a** (0.12 mmol), selected base (0.12 mmol) and selected *pre*-**C**atalyst (20 mol %) in selected solvent (1.0 mL) at room temperature (∼21 °C).

b Isolated yield after column chromatography.

c Determined by ^1^H NMR and HPLC analysis.

d Determined by chiral HPLC analysis, only for major *E*-isomer; for *er* of minor isomer, please refer to the SI file.

e **CPA-1** (20 mol %) was used as an additive

f 1 mol % of *pre*-**C3** was used.

g Reaction was conducted at 0 °C.

A slightly increased enantiopurity (68:32 *er*) was observed for the product **3a** isolated from the model reaction catalyzed by a nitro-substituted Bode catalyst (*pre*-**C2**, entry 2).^20^ Further improvement in enantiocontrol was achieved by changing the precursor counteranion to tetrafluoroborate (*pre*-**C3**, entry 3), which led to the formation of the expected product in excellent isolated yield (90%) with a good level of stereocontrol (79:21 *er*, 13:1 *E*/*Z*). In contrast, no improvement in stereoselectivity was observed using other morpholine-based precursors (such as *pre*-**C4**) or bifunctional catalyst combining carbene and hydrogen-bond donor functionality (*pre*-**C5**, entry 5). Following the optimization of the model reaction catalyzed by *pre*-**C3**, it was revealed that the reaction is tolerant to changes in base or solvent. For example, substituting cesium carbonate with sodium carbonate resulted in a nearly quantitative yield of product **3a** with retained enantioselectivity, obtained as a single *E*-isomer (entry 6). A decreased yield but improved enantiocontrol (83:17 *er*) was observed when the model reaction was conducted in the presence of DABCO (entry 7). Building on this result, we continued optimization using DABCO as the base. Upon the next optimization, we identified chlorinated solvents as particularly suitable for this transformation (entries 8, 9). For instance, the reaction conducted in tetrachloromethane (entry 9) provided product **3a** in moderate yield (57%) with the highest enantiopurity observed (91:9 *er*). Encouragingly, the enantiopurity could be further increased to 99.7:0.3 *er* by crystallization from isopropanol. Other solvents, such as chlorobenzene, did not improve enantioselectivity (entry 11). However, the model reaction conducted in chlorobenzene reached full conversion of the starting material within 1 h, which we considered a promising outcome for the following optimization. Based on a previous report,^21^ we tested chiral phosphoric acid (**CPA-1**) as an additive for the desymmetric acylation. Notably, no improvement in yield or stereochemical outcome was observed (entry 12). Interestingly, lowering *pre*-**C3** loading to 1 mol % (entry 13) did not result in a decrease in performance. Finally, performing the model reaction at 0 °C (entry 14) furnished the desired product in reduced yield (53%) with minimal change in enantiocontrol.

Considering the above-mentioned optimization results, we propose using 1 mol % of *pre*-**C3** with DABCO in chlorobenzene as the optimal condition for the desymmetric acylation reaction (entry 13).

After optimizing the reaction conditions, we investigated various carbonyl derivatives as alternatives to α-bromocinnamic aldehydes (Scheme 1). First, we tested carbonyl compounds, including activated ester and *trans*-cinnamaldehyde, as potential acylation partners leading to product **3a** (Scheme 1A). Notably, in the reaction with *trans*-cinnamaldehyde, oxidation of the Breslow intermediate was required to generate the corresponding acylation partner. Drawing inspiration from previous studies,^22^ we employed an excess of **DQ** (Kharasch reagent, 3,3′,5,5′-tetra-*tert*-butyldiphenoquinone) as the oxidant. However, this approach did not improve either the yield or the stereochemical outcome. For example, oxidative esterification under the optimized conditions afforded the expected product in a lower isolated yield (44%) while maintaining enantioselectivity (83:17 *er*, single *E*-isomer). Next, we examined saturated analogues as possible alternatives. However, no acylation product was detected under the reaction conditions (Scheme 1B). As a final example, we explored dialdehyde-based ethers, which have been widely studied in desymmetric esterifications and are known to give rise to highly enantioenriched axially chiral derivatives.^23^ This concept, termed as “double desymmetrization” (Scheme 1C),^24^ was based on the hypothesis that the introduction of an additional stereogenic element (stereogenic axis) could enhance asymmetric induction in both desymmetrization processes and potentially allow diastereomer separation. Unfortunately, neither our optimized nor previously reported conditions resulted in full conversion of the starting material, and no improvement in stereochemical outcome was observed. For example, under our optimized conditions, the expected product **5b** was isolated in moderate yield (49%) as an inseparable mixture of diastereomers (1:1.4 *dr*) with moderate enantiopurity (74:26/83:13 *er*).

**Scheme 1:**
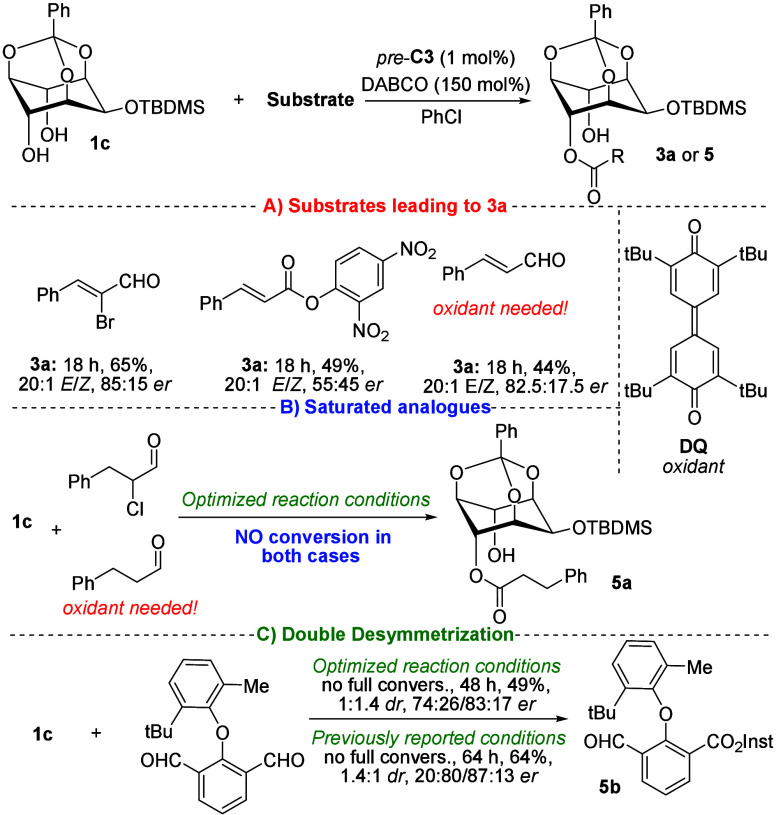
Screening of Various Acylation Reagents

Next, we investigated the effect of various substrates on reaction efficiency in terms of yield and stereochemical outcome (Scheme 2). Interestingly, the model reaction performed with the opposite enantiomer of the chiral precatalyst (*ent*-*pre*-**C3**) provided the enantiomeric product *ent*-**3a** in high yield (92%) with virtually retained enantiopurity (88:12 *er*). Then, we examined the influence of protecting groups on the *myo*-inositol derivative **1** (Scheme 2A). Changing the orthobenzoate to an orthoformate resulted in both lower yield and reduced enantioselectivity of **3b**. Variations of the silyl-protecting group did not improve the outcome; for example, replacement with a benzoyl group resulted in a nearly racemic mixture (53:47 *er*). Notably, the monoprotected derivative **1a** (lacking protection at the equatorial hydroxyl group) afforded the diacylated product in low yield (23%) and moderate enantioselectivity (72:28 *er*).

**Scheme 2:**
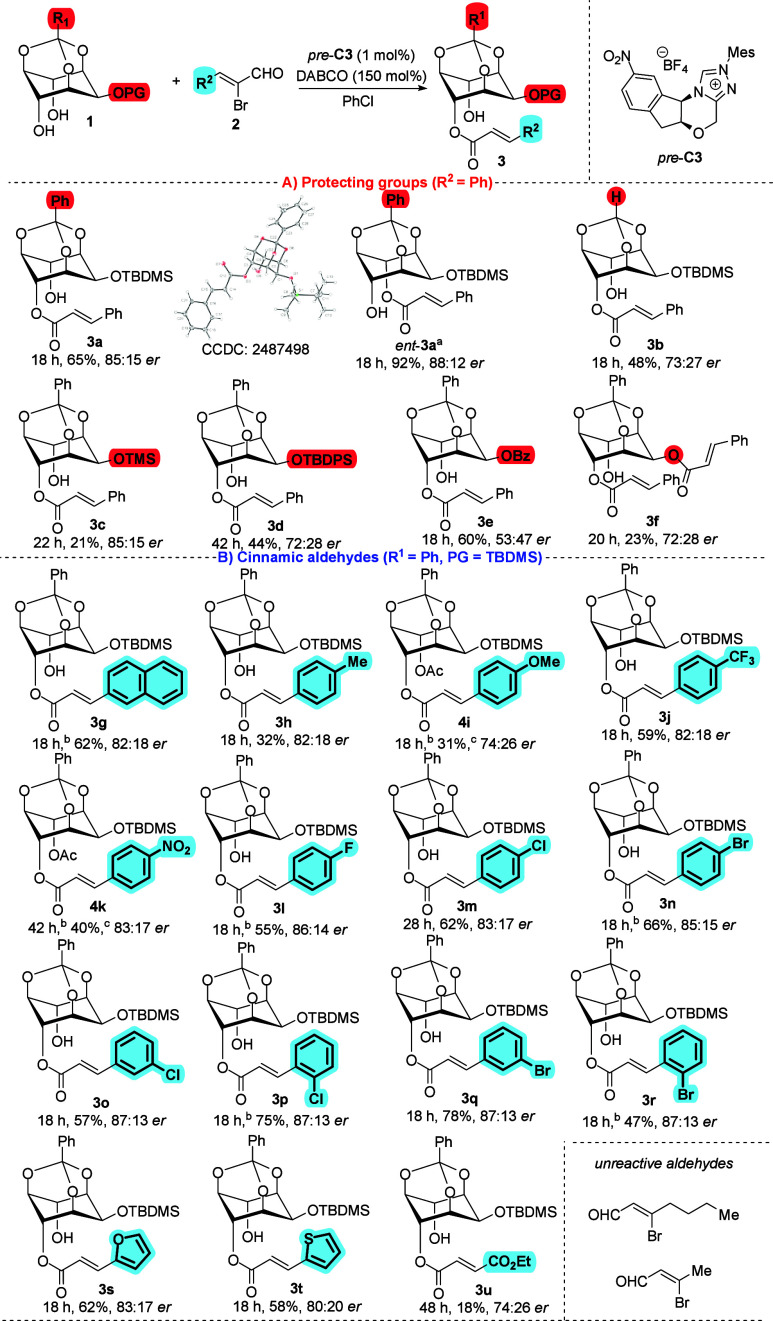
Substrate Scope

a
*Ent-pre*-**C3** was used.
b Full consumption of **1** was not observed, aldehyde **2** was consumed.
c Separated after acetylation, yield refer to compound **4** (over two steps).

Later, we explored the influence of various α-bromocinnamic aldehydes **2** in the acylation reaction with **1c** (Scheme 2B). Gratifyingly, no significant changes in yield or enantioselectivity were observed with a naphthalene-derived cinnamic aldehyde. However, significantly decreased yields were obtained with α-bromocinnamic aldehydes bearing electron-donating groups (EDGs) in the *para*-position of the aromatic ring. A similar trend was observed with strong electron-withdrawing groups (EWGs) at the same position. For example, the trifluoromethyl-substituted product **3j** was obtained in 59% yield with 82:18 *er*. In contrast, aldehydes containing weak EWGs furnished the corresponding products in comparable yields to the model reaction but with reduced enantioselectivity. Overall, the expected products **3l**-**n** were isolated in yields ranging from 55 to 67% yield with enantioselectivities above 83:17 *er*. We also investigated the effect of substituent position on the aromatic ring using chloro- and bromo-substituted cinnamic aldehydes. Notably, no significant effect was observed. For example, the desymmetric acylation of *meta*-bromo-α-bromocinnamic aldehyde with **1c** furnished product **3q** in 78% yield with 87:13 *er*. Gratifyingly, the substrate scope could be further extended to heterocyclic α-bromocinnamic aldehydes as well as to ester-derived aldehyde. In contrast, no conversion of the starting material was observed with an aliphatic α-bromounsaturated aldehydes.

Finally, to evaluate the practicality and synthetic utility of our methodology (Scheme 3). We performed a gram-scale reaction of **1c** under the optimized conditions (Scheme 3A). The desired product **3a** was isolated in a 50% yield with enantioselectivity comparable to the small-scale reaction.

**Scheme 3:**
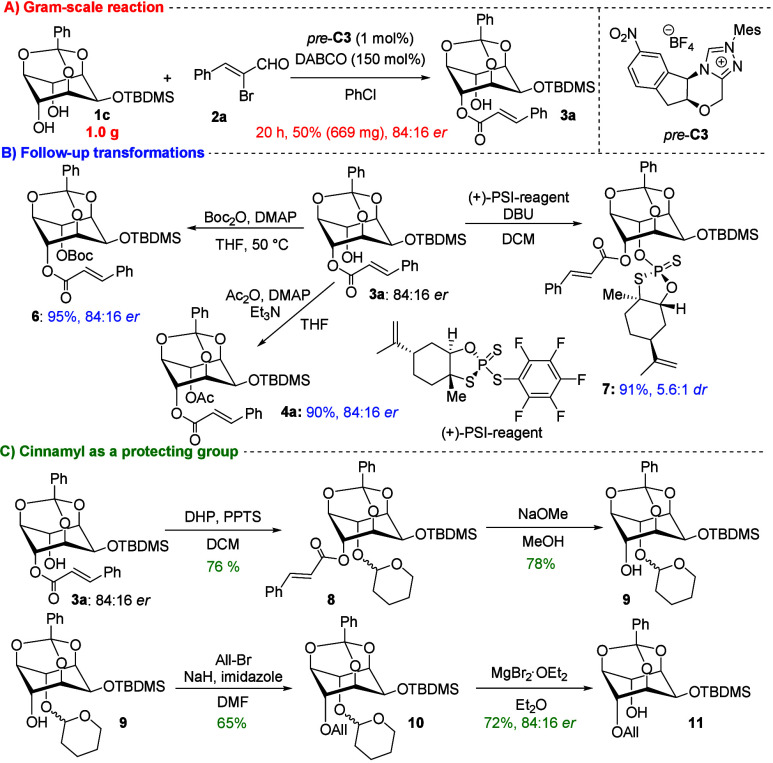
Gram-Scale Reaction and Synthetic Utility Demonstration

Further transformation of the free hydroxyl group (Scheme 3B) was achieved by acylation with Boc or acetyl groups under DMAP-catalyzed conditions, affording fully protected derivatives in nearly quantitative yields while retaining enantiopurity. Inspired by the biological relevance of *myo*-inositols, we next introduced phosphorylated analogues. Using the chiral (+)-PSI reagent developed by Phil S. Baran and co-workers,^25^ phosphorylation of **3a** furnished the expected, hardly separable diastereomeric products in excellent yield, with a diastereomeric ratio consistent with the enantiopurity of the starting material.

We further examined the synthetic utility of the cinnamyl group as a protecting group (Scheme 3C). To preserve molecular chirality, the free hydroxyl group was first protected with a tetrahydropyranyl (THP) group, affording the expected diastereomeric product **8** in excellent yield. Importantly, selective removal of the cinnamyl group under Zemplén-like conditions proceeded smoothly, giving the desired product **9** in excellent yield. To verify that enantiopurity was retained throughout, we carried out allylation of the free hydroxyl group under standard conditions, followed by selective THP deprotection. Notably, for the deprotection of THP magnesium bromide was used,^26^ under these conditions all protecting groups remained intact, and the final product was obtained in excellent yield with preserved optical purity from **3a**.

## Conclusion

In summary, we have developed an efficient and versatile methodology for the enantioselective desymmetric acylation of prochiral *myo*-inositol-derived diols, providing straightforward access to valuable chiral derivatives.^27^ This operationally simple approach demonstrates the potential of organocatalytic desymmetrization in the synthesis of biologically relevant molecules. The method features broad functional group tolerance, scalability, and synthetic applicability, as demonstrated by follow-up transformations. Ongoing studies in our laboratory are directed toward applying this methodology to the synthesis of biologically relevant *myo*-inositol derivatives.

## Experimental Section

Chemicals and solvents were purchased from commercial suppliers and purified using standard techniques. Thin-layer chromatography (TLC) was performed using silica gel plates Merck 60 F_254_. The compounds were visualized by irradiation with UV light and/or by treatment with a solution of phosphomolybdic acid (AMC) followed by heating. Column chromatography was performed using silica gel SiliCycle-SiliaFlash P60 (particle size: 40–63 μm, pore diameter: 60 Å. ^1^H, ^13^C NMR, ^19^F and ^31^P spectra were recorded with Bruker AVANCE III 400. Chemical shifts for protons are given in δ relative to tetramethylsilane (TMS) and referenced to residual protium in the NMR solvent (CDCl_3_: δ_H_ = 7.26 ppm, DMSO-*d*_6_: δ_H_ = 2.50 ppm, D_2_O: δ_H_ = 4.79 ppm). Chemical shifts for carbon are referenced to the carbon of the NMR solvent (CDCl_3_: δ_C_ = 77.16 ppm, DMSO-*d*_6_: δ_C_ = 39.52 ppm). The coupling constants *J* are given in hertz. IR DRIFT spectra were recorded on a Nicolet AVATAR 370 FT-IR in cm^–1^. Chiral HPLC was performed on a LC20AD Shimadzu liquid chromatograph with an SPD-M20A diode array detector with Daicel Chiralpak IA, Daicel Chiralpak IB, Daicel Chiralpak IC, Daicel Chiralpak IG, Daicel Chiralpak IH and Daicel Chiralpak ODH columns. For chiral HPLC, the samples were prepared by dissolving them in *i*-PrOH. Optical rotations were measured on AU-Tomatica polarimeter, Autopol III, and specific optical rotations are given in concentrations *c* [g/100 mL], the samples were prepared by dissolving them in specified solvent for each compound. All melting points were measured on a Büchi melting point B-545 apparatus, in an open glass capillary, and all values are uncorrected. High-resolution mass spectra were recorded on an LCQ Fleet spectrometer using a Bruker Compact QTOF-MS controlled by the Compass 1.9 Control software to measure the ESI high-resolution mass spectra. The monoisotopic mass values were calculated using Data analysis software v 4.4. The analysis was conducted in the positive or negative ion mode at a scan range from *m*/*z* 50 to 1000, and nitrogen was used as nebulizer gas at a pressure of 4 psi and flow of 3 L/min for the dry gas. The capillary voltage and temperature were set at 4500 V and 220 °C, respectively. For HRMS, the samples were prepared by dissolving them in methanol.

### General Procedure A: Preparation of Bridged *myo*-Inositols

Synthesis of protected *myo*-inositols (**1a,b**) was conducted under modified conditions previously reported in the literature.^28^

Round bottom flask equipped with a magnetic stirring bar was charged with *myo*-inositol (1.0 equiv), trialkylorthoacylate (1.8–2.2 equiv), *p*-toluenesulfonic acid monohydrate (15–30 mol %) and DMF (1–1.5 mL/mmol). This mixture was then heated at 100–145 °C (oil bath) for 3–4 h. After cooling to the room temperature was the acid quenched by adding triethylamine (15–30 mol %). The solvent was evaporated under reduced pressure. Product was purified either by filtration or by column chromatography.

#### D-*myo*-Inositol-1,3,5-orthobenzoate (**1a**)

The title compound was synthesized according to the general procedure A, using *myo*-inositol (9.01 g, 50 mmol), trimethylorthobenzoate (18.9 mL, 110 mmol), *p*-toluenesulfonic acid monohydrate (2.76 g, 14.5 mmol) and DMF (80 mL) and heating to 145 °C overnight. The reaction was quenched by adding Et_3_N (2.1 mL,15 mmol). The crude product was purified by column chromatography (hexane/EtOAc – 1:10), affording **1a** (6.2 g, 47%) as a white amorphous solid.

^1^H NMR (400 MHz, DMSO-*d*_*6*_) δ 7.60–7.53 (m, 2H), 7.41–7.29 (m, 3H), 5.51 (s, 2H), 5.33 (d, *J* = 6.3 Hz, 1H), 4.44–4.38 (m, 2H), 4.24–4.19 (m, 1H), 4.18–4.13 (m, 2H), 4.09 (d, *J* = 5.0 Hz, 1H) ppm. ^13^C­{^1^H} NMR (101 MHz, DMSO-*d*_*6*_) δ 137.9, 129.0, 127.6 (2C), 125.5 (2C), 106.5, 75.8 (2C), 70.1, 67.2 (2C), 57.8. HRMS (ESI+) *m*/*z*: calcd. for C_13_H_14_NaO_6_ [M + Na]^+^: 289.0683, found: 289.0679. Our physical and spectroscopic data matched previously reported data.^18^

#### D-*myo*-Inositol-1,3,5-orthoformate (**1b**)

The title compound was synthesized according to the general procedure A, using *myo*-inositol (6.00 g, 33.3 mmol), triethylorthoformate (10.0 mL, 60.6 mmol), *p*-toluenesulfonic acid monohydrate (1.01 g, 5.3 mmol) and DMF (38 mL) and heating to 100 °C (oil bath) overnight. The product was purified by filtration from EtOAc/MeOH, affording **1b** (1.89 g, 30%) as a white/beige amorphous solid.

^1^H NMR (400 MHz, D_2_O) δ 5.62 (s, 1H), 4.62–4.57 (m, 2H), 4.38–4.33 (m, 1H), 4.31–4.23 (m, 3H) ppm. ^13^C­{^1^H} NMR (101 MHz, D_2_O) δ 102.1, 73.8 (2C), 69.3, 66.7 (2C), 59.6 ppm. HRMS (ESI−) *m*/*z*: calcd. for C_7_H_9_O_6_ [M - H]^−^: 189.0405, found: 189.0399. Our physical and spectroscopic data matched previously reported data.^29^

### General Procedure B: Preparation of 2-Protected Bridged *myo*-Inositols

To a stirred solution of a bridged *myo*-inositol (1.0 equiv) with 2,6-lutidine (2.5 equiv) or imidazole (2.2 equiv) in DMF (2.5 mL/mmol) was added the protecting agent (1.05 equiv) portionwise. The reaction was stirred at room temperature until full conversion (TLC monitored). The solvent was then evaporated under reduced pressure. The crude product was purified by column chromatography.

#### 2-O-*tert*-Butyldimethylsilyl-d-*myo*-inositol-1,3,5-orthobenzoate (**1c**)

The title compound was synthesized according to the general procedure B, using bridged *myo*-inositol **1a** (2.06 g, 7.74 mmol), TBDMSCl (1.22 g, 8.12 mmol), 2,6-lutidine (2.24 mL, 19.34 mmol) and DMF (20 mL). The crude product was purified by column chromatography (hexane/EtOAc – 10:1 to 1:1), affording **1c** (1.59 g, 54%) as a white/beige amorphous solid.

^1^H NMR (400 MHz, CDCl_3_) δ 7.68–7.60 (m, 2H), 7.40–7.33 (m, 3H), 4.61–4.54 (m, 2H), 4.30–4.23 (m, 4H), 3.48 (s, 2H), 0.97 (s, 9H), 0.16 (s, 6H) ppm. ^13^C­{^1^H} NMR (101 MHz, CDCl_3_) δ 137.2, 129.7, 128.2 (2C), 125.5 (2C), 107.2, 76.1 (2C), 69.6, 68.5 (2C), 59.8, 26.0 (3C), 18.4, −4.4 (2C) ppm. HRMS (ESI+) *m*/*z*: calcd. for C_19_H_29_O_6_Si [M + H]^+^: 381.1728, found: 381.1729. Our physical and spectroscopic data matched previously reported data.^18^

#### 2-O-*tert*-Butyldimethylsilyl-d-*myo*-inositol-1,3,5-orthoformate (**1d**)

The title compound was synthesized according to the general procedure B, using bridged *myo*-inositol **1b** (800 mg, 4.21 mmol), TBDMSCl (666 mg, 4.42 mmol), 2,6-lutidine (1.22 mL, 10.52 mmol) and DMF (11 mL). The product was purified by column chromatography (hexane/EtOAc – 3:1 to 1:1), affording **1d** (481 mg, 38%) as a white/beige amorphous solid.

^1^H NMR (400 MHz, CDCl_3_) δ 5.50 (s, 1H), 4.63–4.55 (m, 2H), 4.31–4.23 (m, 2H), 4.19–4.12 (m, 2H), 3.30–3.24 (m, 2H), 0.95 (s, 9H), 0.16 (s, 6H) ppm. ^13^C­{^1^H} NMR (101 MHz, CDCl_3_) δ 102.6, 74.8 (2C), 68.9, 68.8 (2C), 60.7, 26.1 (3C), 18.6, −4.5 (2C) ppm. HRMS (ESI+) *m*/*z*: calcd. for C_13_H_24_NaO_6_Si [M + Na]^+^: 327.1234, found: 327.1235. Our physical and spectroscopic data matched previously reported data.^30^

#### 2-O-Trimethylsilyl-d-*myo*-inositol-1,3,5-orthobenzoate (**1e**)

The title compound was synthesized according to the general procedure B, using bridged *myo*-inositol **1a** (500 mg, 1.88 mmol), TMSCl (260 μL, 2.07 mmol), imidazole (281 mg, 4.13 mmol) and DMF (5 mL). The product was purified by column chromatography (hexane/EtOAc – 10:1 to 1:1), affording **1e** (75 mg, 12%) as a white/beige amorphous solid.

^1^H NMR (400 MHz, CDCl_3_) δ 7.70–7.61 (m, 2H), 7.41–7.31 (m, 3H), 4.72–4.63 (m, 2H), 4.38–4.34 (m, 1H), 4.34–4.27 (m, 3H), 3.34–3.25 (m, 2H), 0.22 (s, 9H) ppm. ^13^C­{^1^H} NMR (101 MHz, CDCl_3_) δ 137.1, 129.6, 128.2 (2C), 125.6 (2C), 107.2, 76.1 (2C), 69.8, 68.6 (2C), 59.6, 0.4 (3C) ppm. HRMS (ESI+) *m*/*z*: calcd. for C_16_H_22_NaO_6_Si [M + Na]^+^: 361.1078, found: 361.1076.

#### 2-O-*tert*-Butyldiphenylsilyl-d-*myo*-inositol-1,3,5-orthobenzoate (**1f**)

The title compound was synthesized according to the general procedure using bridged *myo*-inositol **1a** (500 mg, 1.88 mmol), TBDPSCl (537 μL, 2.07 mmol), imidazole (281 mg, 4.13 mmol) and DMF (5 mL). The product was purified by column chromatography (hexane/EtOAc – 10:1 to 3:1), affording **1f** (545 mg, 58%) as a white/beige amorphous solid.

^1^H NMR (400 MHz, CDCl_3_) δ 7.75–7.66 (m, 4H), 7.61–7.41 (m, 8H), 7.40–7.26 (m, 3H), 4.75–4.70 (m, 1H), 4.67–4.60 (m, 1H), 4.47–4.43 (m, 1H), 4.34–4.28 (m, 2H), 4.08 (d, *J* = 10.2 Hz, 1H), 4.05–4.00 (m, 1H), 3.20–3.12 (m, 1H), 1.12 (s, 9H) ppm. ^13^C­{^1^H} NMR (101 MHz, CDCl_3_) δ 136.6, 135.8 (2C), 135.7 (2C), 131.30, 131.26, 131.0, 130.8, 129.8, 128.5 (2C), 128.4 (2C), 128.2 (2C), 125.3 (2C), 107.2, 76.2, 75.1, 69.7, 69.3, 68.2, 60.0, 27.0 (3C), 19.1 ppm. IR (ATR): ν = 3494, 1338 (OH), 1099, 1055 (C–O) cm^–1^. HRMS (ESI+) *m*/*z*: calcd. for C_29_H_33_O_6_Si [M + H]^+^: 505.2041, found: 505.2062.

#### 2-O-Benzoyl-d-*myo*-inositol-1,3,5-orthobenzoate (**1g**)

The title compound was prepared by alternative strategy. Bridged *myo*-inositol **1a** (500 mg, 1.88 mmol) and imidazole (281 mg, 4.13 mmol, 2.2 equiv) were stirred at 0 °C in DMF (5 mL). Benzoyl chloride (240 μL, 2.07 mmol, 1.1 equiv) was added portionwise and the reaction mixture was stirred at room temperature for 2 days. The solvent was evaporated and the product was purified by column chromatography (hexane/EtOAc – 10:1 to 1:3), affording **1g** (197 mg, 28%) as a white/beige amorphous solid.

^1^H NMR (400 MHz, DMSO-*d*_*6*_) δ 8.09–7.99 (m, 2H), 7.74–7.65 (m, 1H), 7.62–7.53 (m, 4H), 7.45–7.33 (m, 3H), 5.83–5.74 (m, 2H), 5.60 (s, 1H), 4.55–4.50 (m, 2H), 4.50–4.46 (m, 2H), 4.38–4.31 (m, 1H) ppm. ^13^C­{^1^H} NMR (101 MHz, DMSO-*d*_*6*_) δ 165.2, 137.5, 133.6, 129.5, 129.33 (2C), 129.28, 129.2, 128.9 (2C), 127.8 (2C), 125.3 (2C), 106.6, 73.3 (2C), 70.5, 66.9, 63.0 ppm. HRMS (ESI+) *m*/*z*: calcd. for C_20_H_18_NaO_7_ [M + Na]^+^: 393.0945, found: 393.0944. Our physical and spectroscopic data matched previously reported data.^31^

### General Procedure C: Organocatalytic Desymmetrization

Vial (4 mL) was loaded with **1** (0.1 mmol), *pre*-**C3** (0.5 mg, 0.001 mmol), DABCO (16.8 mg, 0.15 mmol) and chlorobenzene (1.0 mL) at room temperature. Then, the corresponding carbonyl reagent **2** (0.15 mmol) was added. The reaction was stirred for an indicated time at room temperature. After completion of the reaction (monitored by TLC) the crude mixture was purified by column chromatography (mixtures of hexane/EtOAc).

*Note:* Racemic products were prepared by reactions with *pre*-**C6**.

### General Procedure D: Organocatalytic Desymmetrization Followed by Protection

Vial (4 mL) was loaded with **1** (0.1 mmol), *pre*-**C3** (0.5 mg, 0.001 mmol), DABCO (16.8 mg, 0.15 mmol) and chlorobenzene (1.0 mL) at room temperature. Then, the corresponding carbonyl reagent **2** (0.15 mmol) was added. The reaction was stirred for an indicated time at room temperature. After completion of the reaction (monitored by TLC) the major impurities were separated by column chromatography (eluted by hexane/EtOAc 3:1). The obtained product with minor impurity was then mixed with Ac_2_O (1.2 equiv), Et_3_N (1.2 equiv), DMAP (10 mol %) in dry THF (10 mL/mmol) in a small vial (4 mL) at 0 °C. The reaction was stirred at room temperature until full conversion (monitored by TLC). Purifying by column chromatography (mixtures of hexane/EtOAc) afforded the product.

*Note:* Racemic products were prepared by reactions with pre-**C6**.

#### (1R,3R,5S,6R,7S,8R,9S)-8-((*tert*-Butyldimethylsilyl)­oxy)-9-hydroxy-3-phenyl-2,4,10-trioxaadamantan-6-yl cinnamate (**3a**)

The title compound was synthesized according to the general procedure C (reaction time: 18 h), using **1c** (38.1 mg, 0.1 mmol) and (2*Z*)-2-bromo-3-phenyl-2-propenal (31.7 mg, 0.15 mmol). The crude product was purified by column chromatography (hexane/EtOAc – 10:1 to 5:1), affording **3a** (33.0 mg, 65%) as a white amorphous solid.

*Note:* Crystals suitable for X-ray analysis (*er* = 99.7:0.3) were obtained by slow evaporation of *i*-PrOH at room temperature.

*Er* = 85.3:14.7 (*ee* = 70%), the enantiomeric excess of product **3a** was determined by high-performance liquid chromatography (HPLC) using a Chiralpak IA column (*n*-heptane/*i*-PrOH – 95:5, flow rate = 1.0 mL/min, λ = 275 nm, *t* = 25 °C): *t*_R_ = 13.4 min (minor), *t*_R_ = 17.5 min (major). [α]_D_^20^ = −18.3 (*c* = 0.4, CHCl_3_). ^1^H NMR (400 MHz, CDCl_3_) δ 7.75 (d, *J* = 16.0 Hz, 1H), 7.72–7.66 (m, 2H), 7.58–7.51 (m, 2H), 7.48–7.32 (m, 6H), 6.42 (d, *J* = 16.0 Hz, 1H), 5.79–5.74 (m, 1H), 4.72–4.67 (m, 1H), 4.60–4.55 (m, 1H), 4.46–4.41 (m, 1H), 4.37–4.30 (m, 2H), 2.42 (bs, 1H), 0.97 (s, 9H), 0.17 (s, 3H), 0.17 (s, 3H) ppm. ^13^C­{^1^H} NMR (101 MHz, CDCl_3_) δ 165.3, 147.2, 137.2, 133.9, 131.1, 129.6, 129.2 (2C), 128.5 (2C), 128.1 (2C), 125.6 (2C), 116.4, 107.7, 76.0, 73.7, 69.4, 69.2, 68.1, 60.3, 26.0 (2C), 18.5, −4.4, −4.5. IR (ATR): ν = 3489 (OH), 1712 (C=O, ester), 1633 (C=C), 1101 (C–O, alcohol) cm^–1^. HRMS (ESI+) *m*/*z*: calcd. for C_28_H_35_O_7_Si [M + H]^+^: 511.2147, found: 511.2138.

#### (1S,3S,5R,6S,7R,8S,9R)-8-((*tert*-Butyldimethylsilyl)­oxy)-9-hydroxy-3-phenyl-2,4,10-trioxaadamantan-6-yl cinnamate (ent-**3a**)

The title compound was synthesized according to the general procedure C (reaction time: 18 h), using **1c** (38.1 mg, 0.1 mmol) and (2*Z*)-2-bromo-3-phenyl-2-propenal (31.7 mg, 0.15 mmol) and *ent-pre*-**C4** (0.5 mg, 0.001 mmol) instead *pre*- **C4**. The crude product was purified by column chromatography (hexane/EtOAc – 10:1 to 5:1), affording *ent*-**3a** (47.0 mg, 92%) as a white amorphous solid.

*Er* = 87.9:12.1 (*ee* = 76%), the enantiomeric excess of product *ent*-**3a** was determined by HPLC using a Chiralpak IA column (*n*-heptane/*i*-PrOH – 95:5, flow rate = 1.0 mL/min, λ = 275 nm, *t* = 25 °C): *t*_R_ = 13.4 min (major), *t*_R_ = 17.5 min (minor). [α]_D_^20^ = +16.7 (*c* = 0.5, CHCl_3_). Other analytical data agree with the data of the opposite enantiomer (**3a**).

#### (1R,3R,5S,6R,7S,8R,9S)-8-((*tert*-Butyldimethylsilyl)­oxy)-9-hydroxy-2,4,10-trioxaadamantan-6-yl cinnamate (**3b**)

The title compound was synthesized according to the general procedure C (reaction time: 18 h), using **1d** (30.4 mg, 0.1 mmol) and (2*Z*)-2-bromo-3-phenyl-2-propenal (31.7 mg, 0.15 mmol). The crude product was purified by column chromatography (hexane/EtOAc – 10:1 to 3:1), affording **3b** (21.0 mg, 48%) as a white amorphous solid.

*Er* = 73.2:26.8 (*ee* = 46%), the enantiomeric excess of product **3b** was determined by HPLC using a Chiralpak IA column (*n*-heptane/*i*-PrOH – 95:5, flow rate = 1.0 mL/min, λ = 277 nm, *t* = 25 °C): *t*_R_ = 15.8 min (minor), *t*_R_ = 19.7 min (major). [α]_D_^20^ = −16.9 (*c* = 0.7, CHCl_3_). ^1^H NMR (400 MHz, CDCl_3_) δ 7.72 (d, *J* = 15.9 Hz, 1H), 7.56–7.51 (m, 2H), 7.47–7.36 (m, 3H), 6.38 (d, *J* = 15.9 Hz, 1H), 5.68–5.63 (m, 1H), 5.57 (d, *J* = 1.3 Hz, 1H), 4.64–4.58 (m, 1H), 4.48–4.43 (m, 1H), 4.31–4.24 (m, 2H), 4.19–4.15 (m, 1H), 2.38 (d, *J* = 7.2 Hz, 1H), 0.95 (s, 9H), 0.164 (s, 3H), 0.160 (s, 3H) ppm. ^13^C­{^1^H} NMR (101 MHz, CDCl_3_) δ 165.1, 147.2, 133.9, 131.1, 129.2 (2C), 128.5 (2C), 116.3, 103.0, 74.7, 72.3, 69.4, 68.4, 68.2, 61.2, 26.1 (3C), 18.6, −4.52, −4.49 ppm. IR (ATR): ν = 3492 (OH), 1714 (C=O, ester), 1635, 984 (C=C) cm^–1^. HRMS (ESI+) *m*/*z*: calcd. for C_22_H_31_O_7_Si [M + H]^+^: 435.1834, found: 435.1844.

#### (1R,3R,5S,6R,7S,8S,9R)-8-Hydroxy-3-phenyl-9-((trimethylsilyl)­oxy)-2,4,10-trioxaadamantan-6-yl cinnamate (**3c**)

The title compound was synthesized according to the general procedure C (reaction time: 22 h), using **1e** (33.8 mg, 0.1 mmol) and (2*Z*)-2-bromo-3-phenyl-2-propenal (31.7 mg, 0.15 mmol). The crude product was purified by column chromatography (hexane/EtOAc – 10:1 to 3:1), affording **3c** (10.0 mg, 21%) as a white amorphous solid.

*Er* = 85.1:14.9 (*ee* = 70%), the enantiomeric excess of product **3c** was determined by HPLC using a Chiralpak IA column (*n*-heptane/*i*-PrOH – 95:5, flow rate = 1.0 mL/min, λ = 277 nm, *t* = 25 °C): *t*_R_ = 15.8 min (minor), *t*_R_ = 23.3 min (major). [α]_D_^20^ = −15.5 (*c* = 0.3, CHCl_3_). ^1^H NMR (400 MHz, CDCl_3_) δ 7.76 (d, *J* = 16.0 Hz, 1H), 7.71–7.67 (m, 2H), 7.58–7.53 (m, 2H), 7.45–7.34 (m, 6H), 6.43 (d, *J* = 15.9 Hz, 1H), 5.81–5.77 (m, 1H), 4.76–4.70 (m, 1H), 4.60–4.56 (m, 1H), 4.47–4.42 (m, 1H), 4.38–4.34 (m, 1H), 4.32–4.29 (m, 1H), 2.40 (d, *J* = 7.3 Hz, 1H), 0.22 (s, 9H) ppm. ^13^C­{^1^H} NMR (101 MHz, CDCl_3_) δ 165.2, 147.3, 137.0, 134.0, 131.2, 129.7, 129.2 (2C), 128.5 (2C), 128.1 (2C), 125.6 (2C), 116.3, 107.7, 76.0, 73.6, 69.4, 69.3, 68.1, 60.1, 0.4 (3C) ppm. IR (ATR): ν = 3379 (OH), 1712 (C=O, ester), 1633, 976 (C=C), 1101 (C–O, alcohol) cm^–1^. HRMS (ESI+) *m*/*z*: calcd. for C_25_H_29_O_7_Si [M + H]^+^: 469.1677, found: 469.1689.

#### (1R,3R,5S,6R,7S,8R,9S)-8-((*tert*-Butyldiphenylsilyl)­oxy)-9-hydroxy-3-phenyl-2,4,10-trioxaadamantan-6-yl cinnamate (**3d**)

The title compound was synthesized according to the general procedure C (reaction time: 42 h), using **1f** (50.5 mg, 0.1 mmol) and (2*Z*)-2-bromo-3-phenyl-2-propenal (31.7 mg, 0.15 mmol). The crude product was purified by column chromatography (hexane/EtOAc – 10:1 to 5:1), affording **3d** (28.0 mg, 44%) as a white amorphous solid.

*Er* = 71.6:28.4 (*ee* = 43%), the enantiomeric excess of product **3d** was determined by HPLC using a Chiralpak IA column (*n*-heptane/*i*-PrOH – 97:3, flow rate = 1.0 mL/min, λ = 190 nm, *t* = 25 °C): *t*_R_ = 13.4 min (minor), *t*_R_ = 16.1 min (major). [α]_D_^20^ = +33.0 (*c* = 1.0, CHCl_3_). ^1^H NMR (400 MHz, CDCl_3_) δ 7.83 (d, *J* = 16.0 Hz, 1H), 7.80–7.76 (m, 2H), 7.74–7.69 (m, 2H), 7.61–7.38 (m, 14H), 7.35–7.30 (m, 2H), 6.61 (d, *J* = 16.0 Hz, 1H), 5.77–5.73 (m, 1H), 4.77–4.68 (m, 2H), 4.65–4.60 (m, 1H), 4.46–4.41 (m, 1H), 4.27–4.18 (m, 2H), 1.17 (s, 9H) ppm. ^13^C­{^1^H} NMR (101 MHz, CDCl_3_) δ 166.5, 146.0, 136.8, 136.1 (2C), 135.7 (2C), 134.5, 131.4, 131.15, 131.0, 130.8, 130.6, 129.7, 129.1 (2C), 128.41 (2C), 128.38 (4C), 128.2 (2C), 125.4 (2C), 117.8, 107.2, 74.1, 72.7, 69.8 (2C), 68.5, 62.1, 27.1 (3C), 19.1 ppm. IR (ATR): ν = 3490 (OH), 1712 (C=O, ester), 1635, 978 (C=C), 1101 (C–O, alcohol) cm^–1^. HRMS (ESI+) *m*/*z*: calcd. for C_38_H_39_O_7_Si [M + H]^+^: 635.2460, found: 635.2470.

#### (1S,3R,5R,6R,7S,8R,9S)-8-(Cinnamoyloxy)-9-hydroxy-3-phenyl-2,4,10-trioxaadamantan-6-yl benzoate (**3e**)

The title compound was synthesized according to the general procedure C (reaction time: 18 h), using **1g** (37.0 mg, 0.1 mmol) and (2*Z*)-2-bromo-3-phenyl-2-propenal (31.7 mg, 0.15 mmol). The crude product was purified by column chromatography (hexane/EtOAc – 10.1 to 3:1), affording 3e (30.0 mg, 60%) as a white amorphous solid.

*Er* = 53.3:46.7 (*ee* = 7%), the enantiomeric excess of product **3e** was determined by HPLC using a Chiralpak IA column (*n*-heptane/*i*-PrOH – 90:10, flow rate = 1.0 mL/min, λ = 276 nm, *t* = 25 °C): *t*_R_ = 16.9 min (major), *t*_R_ = 21.5 min (minor). [α]_D_^20^ = −6.3 (*c* = 0.7, CHCl_3_). ^1^H NMR (400 MHz, CDCl_3_) δ 8.19–8.13 (m, 2H), 7.79 (d, *J* = 16.0 Hz, 1H), 7.72 (dd, *J* = 6.7, 3.0 Hz, 2H), 7.62–7.51 (m, 3H), 7.52–7.37 (m, 8H), 6.52 (d, *J* = 16.0 Hz, 1H), 5.93–5.86 (m, 1H), 5.73–5.68 (m, 1H), 4.88–4.81 (m, 1H), 4.80–4.76 (m, 1H), 4.76–4.72 (m, 1H), 4.72–4.66 (m, 1H), 2.84 (d, *J* = 6.6 Hz, 1H) ppm. ^13^C­{^1^H} NMR (101 MHz, CDCl_3_) δ 166.4, 165.5, 147.4, 136.8, 134.0, 133.6, 131.0, 130.1 (2C), 129.9, 129.7, 129.1 (2C), 128.62 (2C), 128.55 (2C), 128.3, 125.5, 116.4, 107.8, 73.3, 71.1, 69.4, 68.6, 67.7, 63.0 ppm. IR (ATR): ν = 3485 (OH), 1712, 1693 (C=O, ester), 1635, 968 (C=C), 1103 (C–O, alcohol) cm^–1^. HRMS (ESI+) *m*/*z*: calcd. for C_29_H_25_O_8_ [M + H]^+^: 501.1544, found: 501.1554.

#### (1R,3R,5S,6R,7S,8R,9S)-9-Hydroxy-3-phenyl-2,4,10-trioxaadamantane-6,8-diyl (2E,2’E)-bis­(3-phenyl acrylate) (**3f**)

The title compound was synthesized according to the general procedure C (reaction time: 20 h), using **1a** (26.6 mg, 0.1 mmol) and (2*Z*)-2-bromo-3-phenyl-2-propenal (52.8 mg, 0.25 mmol). The crude product was purified by column chromatography (hexane/EtOAc – 10:1 to 1:1), affording **3f** (12.0 mg, 23%) as a beige amorphous solid.

*Er* = 72.4:27.6 (*ee* = 45%), the enantiomeric excess of product **3f** was determined by HPLC using a Chiralpak IA column (*n*-heptane/*i*-PrOH – 90:10, flow rate = 1.0 mL/min, λ = 276 nm, *t* = 25 °C): *t*_R_ = 23.3 min (major), *t*_R_ = 43.4 min (minor). [α]_D_^20^ = – 45.8 (*c* = 0.5, CHCl_3_) ^1^H NMR (400 MHz, CDCl_3_) δ 7.80 (d, *J* = 16.0 Hz, 1H), 7.80 (d, *J* = 15.9 Hz, 1H), 7.75–7.68 (m, 2H), 7.55 (dq, *J* = 7.7, 2.8 Hz, 4H), 7.41 (dh, *J* = 6.3, 3.0 Hz, 9H), 6.61 (d, *J* = 16.0 Hz, 1H), 6.49 (d, *J* = 15.9 Hz, 1H), 5.90–5.84 (m, 1H), 5.59–5.54 (m, 1H), 4.84–4.77 (m, 1H), 4.73–4.66 (m, 2H), 4.66–4.61 (m, 1H), 2.56 (bs, 1H) ppm. ^13^C­{^1^H} NMR (101 MHz, CDCl_3_) δ 166.7, 165.3, 147.5, 146.3, 136.7, 134.3, 134.0, 131.1, 130.7, 129.9, 129.15 (2C), 129.08 (2C), 128.6 (2C), 128.4 (2C), 128.3 (2C), 125.5 (2C), 117.6, 116.3, 107.8, 73.4, 71.1, 69.4, 68.6, 67.8, 62.4 ppm. IR (ATR): ν = 3473 (OH), 1709 (C=O, ester), 1633, 976 (C=C), 1101 (C–O, alcohol) cm^–1^. HRMS (ESI+) *m*/*z*: calcd. for C_31_H_27_O_8_ [M + H]^+^: 527.1700, found: 527.1714.

#### (1R,3R,5S,6R,7S,8R,9S)-8-((*tert*-Butyldimethylsilyl)­oxy)-9-hydroxy-3-phenyl-2,4,10-trioxaadamantan-6-yl (E)-3-(naphthalen-2-yl)­acrylate (**3g**)

The title compound was synthesized according to the general procedure C (reaction time: 18 h), using **1c** (38.1 mg, 0.1 mmol) and (2*Z*)-2-bromo-3-(naphtalen-2-yl)-2-propenal (39.2 mg, 0.15 mmol). The crude product was purified by column chromatography (hexane/EtOAc – 10:1 to 3:1), affording **3g** (35.0 mg, 62%) as a white amorphous solid.

*Er* = 81.8:18.2 (*ee* = 64%), the enantiomeric excess of product **3g** was determined by HPLC using a Chiralpak IA column (*n*-heptane/*i*-PrOH – 95:5, flow rate = 1.0 mL/min, λ = 271 nm, *t* = 25 °C): *t*_R_ = 17.2 min (minor), *t*_R_ = 24.5 min (major). [α]_D_^20^ = −18.7 (*c* = 0.6, CHCl_3_) ^1^H NMR (400 MHz, CDCl_3_) δ 7.97–7.79 (m, 5H), 7.75–7.63 (m, 3H), 7.60–7.49 (m, 2H), 7.40–7.36 (m, 3H), 6.53 (d, *J* = 15.9 Hz, 1H), 5.84–5.78 (m, 1H), 4.76–4.69 (m, 1H), 4.64–4.58 (m, 1H), 4.49–4.43 (m, 1H), 4.39–4.33 (m, 2H), 2.48 (s, 1H), 0.98 (s, 9H), 0.185 (s, 3H), 0.182 (s, 3H) ppm. ^13^C­{^1^H} NMR (101 MHz, CDCl_3_) δ 165.3, 147.3, 137.2, 134.6, 133.3, 131.4, 130.8, 129.6, 129.0, 128.8, 128.2 (2C), 128.0, 127.8, 127.1, 125.6 (2C), 123.5, 116.4, 107.7, 76.0, 73.7, 69.5, 69.3, 68.2, 60.3, 26.0 (3C), 18.5, −4.45, −4.35 ppm. IR (ATR): ν = 3452 (OH), 1709 (C=O, ester), 1630, 958 (C=C), 1099 (C–O, alcohol) cm^–1^. HRMS (ESI+) *m*/*z*: calcd. for C_32_H_36_NaO_7_Si [M + Na]^+^: 583.2123, found: 583.2114.

#### (1R,3R,5S,6R,7S,8R,9S)-8-((*tert*-Butyldimethylsilyl)­oxy)-9-hydroxy-3-phenyl-2,4,10-trioxaadamantan-6-yl (E)-3-(p-tolyl)­acrylate (**3h**)

The title compound was synthesized according to the general procedure C (reaction time: 18 h), using **1c** (38.1 mg, 0.1 mmol) and (2*Z*)-2-bromo-3-(4-methylphenyl)-2-propenal (33.8 mg, 0.15 mmol). The crude product was purified by column chromatography (hexane/EtOAc – 7:1 to 3:1), affording **3h** (17.0 mg, 32%) as a white amorphous solid.

*Er* = 82.3:17.7 (*ee* = 65%), the enantiomeric excess of product **3h** was determined by HPLC using a Chiralpak IA column (*n*-heptane/*i*-PrOH – 95:5, flow rate = 1.0 mL/min, λ = 284 nm, *t* = 25 °C): *t*_R_ = 17.1 min (minor), *t*_R_ = 20.6 min (major). [α]_D_^20^ = −15.8 (*c* = 0.7, CHCl_3_) ^1^H NMR (400 MHz, CDCl_3_) δ 7.72 (d, *J* = 15.9 Hz, 1H), 7.71–7.65 (m, 2H), 7.44 (d, *J* = 8.0 Hz, 2H), 7.39–7.35 (m, 3H), 7.22 (d, *J* = 7.9 Hz, 2H), 6.37 (d, *J* = 15.9 Hz, 1H), 5.80–5.75 (m, 1H), 4.73–4.66 (m, 1H), 4.60–4.54 (m, 1H), 4.46–4.40 (m, 1H), 4.37–4.32 (m, 1H), 4.32–4.29 (m, 1H), 2.39 (s, 3H), 1.61 (bs, 1H), 0.96 (s, 9H), 0.164 (s, 3H), 0.160 (s, 3H) ppm. ^13^C­{^1^H} NMR (101 MHz, CDCl_3_) δ 165.4, 147.3, 141.8, 137.2, 131.2, 129.9 (2C), 129.6, 128.5 (2C), 128.2 (2C), 125.6 (2C), 115.1, 107.7, 76.0, 73.6, 69.4, 69.2, 68.2, 60.3, 26.0 (3C), 21.7, 18.5, −4.4, −4.5 ppm. IR (ATR): ν = 3500 (OH), 1712 (C=O, ester), 1633, 960 (C=C), 1101 (C–O, alcohol) cm^–1^. HRMS (ESI+) *m*/*z*: calcd. for C_29_H_37_O_7_Si [M + H]^+^: 525.2303, found: 525.2322.

#### (1R,3R,5S,6R,7S,8R,9S)-8-((*tert*-Butyldimethylsilyl)­oxy)-9-hydroxy-3-phenyl-2,4,10-trioxaadamantan-6-yl (E)-3-(4-methoxyphenyl)­acrylate (**3j**)

The title compound was synthesized according to the general procedure C (reaction time: 18 h), using **1c** (38.1 mg, 0.1 mmol) and (2*Z*)-2-bromo-3-(4-trifluoromethylphenyl)-2-propenal (41.9 mg, 0.15 mmol). The crude product was purified by column chromatography (hexane/EtOAc – 5:1), affording **3j** (34.0 mg, 59%) as a white amorphous solid.

*Er* = 81.9:18.1 (*ee* = 64%), the enantiomeric excess of product **3j** was determined by HPLC using a Chiralpak IA column (*n*-heptane/*i*-PrOH – 95:5, flow rate = 1.0 mL/min, λ = 271 nm, *t* = 25 °C): *t*_R_ = 10.6 min (minor), *t*_R_ = 15.1 min (major). [α]_D_^20^ = −13.3 (*c* = 0.4, CHCl_3_. ^1^H NMR (400 MHz, CDCl_3_) δ 7.75 (d, *J* = 16.0 Hz, 1H), 7.70–7.62 (m, 6H), 7.41–7.33 (m, 3H), 6.49 (d, *J* = 16.0 Hz, 1H), 5.79–5.74 (m, 1H), 4.75–4.69 (m, 1H), 4.63–4.57 (m, 1H), 4.46–4.40 (m, 1H), 4.36–4.30 (m, 2H), 2.34 (d, *J* = 6.9 Hz, 1H), 0.97 (s, 9H), 0.17 (s, 3H), 0.16 (s, 3H) ppm. ^13^C­{^1^H} NMR (101 MHz, CDCl_3_) δ 164.9, 145.1, 137.3, 137.1, 132.5 (q, *J* = 32.8 Hz), 129.7, 128.6 (2C), 128.2 (2C), 126.1 (q, *J* = 3.7 Hz, 2C), 125.6 (2C), 123.8 (q, *J* = 272.3 Hz), 119.1, 107.7, 76.0, 73.6, 69.7, 69.2, 68.0, 60.3, 26.0 (3C), 18.5, −4.4, −4.5 ppm. ^19^F NMR (376 MHz, CDCl_3_) δ – 62.94 (s, 3F). IR (ATR): ν = 3502 (OH), 1712 (C=O, ester), 1635, 958 (C=C), 1315 (C–F), 1101 (C–O, alcohol) cm^–1^. HRMS (ESI+) *m*/*z*: calcd. for C_29_H_33_F_3_NaO_7_Si [M + Na]^+^: 601.1840, found: 601.1821.

#### (1R,3R,5S,6R,7S,8R,9S)-8-((*tert*-butyldimethylsilyl)­oxy)-9-hydroxy-3-phenyl-2,4,10-trioxaadamantan-6-yl (E)-3-(4-fluorophenyl)­acrylate (**3l**)

The title compound was synthesized according to the general procedure C (reaction time: 18 h), using **1c** (38.1 mg, 0.1 mmol) and (2*Z*)-2-bromo-3-(4-fluorophenyl)-2-propenal (34.4 mg, 0.15 mmol). The crude product was purified by column chromatography (hexane/EtOAc – 8:1 to 3:1), affording **3l** (29.0 mg, 55%) as a white amorphous solid.

*Er* = 85.8:14.2 (*ee* = 72%), the enantiomeric excess of product **3l** was determined by HPLC using a Chiralpak IA column (*n*-heptane/*i*-PrOH – 95:5, flow rate = 1.0 mL/min, λ = 190 nm, *t* = 25 °C): *t*_R_ = 12.5 min (minor), *t*_R_ = 16.3 min (major). [α]_D_^20^ = −18.8 (*c* = 0.4, CHCl_3_). ^1^H NMR (400 MHz, CDCl_3_) δ 7.75–7.63 (m, 3H), 7.57–7.48 (m, 2H), 7.41–7.33 (m, 3H), 7.10 (t, *J* = 8.5 Hz, 2H), 6.34 (d, *J* = 15.9 Hz, 1H), 5.79–5.74 (m, 1H), 4.74–4.67 (m, 1H), 4.60–4.55 (m, 1H), 4.45–4.40 (m, 1H), 4.36–4.32 (m, 1H), 4.32–4.29 (m, 1H), 2.35 (s, 1H), 0.97 (s, 9H), 0.17 (s, 3H), 0.16 (s, 3H) ppm. ^13^C­{^1^H} NMR (101 MHz, CDCl_3_) δ 165.2, 164.4 (d, *J* = 252.7 Hz), 145.8, 137.1, 130.4 (d, *J* = 8.6 Hz, 2C), 130.2 (d, *J* = 3.4 Hz), 129.6, 128.2 (2C), 125.6 (2C), 116.4 (d, *J* = 22.0 Hz, 2C), 116.1 (d, *J* = 2.5 Hz), 107.7, 76.0, 73.6, 69.5, 69.2, 68.1, 60.3, 26.0 (3C), 18.5, −4.4, −4.5 ppm. ^19^F NMR (376 MHz, CDCl_3_) δ – 108.23 (tt, *J* = 8.2, 5.2 Hz, 1F). IR (ATR): ν = 3500 (OH), 1716 (C=O, ester), 1635, 962 (C=C), 1325 (C–F), 1101 (C–O, alcohol) cm^–1^. HRMS (ESI+) *m*/*z*: calcd. for C_28_H_33_FNaO_7_Si [M + Na]^+^: 551.1872, found: 551.1862.

#### (1R,3R,5S,6R,7S,8R,9S)-8-((*tert*-Butyldimethylsilyl)­oxy)-9-hydroxy-3-phenyl-2,4,10-trioxaadamantan-6-yl (E)-3-(4-chlorophenyl)­acrylate (**3m**)

The title compound was synthesized according to the general procedure C (reaction time: 28 h), using **1c** (38.1 mg, 0.1 mmol) and (2*Z*)-2-bromo-3-(4-chlorophenyl)-2-propenal (36.8 mg, 0.15 mmol). The crude product was purified by column chromatography (hexane/EtOAc – 7:1 to 3:1), affording **3m** (34.0 mg, 62%) as a white amorphous solid.

*Er* = 82.7:17.3 (*ee* = 65%), the enantiomeric excess of product **3m** was determined by HPLC using a Chiralpak IA column (*n*-heptane/*i*-PrOH – 95:5, flow rate = 1.0 mL/min, λ = 283 nm, *t* = 25 °C): *t*_R_ = 12.7 min (minor), *t*_R_ = 17.1 min (major). [α]_D_^20^ = −16.5 (*c* = 0.7, CHCl_3_) ^1^H NMR (400 MHz, CDCl_3_) δ 7.73–7.64 (m, 3H), 7.49–7.45 (m, 2H), 7.42–7.33 (m, 5H), 6.39 (dd, *J* = 16.0, 1.2 Hz, 1H), 5.78–5.73 (m, 1H), 4.74–4.68 (m, 1H), 4.61–4.55 (m, 1H), 4.45–4.39 (m, 1H), 4.36–4.29 (m, 2H), 2.29 (bs, 1H), 0.96 (s, 9H), 0.164 (s, 3H), 0.161 (s, 3H) ppm. ^13^C­{^1^H} NMR (101 MHz, CDCl_3_) δ 165.1, 145.7, 137.1, 137.1, 132.4, 129.63 (2C), 129.62, 129.5 (2C), 128.2 (2C), 125.6 (2C), 117.0, 107.7, 76.0, 73.6, 69.5, 69.2, 68.1, 60.3, 26.0 (3C), 18.5, −4.4, −4.5 ppm. IR (ATR): ν = 3508 (OH), 1712 (C=O, ester), 1633, 960 (C=C), 1101 (C–O, alcohol) cm^–1^. HRMS (ESI+) *m*/*z*: calcd. for C_28_H_33_ClNaO_7_Si [M + Na]^+^: 567.1576, found: 567.1564.

#### (1R,3R,5S,6R,7S,8R,9S)-8-((*tert*-Butyldimethylsilyl)­oxy)-9-hydroxy-3-phenyl-2,4,10-trioxaadamantan-6-yl (E)-3-(4-bromophenyl)­acrylate (**3n**)

The title compound was synthesized according to the general procedure C (reaction time: 18 h), using **1c** (38.1 mg, 0.1 mmol) and (2*Z*)-2-bromo-3-(4-bromophenyl)-2-propenal (43.5 mg, 0.15 mmol). The crude product was purified by column chromatography (hexane/EtOAc – 10:1 to 6:1), affording **3n** (39.0 mg, 66%) as a white amorphous solid.

*Er* = 84.6:15.4 (*ee* = 69%), the enantiomeric excess of product **3n** was determined by HPLC using a Chiralpak IA column (*n*-heptane/*i*-PrOH – 95:5, flow rate = 1.0 mL/min, λ = 286 nm, *t* = 25 °C): *t*_R_ = 13.0 min (minor), *t*_R_ = 18.1 min (major). [α]_D_^20^ = −23.6 (*c* = 0.5, CHCl_3_) ^1^H NMR (400 MHz, CDCl_3_) δ 7.70–7.64 (m, 3H), 7.57–7.52 (m, 2H), 7.42–7.35 (m, 5H), 6.40 (d, *J* = 16.0 Hz, 1H), 5.79–5.73 (m, 1H), 4.73–4.67 (m, 1H), 4.60–4.55 (m, 1H), 4.44–4.39 (m, 1H), 4.36–4.29 (m, 2H), 2.39 (bs, 1H), 0.96 (s, 9H), 0.164 (s, 3H), 0.160 (s, 3H) ppm. ^13^C­{^1^H} NMR (101 MHz, CDCl_3_) δ 165.1, 145.7, 137.1, 132.8, 132.4 (2C), 129.8 (2C), 129.6, 128.2 (2C), 125.6 (2C), 125.5, 117.1, 107.7, 76.0, 73.6, 69.6, 69.2, 68.1, 60.3, 26.0 (3C), 18.5, −4.4, −4.5 ppm. IR (ATR): ν = 3475 (OH), 1712 (C=O, ester), 1633, 982 (C=C), 1101 (C–O, alcohol) cm^–1^. HRMS (ESI+) *m*/*z*: calcd. for C_28_H_33_BrNaO_7_Si [M + Na]^+^: 611.1071, found: 611.1057.

#### (1R,3R,5S,6R,7S,8R,9S)-8-((*tert*-Butyldimethylsilyl)­oxy)-9-hydroxy-3-phenyl-2,4,10-trioxaadamantan-6-yl (E)-3-(3-chlorophenyl)­acrylate (**3o**)

The title compound was synthesized according to the general procedure C (reaction time: 18 h), using **1c** (38.1 mg, 0.1 mmol) and (2*Z*)-2-bromo-3-(3-chlorophenyl)-2-propenal (36.8 mg, 0.15 mmol). The crude product was purified by column chromatography (hexane/EtOAc – 7:1 to 3:1), affording **3o** (31.0 mg, 57%) as a white amorphous solid.

*Er* = 87.3:12.7 (*ee* = 75%), the enantiomeric excess of product **3o** was determined by HPLC using a Chiralpak IA column (*n*-heptane/*i*-PrOH – 95:5, flow rate = 1.0 mL/min, λ = 209 nm, *t* = 25 °C): *t*_R_ = 10.1 min (minor), *t*_R_ = 13.7 min (major). [α]_D_^20^ = −19.8 (*c* = 1.4, CHCl_3_) ^1^H NMR (400 MHz, CDCl_3_) δ 7.71–7.64 (m, 3H), 7.53 (t, *J* = 1.8 Hz, 1H), 7.44–7.31 (m, 6H), 6.42 (d, *J* = 15.9 Hz, 1H), 5.79–5.74 (m, 1H), 4.76–4.68 (m, 1H), 4.61–4.56 (m, 1H), 4.44–4.40 (m, 1H), 4.36–4.29 (m, 2H), 2.31 (bs, 1H), 0.97 (s, 9H), 0.17 (s, 3H), 0.16 (s, 3H) ppm. ^13^C­{^1^H} NMR (101 MHz, CDCl_3_) δ 164.9, 145.5, 137.1, 135.8, 135.2, 130.9, 130.4, 129.6, 128.2 (2C), 128.1, 126.7, 125.6 (2C), 117.9, 107.7, 76.0, 73.6, 69.6, 69.2, 68.1, 60.3, 26.0 (3C), 18.5, −4.4, −4.5 ppm. IR (ATR): ν = 3508 (OH), 1714 (C=O, ester), 1633, 989 (C=C), 1101 (C–O, alcohol) cm^–1^. HRMS (ESI+) *m*/*z*: calcd. for C_28_H_33_ClNaO_7_Si [M + Na]^+^: 567.1576, found: 567.1562.

#### (1R,3R,5S,6R,7S,8R,9S)-8-((*tert*-Butyldimethylsilyl)­oxy)-9-hydroxy-3-phenyl-2,4,10-trioxaadamantan-6-yl (E)-3-(2-chlorophenyl)­acrylate (**3p**)

The title compound was synthesized according to the general procedure C (reaction time: 18 h), using **1c** (38.1 mg, 0.1 mmol) and (2*Z*)-2-bromo-3-(2-chlorophenyl)-2-propenal (36.8 mg, 0.15 mmol). The crude product was purified by column chromatography (hexane/EtOAc – 7:1 to 3:1), affording **3p** (41.0 mg, 75%) as a white amorphous solid.

*Er* = 87.3:12.7 (*ee* = 75%), the enantiomeric excess of product **3p** was determined by HPLC using a Chiralpak IA column (*n*-heptane/*i*-PrOH – 95:5, flow rate = 1.0 mL/min, λ = 276 nm, *t* = 25 °C): *t*_R_ = 13.4 min (minor), *t*_R_ = 15.9 min (major). [α]_D_^20^ = −18.3 (*c* = 0.4, CHCl_3_). ^1^H NMR (400 MHz, CDCl_3_) δ 8.17 (d, *J* = 16.0 Hz, 1H), 7.71–7.67 (m, 2H), 7.64 (dd, *J* = 7.7, 1.8 Hz, 1H), 7.44 (dd, *J* = 7.9, 1.5 Hz, 1H), 7.41–7.34 (m, 4H), 7.34–7.28 (m, 1H), 6.41 (d, *J* = 16.0 Hz, 1H), 5.81–5.74 (m, 1H), 4.75–4.68 (m, 1H), 4.63–4.57 (m, 1H), 4.46–4.40 (m, 1H), 4.37–4.32 (m, 2H), 2.32 (bs, 1H), 0.96 (s, 9H), 0.17 (s, 3H), 0.16 (s, 3H) ppm. ^13^C­{^1^H} NMR (101 MHz, CDCl_3_) δ 164.8, 142.6, 137.2, 135.4, 132.2, 131.8, 130.4, 129.6, 128.2 (2C), 127.8, 127.4, 125.6 (2C), 119.1, 107.7, 76.0, 73.7, 69.5, 69.2, 68.1, 60.3, 26.0 (3C), 18.4, −4.4, −4.5. IR (ATR): ν = 3471 (OH), 1697 (C=O, ester), 1633, 989 (C=C), 1101 (C–O, alcohol) cm^–1^. HRMS (ESI+) *m*/*z*: calcd. for C_28_H_33_ClNaO_7_Si [M + Na]^+^: 567.1576, found: 567.1561.

#### (1R,3R,5S,6R,7S,8R,9S)-8-((*tert*-Butyldimethylsilyl)­oxy)-9-hydroxy-3-phenyl-2,4,10-trioxaadamantan-6-yl (E)-3-(2-chlorophenyl)­acrylate (**3q**)

The title compound was synthesized according to the general procedure C (reaction time: 18 h), using **1c** (38.1 mg, 0.1 mmol) and (2*Z*)-2-bromo-3-(3-bromophenyl)-2-propenal (43.5 mg, 0.15 mmol). The crude product was purified by column chromatography (hexane/EtOAc – 10:1 to 6:1), affording **3q** (46.0 mg, 78%) as a white amorphous solid.

*Er* = 86.7:13.3 (*ee* = 73%), the enantiomeric excess of product **3q** was determined by HPLC using a Chiralpak IA column (*n*-heptane/*i*-PrOH – 95:5, flow rate = 1.0 mL/min, λ = 275 nm, *t* = 25 °C): *t*_R_ = 13.2 min (minor), *t*_R_ = 15.9 min (major). [α]_D_^20^ = −10.0 (*c* = 0.6, CHCl_3_) ^1^H NMR (400 MHz, CDCl_3_) δ 8.12 (d, *J* = 15.9 Hz, 1H), 7.72–7.66 (m, 2H), 7.66–7.59 (m, 2H), 7.43–7.30 (m, 4H), 7.30–7.24 (m, 1H), 6.37 (d, *J* = 15.9 Hz, 1H), 5.81–5.75 (m, 1H), 4.75–4.69 (m, 1H), 4.62–4.56 (m, 1H), 4.45–4.40 (m, 1H), 4.37–4.32 (m, 2H), 2.34 (bs, 1H), 0.96 (s, 9H), 0.17 (s, 6H) ppm. ^13^C­{^1^H} NMR (101 MHz, CDCl_3_) δ 164.7, 145.1, 137.2, 134.0, 133.7, 131.9, 129.6, 128.2 (2C), 127.99, 127.97, 125.8, 125.6 (2C), 119.4, 107.7, 76.0, 73.7, 69.5, 69.2, 68.0, 60.3, 26.0 (3C), 18.4, −4.4, −4.4 ppm. IR (ATR): ν = 3479 (OH), 1716 (C=O, ester), 1631, 976 (C=C), 1099 (C–O, alcohol) cm^–1^. HRMS (ESI+) *m*/*z*: calcd. for C_28_H_33_BrNaO_7_Si [M + Na]^+^: 611.1071, found: 611.1057.

#### (1R,3R,5S,6R,7S,8R,9S)-8-((*tert*-Butyldimethylsilyl)­oxy)-9-hydroxy-3-phenyl-2,4,10-trioxaadamantan-6-yl (E)-3-(2-bromophenyl)­acrylate (**3r**)

The title compound was synthesized according to the general procedure C (reaction time: 18 h), using **1c** (38.1 mg, 0.1 mmol) and (2*Z*)-2-bromo-3-(2-bromophenyl)-2-propenal (43.5 mg, 0.15 mmol). The crude product was purified by column chromatography (hexane/EtOAc – 10:1 to 6:1), affording **3r** (28.0 mg, 47%) as a white amorphous solid.

*Er* = 87.0:13.0 (*ee* = 74%), the enantiomeric excess of product **3r** was determined by HPLC using a Chiralpak IA column (*n*-heptane/*i*-PrOH – 95:5, flow rate = 1.0 mL/min, λ = 271 nm, *t* = 25 °C): *t*_R_ = 10.6 min (minor), *t*_R_ = 14.3 min (major). [α]_D_^20^ = −23.0 (*c* = 0.4, CHCl_3_) ^1^H NMR (400 MHz, CDCl_3_) δ 7.71–7.62 (m, 4H), 7.55 (ddd, *J* = 8.0, 1.9, 1.0 Hz, 1H), 7.45 (dd, *J* = 7.9, 1.4 Hz, 1H), 7.40–7.34 (m, 3H), 7.32–7.26 (m, 1H), 6.41 (d, *J* = 15.9 Hz, 1H), 5.78–5.73 (m, 1H), 4.73–4.68 (m, 1H), 4.60–4.56 (m, 1H), 4.44–4.40 (m, 1H), 4.35–4.29 (m, 2H), 2.23 (s, 1H), 0.97 (s, 9H), 0.17 (d, *J* = 1.6 Hz, 6H) ppm. ^13^C­{^1^H} NMR (101 MHz, CDCl_3_) δ 164.9, 145.3, 137.1, 136.0, 133.8, 131.0, 130.7, 129.6, 128.2 (2C), 127.2, 125.6 (2C), 123.3, 117.9, 107.7, 76.0, 73.6, 69.6, 69.2, 68.0, 60.3, 26.0 (3C), 18.5, −4.4, −4.5 ppm. IR (ATR): ν = 3514 (OH), 1714 (C=O, ester), 1633, 962 (C=C), 1101 (C–O, alcohol) cm^–1^. HRMS (ESI+) *m*/*z*: calcd. for C_28_H_33_BrNaO_7_Si [M + Na]^+^: 611.1071, found: 611.1063.

#### (1R,3R,5S,6R,7S,8R,9S)-8-((*tert*-Butyldimethylsilyl)­oxy)-9-hydroxy-3-phenyl-2,4,10-trioxaadamantan-6-yl (E)-3-(furan-2-yl)­acrylate (**3s**)

The title compound was synthesized according to the general procedure C (reaction time: 18 h), using **1c** (38.1 mg, 0.1 mmol) and (2*Z*)-2-bromo-3-(furan-2-yl)-2-propenal (30.1 mg, 0.15 mmol). The crude product was purified by column chromatography (hexane/EtOAc – 8:1 to 4:1), affording **3s** (31.0 mg, 62%) as a beige amorphous solid.

*Er* = 82.5:17.5 (*ee* = 65%), the enantiomeric excess of product **3s** was determined by HPLC using a Chiralpak IA column (*n*-heptane/*i*-PrOH – 95:5, flow rate = 1.0 mL/min, λ = 303 nm, *t* = 25 °C): *t*_R_ = 13.2 min (minor), *t*_R_ = 20.5 min (major). [α]_D_^20^ = −20.3 (*c* = 0.3, CHCl_3_) ^1^H NMR (400 MHz, CDCl_3_) δ 7.71–7.65 (m, 2H), 7.52 (d, *J* = 1.8 Hz, 1H), 7.48 (d, *J* = 15.6 Hz, 1H), 7.42–7.33 (m, 3H), 6.68 (d, *J* = 3.4 Hz, 1H), 6.51 (dd, *J* = 3.4, 1.8 Hz, 1H), 6.29 (d, *J* = 15.6 Hz, 1H), 5.79–5.72 (m, 1H), 4.72–4.65 (m, 1H), 4.58–4.52 (m, 1H), 4.44–4.39 (m, 1H), 4.36–4.31 (m, 1H), 4.30–4.27 (m, 1H), 2.17 (bs, 1H), 0.96 (s, 9H), 0.16 (s, 3H), 0.15 (s, 3H) ppm. ^13^C­{^1^H} NMR (101 MHz, CDCl_3_) δ 165.3, 150.6, 145.6, 137.2, 133.1, 129.6, 128.2 (2C), 125.6 (2C), 116.4, 113.7, 112.8, 107.7, 76.0, 73.6, 69.4, 69.2, 68.2, 60.2, 26.0 (3C), 18.5, −4.4, −4.5 ppm. IR (ATR): ν = 3467 (OH), 1709 (C=O, ester), 1630, 970 (C=C), 1097 (C–O, alcohol) cm^–1^. HRMS (ESI+) *m*/*z*: calcd. for C_26_H_32_NaO_8_Si [M + Na]^+^: 523.1759, found: 523.1747.

#### (1R,3R,5S,6R,7S,8R,9S)-8-((*tert*-Butyldimethylsilyl)­oxy)-9-hydroxy-3-phenyl-2,4,10-trioxaadamantan-6-yl (E)-3-(thiophen-2-yl)­acrylate (**3t**)

The title compound was synthesized according to the general procedure C (reaction time: 18 h), using **1c** (38.1 mg, 0.1 mmol) and (2*Z*)-2-bromo-3-(thiophen-2-yl)-2-propenal (32.6 mg, 0.15 mmol). The crude product was purified by column chromatography (hexane/EtOAc – 5:1), affording **3t** (30.0 mg, 58%) as a beige amorphous solid.

*Er* = 80.0:20.0 (*ee* = 60%), the enantiomeric excess of product **3t** was determined by HPLC using a Chiralpak IA column (*n*-heptane/*i*-PrOH – 95:5, flow rate = 1.0 mL/min, λ = 309 nm, *t* = 25 °C): *t*_R_ = 20.0 min (minor), *t*_R_ = 24.2 min (major). [α]_D_^20^ = −18.4 (*c* = 0.4, CHCl_3_). ^1^H NMR (400 MHz, CDCl_3_) δ 7.85 (d, *J* = 15.6 Hz, 1H), 7.73–7.64 (m, 2H), 7.46–7.43 (m, 1H), 7.40–7.35 (m, 3H), 7.30 (d, *J* = 3.6 Hz, 1H), 7.08 (dd, *J* = 5.1, 3.6 Hz, 1H), 6.20 (d, *J* = 15.6 Hz, 1H), 5.78–5.72 (m, 1H), 4.72–4.65 (m, 1H), 4.58–4.52 (m, 1H), 4.44–4.39 (m, 1H), 4.36–4.31 (m, 1H), 4.31–4.29 (m, 1H), 2.46 (s, 1H), 0.97 (s, 9H), 0.17 (s, 3H), 0.16 (s, 3H) ppm. ^13^C­{^1^H} NMR (101 MHz, CDCl_3_) δ 165.1, 139.5, 139.1, 137.2, 132.1, 129.64, 129.60, 128.5, 128.1 (2C), 125.6 (2C), 114.8, 107.7, 76.0, 73.6, 69.4, 69.2, 68.1, 60.3, 26.0 (3C), 18.5, −4.4, −4.5 ppm. IR (ATR): ν = 3489 (OH), 1712 (C=O, ester), 1620, 970 (C=C), 1101 (C–O, alcohol) cm^–1^. HRMS (ESI+) *m*/*z*: calcd. for C_26_H_32_NaO_7_SSi [M + Na]^+^: 539.1530, found: 539.1524.

#### (1R,3R,5S,6R,7S,8R,9S)-8-((*tert*-Butyldimethylsilyl)­oxy)-9-hydroxy-3-phenyl-2,4,10-trioxaadamantan-6-yl ethyl fumarate (**3u**)

The title compound was synthesized according to the general procedure C (reaction time: 18 h), using **1c** (38.1 mg, 0.1 mmol) and ethyl (*Z*)-3-bromo-4-oxobut-2-enoate (31.1 mg, 0.15 mmol). The crude product was purified by column chromatography (hexane/EtOAc – 5:1), affording **3u** (9.0 mg, 18%) as a beige amorphous solid.

*Er* = 74.1:25.9 (*ee* = 48%), the enantiomeric excess of product **3u** was determined by HPLC using a Chiralpak IB column (*n*-heptane/*i*-PrOH – 80:20, flow rate = 1.0 mL/min, λ = 208 nm, *t* = 25 °C): *t*_R_ = 5.2 min (minor), *t*_R_ = 6.3 min (major). [α]_D_^20^ = −11.1 (*c* = 0.5, CHCl_3_). ^1^H NMR (400 MHz, CDCl_3_) δ 7.69–7.62 (m, 2H), 7.40–7.33 (m, 3H), 6.89 (d, *J* = 15.8 Hz, 1H), 6.84 (d, *J* = 15.7 Hz, 1H), 5.71–5.66 (m, 1H), 4.71–4.64 (m, 1H), 4.58–4.53 (m, 1H), 4.39–4.34 (m, 1H), 4.33–4.23 (m, 4H), 2.31 (d, *J* = 6.2 Hz, 1H), 1.34 (t, *J* = 7.1 Hz, 3H), 0.96 (s, 9H), 0.153 (s, 3H), 0.148 (s, 3H) ppm. ^13^C­{^1^H} NMR (101 MHz, CDCl_3_) δ 164.8, 163.7, 137.1, 135.4, 132.3, 129.7, 128.2 (2C), 125.5 (2C), 107.7, 75.9, 73.5, 70.0, 69.1, 67.7, 61.8, 60.2, 26.0 (3C), 18.4, 14.2, −4.4, −4.5 ppm. IR (ATR): ν = 3473 (OH), 1722 (C=O, ester), 1101 (C–O, alcohol), 962 (C=C) cm^–1^. HRMS (ESI+) *m*/*z*: calcd. for C_25_H_35_O_9_Si [M + H]^+^: 507.2045, found: 507.2055.

#### (1R,3R,5S,6R,7S,8S,9R)-8-Acetoxy-9-((*tert*-butyldimethylsilyl)­oxy)-3-phenyl-2,4,10-trioxaadamantan-6-yl (E)-3-(4-methoxyphenyl)­acrylate (**4i**)

The title compound was synthesized according to the general procedure D (reaction time of first step: 18 h), using **1c** (38.1 mg, 0.1 mmol) and (2*Z*)-2-bromo-3-(4-methoxyphenyl)-2-propenal (36.2 mg, 0.15 mmol). The crude product was purified after the second step was purified by column chromatography (hexane/EtOAc – 10:1 to 7:1), affording **4i** (18.0 mg, 31%) as a white amorphous solid.

*Er* = 73.5:26.5 (*ee* = 47%), the enantiomeric excess of product **4i** was determined by HPLC using a Chiralpak IG column (*n*-heptane/*i*-PrOH – 80:20, flow rate = 1.0 mL/min, λ = 310 nm, *t* = 25 °C): *t*_R_ = 14.3 min (minor), *t*_R_ = 16.5 min (major). [α]_D_^20^ = +6.3 (*c* = 0.4, CHCl_3_). ^1^H NMR (400 MHz, CDCl_3_) δ 7.72–7.65 (m, 3H), 7.50–7.45 (m, 2H), 7.40–7.35 (m, 3H), 6.97–6.91 (m, 2H), 6.26 (d, *J* = 15.9 Hz, 1H), 5.71–5.66 (m, 1H), 5.66–5.62 (m, 1H), 4.81–4.77 (m, 1H), 4.44–4.39 (m, 1H), 4.39–4.35 (m, 1H), 4.31–4.27 (m, 1H), 3.86 (s, 3H), 2.03 (s, 3H), 0.98 (s, 9H), 0.17 (s, 6H) ppm. ^13^C­{^1^H} NMR (101 MHz, CDCl_3_) δ 169.4, 165.6, 162.1, 146.2, 137.0, 130.0 (2C), 129.7, 128.2 (2C), 126.6, 125.6 (2C), 114.7 (2C), 114.1, 108.0, 73.6, 73.4, 68.6, 68.5, 67.2, 60.9, 55.6, 26.0 (3C), 20.9, 18.5, −4.5 (2C). IR (ATR): ν = 1751, 1705 (C=O, ester), 978 (C=C) cm^–1^. HRMS (ESI+) *m*/*z*: calcd. for C_31_H_39_O_9_Si [M + H]^+^: 583.2358, found: 583.2362.

#### (1R,3R,5S,6R,7S,8S,9R)-8-Acetoxy-9-((*tert*-butyldimethylsilyl)­oxy)-3-phenyl-2,4,10-trioxaadamantan-6-yl (E)-3-(4-nitrophenyl)­acrylate (**4k**)

The title compound was synthesized according to the general procedure D (reaction time of first step: 42 h), using **1c** (38.1 mg, 0.1 mmol) and (2*Z*)-2-bromo-3-(4-nitrophenyl)-2-propenal (38.4 mg, 0.15 mmol). The crude product was purified after the second step by column chromatography (hexane/EtOAc – 10:1 to 5:1), affording **4k** (24.0 mg, 40%) as a white amorphous solid.

*Er* = 83.4:16.6 (*ee* = 67%), the enantiomeric excess of product **4k** was determined by HPLC using a Chiralpak IB column (*n*-heptane/*i*-PrOH – 80:20, flow rate = 1.0 mL/min, λ = 294 nm, *t* = 25 °C): *t*_R_ = 18.9 min (minor), *t*_R_ = 20.9 min (major). [α]_D_^20^ = +6.0 (*c* = 0.5, CHCl_3_). ^1^H NMR (400 MHz, CDCl_3_) δ 8.30–8.25 (m, 2H), 7.78 (d, *J* = 16.0 Hz, 1H), 7.73–7.62 (m, 4H), 7.43–7.32 (m, 3H), 6.52 (d, *J* = 16.0 Hz, 1H), 5.75–5.69 (m, 1H), 5.67–5.62 (m, 1H), 4.82–4.77 (m, 1H), 4.46–4.41 (m, 1H), 4.41–4.36 (m, 1H), 4.28–4.24 (m, 1H), 2.04 (s, 3H), 0.97 (s, 9H), 0.17 (s, 6H) ppm. ^13^C­{^1^H} NMR (101 MHz, CDCl_3_) δ 169.1, 164.5, 149.0, 143.7, 139.8, 136.8, 129.8, 128.9 (2C), 128.2 (2C), 125.5 (2C), 124.5 (2C), 120.8, 108.0, 73.34, 73.27, 69.2, 68.6, 67.1, 60.7, 26.0 (3C), 20.9, 18.5, −4.44, −4.45 ppm. IR (ATR): ν = 1747, 1726 (C=O, ester), 1520 (N–O), 993, 982 (C=C) cm^–1^. HRMS (ESI+) *m*/*z*: calcd. for C_30_H_36_NO_10_Si [M + H]^+^: 598.2103, found: 598.2091.

#### (1R,3R,5S,6R,7S,8R,9S)-8-((*tert*-Butyldimethylsilyl)­oxy)-9-hydroxy-3-phenyl-2,4,10-trioxaadamantan-6-yl 2-(2-(*tert*-butyl)-6-methylphenoxy)-3-formylbenzoate (**5b**)

The title compound was synthesized according to the general procedure A. A small vial was loaded with **1c** (38.1 mg, 0.1 mmol), 2-(2-(*tert*-butyl)-6-methylphenoxy)­isophthalaldehyde (44.5 mg, 0.15 mmol) and 3,3′,5,5′-tetra-*tert*-butyldiphenoquinone (61.3 mg, 0.15 mmol), DABCO (16.8 mg, 0.15 mmol), *pre*-**C3** (0.5 mg, 1 mol %) and chlorobenzene (1 mL). The reaction mixture was stirred for 3 days at room temperature. The crude product was purified by column chromatography (hexane/EtOAc – 10:1 to 5:1), affording **5b** (33.0 mg, 49%) as a white amorphous solid.

*Note*: Diastereomeric ratio was determined by ^1^H NMR of crude reaction mixture.

*Dr* = 1:1.4, *er* = 74:26/83:17 (*ee* = 48/66%), the enantiomeric excess of product **5b** was determined by HPLC using a Chiralpak IB column (*n*-heptane/*i*-PrOH – 97:3, flow rate = 1.0 mL/min, λ = 206 nm, *t* = 25 °C): *t*_R_ = 16.1 min (major), *t*_R_ = 17.6 min (minor), *t*_R_ = 20.2 min (minor’), *t*_R_ = 24.9 min (major’).

The title compound was also prepared according to the procedure reported in the literature.^8^ A small vial was loaded with **1c** (190 mg, 0.5 mmol), 2-(2-(*tert*-butyl)-6-methylphenoxy)­isophthalaldehyde (30.0 mg, 0.1 mmol) and 3,3′,5,5′-tetra-*tert*-butyldiphenoquinone (49.0 mg, 0.12 mmol), DBU (22.0 μL, 0.15 mmol), *pre*-**C4** (0.5 mg, 1 mol %) and 1,4-dioxane (1 mL). The reaction mixture was stirred for 3 days at room temperature. The crude product was purified by column chromatography (hexane/EtOAc – 10:1 to 5:1), affording **5b** (43.0 mg, 64%).

Note: Diastereomeric ratio was determined by ^1^H NMR of crude reaction mixture.

*Dr* = 1.4:1, *er* = 20:80/87:13 (*ee* = 60/74%), the enantiomeric excess of product **5b** was determined by HPLC using a Chiralpak IB column (*n*-heptane/*i*-PrOH – 97:3, flow rate = 1.0 mL/min, λ = 208 nm, *t* = 25 °C): *t*_R_ = 16.1 min (minor), *t*_R_ = 17.6 min (major), *t*_R_ = 20.2 min (minor’), *t*_R_ = 24.9 min (major’).

^1^H NMR (400 MHz, CDCl_3_) δ 9.83 (s, 1H), 9.53 (s, 1H), 8.00–7.88 (m, ND), 7.69–7.62 (m, ND), 7.40–7.29 (m, ND), 7.23–7.07 (m, ND), 7.06–6.97 (m, ND), 5.74–5.68 (m, 1H), 5.44–5.39 (m, 1H’), 4.73–4.59 (m, 1H+2H’), 4.58–4.53 (m, 1H), 4.47–4.42 (m, 1H), 4.35–4.24 (m, 2H+3H’), 2.34 (bs, 1H+1H’), 1.93 (s, 3H), 1.91 (s, 3H’), 1.46 (s, 9H’), 1.44 (s, 9H), 0.94 (s, 9H), 0.93 (s, 9H’), 0.12 (s, 6H), 0.08 (s, 3H’), 0.04 (s, 3H’) ppm. ^13^C NMR (101 MHz, CDCl_3_) δ 188.2 (C’), 187.8 (C), 163.92 (C), 163.86 (C’), 157.9 (C), 157.7 (C’), 155.1 (C), 154.7 (C’), 141.5 (C’), 141.1 (C), 137.12 (C’), 137.07 (C), 136.9 (C), 136.8 (C’), 133.8 (C), 133.1 (C’), 131.2 (C), 130.7 (C’), 129.7 (C), 129.6 (C’), 128.2 (2C+2C’), 127.7 (C), 127.5 (C’), 127.3 (C), 127.0 (C’), 126.4 (C+C’), 125.9 (C), 125.8 (C’), 125.5 (2C+2C’), 122.32 (C), 122.30 (C’), 122.1 (C+C’?), 107.7 (C+C’), 76.1, 75.90 (C’), 75.89 (C), 73.6 (C’), 73.5 (C), 70.3 (C), 70.1 (C’), 69.1 (C), 68.9 (C’), 67.82 (C’), 67.81 (C), 60.3 (C+C’), 35.5 (C’), 35.4 (C), 30.6 (3C’), 30.5 (3C), 25.98 (3C), 25.95 (3C’), 18.44 (C), 18.37 (C’), 17.74 (C’), 17.66 (C), −4.37 (C), −4.48 (C), −4.50 (C’), −4.6 (C’) ppm. IR (ATR): ν = 3479 (OH), 1739 (C=O, ester), 1684 (C=O, aldehyde), 1099 (C–O, alcohol) cm^–1^. HRMS (ESI+) *m*/*z*: calcd. for C_38_H_47_O_9_Si [M + H]^+^: 675.2984, found: 675.3002.

#### (1R,3R,5S,6R,7S,8R,9S)-8-((*tert*-Butyldimethylsilyl)­oxy)-9-hydroxy-3-phenyl-2,4,10-trioxaadamantan-6-yl cinnamate (**3a**)

The round-bottom flask (100 mL) was charged with inositol **1c** (1.00 g, 2.63 mmol, 1.0 equiv), (2*Z*)-2-bromo-3-phenyl-2-propenal **2a** (832 mg, 3.94 mmol, 1.5 equiv), *pre*-**C4** (12 mg, 26.3 μmol, 1 mol %), DABCO (442 mg, 3.94 mmol, 1.5 equiv) and DCM (26 mL). The reaction was stirred at room temperature (∼23 °C) for 18 h. After full conversion of inositol the solvent was evaporated under reduced pressure. The crude product was purified by column chromatography (hexane/EtOAc – 10:1 to 3:1), affording **3a** (669 mg, 50%) as a white amorphous solid. *Er* = 83.9:16.1 (68% *ee*).

All analytical data are consistent with those obtained for the compound prepared on a 0.1 mmol scale.

#### (1R,3S,5S,6R,7R,8S,9S)-8-((*tert*-Butoxycarbonyl)­oxy)-9-((*tert*-butyldimethylsilyl)­oxy)-3-phenyl-2,4,10-trioxaadamantan-6-yl cinnamate (**6**)

The product of desymmetrization **3a** (51 mg, 0.1 mmol) was added to the vial (4 mL). Boc_2_O (26 mg, 0.12 mmol), DMAP (0.6 mg, 5 mol %) and anhydrous THF (1 mL) were added. The reaction was heated to 50 °C and stirred until full conversion (monitored by TLC, ∼3 h). The crude product was purified by column chromatography (hexane/EtOAc – 5:1) affording **6** (52 mg, 85%) as a colorless oil.

*Er* = 84.3:15.7 (*ee* = 69%), the enantiomeric excess of product **6** was determined by HPLC using a Chiralpak ODH column (*n*-heptane/*i*-PrOH – 99:1, flow rate = 1.0 mL/min, λ = 275 nm, *t* = 25 °C): *t*_R_ = 17.4 min (major), *t*_R_ = 31.3 min (minor). [α]_D_^20^ = +7.9 (*c* = 0.4, CHCl_3_). ^1^H NMR (400 MHz, CDCl_3_) δ 7.75 (d, *J* = 16.1 Hz, 1H), 7.72–7.67 (m, 2H), 7.59–7.52 (m, 2H), 7.47–7.33 (m, 6H), 6.44 (d, *J* = 16.0 Hz, 1H), 5.77–5.71 (m, 1H), 5.45–5.39 (m, 1H), 4.87–4.81 (m, 1H), 4.47–4.38 (m, 2H), 4.35–4.30 (m, 1H), 1.37 (s, 9H), 0.98 (s, 9H), 0.18 (s, 6H) ppm. ^13^C­{^1^H} NMR (101 MHz, CDCl_3_) δ 165.3, 152.1, 146.4, 137.0, 134.2, 130.9, 129.7, 129.1 (2C), 128.3 (2C), 128.1 (2C), 125.6 (2C), 117.1, 107.9, 83.3, 73.4 (2C), 70.6, 68.1, 67.1, 60.8, 27.7 (3C), 26.0 (3C), 18.5, −4.4, −4.5 ppm. IR (ATR): ν = 1745, 1716 (C=O, ester), 1635, 976 (C=C) cm^–1^. HRMS (ESI+) *m*/*z*: calcd. for C_33_H_43_O_9_Si [M + H]^+^: 611.2671, found: 611.2674.

#### (1R,3R,5S,6R,7S,8S,9R)-8-acetoxy-9-((*tert*-Butyldimethylsilyl)­oxy)-3-phenyl-2,4,10-trioxaadamantan-6-yl cinnamate (**4a**)

The product of desymmetrization **3a** (51 mg, 0.1 mmol) was added to the vial (4 mL). Ac_2_O (11 μL, 0.12 mmol), DMAP (1.2 mg, 10 mol %), Et_3_N (17 μL, 0.12 mmol) and dry THF (1 mL) were added. The reaction was stirred at room temperature until full conversion (monitored by TLC, ∼30 min). The crude product was purified by column chromatography (hexane/EtOAc – 5:1) affording **4a** (50 mg, 90%) as a colorless oil.

*Er* = 84.0:16.0 (*ee* = 68%), the enantiomeric excess of product **4a** was determined by HPLC using a Chiralpak IG column (*n*-heptane/*i*-PrOH – 95:5, flow rate = 1.0 mL/min, λ = 276 nm, *t* = 25 °C): *t*_R_ = 19.8 min (major), *t*_R_ = 22.1 min (minor). [α]_D_^20^ = +9.8 (*c* = 0.4, CHCl_3_). ^1^H NMR (400 MHz, CDCl_3_) δ 7.75 (d, *J* = 16.0 Hz, 1H), 7.72–7.67 (m, 2H), 7.53 (dt, *J* = 6.7, 2.3 Hz, 2H), 7.44 (dd, *J* = 5.1, 1.9 Hz, 3H), 7.41–7.36 (m, 3H), 6.41 (d, *J* = 16.0 Hz, 1H), 5.73–5.68 (m, 1H), 5.68–5.63 (m, 1H), 4.84–4.78 (m, 1H), 4.47–4.41 (m, 1H), 4.41–4.36 (m, 1H), 4.32–4.28 (m, 1H)­f, 2.04 (s, 3H), 0.98 (s, 9H), 0.18 (s, 6H) ppm. ^13^C­{^1^H} NMR (101 MHz, CDCl_3_) δ 169.3, 165.3, 146.5, 136.9, 133.9, 131.1, 129.7, 129.3 (2C), 128.2 (2C), 128.1 (2C), 125.6 (2C), 116.7, 108.0, 73.5, 73.4, 68.62, 68.57, 67.2, 60.8, 26.0 (3C), 20.9, 18.5, −4.5 (2C) ppm. IR (ATR): ν = 1749, 1712 (C=O, ester), 978 (C=C) cm^–1^. HRMS (ESI+) *m*/*z*: calcd. for C_30_H_37_O_8_Si [M + H]^+^: 553.2252, found: 553.2261.

#### (1S,3R,5R,6S,7S,8S,9S)-8-((*tert*-Butyldimethylsilyl)­oxy)-9-(((2S,3aS,6R,7aS)-3a-methyl-6-(prop-1-en-2-yl)-2-sulfidohexahydrobenzo­[d]­[1,3,2]­oxathiaphosphol-2-yl)­oxy)-3-phenyl-2,4,10-trioxaadamantan-6-yl cinnamate (**7**)

The vial (4 mL) was charged with the desymmetrization product **3a** (51 mg, 0.1 mmol), PSI-reagent (67 mg, 0.15 mmol) and DCM (1 mL) under inert atmosphere. Then DBU (23 μL, 0.15 mmol) was slowly added. The reaction mixture was stirred at room temperature until full conversion (monitored by TLC, ∼30 min). The crude product was purified by column chromatography (hexane/EtOAc – 20:1 to 10:1) affording **7** (69 mg, 91%) as a colorless oil.

*Dr* = 5.6:1.0, the diastereomeric ratio product **7** was determined by NMR spectroscopy.

^1^H NMR (400 MHz, CDCl_3_, only major diastereomer) δ 7.75–7.66 (m, 3H), 7.55–7.50 (m, 2H), 7.44–7.35 (m, 6H), 6.40 (d, *J* = 16.1 Hz, 1H), 5.76–5.71 (m, 1H), 5.54–5.47 (m, 1H), 4.97–4.94 (m, 1H), 4.70–4.65 (m, 2H), 4.65–4.61 (m, 1H), 4.42–4.39 (m, 1H), 4.35–4.31 (m, 1H), 2.51–2.44 (m, 1H), 2.27–2.17 (m, 1H), 1.75–1.53 (m, 12H), 0.97 (s, 9H), 0.18 (s, 6H) ppm. ^13^C­{^1^H} NMR (101 MHz, CDCl_3_, only major diastereomer) δ 165.2, 146.2, 144.6, 136.8, 134.1, 130.9, 129.7, 129.1 (2C), 128.5 (2C), 128.1 (2C), 125.6 (2C), 117.3, 112.0, 86.3, 73.8 (d, *J* = 2.7 Hz), 73.6, 71.9 (d, *J* = 7.8 Hz), 68.5 (d, *J* = 5.5 Hz), 68.2, 66.3, 60.1, 38.7, 33.1 (d, *J* = 9.1 Hz), 27.8 (d, *J* = 15.4 Hz), 26.1 (3C), 23.2, 22.6, 21.9, 18.6, −4.4, −4.5 ppm, one qC was not assigned due to possible overlap with other signals. ^31^P NMR (162 MHz, CDCl_3_, only major diastereomer) δ 100.9 ppm. IR (ATR): ν = 1716 (C=O, ester), 1635, 980 (C=C) cm^–1^. HRMS (ESI+) *m*/*z*: calcd. for C_38_H_50_O_8_PS_2_Si [M + H]^+^: 757.2448, found: 757.2457.

#### (1S,3R,5S,6S,7R,8R,9R)-8-((*tert*-Butyldimethylsilyl)­oxy)-3-phenyl-9-(((E)-prop-1-en-1-yl)­oxy)-2,4,10-trioxaadamantan-6-ol (**11**)

To a solution of the product of desymmetrization **3a** (102 mg, 200 μmol) and pyridinium *p*-toluenesulfonate (22 mg, 88 μmol, 44 mol %) in DCM (2 mL) was added 3,4-dihydropyran (80 mg, 880 μmol, 4.4 equiv). The reaction was stirred at room temperature for 16 h. The solvent was evaporated, crude product was dissolved in EtOAc and washed with water (1 × 30 mL), HCl (1m, 1 × 30 mL), sat. solution of NaHCO_3_ (1 × 30 mL) and brine (1 × 30 mL). The organic phase was dried over MgSO_4_ and the product was purified by a short column chromatography (hexane/EtOAc – 10:1 to 8:1) affording the first intermediate **8** (90 mg, 76%). This intermediate **8** (90 mg, 151 μmol) was then dissolved in MeOH (1.5 mL) and solution of NaOMe (10.2 mg, 189 μmol, 1.25 equiv) in MeOH (0.5 mL) was slowly added. The reaction mixture was stirred at room temperature for 1 h (full conversion monitored by TLC). Simple column chromatography (hexane/EtOAc – 10:1 to 8:1) yielded the intermediate **9** (55 mg, 78%). After that the intermediate **9** (55 mg, 118 μmol) was mixed with imidazole (0.4 mg, 6 μmol, 5 mol %) in dry DMF (1.1 mL) in a small vial (4 mL). NaH (9.5 mg, 237 μmol, 2.0 equiv) was added at 0 °C (water/ice cooling bath) and the mixture was stirred for 30 min at room temperature. Then allyl bromide (15 μL, 178 μmol, 1.5 equiv) was added dropwise at 0 °C (water/ice cooling bath). The reaction mixture was stirred at room temperature until full conversion (monitored by TLC, 1 h). The reaction was then quenched with MeOH (2 mL) and diluted with EtOAc (30 mL) and brine (30 mL). The water phase was extracted with EtOAc (3 × 30 mL). The combined organic phases were washed with brine (2 × 30 mL). After drying over MgSO_4_ the crude product was purified by short column chromatography (hexane/EtOAc – 10:1 to 8:1) affording the intermediate **10** (39 mg, 65%). The intermediate **10** (39 mg, 77 μmol) was added to a small vial and mixed under argon atmosphere with MgBr_2_·OEt_2_ (94 mg, 363 μmol, 4.7 equiv) and Et_2_O (1.5 mL). The reaction mixture was stirred at room temperature for 2 h. The reaction was then quenched by saturated NH_4_Cl solution at 0 °C. The water phase was extracted with EtOAc and the combined organic phases were washed with water and brine. After drying over MgSO_4_ the crude product was purified by a column chromatography (hexane/EtOAc – 10:1 to 7:1) affording the final product **11** (24 mg, 72%) as a colorless oil.

*Er* = 83.7:16.3 (*ee* = 67%), the enantiomeric excess of product **11** was determined by HPLC using a Chiralpak IH column (*n*-heptane/*i*-PrOH – 98:2, flow rate = 1.0 mL/min, λ = 207 nm, *t* = 25 °C): *t*_R_ = 6.4 min (major), *t*_R_ = 7.2 min (minor). [α]_D_^20^ = +6.1 (*c* = 1.2, CHCl_3_). ^1^H NMR (400 MHz, CDCl_3_) δ 7.69–7.60 (m, 2H), 7.39–7.31 (m, 3H), 5.97–5.85 (m, 1H), 5.39–5.27 (m, 2H), 4.59–4.52 (m, 1H), 4.48–4.41 (m, 2H), 4.39–4.35 (m, 1H), 4.33–4.29 (m, 1H), 4.24–4.21 (m, 1H), 4.20–4.16 (m, 2H), 3.67 (d, *J* = 10.2 Hz, 1H), 0.97 (s, 9H), 0.16 (s, 6H) ppm. ^13^C­{^1^H} NMR (101 MHz, CDCl_3_) δ 137.4, 133.0, 129.5, 128.1 (2C), 125.5 (2C), 119.5, 107.4, 76.5, 75.0, 74.1, 72.1, 68.5, 68.4, 60.1, 26.0 (3C), 18.4, −4.4, −4.5 ppm. IR (ATR): ν = 3479 (OH), 1716 (C=O, ester), 978 (C=C), 1101 (C–O, alcohol) cm^–1^. HRMS (ESI+) *m*/*z*: calcd. for C_22_H_33_O_6_Si [M + H]^+^: 421.2041, found: 421.2037.

## Supplementary Material





## Data Availability

The data underlying this study are available in the published article and its .

## Notes

Reaction conditions optimization, copies of ^1^H NMR, ^13^C NMR, ^19^F NMR, ^31^P NMR and copies of chiral HPLC (PDF)
FAIR data, including the primary NMR FID files, for compounds **1a**-**1g**, **3a**-**u**, **4a**, **4i**, **4k**, **5b**, **6**, **7**, **11**. (ZIP)
