# Supplementary material for: Enantioselective Organocatalytic Desymmetric Acylation as an Access to Orthogonally Protected myo-Inositols
Source: J Org Chem. 2025 Dec 23;91(1):656–68. doi: 10.1021/acs.joc.5c02735 (PMC12797290; doi:10.1021/acs.joc.5c02735)
Supplement: Supplementary file 1 [file jo5c02735_si_001.pdf]

Enantioselective Organocatalytic Desymmetric Acylation as an Access to Orthogonally Protected *myo*-Inositols

Ondřej Hladík,[1] Vojtěch Dočekal,[1] Ivana Císařová[2] & Jan Veselý[1*]*

[1] Department of Organic Chemistry, Faculty of Science, Charles University, Hlavova 2030/8, 128 43 Prague 2, Czech Republic, e-mail: vojtech.docekal@natur.cuni.cz, jan.vesely@natur.cuni.cz
[2] Department of Inorganic Chemistry, Faculty of Science, Charles University, Hlavova 2030/8, 128 43 Prague 2, Czech Republic

Supporting Information

Table of content

Desymmetrization reaction **S3**

Complete reaction optimization survey *S3*

Crystallographic data **S10**

NMR spectra **S12**

Chiral HPLC **S48**

References **S75**

Desymmetrization reaction

Complete reaction optimization survey

Figure S1. Screened NHC precursors.

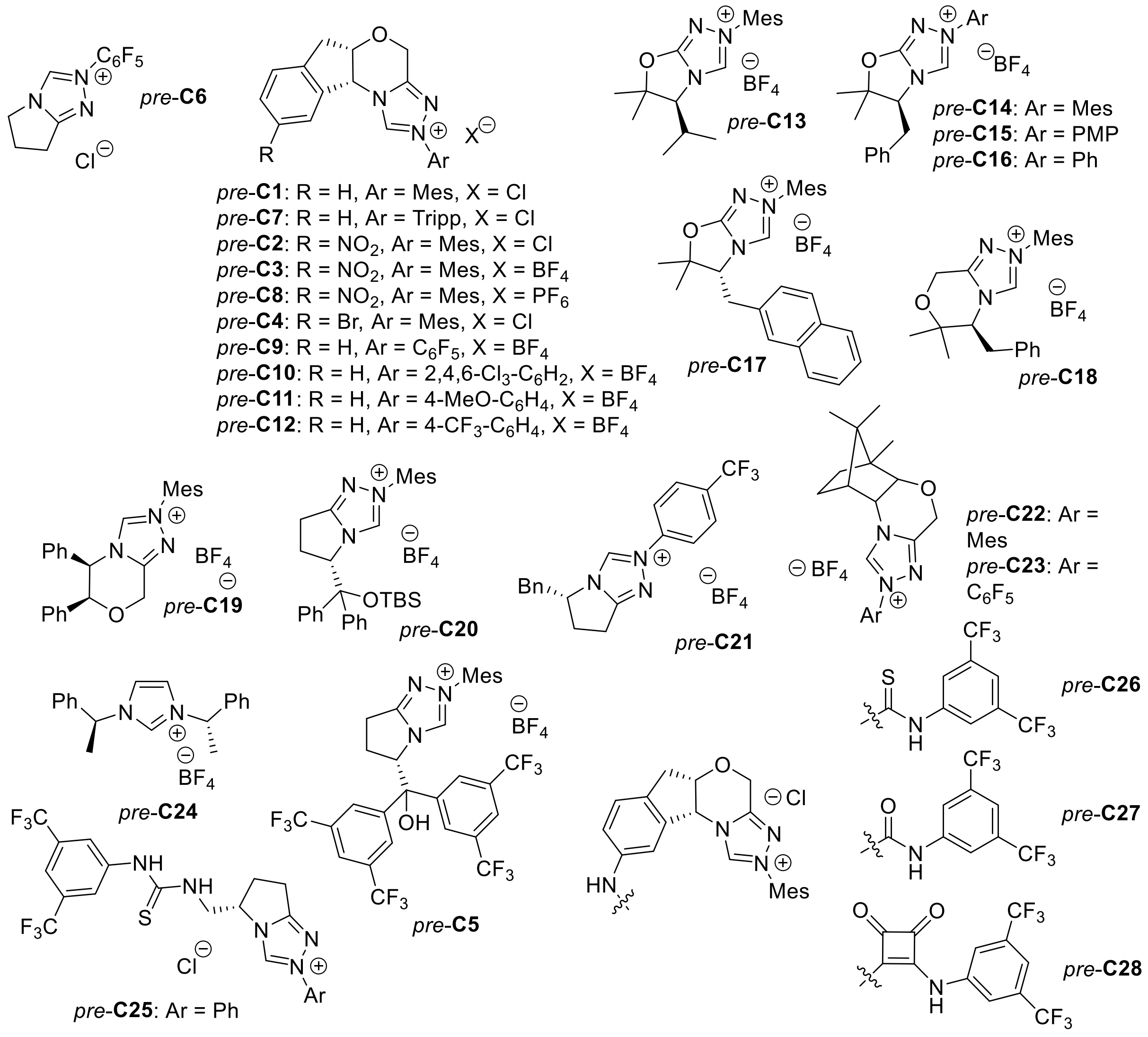

Table S1. Precursor screening.

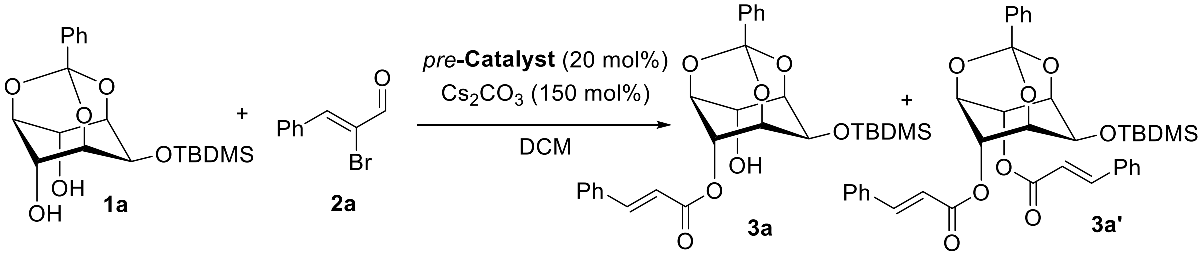

Entry	*pre*-NHC	Time (h)	Conversion[a]	Yield (mono, %)[b]	*E*/*Z*[d]	Yield (di, %)[b]	*er* (*E*/*Z*, %)[c]
1	*pre*-**C6**	1	full	69	5.8:1	-	50:50
2	*pre*-**C1**	18	full	65	5.6:1	8	60:40 / 56.5:43.5
3	*pre*-**C7**	18	full	84	7:1	10	57:43 / 62:38
4	*pre*-**C2**	2	full	61	11:1	8	68:32
2	*ent-pre*-**C2**	18	full	74	8.8:1	-	25:75 / 56:44
6	*pre*-**C3**	18	full	78	12:1	16	76:24
7	*ent-pre*-**C3**	18	full	76	12:1	5	30:70
8	*pre*-**C8**	18	full	78	11:1	-	74:26
9	*ent-pre*-**C8**	18	full	76	11:1	5	32:68
10	*pre*-**C4**	18	full	59	4.8:1	5	69:31 / 61:39
11	*pre*-**C9**	18	full	90	11:1	6	55:45
12	*pre*-**C10**	18	full	82	>20:1	-	65:35
13	*pre*-**C11**	18	full	45	4.3:1	16	52:48 / 64:36
14	*pre*-**C12**	18	full	45	8.4:1	9	58:42 / 58:42
15	*pre*-**C13**	18	full	63	10:1	-	45.5:55.5
16	*ent-pre*-**C13**	18	full	90	11:1	-	55:5:45:5
17	*pre*-**C14**	18	full	90	7.4:1	-	45.5:55.5 / 58.5:41.5
18	*pre*-**C15**	18	full	71	2.8:1	6	49.5:50.5 / 47:53
19	*pre*-**C16**	18	full	88	2.4:1	-	49.5:50.5 / 50:50
20	*pre*-**C17**	18	not full	71	7.5:1	7	50:50 / 46:54
21	*pre*-**C18**	18	full	94	5.4:1	-	45.5:55.5 / 54:46
22	*pre*-**C19**	18	full	84	14:1	3	50.5:49.5
23	*pre*-**C20**	18	full	31	20:1	20	50:50
24	*pre*-**C21**	18	full	86	3.7:1	3	47.5:52.5 / 56.5:43.5
25	*pre*-**C22**	18	full	69	4.6:1	9	50.5:49.5 / 61.5:38.5
26	*pre*-**C23**	18	full	67	20:1	11	50.5:49.5
27	*pre*-**C24**	18	not full	22	11:1	7	50.5:49.5
28	*pre*-**C5**	18	full	96	2.8:1	5	69.5:30.5 / 66.5:33.5
29	*pre*-**C25**	18	full	78	5.5:1	12	50:50 / 53.5:46.5
30	*pre*-**C26**	18	full	94	>20:1	-	52.5:47.5
31	*ent-pre*-**C26**	18	full	82	>20:1	-	52:48
32	*pre*-**C27**	18	full	69	>20:1	14	50:50
33	*pre*-**C28**	18	full	67	>20:1	-	47.5:52.5

Reactions conditions: **1a** (0.1 mmol), **2a** (0.12 mmol), Cs_2CO_3 (0.15 mmol), pre-**Catalyst** (20 mol%) in DCM (1.0 mL) at room temperature. [a] Determined by crude TLC analysis. [b] Isolated after column chromatography. [c] Determined by chiral HPLC. [d] Determined by ^{1}H NMR. er = enantiomeric ratio

Table S2. Base screening

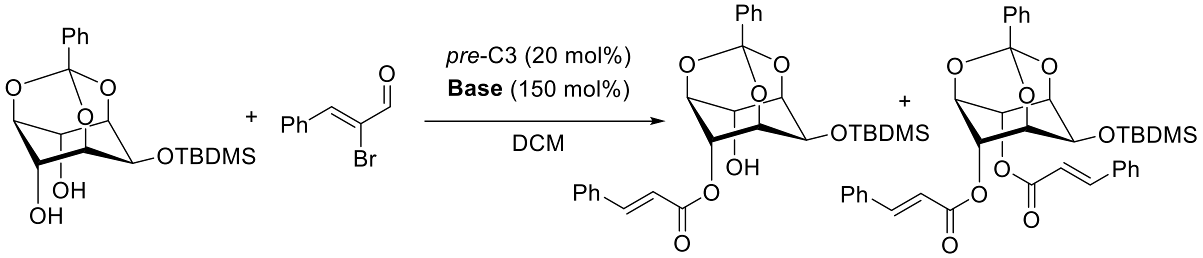

Entry	Base	Time (h)	Conversion[a]	Yield (mono, %)[b]	*E*/*Z*[d]	Yield (di, %)[b]	*er* (*E*/*Z*, %)[c]
1	Cs_2CO_3	18	full	90	13:1	-	78.5:21.5
2	Rb_2CO_3	18	full	86	11:1	-	78:22
3	K_2CO_3	18	full	31	>20:1	22	74.5:25.5
4	Na_2CO_3	18	full	98	>20:1	-	79:21
5	$NaHCO_3$	116	not full	63	>20:1	-	78:22
6	KO*t*Bu	18	full	59	2:1	-	68:32 / 63.5:36.5
7	AcONa	18	full	78	>20:1	-	77:23
8	PhCOONa	18	full	72	>20:1	-	76:24
9	TEA	18	full	88	>20:1	-	76:24
10	DIPEA	18	full	88	5.2:1	-	75:25 / 66.5:33.5
11	DABCO	18	full	53	>20:1	-	83:17
12	DBU	18	full	78	4.3:1	-	70:30 / 68.5:31.5
13	pyridine	18	not full	traces	-	-	-
14	2,6-lutidine	18	not full	16	>20:1	-	82:18
15	proton sponge	18	full	86	7.2:1	-	79:21 / 69:31
16	Me_4-guanidine	18	full	96	4.6:1	-	71:29 / 68:32
17	TBD	18	full	35	3.9:1	22	51:49 / 53:47
18	DBN	18	full	65	15:1	14	65:35

Reactions conditions: inositol (0.1 mmol), bromoenal (0.12 mmol), **Base** (0.15 mmol), pre-C3 (20 mol%) in DCM (1.0 mL) at room temperature. [a] Determined by crude TLC analysis. [b] Isolated after column chromatography. [c] Determined by chiral HPLC. [d] Determined by ^{1}H NMR. er = enantiomeric ratio

Table S3. Solvent screening.

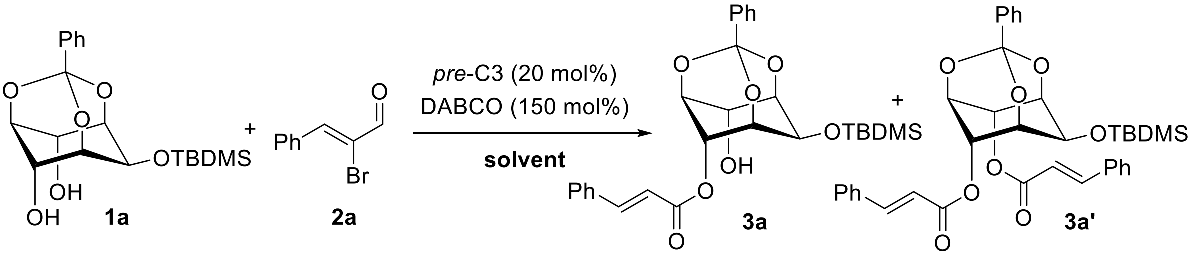

Entry	Solvent	Time (h)	Conversion[a]	Yield (mono, %)[b]	*E/Z*[d]	Yield (di, %)[b]	*er* (*E/Z*, %)[c]
1	DCM	18	full	53	>20:1	---	83:17
2	$CHCl_3$	18	full	47	>20:1	---	88:12
3	1,2-DCE	18	full	49	>20:1	---	81.5:18.5
4	CCl_4	18	full	57	>20:1	traces	91:9 (>99:1)[e]
5	CCl_4[f]	18	full	45	>20:1	traces	87:13
6	benzene	18	full	49	>20:1	---	85:15
7	toluene	18	full	51	>20:1	---	87:13
8	MeCN	1	full	80	>20:1	---	67:33
9	EtOAc	3	full	84	>20:1	---	77.5:22.5
10	C_6F_6	18	full	45	>20:1	---	89.5:10.5
11	C_6F_{14}	18	full	65	>20:1	---	79:21
12	MTBE	2	full	53	>20:1	---	89:11
13	THF	2	full	57	>20:1	---	82.5:17.5
14	PhCl	1	full	61	>20:1	---	85:15
15	1,2-DCB	1	full	45	>20:1	---	85:15
16	PhBr	1	full	45	>20:1	---	86.5:13.5
17	$PhCF_3$	1	full	61	>20:1	---	80:20
18	heptane	1	full	41	>20:1	---	88:12
19	DMF	2	full	65	>20:1	---	68:32

Reactions conditions: **1a** (0.1 mmol), **2a** (0.12 mmol), DABCO (0.15 mmol), pre-C3 (20 mol%) in **Solvent** (1.0 mL) at room temperature. [a] Determined by crude TLC analysis. [b] Isolated after column chromatography. [c] Determined by chiral HPLC. [d] Determined by ^1H NMR. [e] After recrystallization from *i*PrOH. [f] Na_2CO_3 as a base (0.15 mmol). er = enantiomeric ratio

Table S4. Additive screening.

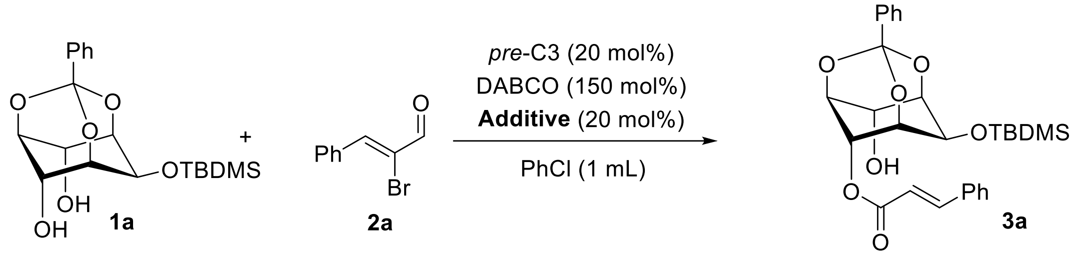

Entry	Additive	Time (h)	Conversion[a]	Yield (mono, %)[b]	*E*/*Z*[d]	*er* (*E*/*Z*, %)[c]
1	4Å MS	1	full	76	>20:1	85:15
2	H_2O[e]	1	full	41	>20:1	85:15
3	LiCl	1	full	86	>20:1	84.5:16.5
4	$Yb(OTf)_3$	18	full	63	>20:1	81.5:18.5
5	(*S*)-CSA	18	full	43	>20:1	85.5:14.5
6	(*R*)-CPA-1	2	full	54	>20:1	84:16
7	Takemoto	2	full	44	>20:1	84:16

Reactions conditions: **1a** (0.1 mmol), **2a** (0.12 mmol), DABCO (0.15 mmol), pre-C3 (20 mol%), **Additive** (20 mol%) in PhCl (1.0 mL) at room temperature. [a] Determined by crude TLC analysis. [b] Isolated after column chromatography. [c] Determined by chiral HPLC. [d] Determined by ^{1}H NMR. [e] 50 mol%. er = enantiomeric ratio, (S)-CSA = (*S*)-camphorsulfonic acid, (*R*)-CPA-1 =

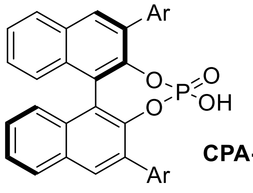

CPA-1: Ar = Tripp

Takemoto =

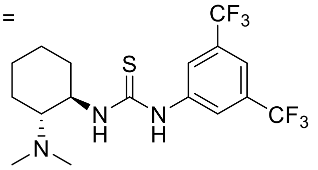

Table S5. Solvent ratio screening.

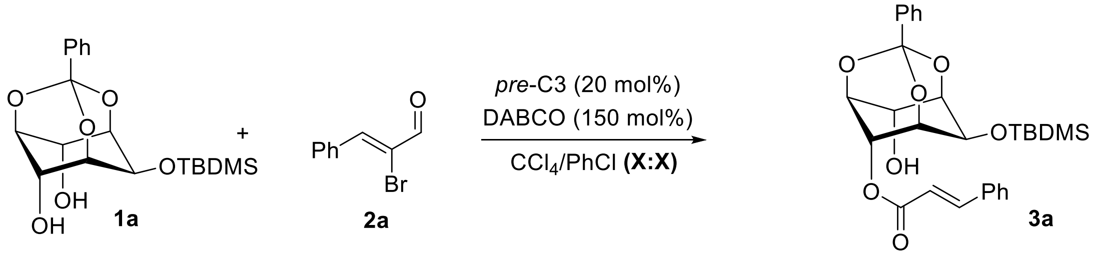

Entry	Ratio	Time (h)	Conversion[a]	Yield (mono, %)[b]	*E*/*Z*[d]	*er* (*E*/*Z*, %)[c]
1	1:0	18	full	57	>20:1	91:9 (>99:1)[e]
2	0:1	1	full	61	>20:1	85:15
3	1:1	2	full	62	>20:1	87:13
4	2:1	2	full	71	>20:1	88.5:11.5
5	4:1	2	full	69	>20:1	89:11
6	8:1	2	full	63	>20:1	89:11
7	1:2	2	full	67	>20:1	85.5:14.5

Reactions conditions: **1a** (0.1 mmol), **2a** (0.12 mmol), DABCO (0.15 mmol), pre-C3 (20 mol%) in CCl_4/PhCl (1.0 mL) at room temperature. [a] Determined by crude TLC analysis. [b] Isolated after column chromatography. [c] Determined by chiral HPLC. [d] Determined by ^{1}H NMR. [e] After recrystallization from *i*PrOH. er = enantiomeric ratio

Table S6. Reagent ratio screening.

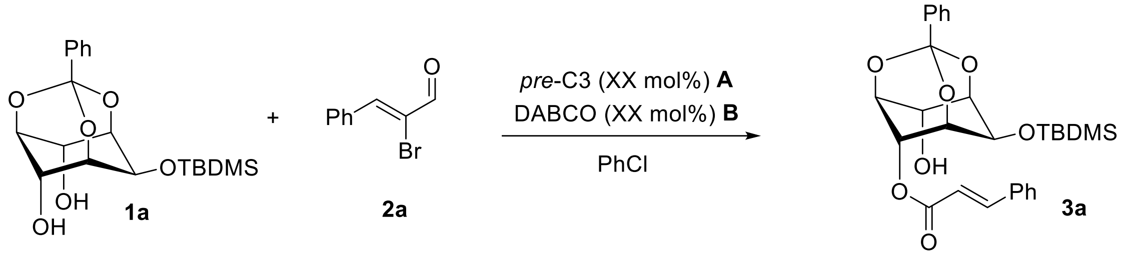

Entry	A	B	2a	Time (h)	Conversion[a]	Yield (mono, %)[b]	*E*/*Z*[d]	*er* (*E/Z*, %)[c]
1	20	150	120	1	full	61	>20:1	85:15
2	10	150	120	18	full	47	>20:1	84.5:15.5
3	5	150	120	18	full	51	>20:1	84:16
4	1	150	120	18	full	53	>20:1	84:16
5	1	300	120	18	full	26	>20:1	87:13
6	1	200	120	18	full	39	>20:1	85:15
7	1	100	120	114	not full	59	>20:1	81:19
8	1	50	120	114	not full	51	>20:1	79:21
9	1	150	150	18	full	65	>20:1	85:15
10	1	150	110	18	full	45	>20:1	81.5:18.5

Reactions conditions: **1a** (0.1 mmol), **2a** (**C** mmol), DABCO (**B** mmol), pre-C3 (**A** mol%) in PhCl (1.0 mL) at room temperature. [a] Determined by crude TLC analysis. [b] Isolated after column chromatography. [c] Determined by chiral HPLC. [d] Determined by ^{1}H NMR. er = enantiomeric ratio

Table S7. Concentration screening.

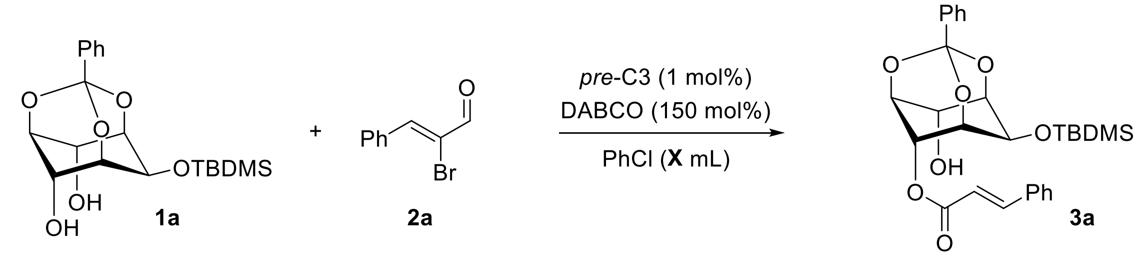

Entry	X	Time (h)	Conversion[a]	Yield (mono, %)[b]	*E*/*Z*[d]	*er* (*E/Z*, %)[c]
1	1.0	18	full	65	>20:1	85:15
2	0.5	18	full	65	>20:1	83:17
3	2.0	18	full	53	>20:1	86:14
4	4.0	18	not full	55	>20:1	87:13

Reactions conditions: **1a** (0.1 mmol), **2a** (0.12 mmol), DABCO (0.15 mmol), pre-C3 (1 mol%) in PhCl (**X** mL) at room temperature. [a] Determined by crude TLC analysis. [b] Isolated after column chromatography. [c] Determined by chiral HPLC. [d] Determined by ^{1}H NMR. er = enantiomeric ratio

Table S8. Temperature screening.

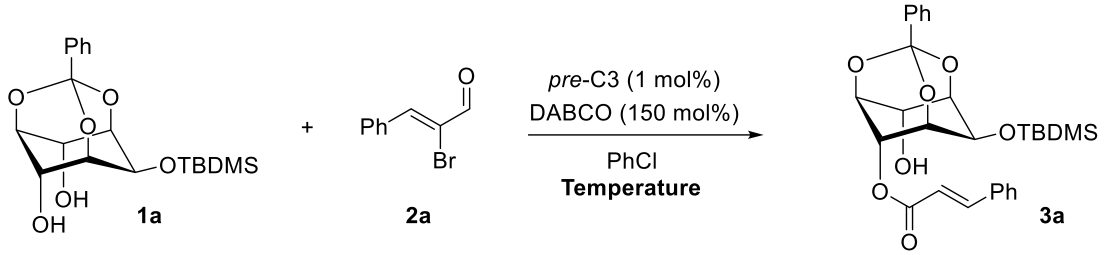

Entry	Temp. (°C)	Time (h)	Conversion[a]	Yield (mono, %)[b]	*E*/*Z*[d]	*er* (*E*/*Z*, %)[c]
1	21	18	full	65	>20:1	85:15
2	40	18	full	63	>20:1	83.5:16.5
3	0	42	not full	53	>20:1	86:14

Reactions conditions: **1a** (0.1 mmol), **2a** (0.12 mmol), DABCO (0.15 mmol), pre-C3 (1 mol%) in PhCl (1 mL) at different **temperature**. [a] Determined by crude TLC analysis. [b] Isolated after column chromatography. [c] Determined by chiral HPLC. [d] Determined by ^{1}H NMR. er = enantiomeric ratio

Crystallographic data

X-ray single crystal experiment for **3a** was performed on Bruker D8 VENTURE Kappa Duo PHOTONIII by IμS micro-focus sealed tube CuKα (λ= 1.54178 Å), equipped with Oxford Cryostream Coller 800. The structure was solved by direct methods (XT)[1] and refined by full matrix least squares based on F^2 (SHELXL2019).[2] Hydrogen atoms were fixed into idealized positions (riding model) and assigned temperature factors either $H_{iso}(H) = 1.2\ U_{eq}$(pivot atom) or 1.5 U_{eq}(pivot atom) for methyl moiety. The hydrogen atom in -O-H moiety was found on difference Fourier map and refined under an assumption of rigid body with displacement parameter $H_{iso}(H) = 1.5\ U_{eq}(O5)$. The determination of absolute structure was based of anomalous dispersion of Si and oxygen atoms.

Crystal data for **3a,** $C_{28}H_{34}O_7Si$, M_r = 510.64; Monoclinic, $P2_1$ (No 4), a = 11.3495 (4) Å, b = 6.8068 (2) Å, c = 17.6152 (6) Å, β = 92.615 (1)°, V = 1359.42 (8) Å^3, Z = 2, D_x = 1.247 Mg m^{-3}, temperature of sample 120(2) K, colorless needle of dimensions 0.51 × 0.05 × 0.03 mm, multi-scan absorption correction (μ = 1.12 mm^{-1}), T_{min} = 0.599, T_{max} = 0.971; a total of 36164 measured reflections (θ_{max}= 79.4°), from which 5775 were unique (R_{int} = 0.031) and 5634 observed according to the $I > 2\sigma(I)$ criterion. The refinement converged ($\Delta/\sigma_{max} < 0.001$) to R = 0.37 for observed reflections and $wR(F^2)$ = 0.102, GOF = 1.03 for 330 parameters and all 5775 reflections. The final difference map displayed no peaks of chemical significance ($\Delta\rho_{max}$ = 0.52, $\Delta\rho_{min}$ -0.29 e.Å^{-3}). Absolute structure parameter: 0.02(3)[3]

X-ray crystallographic data have been deposited with the Cambridge Crystallographic Data Centre under deposition numbers CCDC 2487498. They can be obtained free of charge from the Centre via its website (https://www.ccdc.cam.ac.uk/structures/).

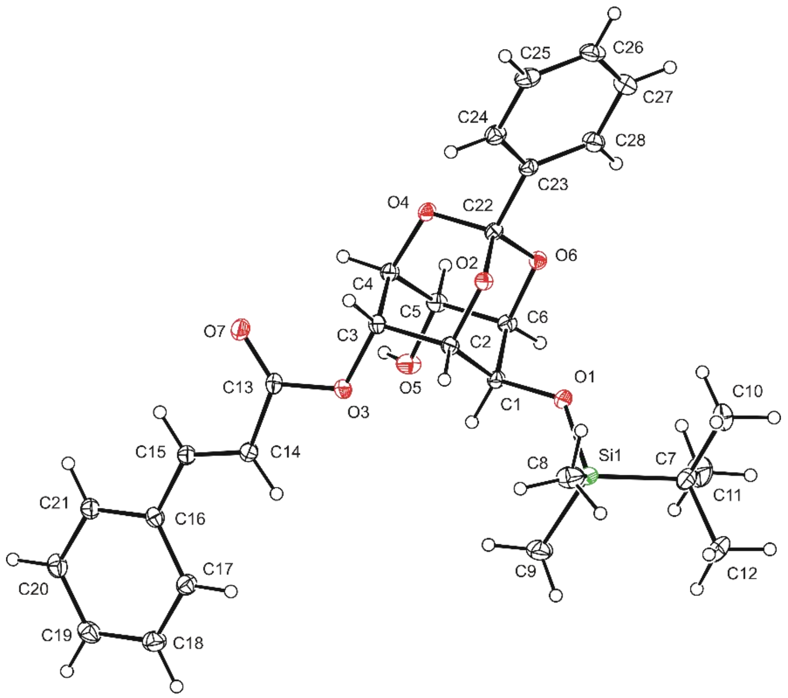

Figure S2. View on molecule of **3a** with atom numbering. Displacement ellipsoids are drawn on 30% probability level (CCDC: 2404192).

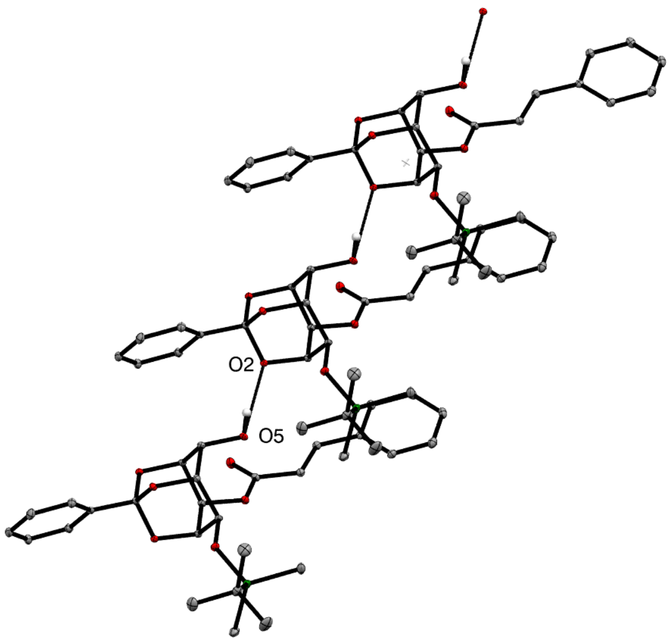

Figure S3. Part of infinite column of **3a** built along two folds screw axis by hydrogen bonds O5-H5…O2 [O5···O2^{i} 2.783 (2) Å, angle at H5 162°]. Symmetry code: (i) x, y+1, z

NMR spectra

Myo-Inositol-1,3,5-orthobenzoate (1a)

^{1}H NMR for **1a** (400 MHz, DMSO-d_6)

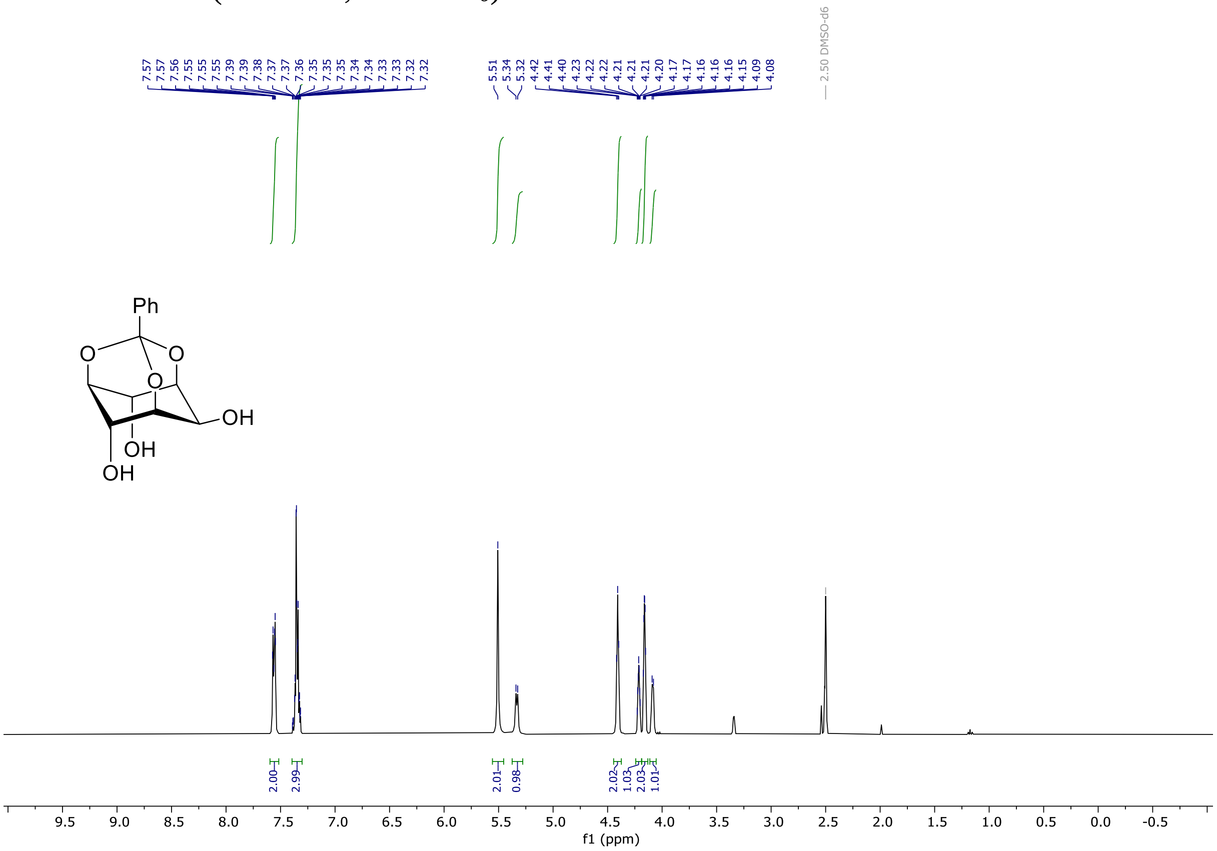

$^{13}C\{^{1}H\}$ NMR for **1a** (101 MHz, DMSO-d_6)

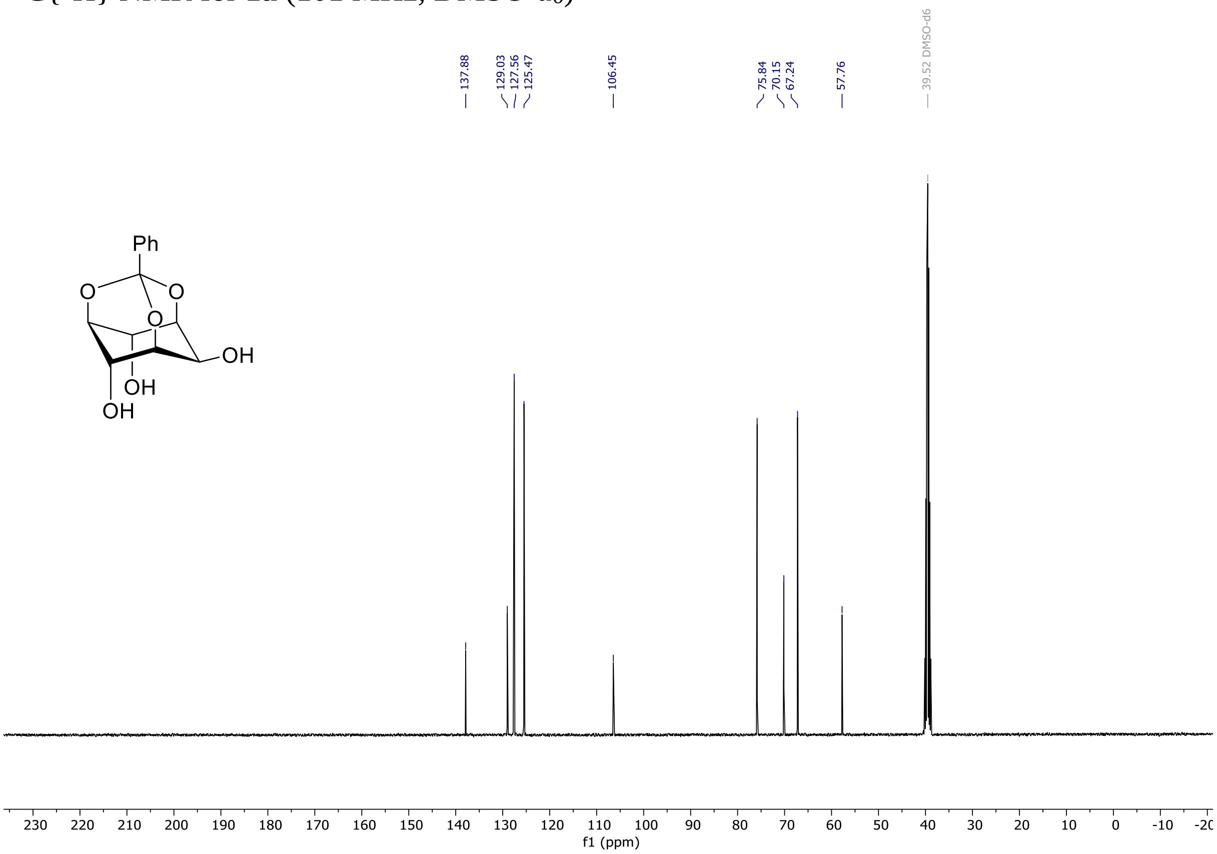

Myo-Inositol-1,3,5-orthoformate (1b)

^{1}H NMR for **1b** (400 MHz, D_2O)

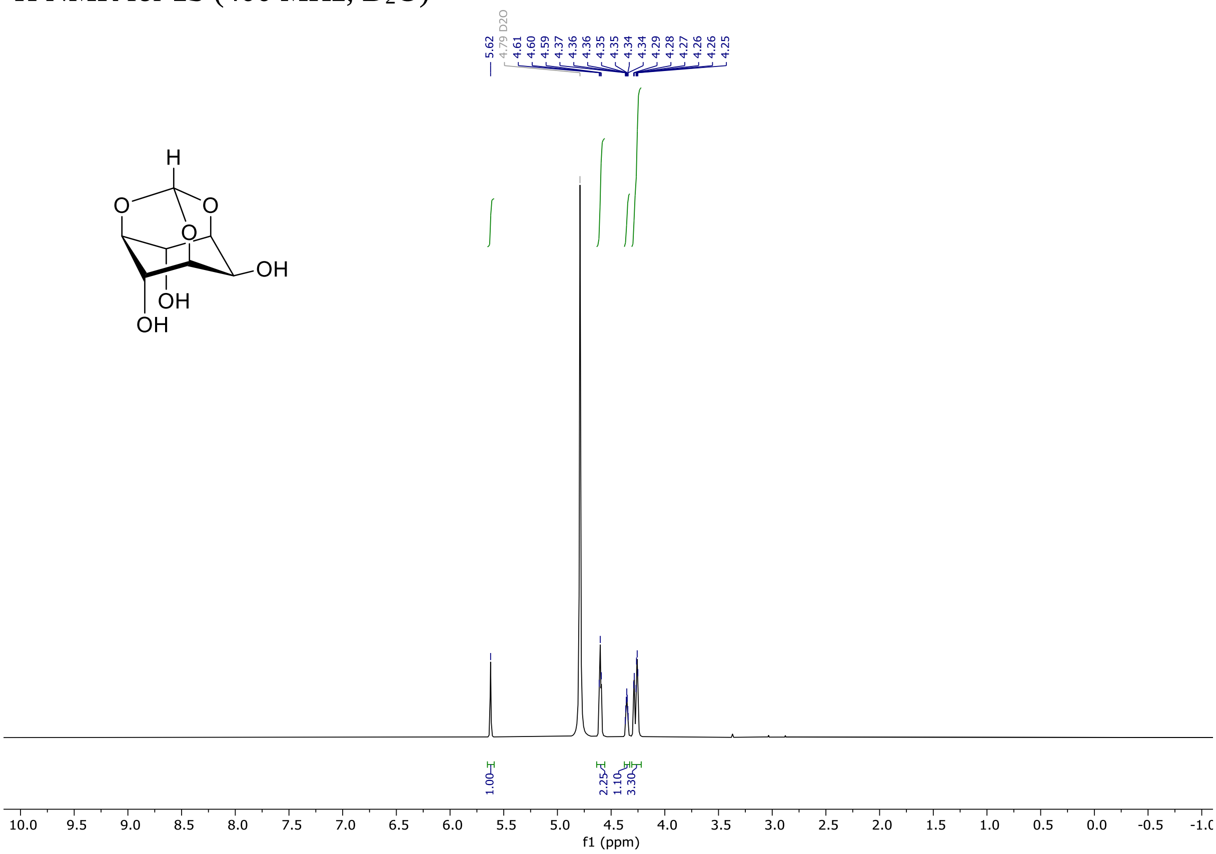

$^{13}C\{^{1}H\}$ NMR for **1b** (101 MHz, D_2O)

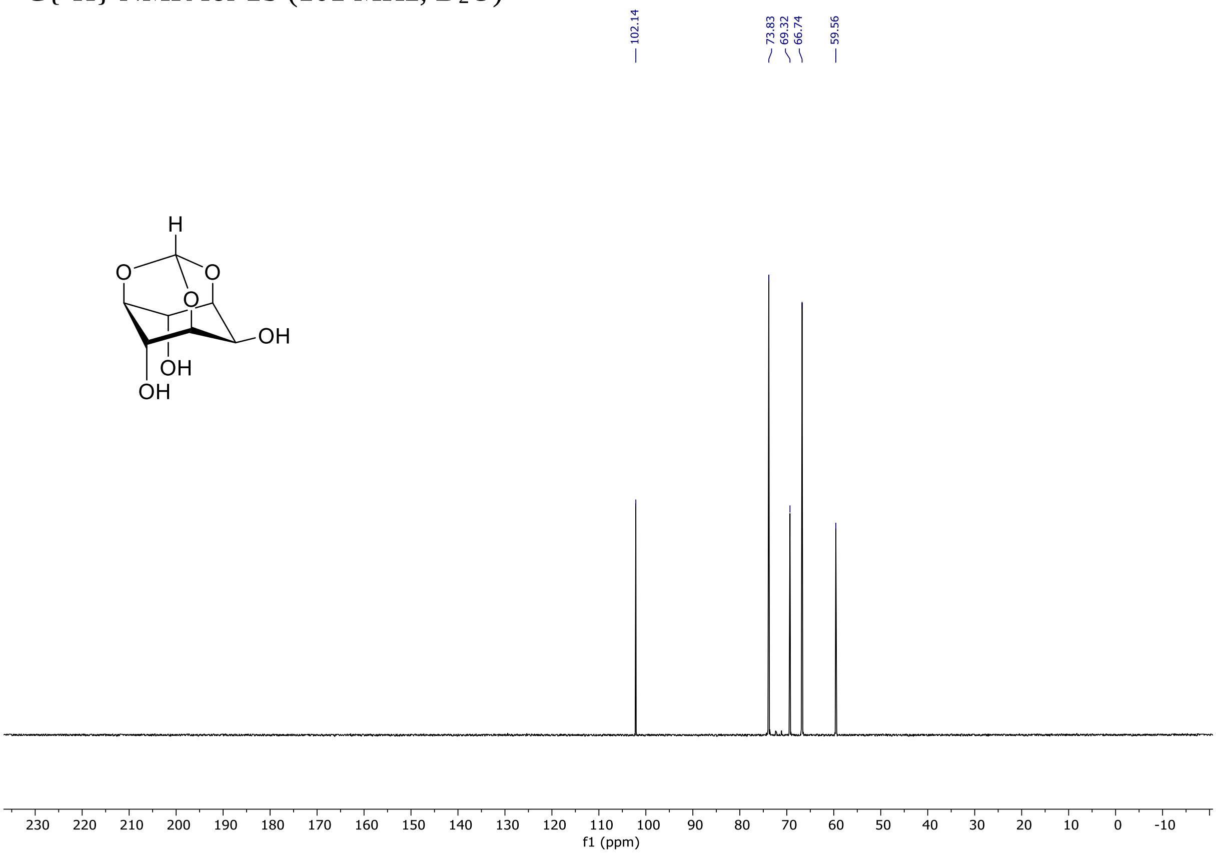

2-*O*-*tert*-Butyldimethylsilyl-*myo*-inositol-1,3,5-orthobenzoate (1c)

^{1}H NMR for **1c** (400 MHz, $CDCl_3$)

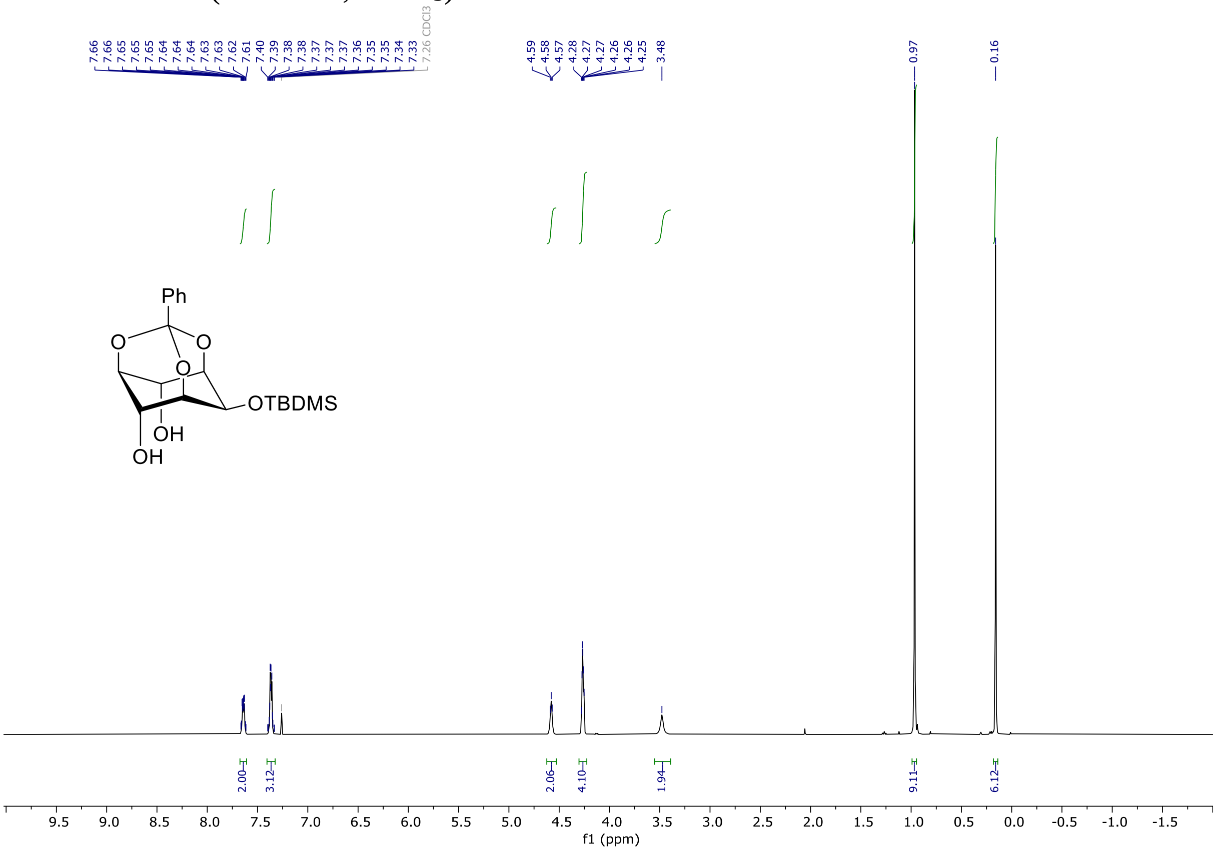

^{13}C{^{1}H} NMR for **1c** (101 MHz, $CDCl_3$)

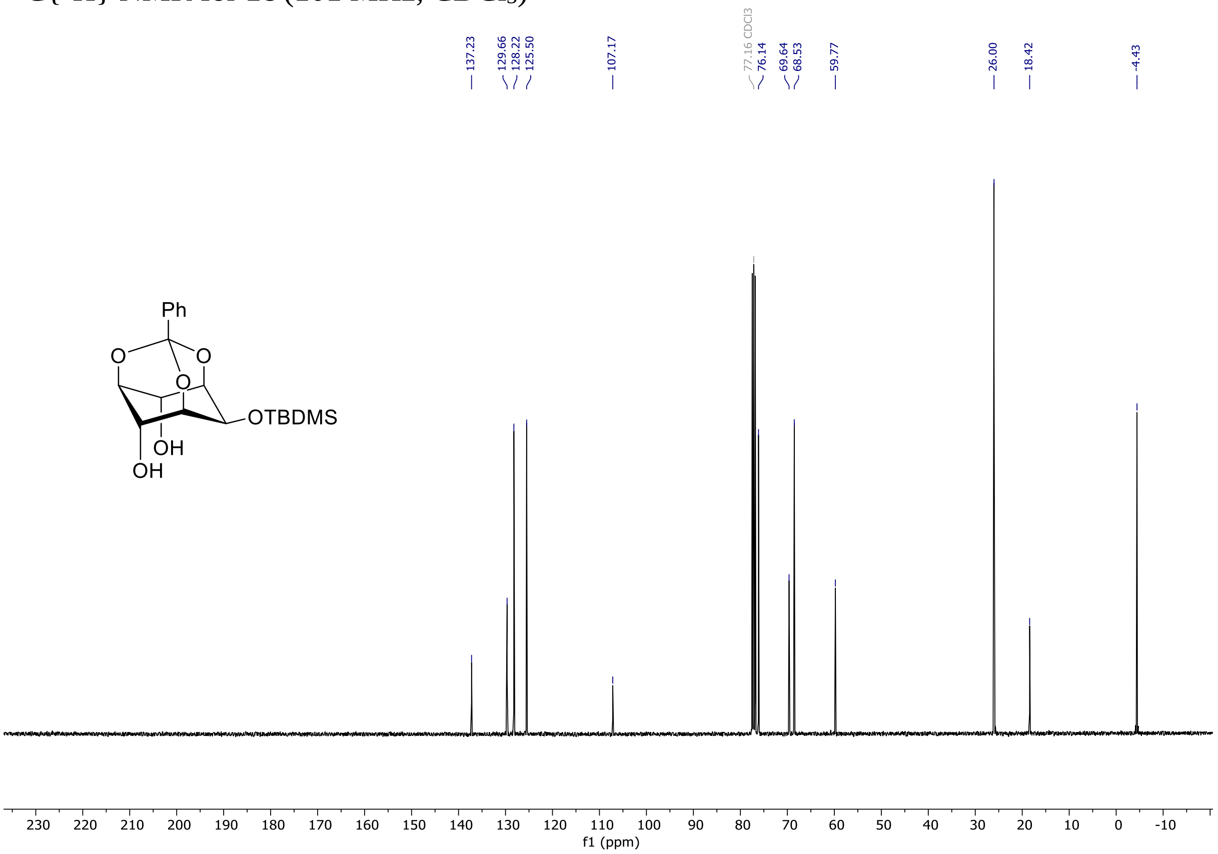

2-*O*-*tert*-Butyldimethylsilyl-*myo*-inositol-1,3,5-orthoformate (1d)

^{1}H NMR for **1d** (400 MHz, $CDCl_3$)

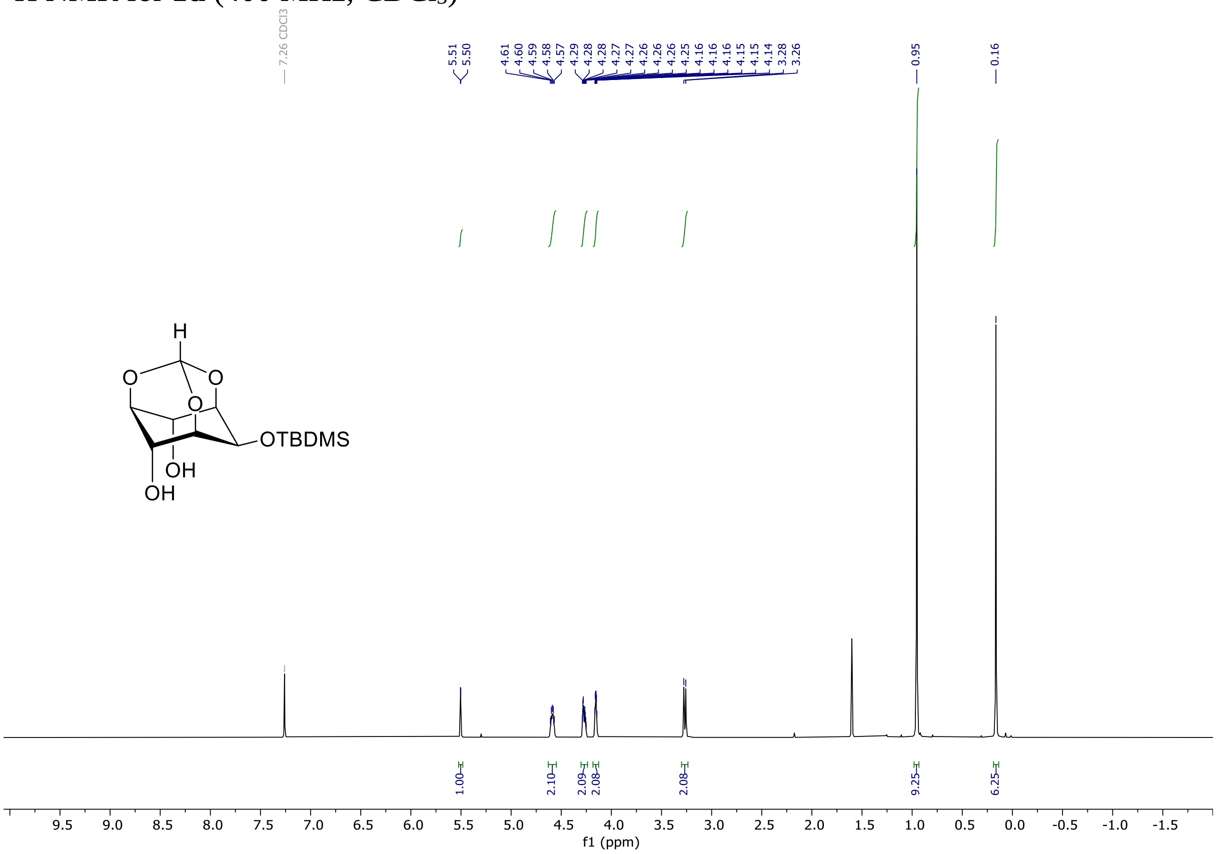

^{13}C{^{1}H} NMR for **1d** (101 MHz, $CDCl_3$)

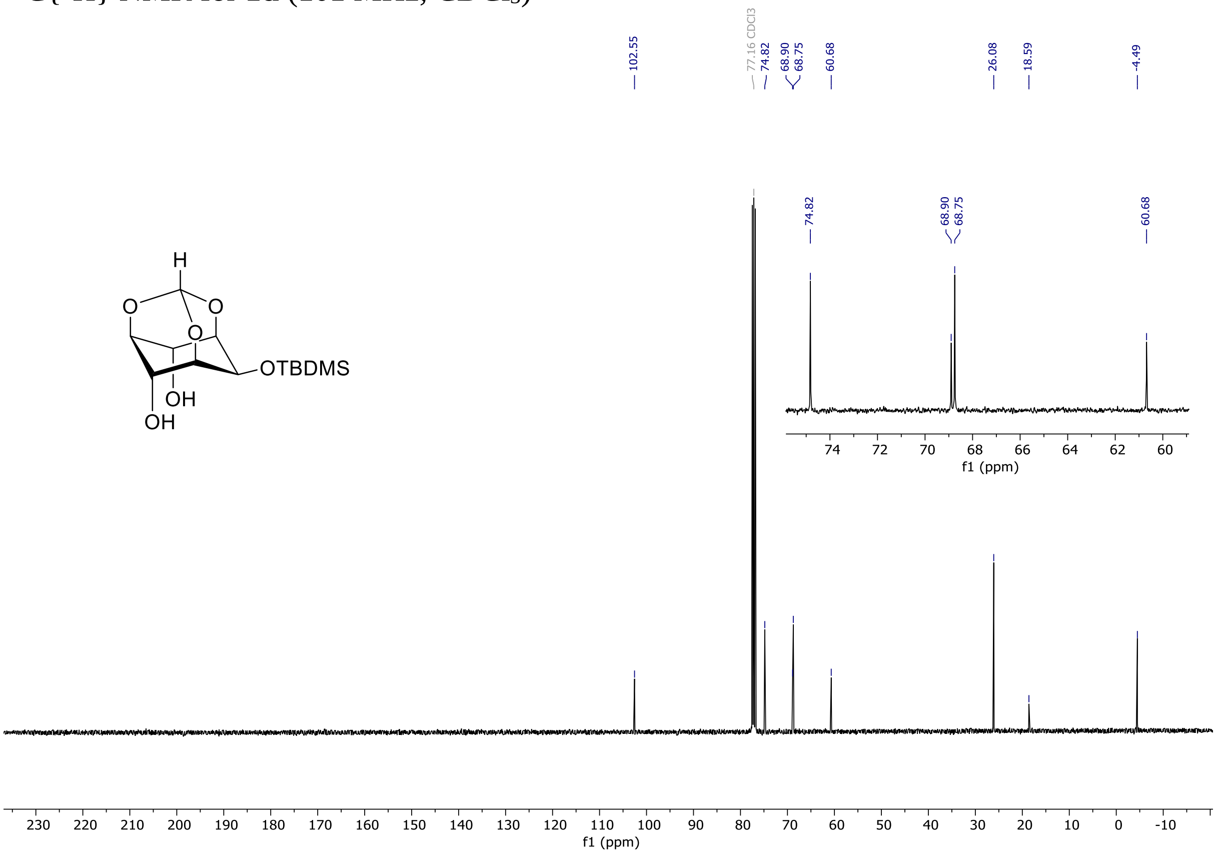

2-*O*-Trimethylsilyl-*myo*-inositol-1,3,5-orthobenzoate (1e)

^{1}H NMR for **1e** (400 MHz, $CDCl_3$)

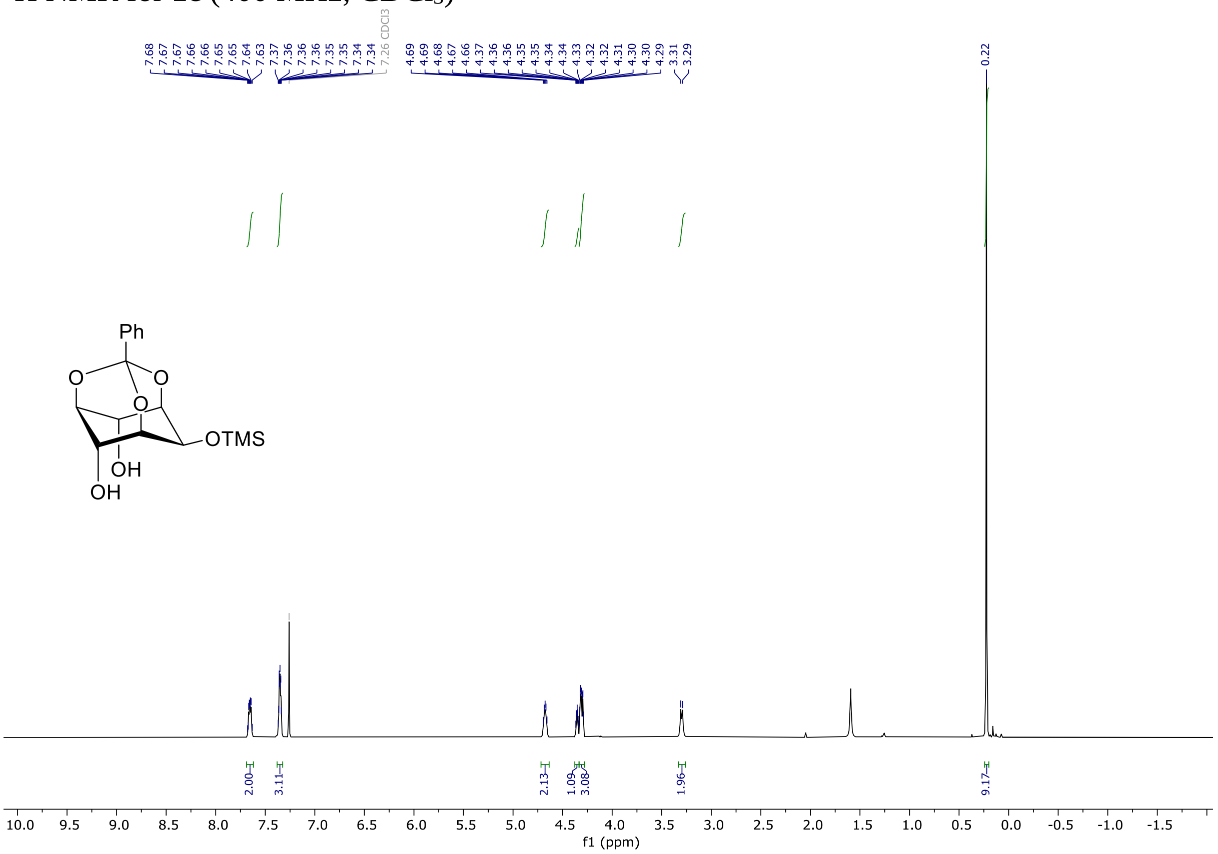

^{13}C{^{1}H} NMR for **1e** (101 MHz, $CDCl_3$)

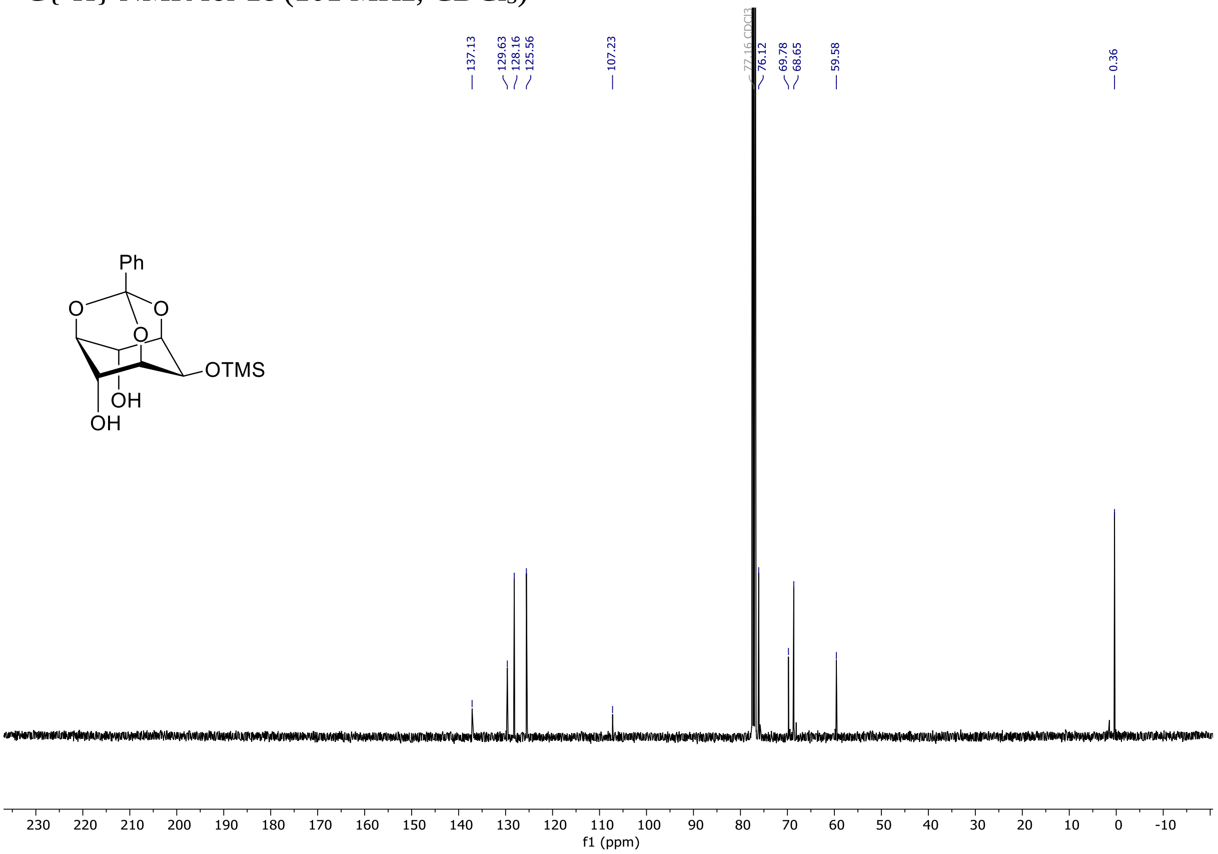

2-*O-tert*-Butyldiphenylsilyl-*myo*-inositol-1,3,5-orthobenzoate (1f)

^{1}H NMR for **1f** (400 MHz, $CDCl_3$)

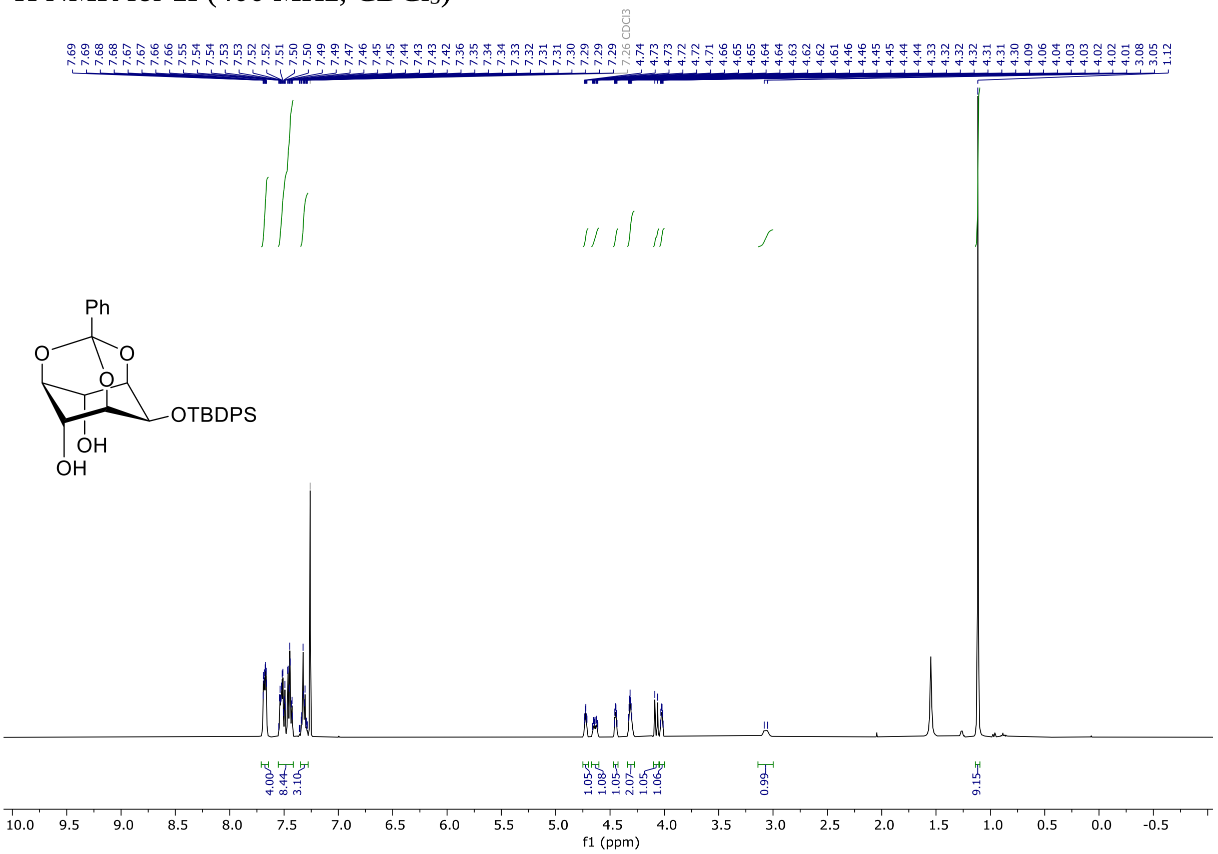

^{13}C{^{1}H} NMR for **1f** (101 MHz, $CDCl_3$)

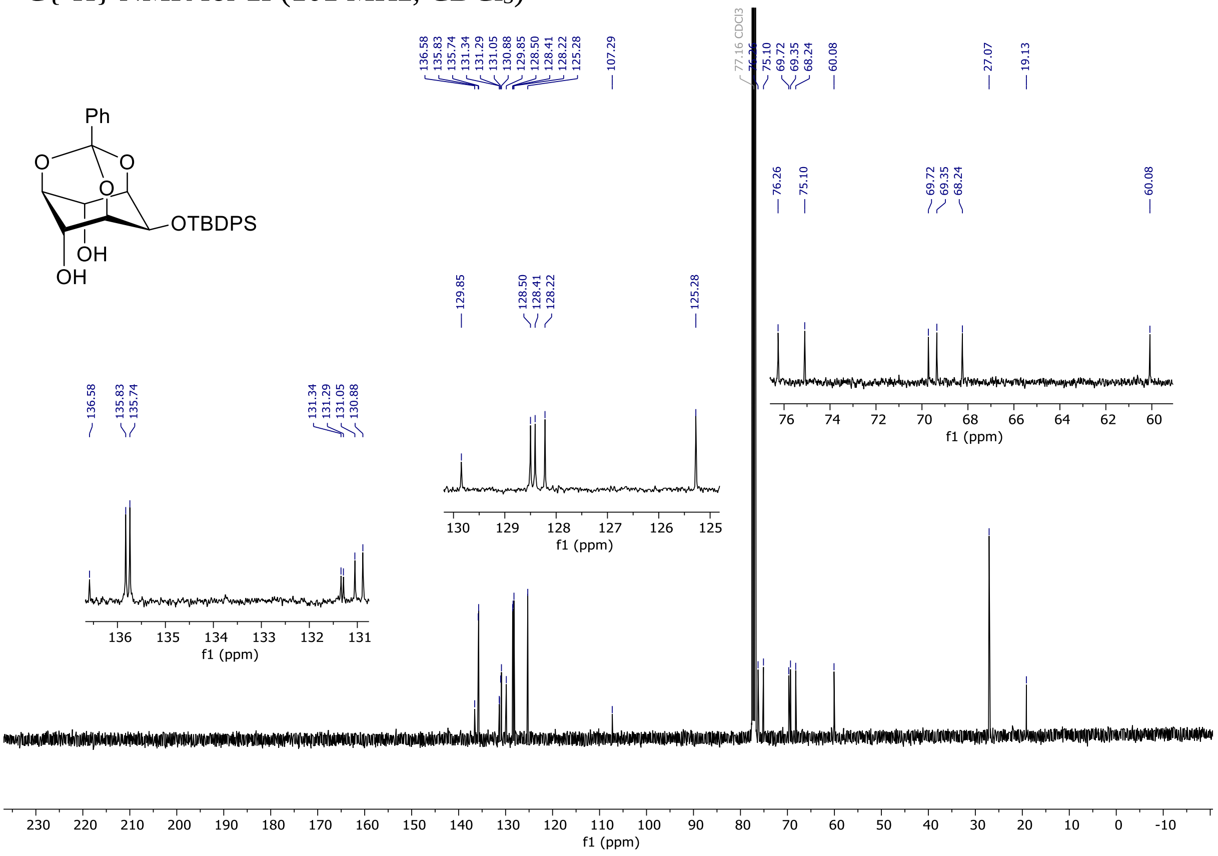

2-*O*-Benzoyl-*myo*-inositol-1,3,5-orthobenzoate (1g)

^{1}H NMR **1g** (400 MHz, DMSO-d_6)

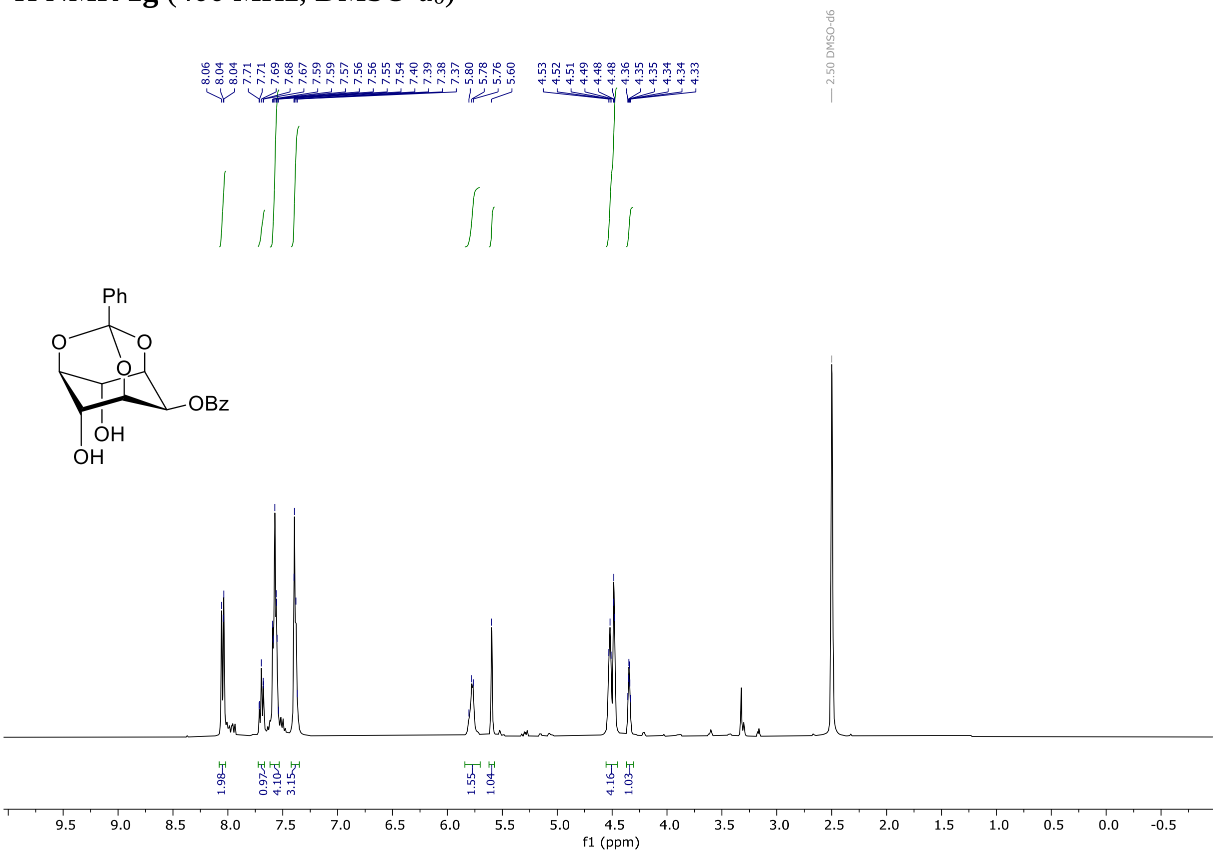

^{13}C{^{1}H} NMR for **1g** (101 MHz, DMSO-d_6)

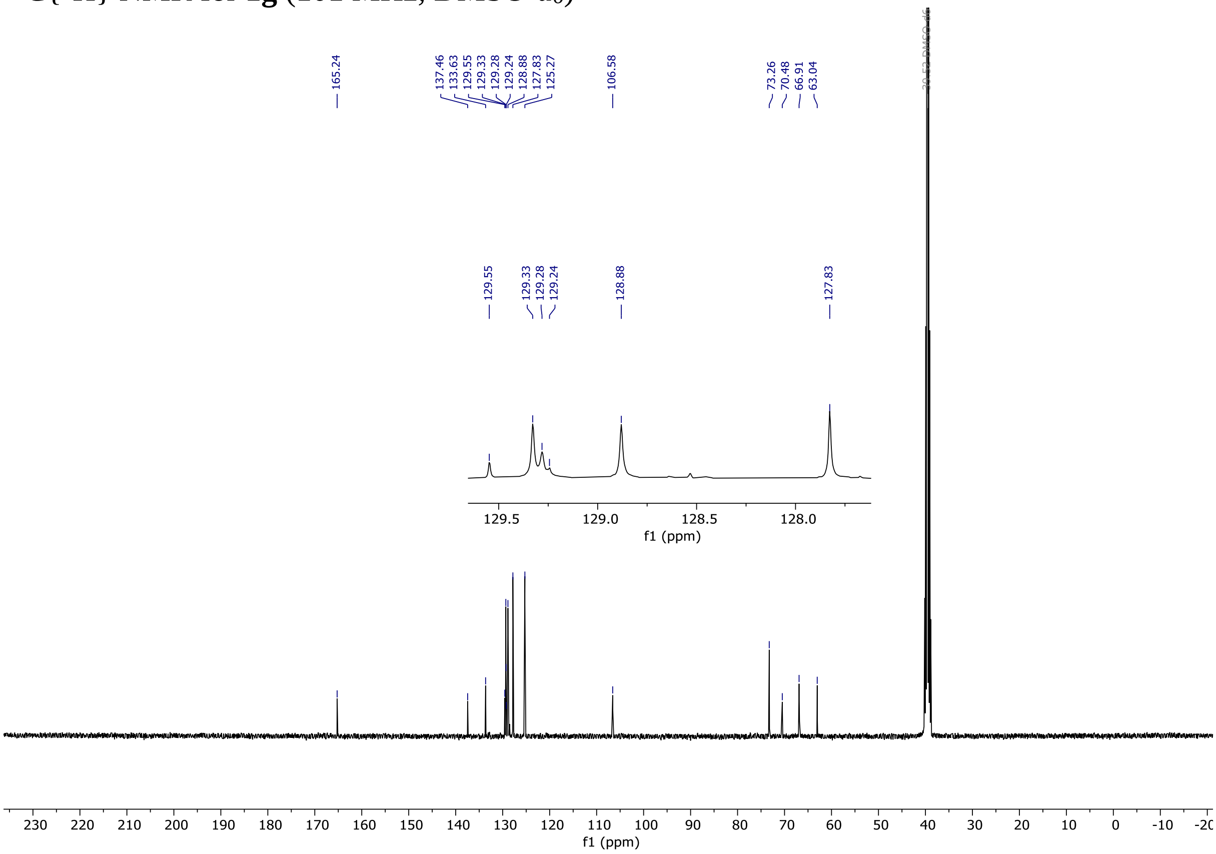

(1*R*,3*R*,5*S*,6*R*,7*S*,8*R*,9*S*)-8-((*tert*-Butyldimethylsilyl)oxy)-9-hydroxy-3-phenyl-2,4,10-trioxaadamantan-6-yl cinnamate (3a)

^{1}H NMR for **3a** (400 MHz, $CDCl_3$)

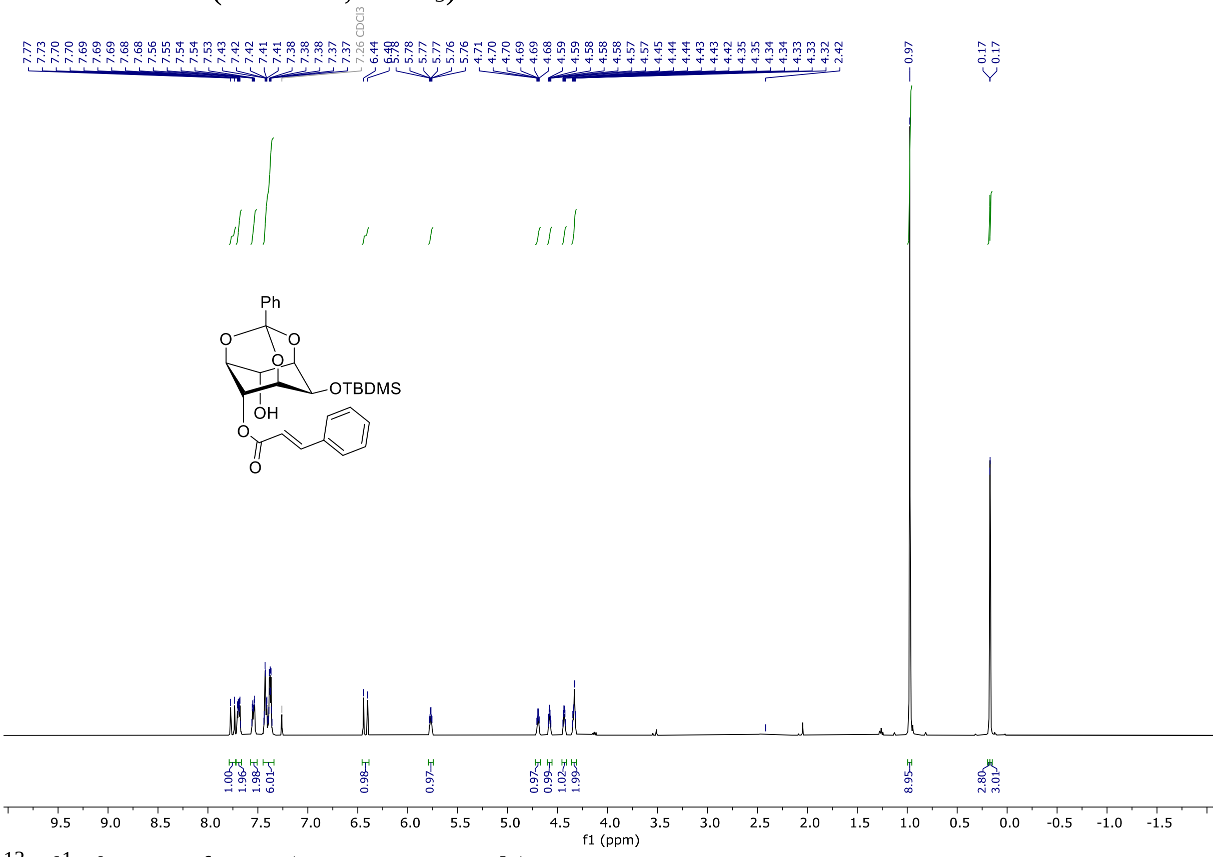

^{13}C{^{1}H} NMR for **3a** (101 MHz, $CDCl_3$)

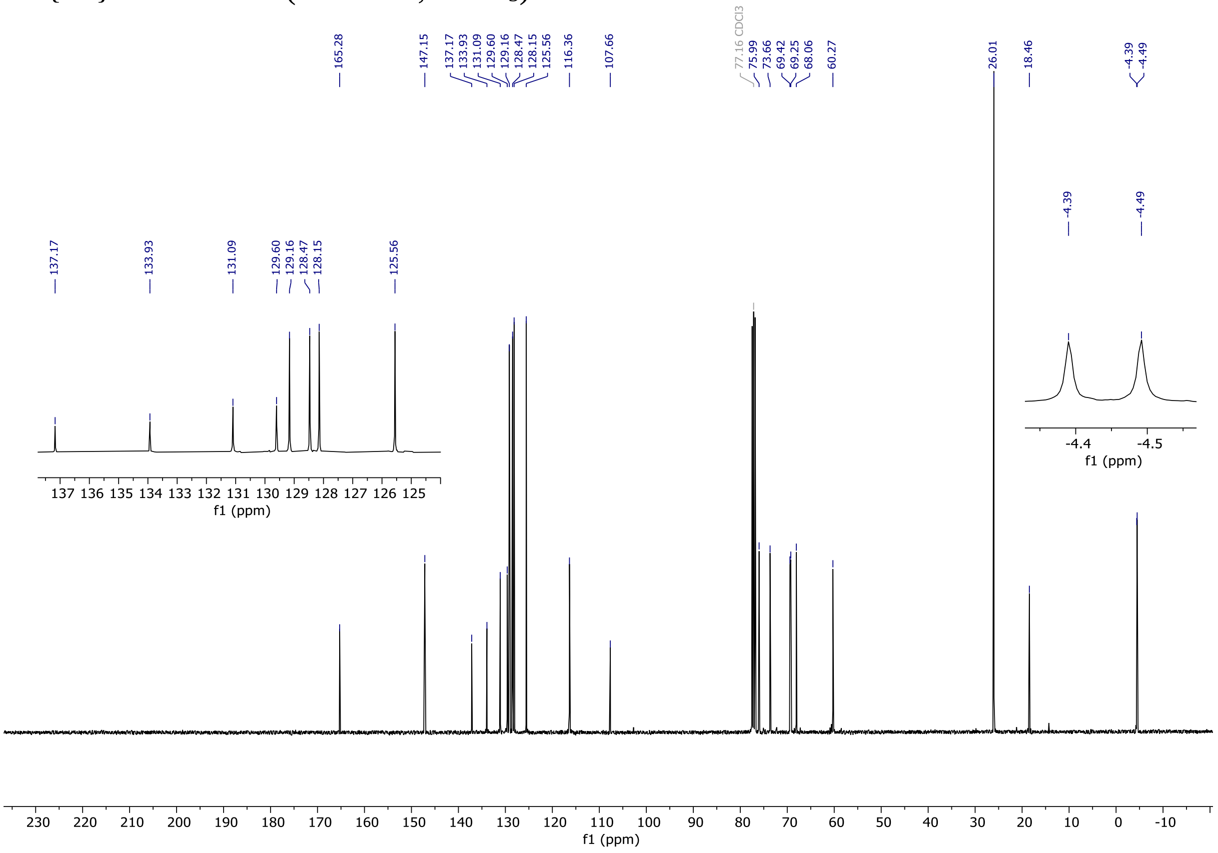

(1*R*,3*R*,5*S*,6*R*,7*S*,8*R*,9*S*)-8-((*tert*-Butyldimethylsilyl)oxy)-9-hydroxy-2,4,10-trioxaadamantan-6-yl cinnamate (3b)

^{1}H NMR for **3b** (400 MHz, $CDCl_3$)

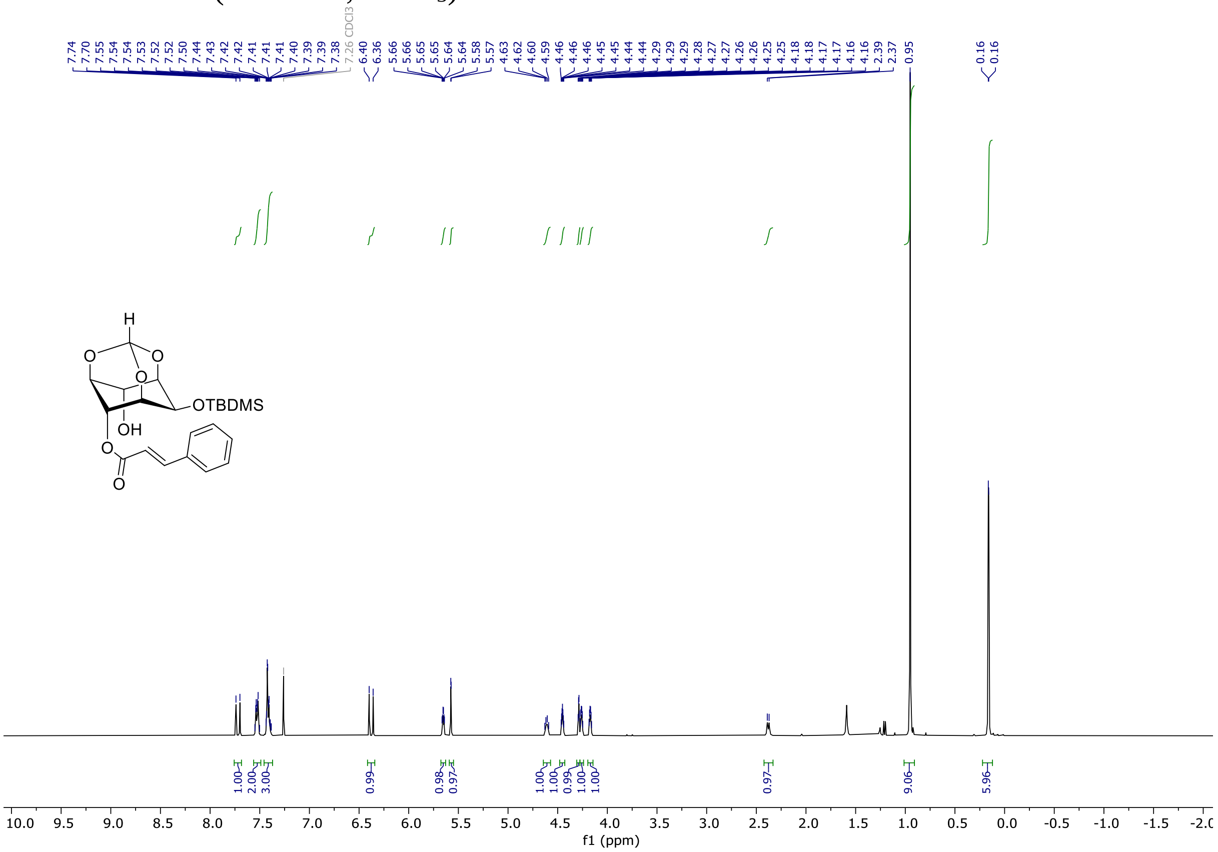

^{13}C{^{1}H} NMR for **3b** (101 MHz, $CDCl_3$)

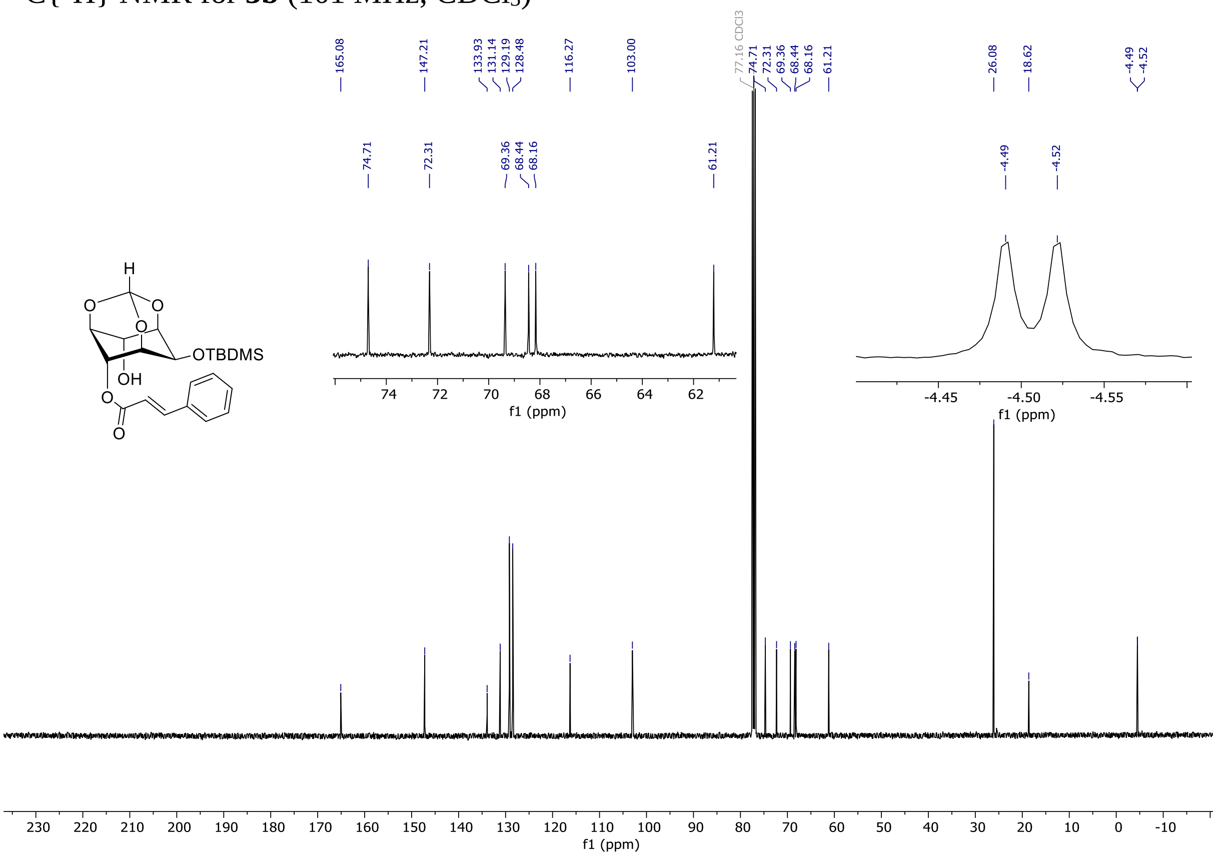

(1*R*,3*R*,5*S*,6*R*,7*S*,8*S*,9*R*)-8-Hydroxy-3-phenyl-9-((trimethylsilyl)oxy)-2,4,10-trioxaadamantan-6-yl cinnamate (3c)

^{1}H NMR for **3c** (400 MHz, $CDCl_3$)

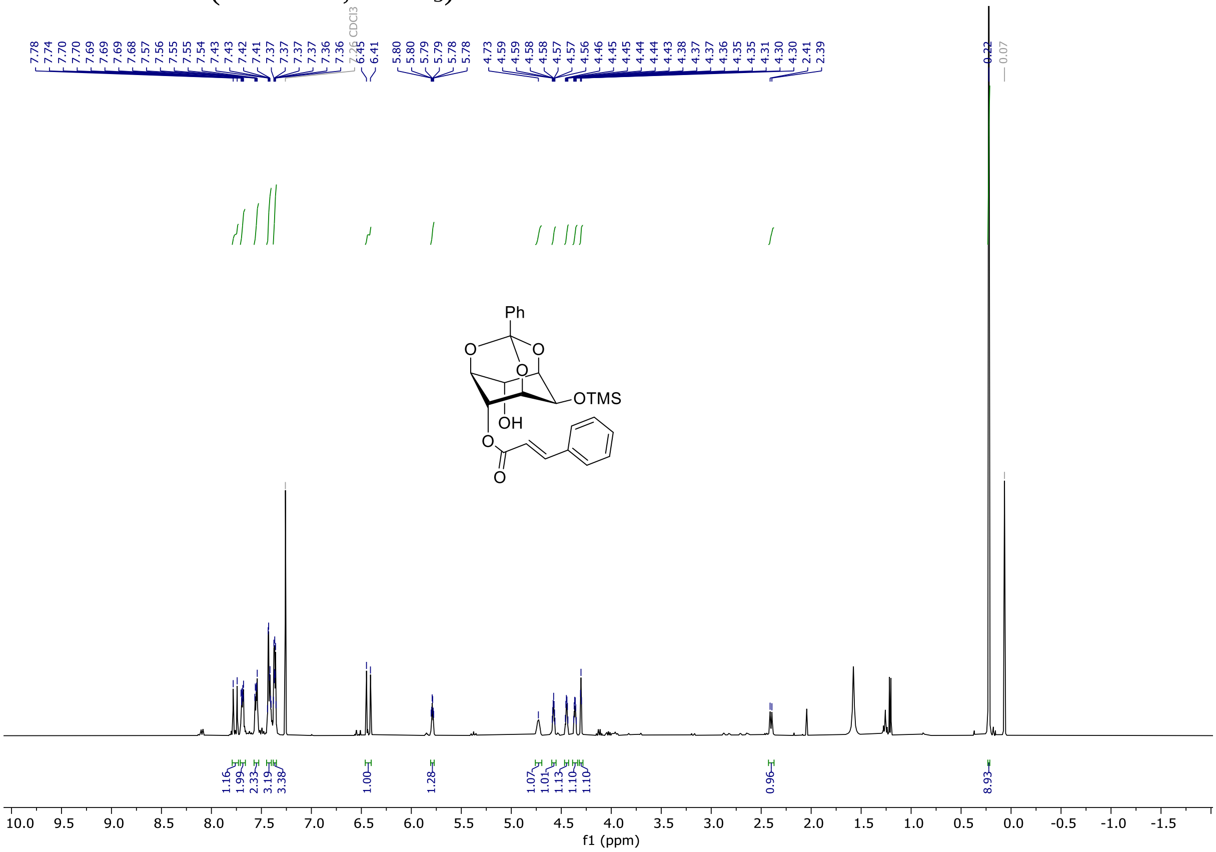

$^{13}C\{^{1}H\}$ NMR for **3c** (101 MHz, $CDCl_3$)

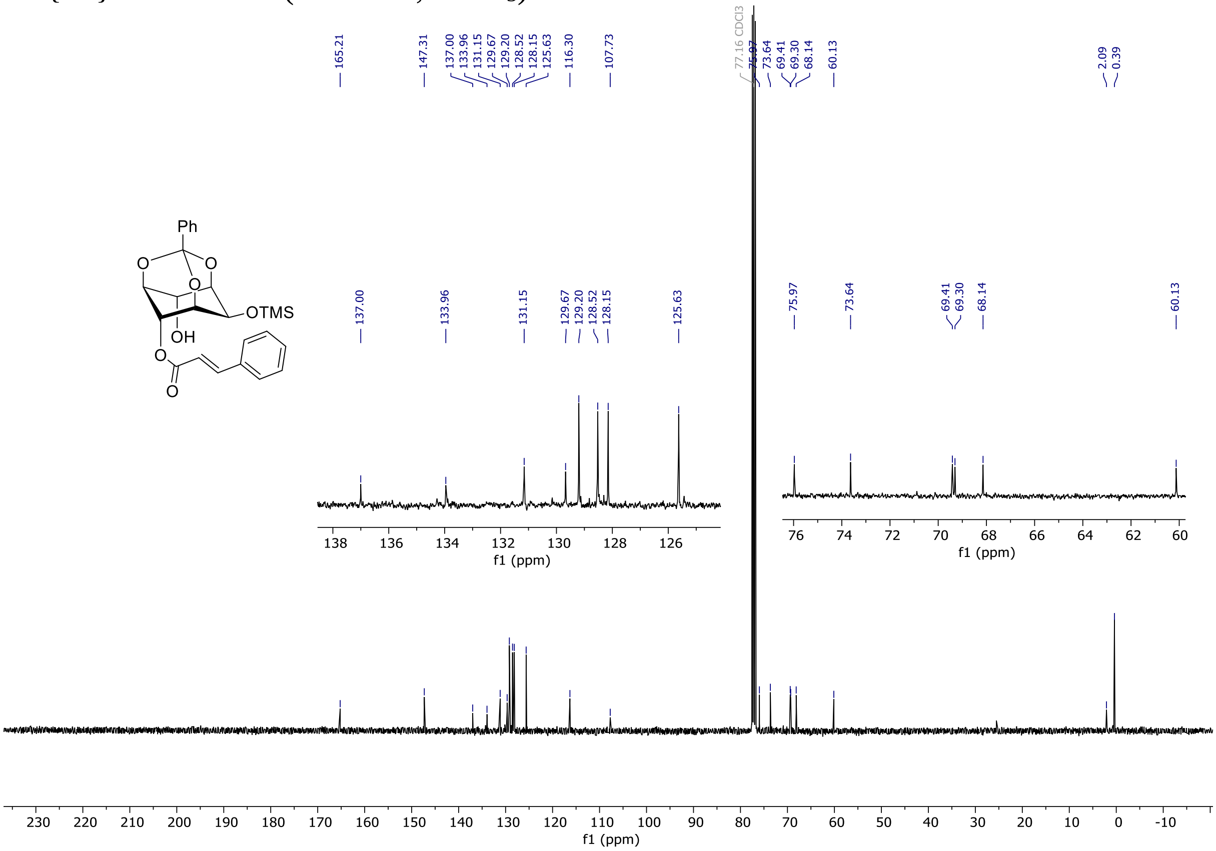

(1*R*,3*R*,5*S*,6*R*,7*S*,8*R*,9*S*)-8-((*tert*-Butyldiphenylsilyl)oxy)-9-hydroxy-3-phenyl-2,4,10-trioxaadamantan-6-yl cinnamate (3d)

^{1}H NMR for **3d** (400 MHz, $CDCl_3$)

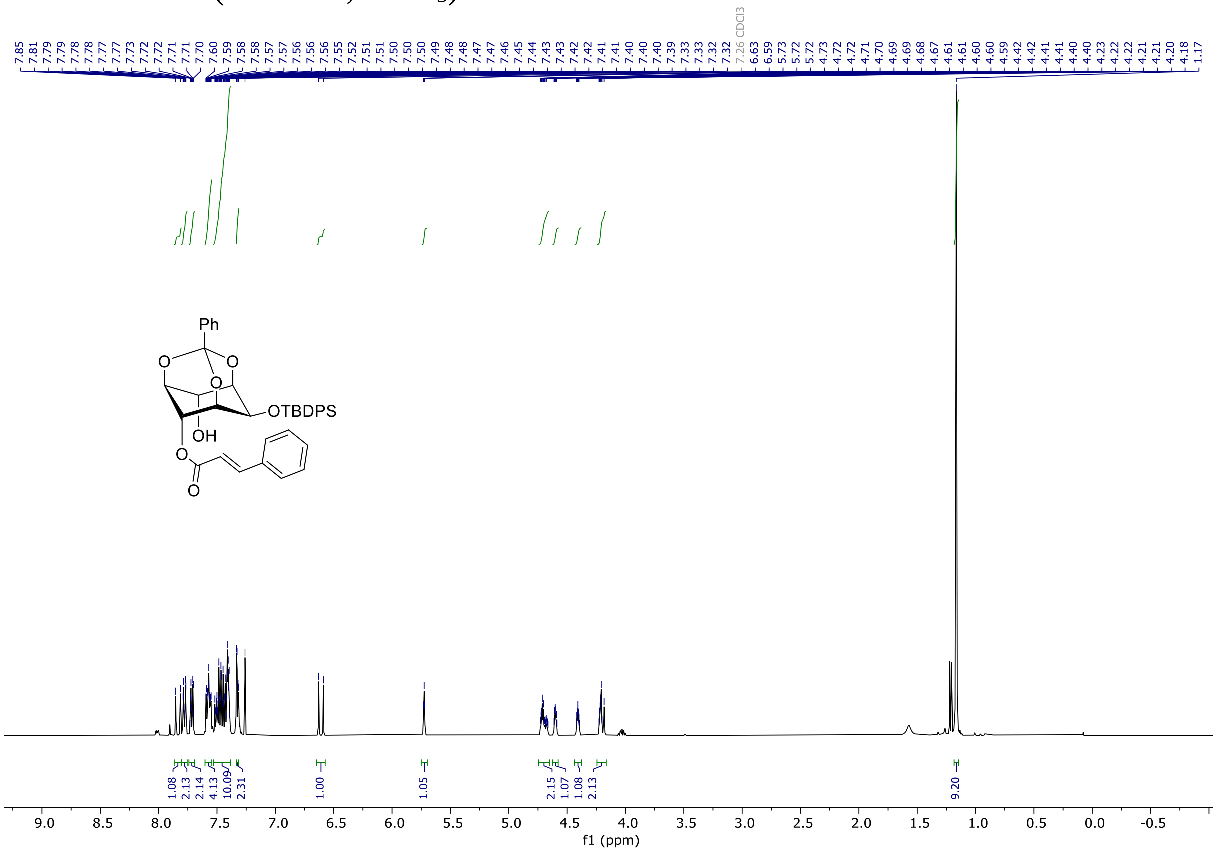

^{13}C{^{1}H} NMR for **3d** (101 MHz, $CDCl_3$)

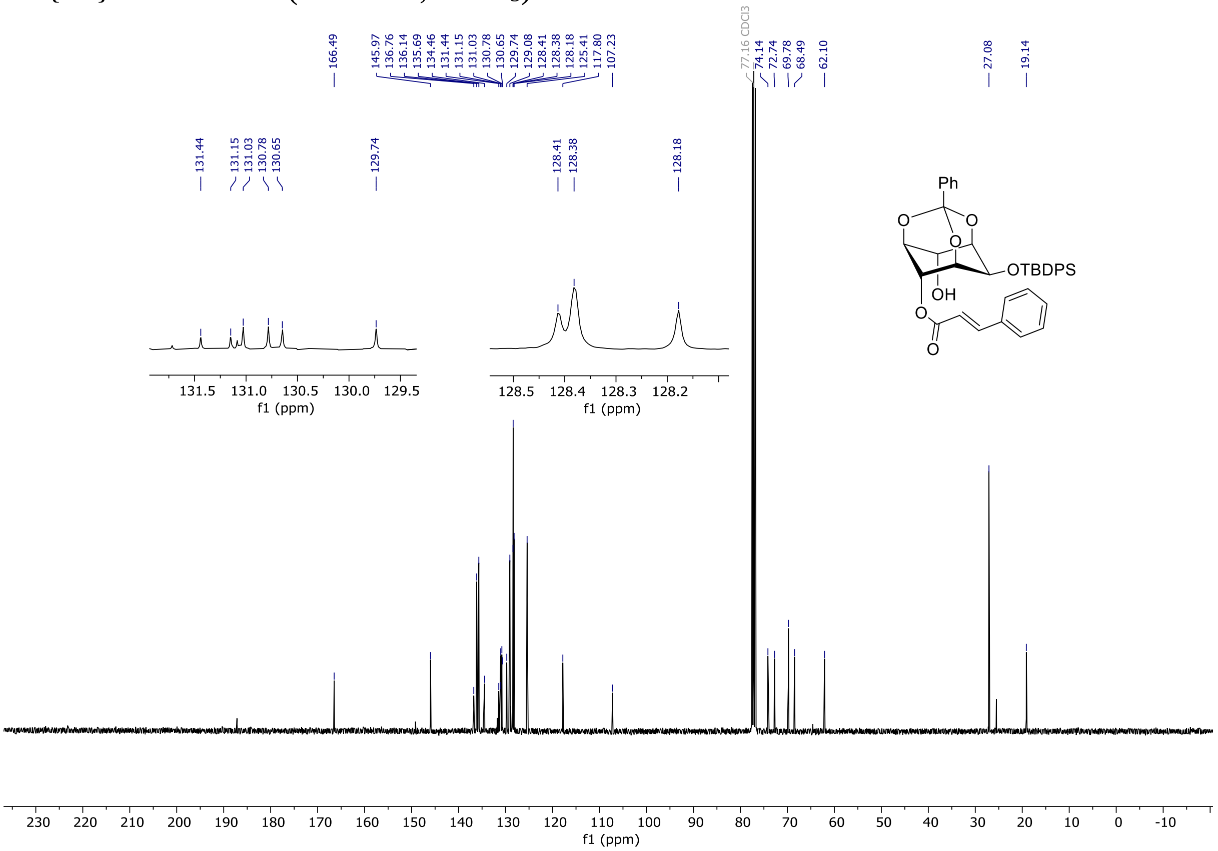

(1*S*,3*R*,5*R*,6*R*,7*S*,8*R*,9*S*)-8-(Cinnamoyloxy)-9-hydroxy-3-phenyl-2,4,10-trioxaadamantan-6-yl benzoate (3e)

^{1}H NMR for **3e** (400 MHz, $CDCl_3$)

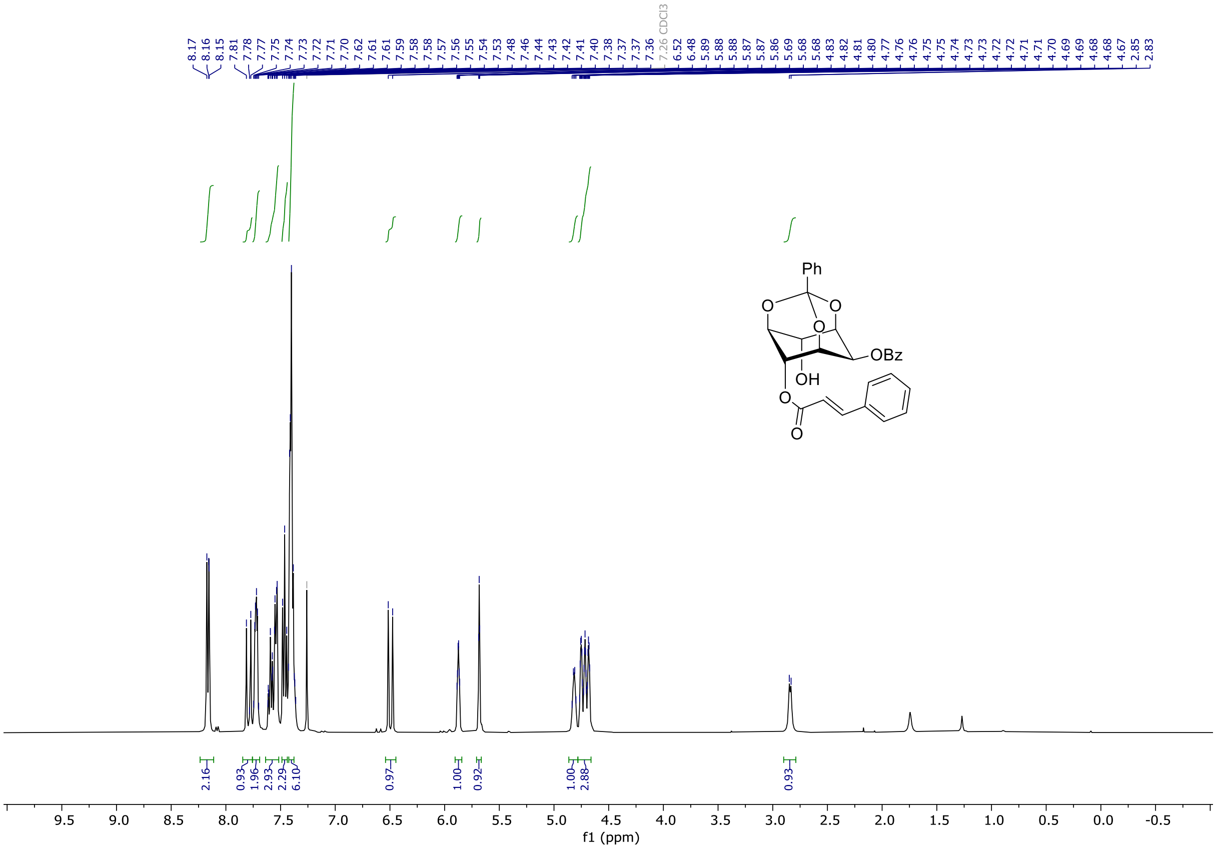

^{13}C{^{1}H} NMR for **3e** (101 MHz, $CDCl_3$)

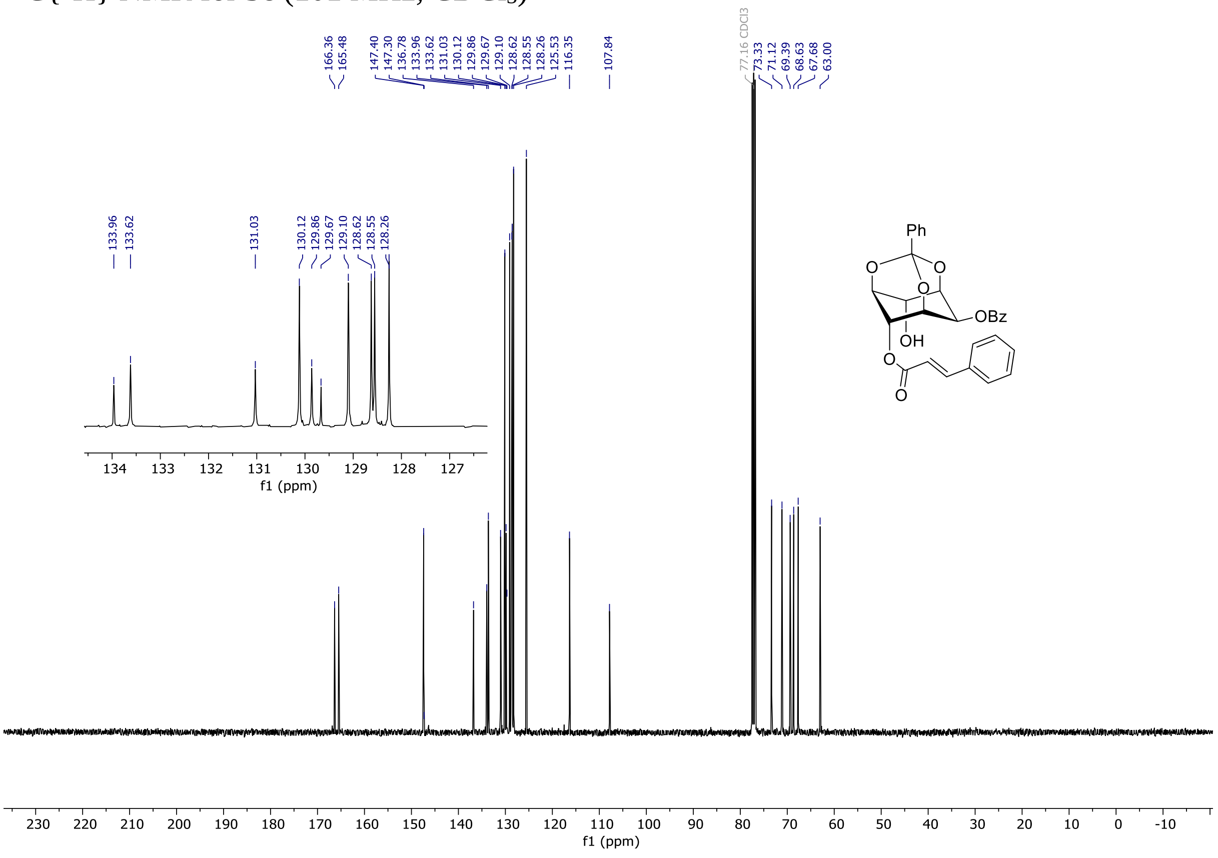

(1*R*,3*R*,5*S*,6*R*,7*S*,8*R*,9*S*)-9-Hydroxy-3-phenyl-2,4,10-trioxaadamantane-6,8-diyl (2*E*,2'*E*)-bis(3-phenylacrylate) (3f)

^{1}H NMR for **3f** (400 MHz, $CDCl_3$)

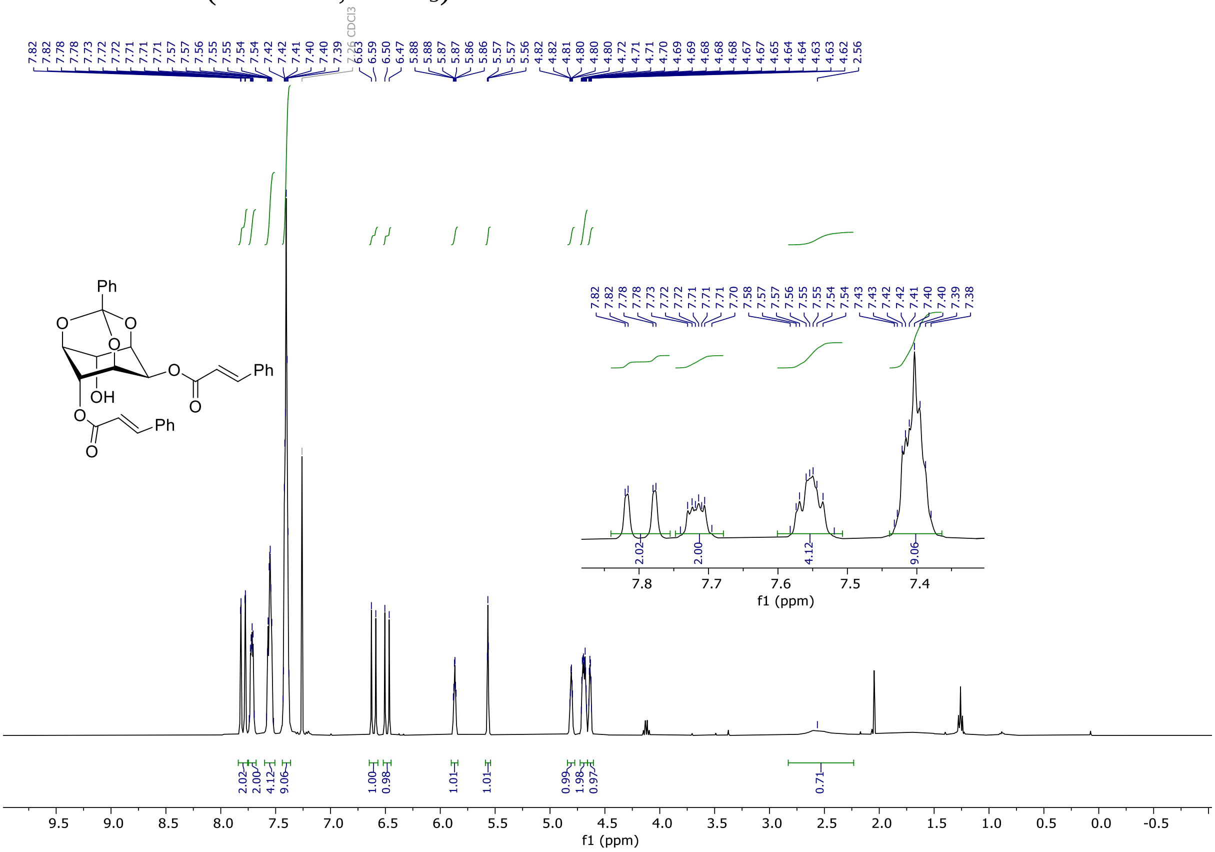

^{13}C{^{1}H} NMR for **3f** (101 MHz, $CDCl_3$)

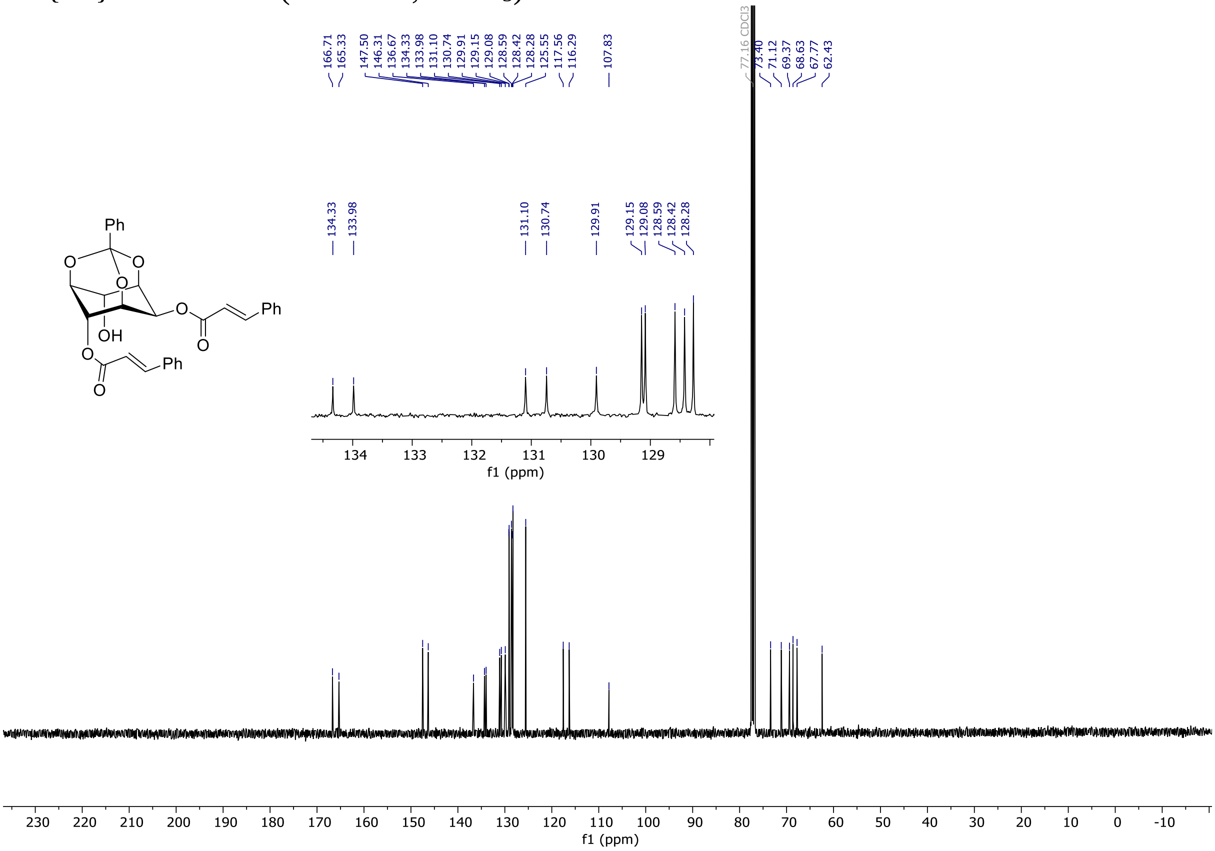

(1*R*,3*R*,5*S*,6*R*,7*S*,8*R*,9*S*)-8-((*tert*-Butyldimethylsilyl)oxy)-9-hydroxy-3-phenyl-2,4,10-trioxaadamantan-6-yl (*E*)-3-(naphthalen-2-yl)acrylate (3g)

^{1}H NMR for **3g** (400 MHz, $CDCl_3$)

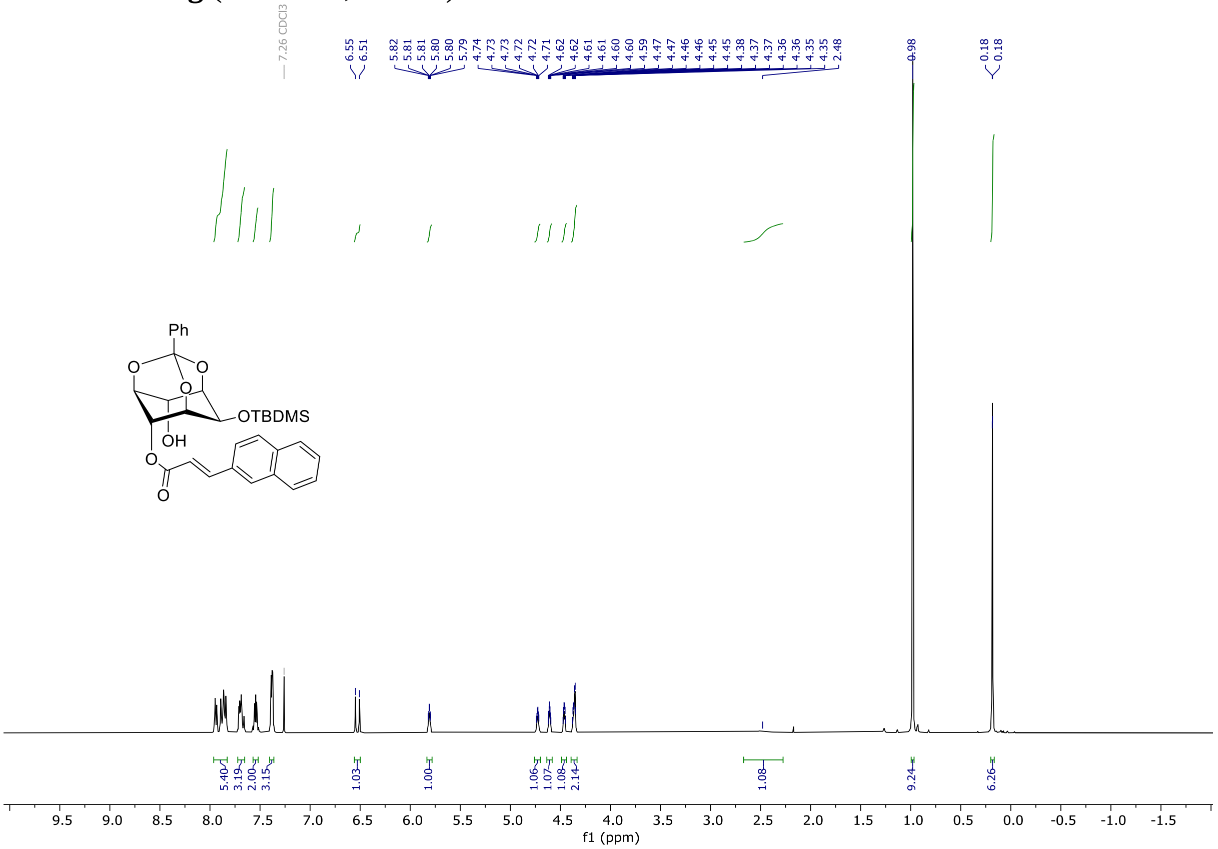

^{13}C{^{1}H} NMR for **3g** (101 MHz, $CDCl_3$)

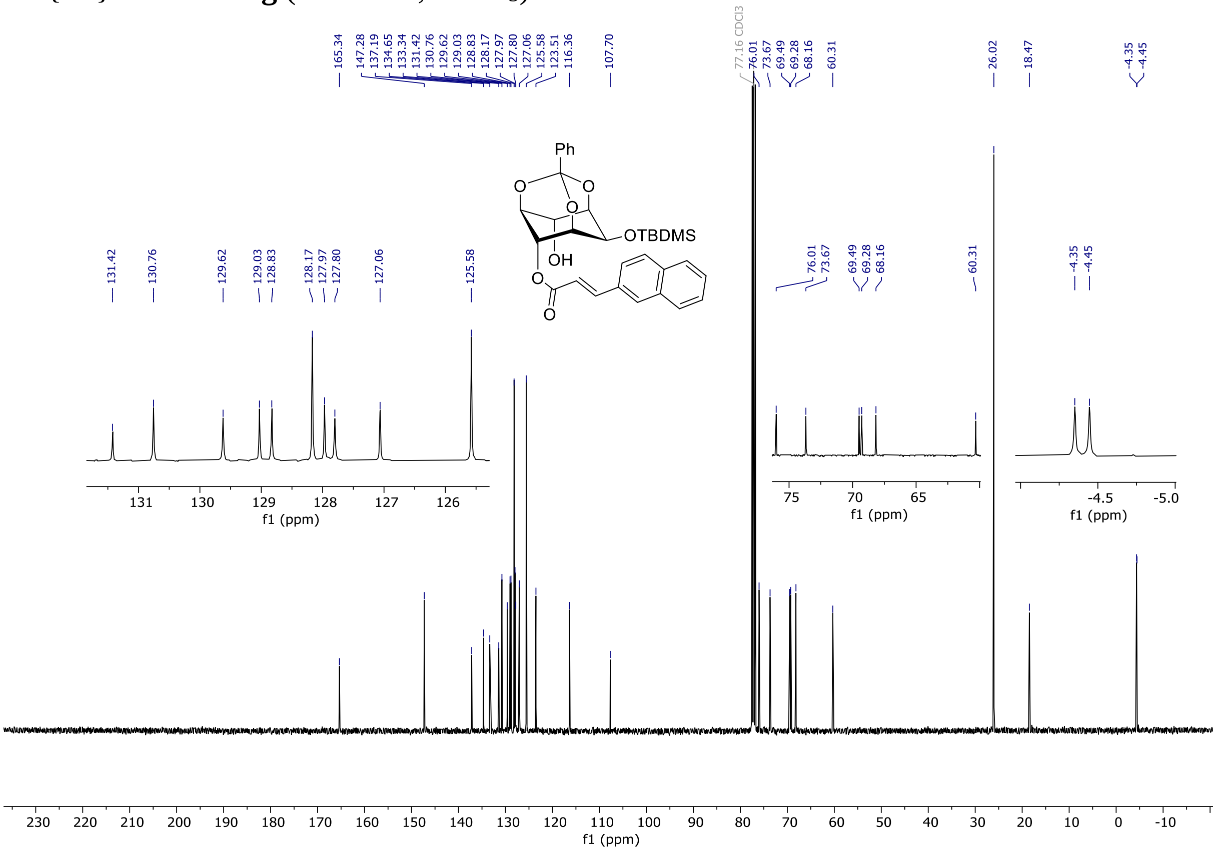

(1*R*,3*R*,5*S*,6*R*,7*S*,8*R*,9*S*)-8-((*tert*-Butyldimethylsilyl)oxy)-9-hydroxy-3-phenyl-2,4,10-trioxaadamantan-6-yl (*E*)-3-(*p*-tolyl)acrylate (3h)

^{1}H NMR for **3h** (400 MHz, $CDCl_3$)

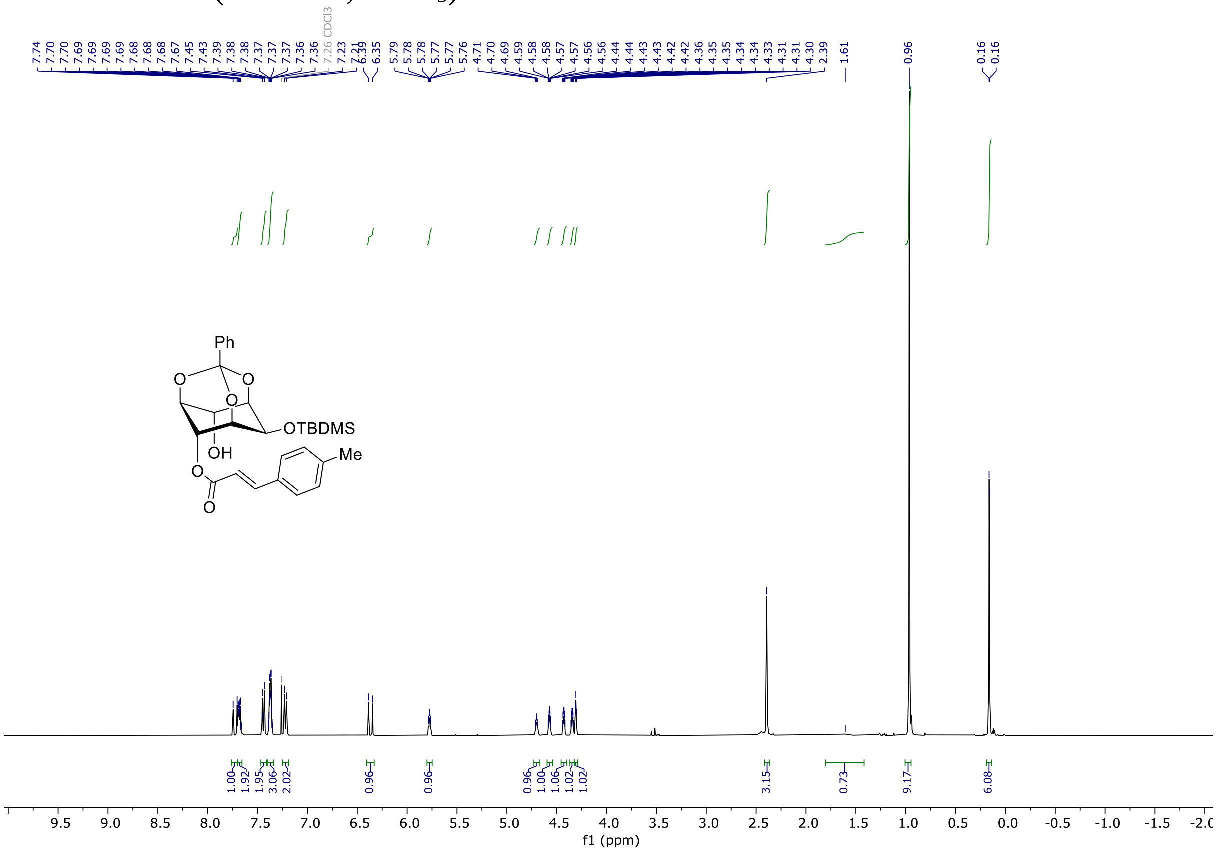

^{13}C{^{1}H} NMR for **3h** (101 MHz, $CDCl_3$)

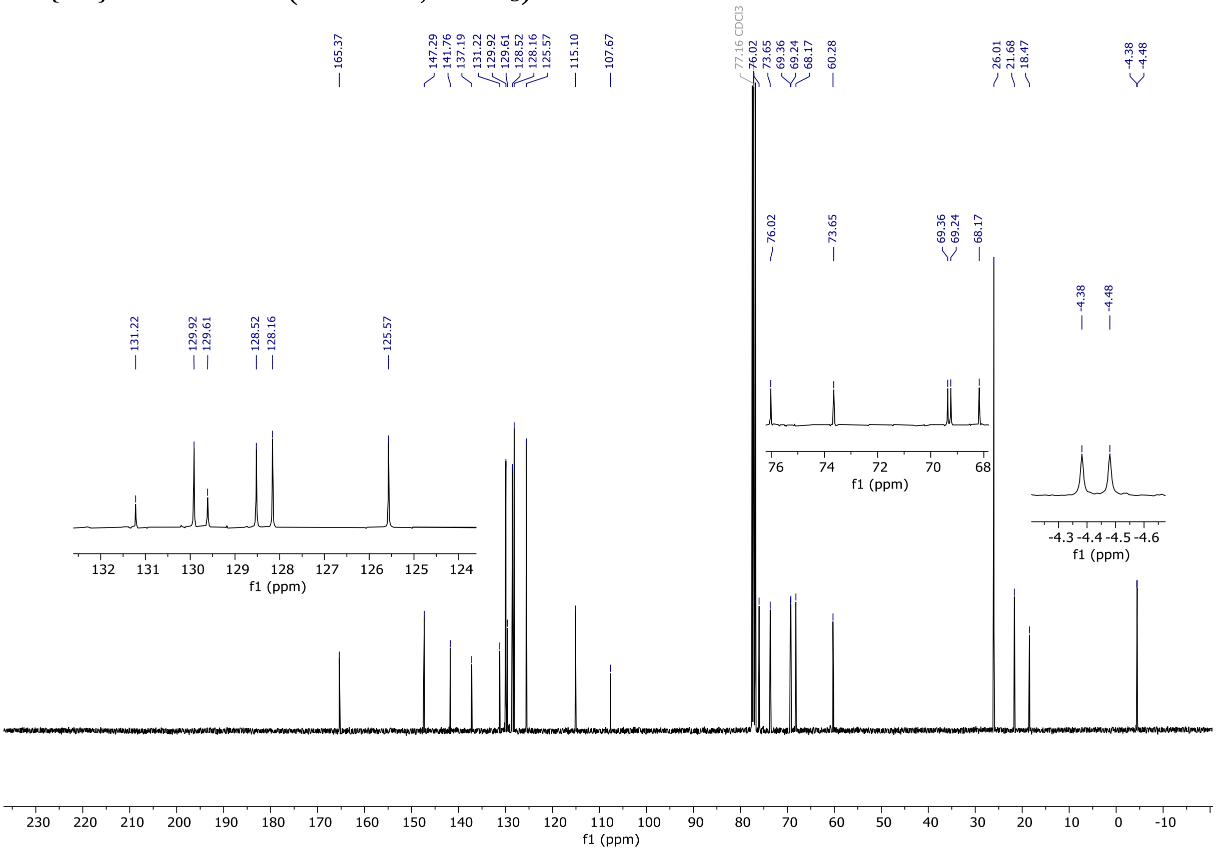

(1*R*,3*R*,5*S*,6*R*,7*S*,8*R*,9*S*)-8-((*tert*-Butyldimethylsilyl)oxy)-9-hydroxy-3-phenyl-2,4,10-trioxaadamantan-6-yl (*E*)-3-(4-methoxyphenyl)acrylate (3j)

^{1}H NMR for **3j** (400 MHz, $CDCl_3$)

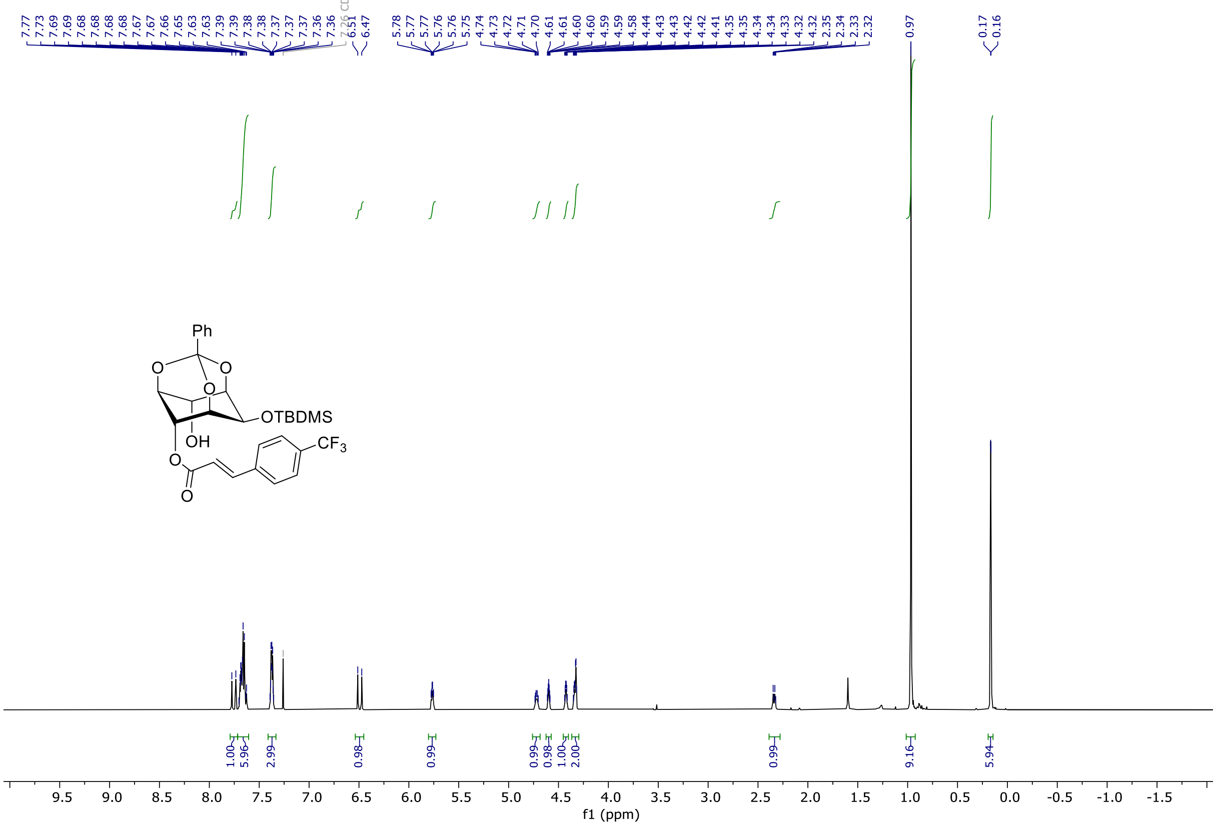

^{13}C{^{1}H} NMR for **3j** (101 MHz, $CDCl_3$)

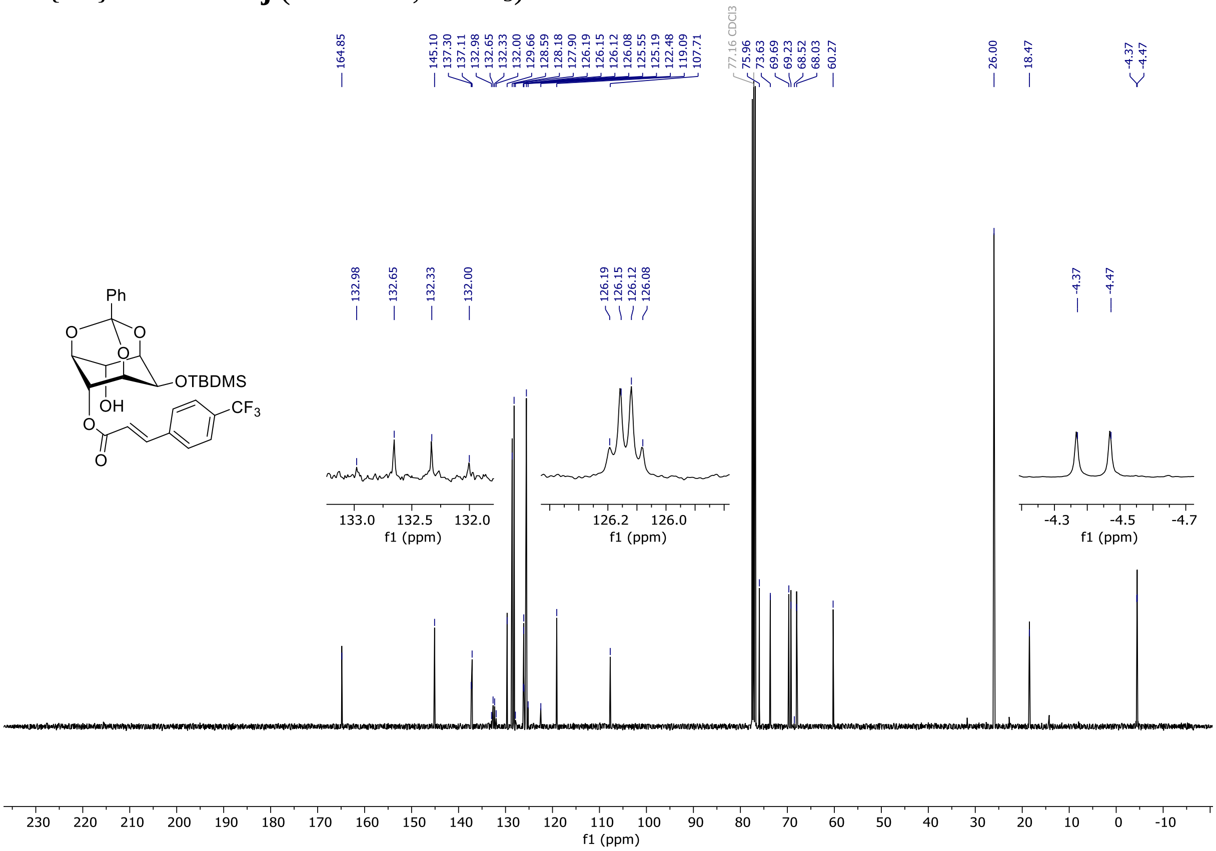

^{19}F NMR for **3j** (376 MHz, $CDCl_3$)

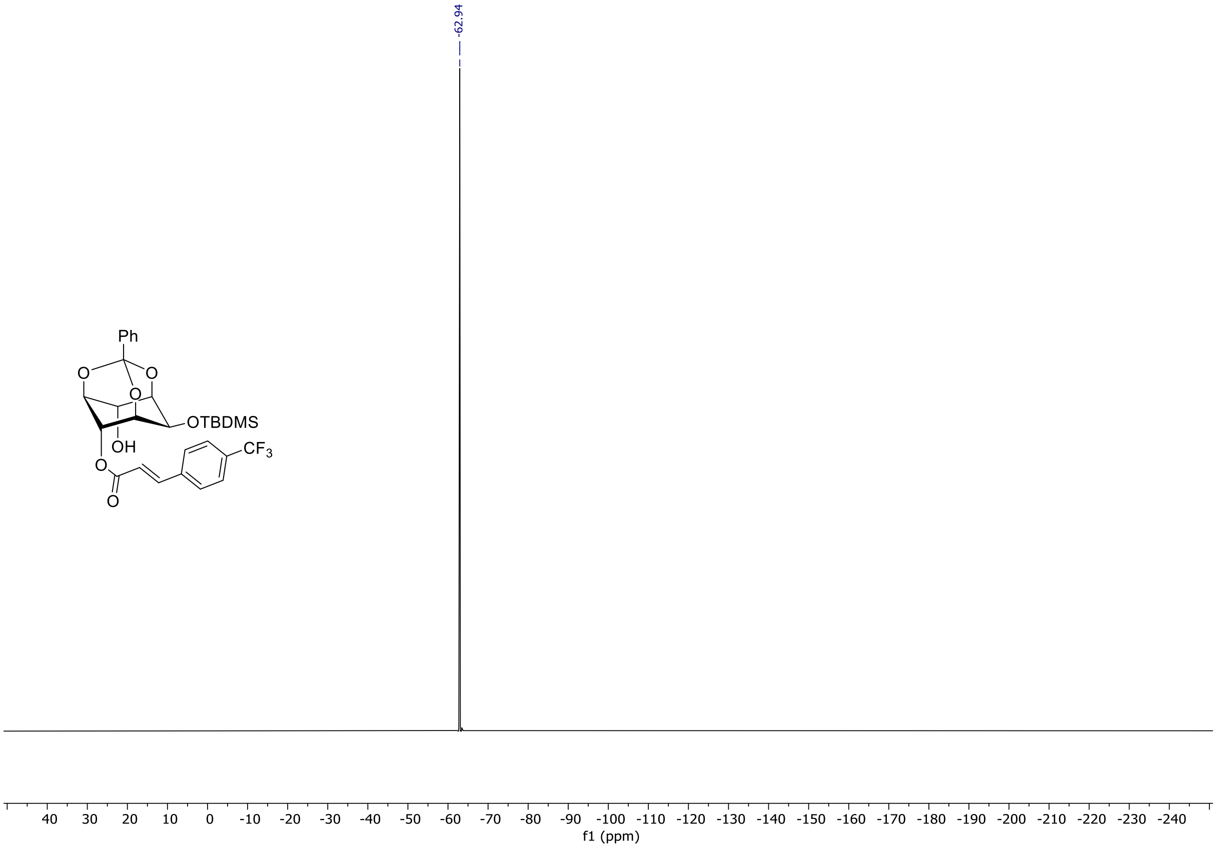

(1*R*,3*R*,5*S*,6*R*,7*S*,8*R*,9*S*)-8-((*tert*-Butyldimethylsilyl)oxy)-9-hydroxy-3-phenyl-2,4,10-trioxaadamantan-6-yl (*E*)-3-(4-fluorophenyl)acrylate (3l)

^{1}H NMR for **3l** (400 MHz, $CDCl_3$)

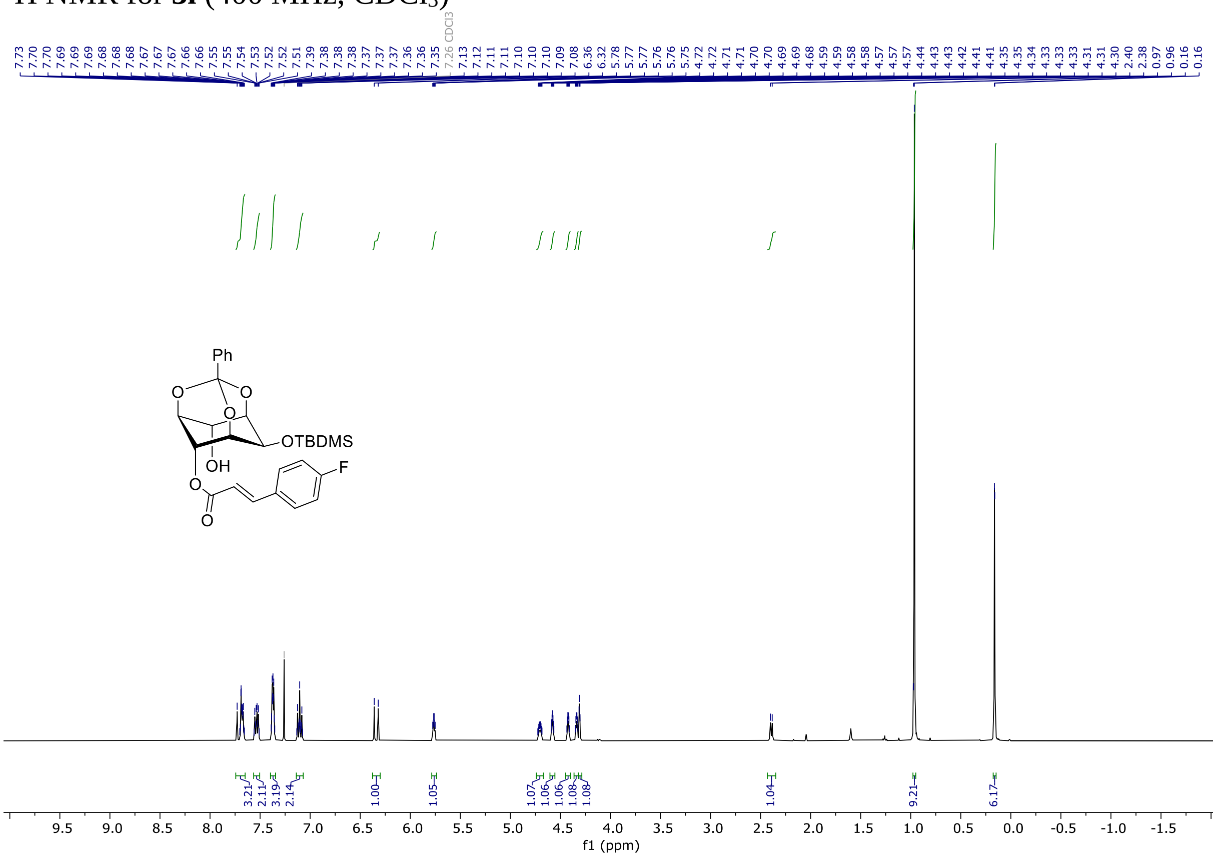

$^{13}C\{^1H\}$ NMR for **3l** (101 MHz, $CDCl_3$)

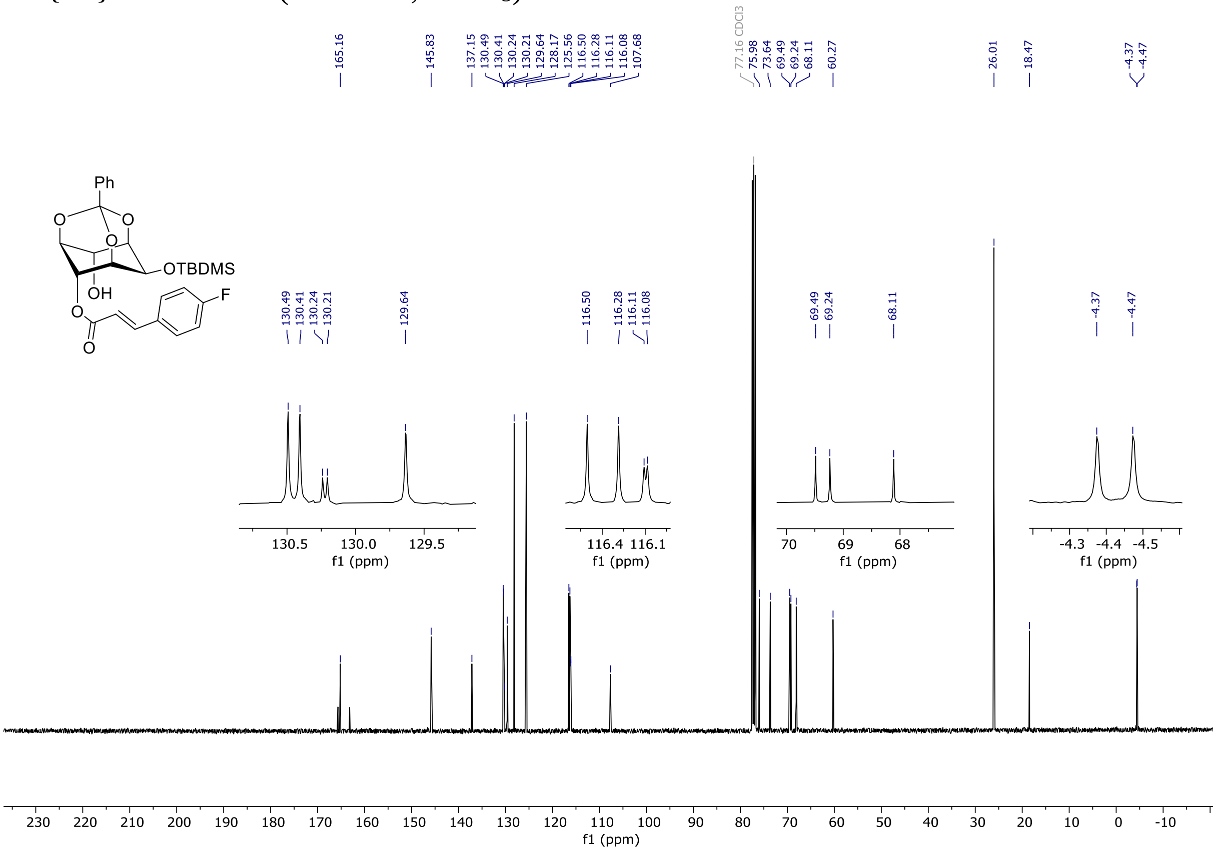

^{19}F NMR for **3l** (376 MHz, $CDCl_3$)

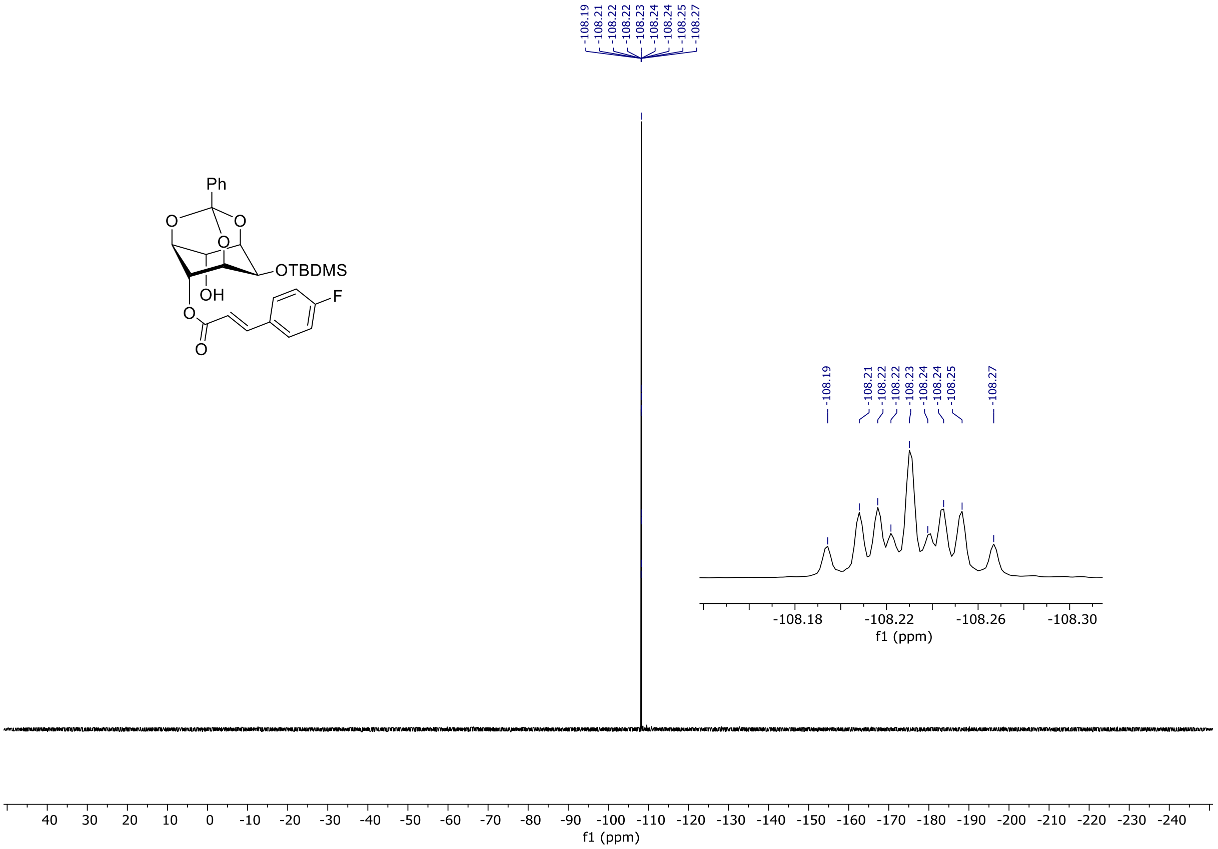

(1*R*,3*R*,5*S*,6*R*,7*S*,8*R*,9*S*)-8-((*tert*-Butyldimethylsilyl)oxy)-9-hydroxy-3-phenyl-2,4,10-trioxaadamantan-6-yl (*E*)-3-(4-chlorophenyl)acrylate (3m)

^{1}H NMR for **3m** (400 MHz, $CDCl_3$)

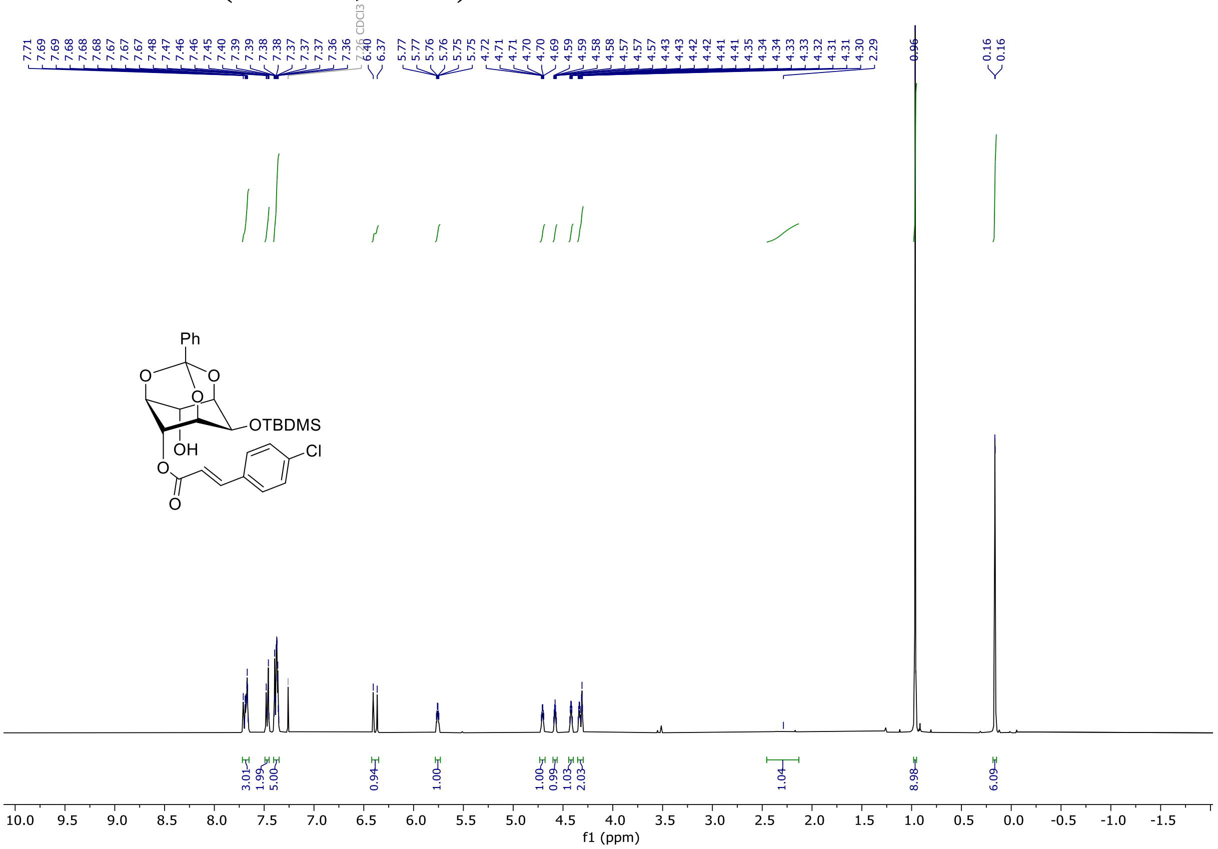

^{13}C{^{1}H} NMR for **3m** (101 MHz, $CDCl_3$)

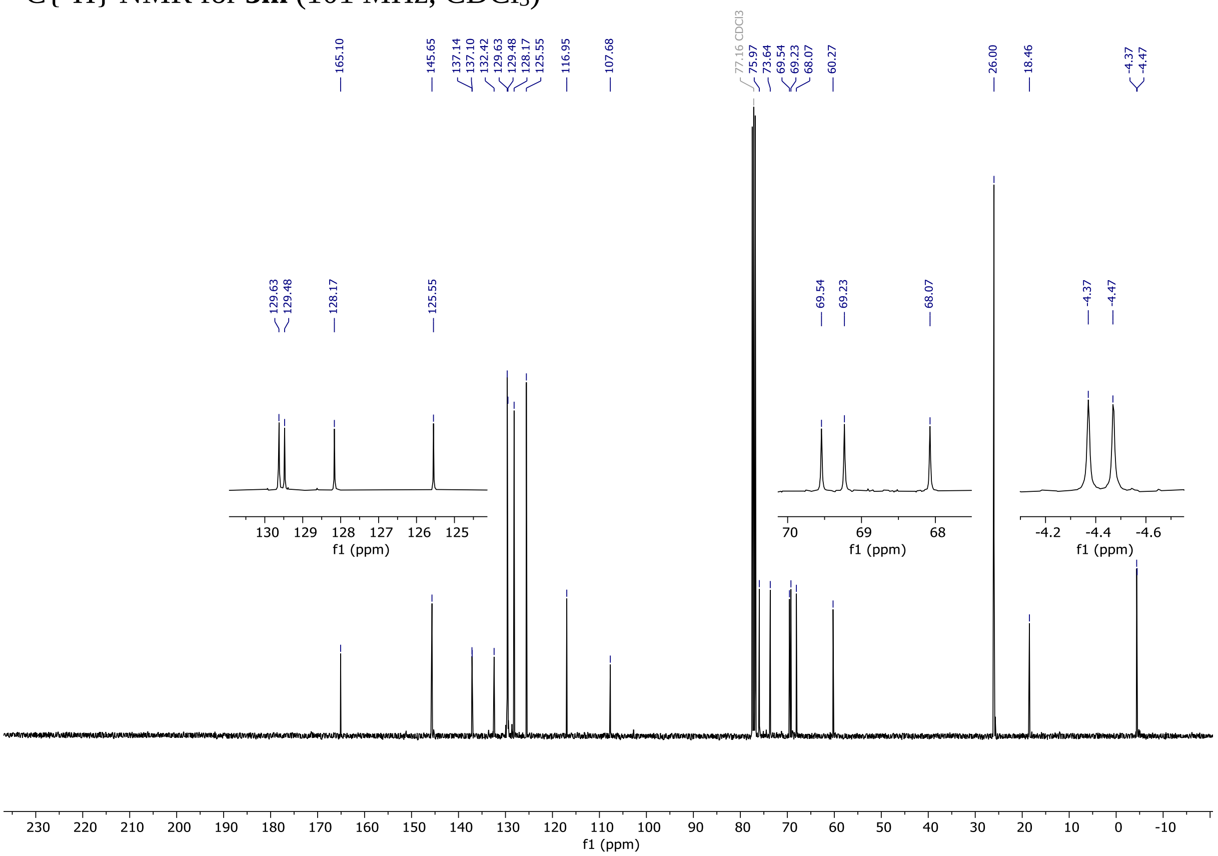

(1*R*,3*R*,5*S*,6*R*,7*S*,8*R*,9*S*)-8-((*tert*-Butyldimethylsilyl)oxy)-9-hydroxy-3-phenyl-2,4,10-trioxaadamantan-6-yl (*E*)-3-(4-bromophenyl)acrylate (3n)

^{1}H NMR for **3n** (400 MHz, $CDCl_3$)

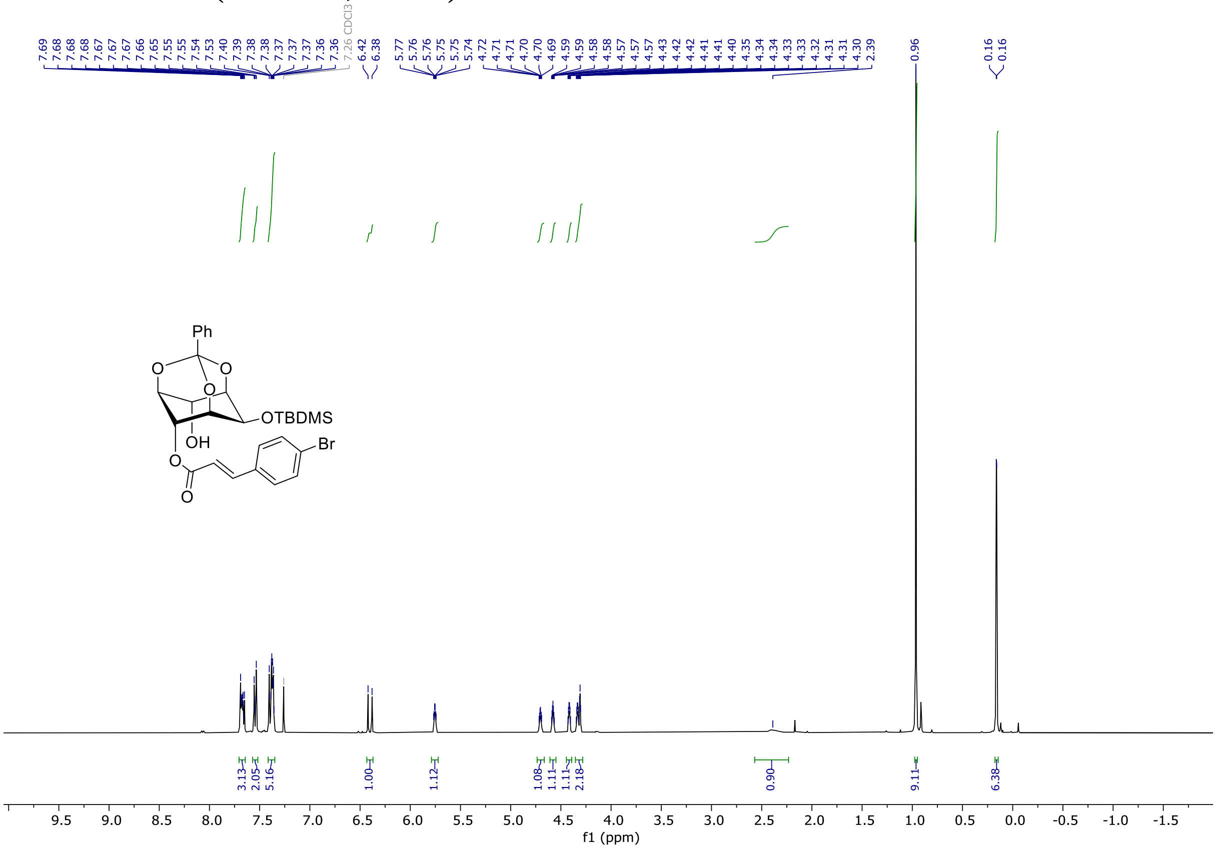

^{13}C{^{1}H} NMR for **3n** (101 MHz, $CDCl_3$)

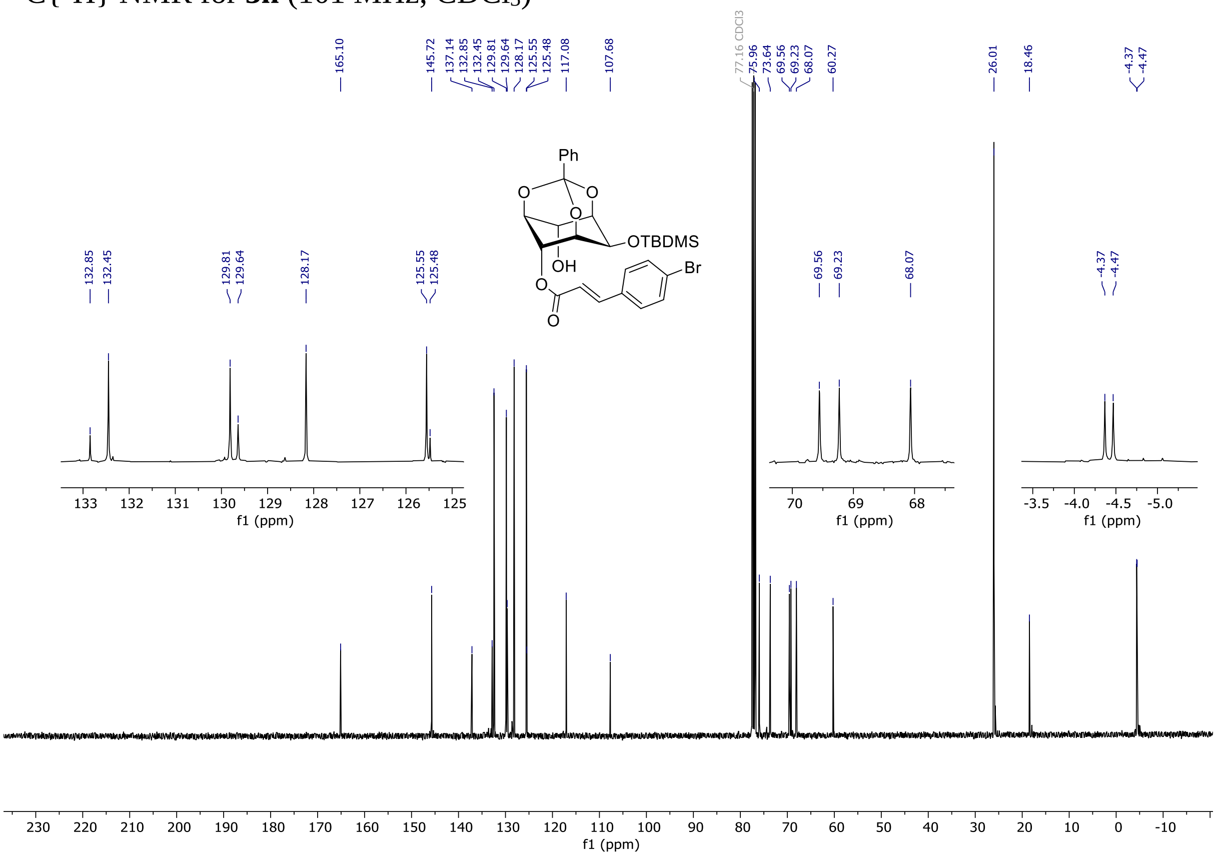

(1*R*,3*R*,5*S*,6*R*,7*S*,8*R*,9*S*)-8-((*tert*-Butyldimethylsilyl)oxy)-9-hydroxy-3-phenyl-2,4,10-trioxaadamantan-6-yl (*E*)-3-(3-chlorophenyl)acrylate (3o)

^{1}H NMR for **3o** (400 MHz, $CDCl_3$)

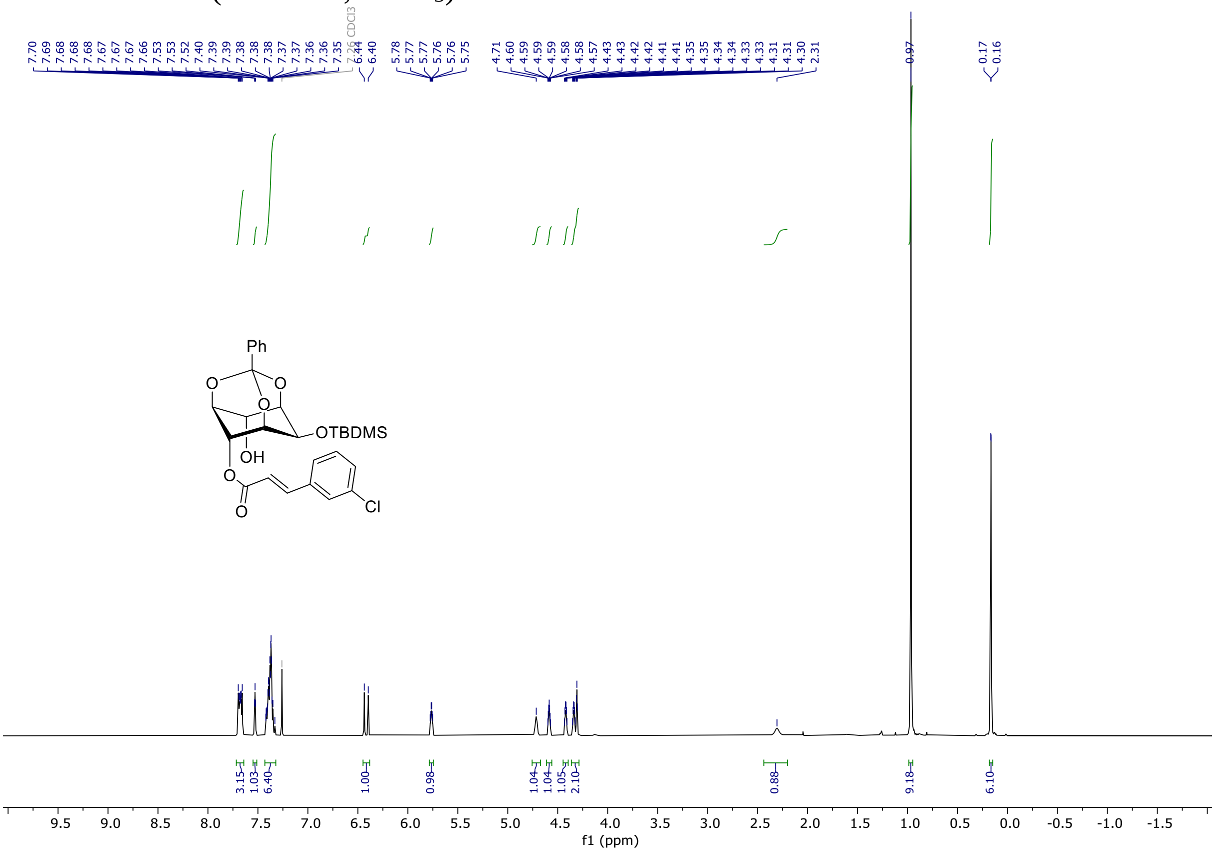

^{13}C{^{1}H} NMR **3o** (101 MHz, $CDCl_3$)

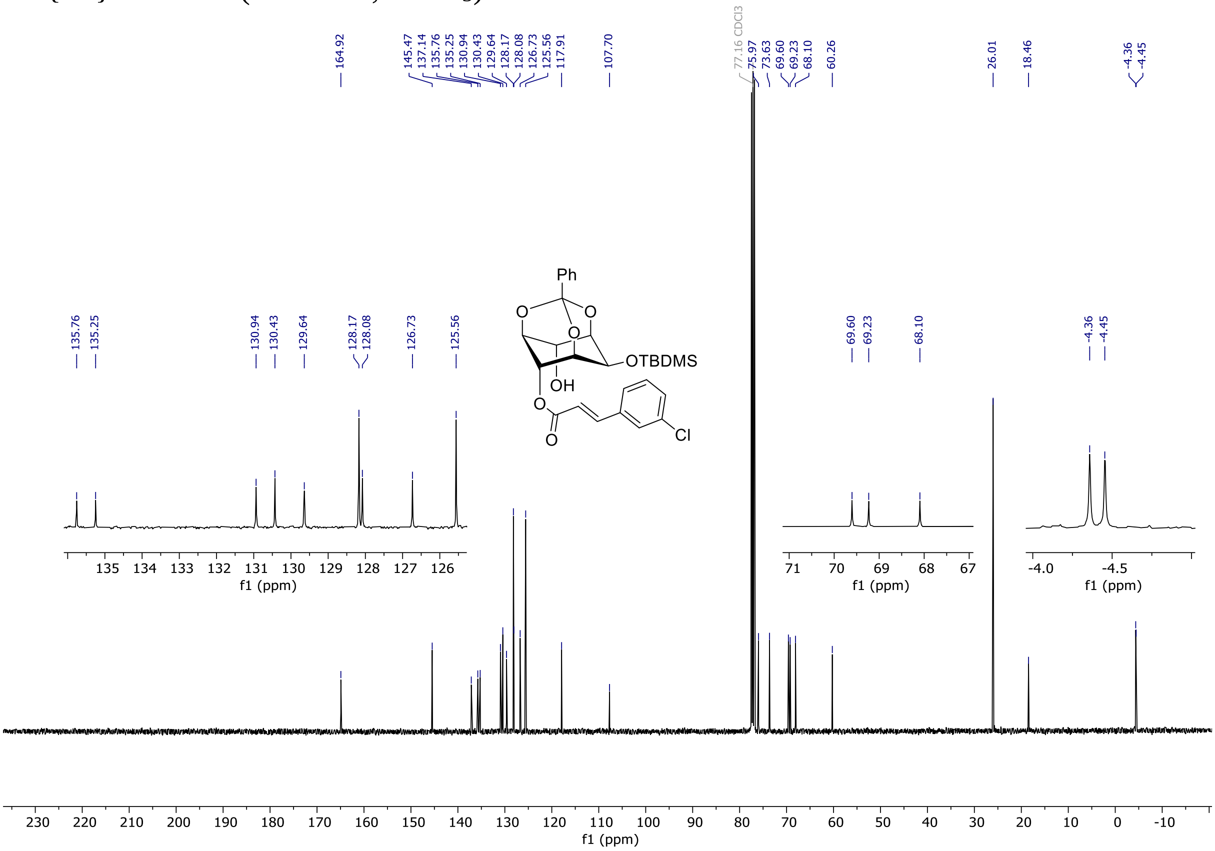

(1*R*,3*R*,5*S*,6*R*,7*S*,8*R*,9*S*)-8-((*tert*-Butyldimethylsilyl)oxy)-9-hydroxy-3-phenyl-2,4,10-trioxaadamantan-6-yl (*E*)-3-(2-chlorophenyl)acrylate (3p)

^{1}H NMR for **3p** (400 MHz, $CDCl_3$)

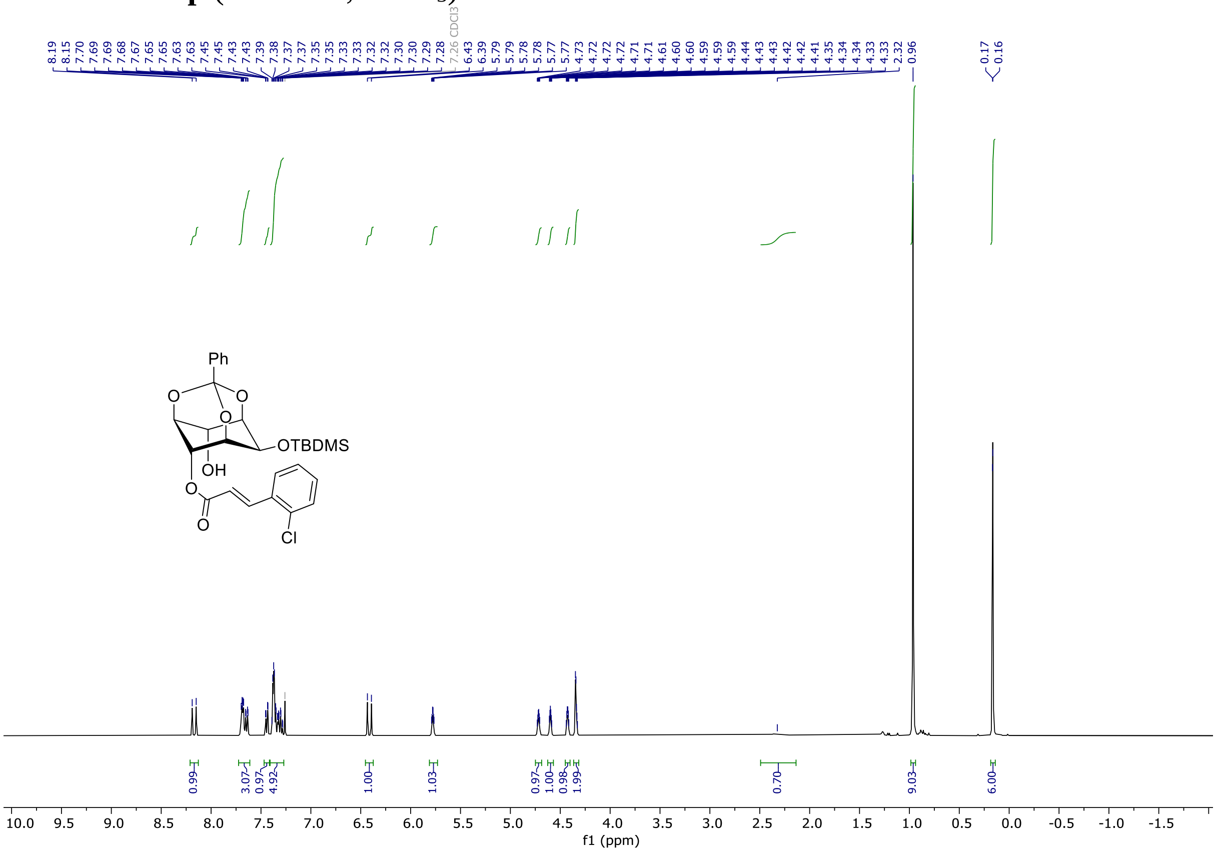

^{13}C{^{1}H} NMR for **3p** (101 MHz, $CDCl_3$)

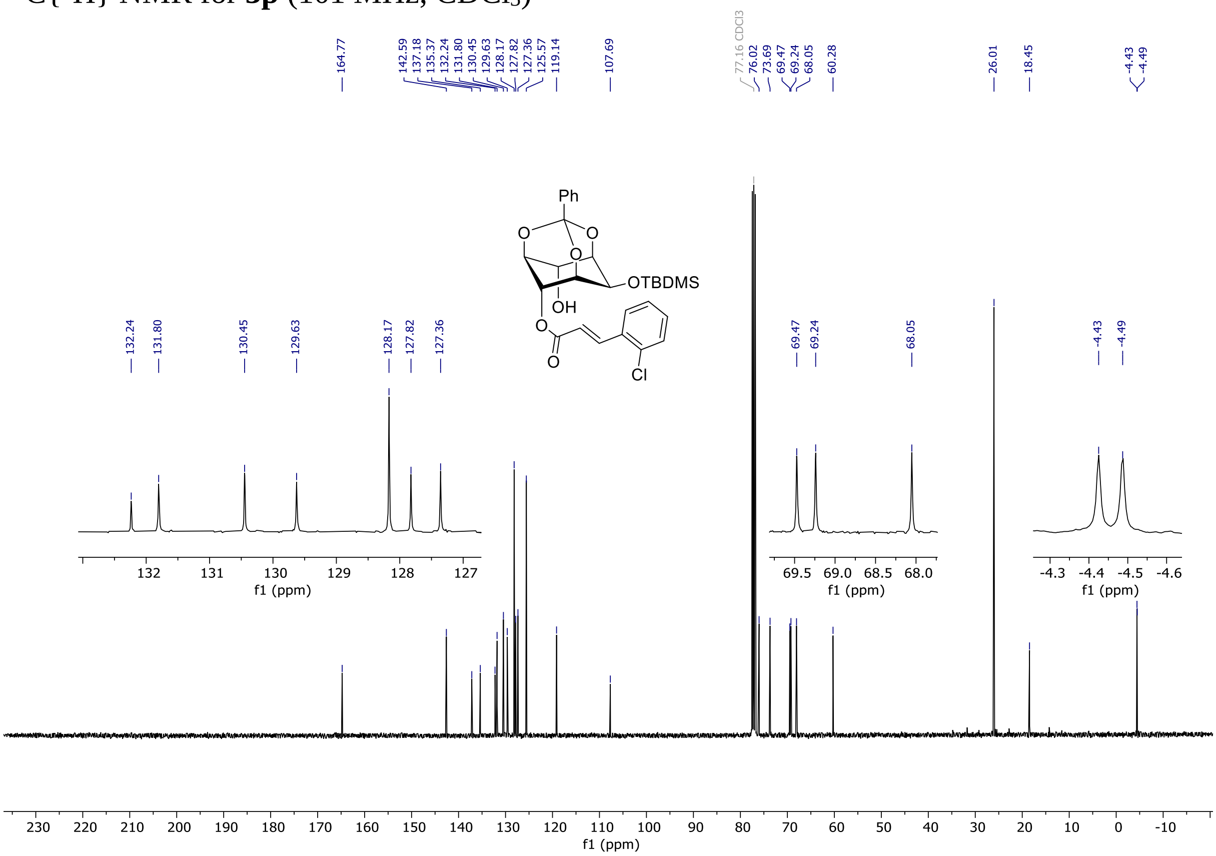

(1*R*,3*R*,5*S*,6*R*,7*S*,8*R*,9*S*)-8-((*tert*-Butyldimethylsilyl)oxy)-9-hydroxy-3-phenyl-2,4,10-trioxaadamantan-6-yl (*E*)-3-(2-chlorophenyl)acrylate (3q)

^{1}H NMR for **3q** (400 MHz, $CDCl_3$)

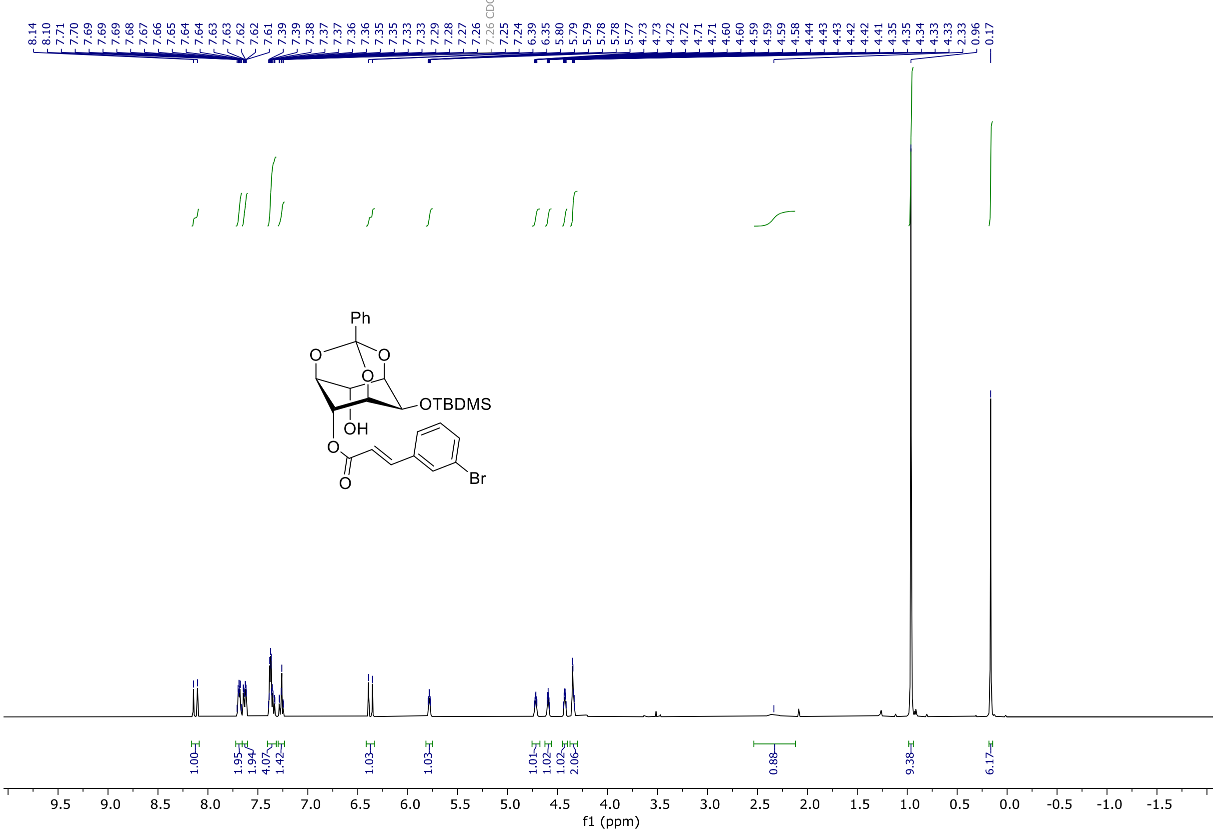

^{13}C{^{1}H} NMR for **3q** (101 MHz, $CDCl_3$)

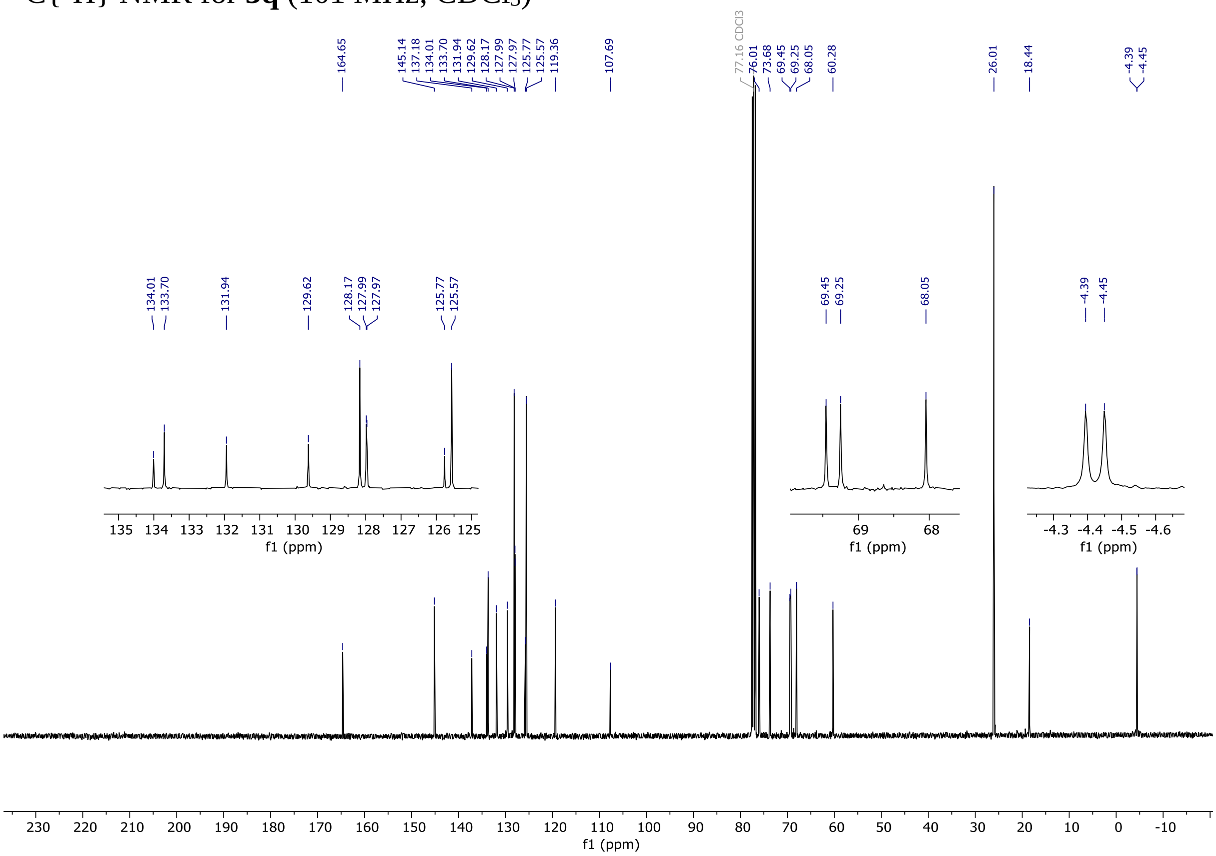

(1*R*,3*R*,5*S*,6*R*,7*S*,8*R*,9*S*)-8-((*tert*-Butyldimethylsilyl)oxy)-9-hydroxy-3-phenyl-2,4,10-trioxaadamantan-6-yl (*E*)-3-(2-bromophenyl)acrylate (3r)

^{1}H NMR for **3r** (400 MHz, $CDCl_3$)

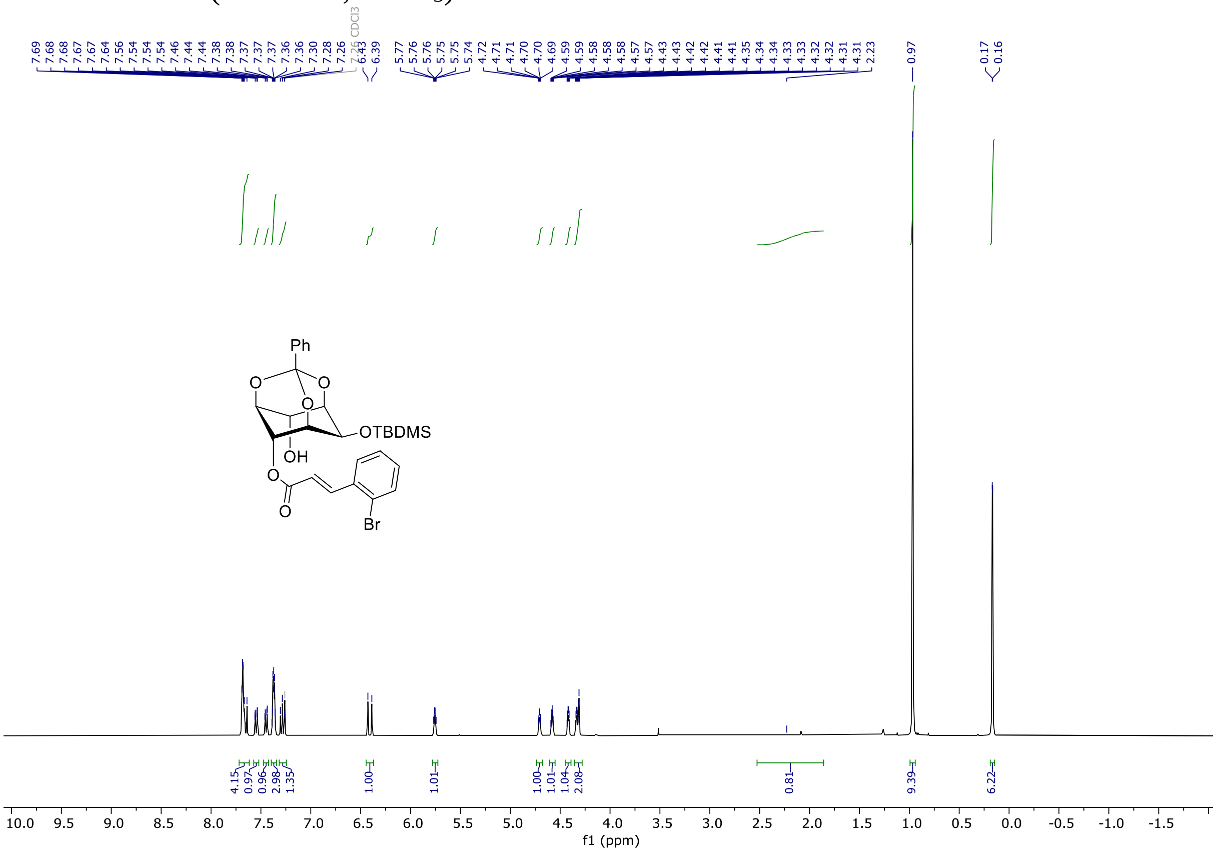

^{13}C{^{1}H} NMR for **3r** (101 MHz, $CDCl_3$)

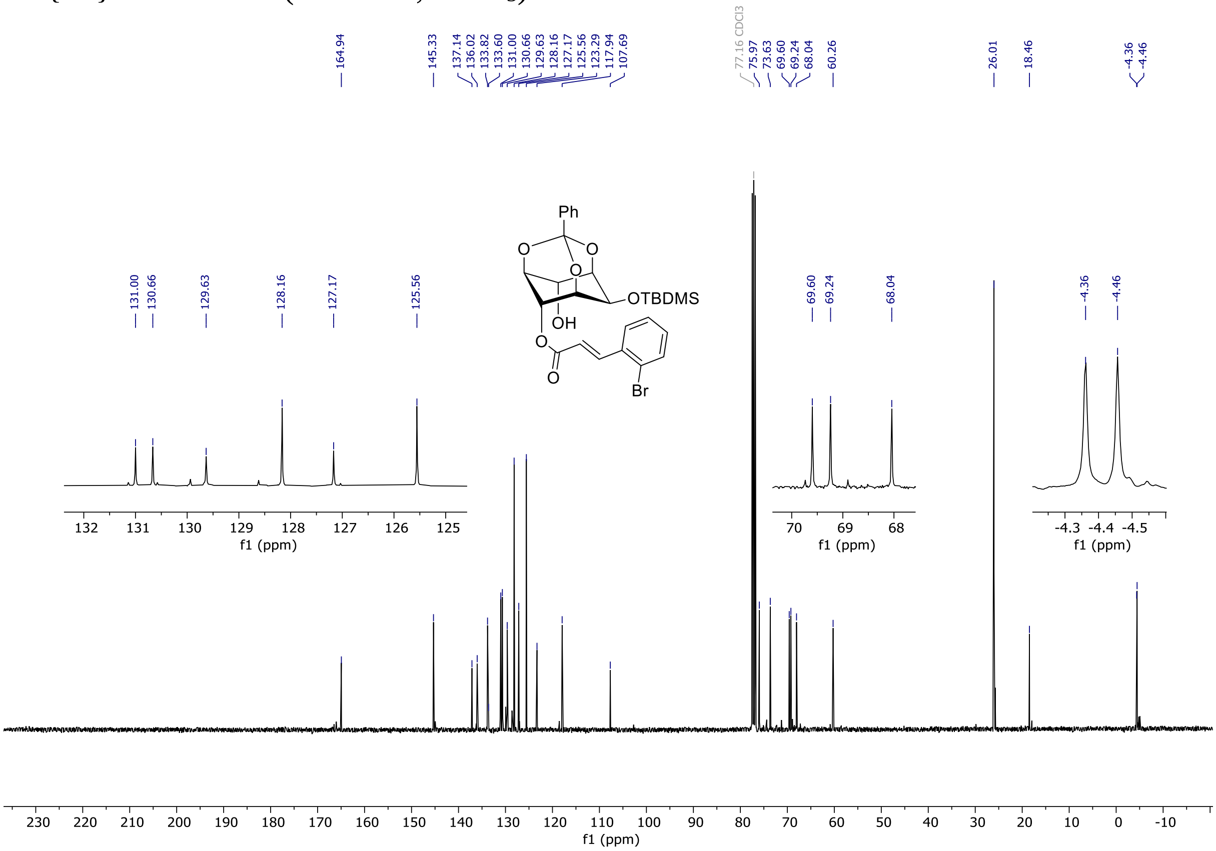

(1*R*,3*R*,5*S*,6*R*,7*S*,8*R*,9*S*)-8-((*tert*-Butyldimethylsilyl)oxy)-9-hydroxy-3-phenyl-2,4,10-trioxaadamantan-6-yl (*E*)-3-(furan-2-yl)acrylate (3s)

^{1}H NMR for **3s** (400 MHz, $CDCl_3$)

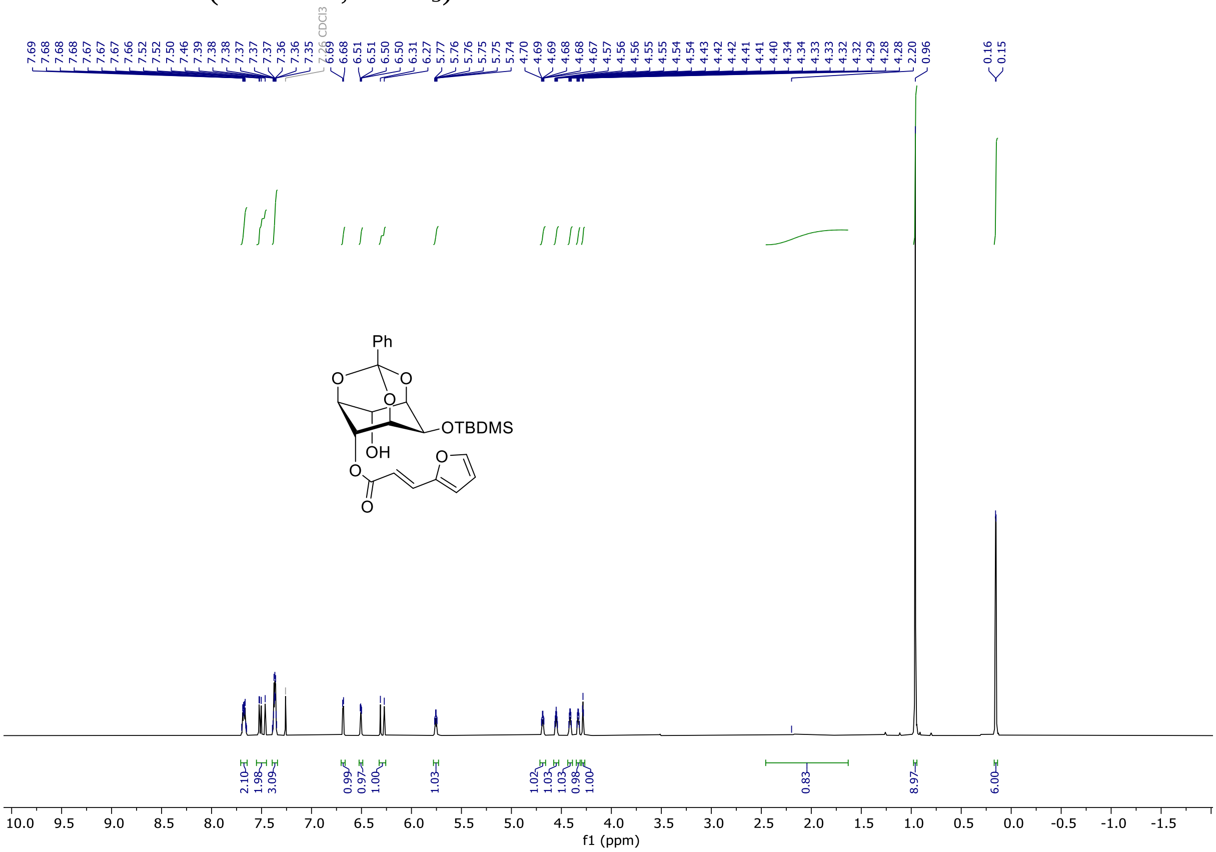

$^{13}C\{^{1}H\}$ NMR for **3s** (101 MHz, $CDCl_3$)

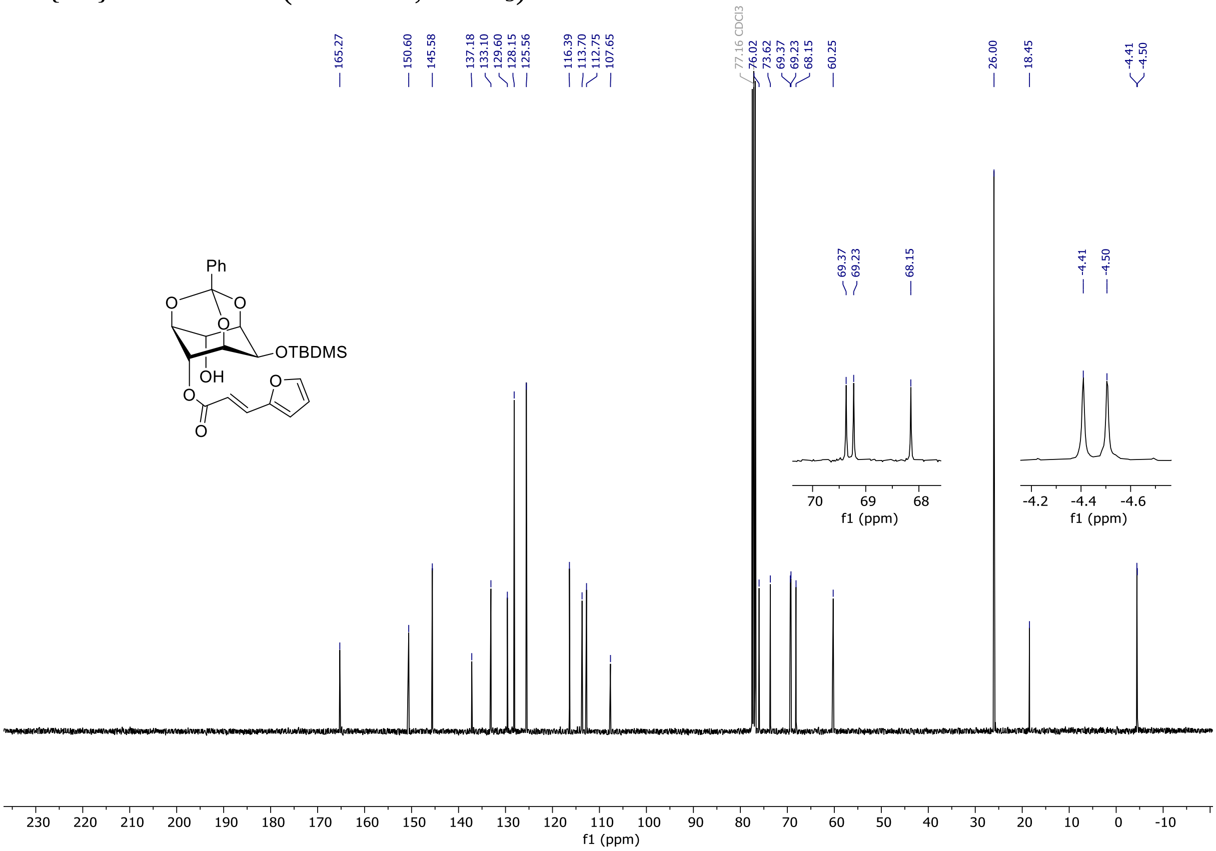

(1*R*,3*R*,5*S*,6*R*,7*S*,8*R*,9*S*)-8-((*tert*-Butyldimethylsilyl)oxy)-9-hydroxy-3-phenyl-2,4,10-trioxaadamantan-6-yl (*E*)-3-(thiophen-2-yl)acrylate (3t)

^{1}H NMR for **3t** (400 MHz, $CDCl_3$)

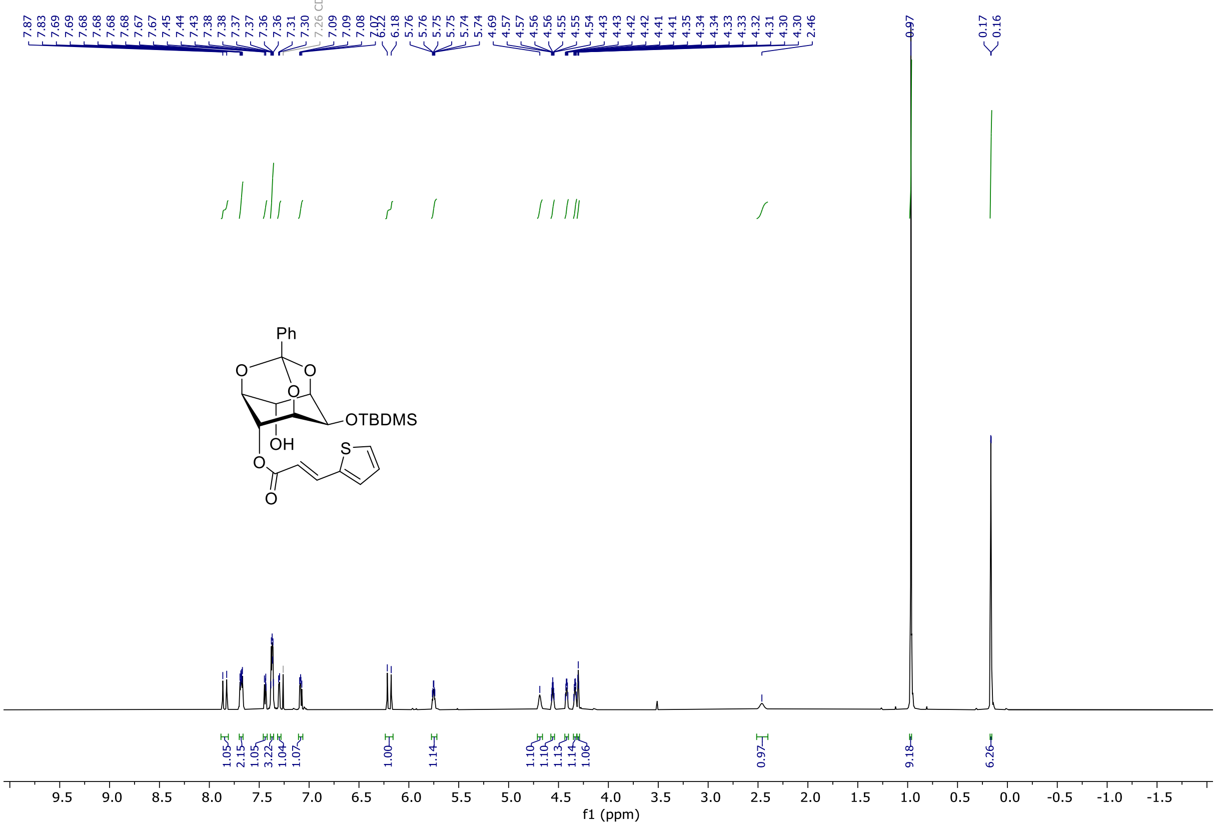

^{13}C{^{1}H} NMR for **3t** (101 MHz, $CDCl_3$)

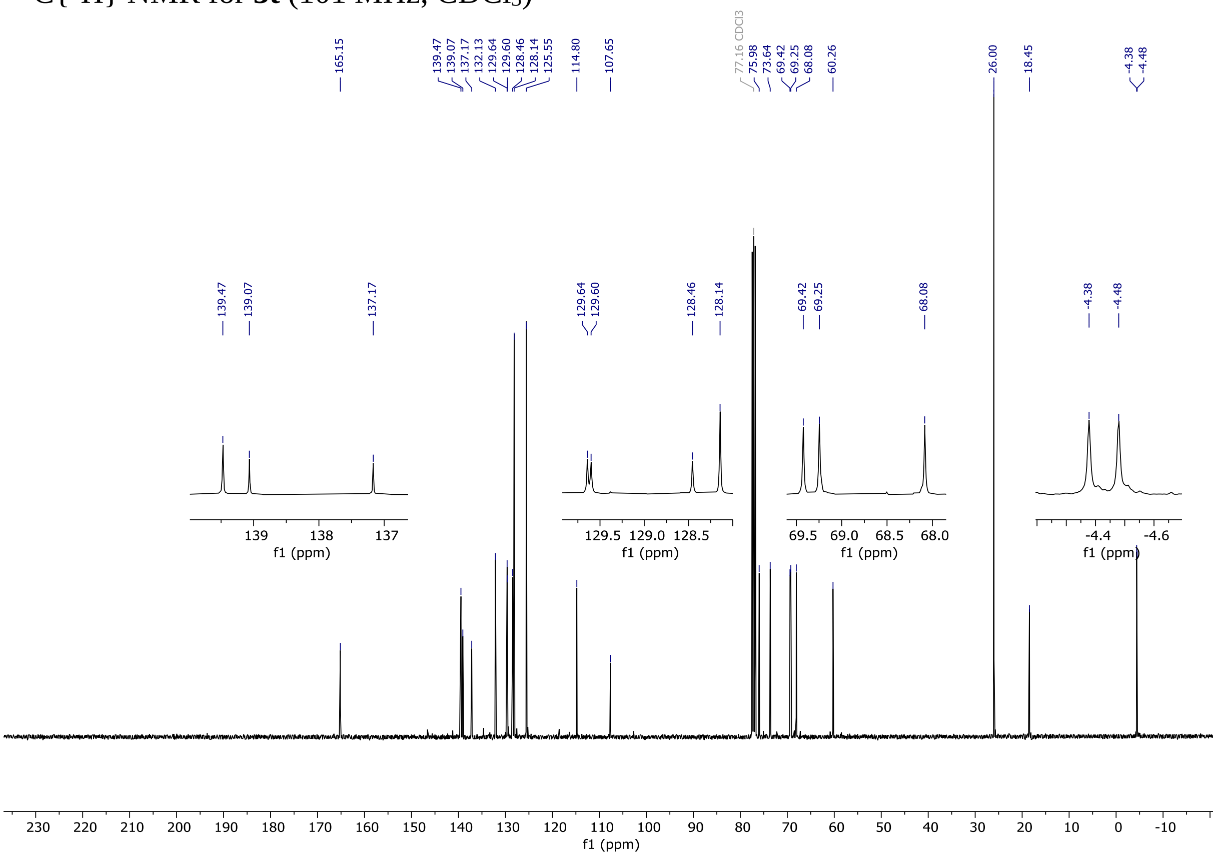

(1*R*,3*R*,5*S*,6*R*,7*S*,8*R*,9*S*)-8-((*tert*-Butyldimethylsilyl)oxy)-9-hydroxy-3-phenyl-2,4,10-trioxaadamantan-6-yl ethyl fumarate (3u)

^{1}H NMR of **3u** (400 MHz, $CDCl_3$)

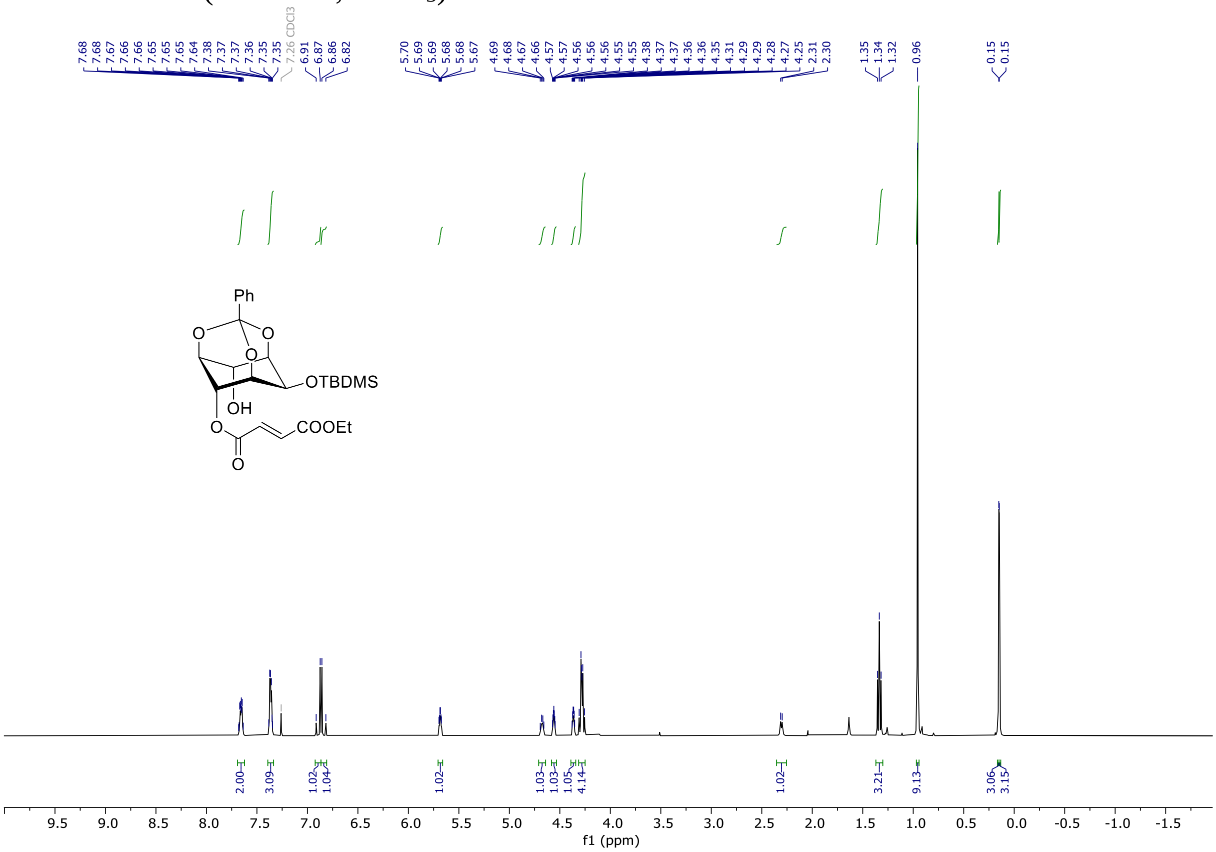

^{13}C NMR of **3u** (101 MHz, $CDCl_3$)

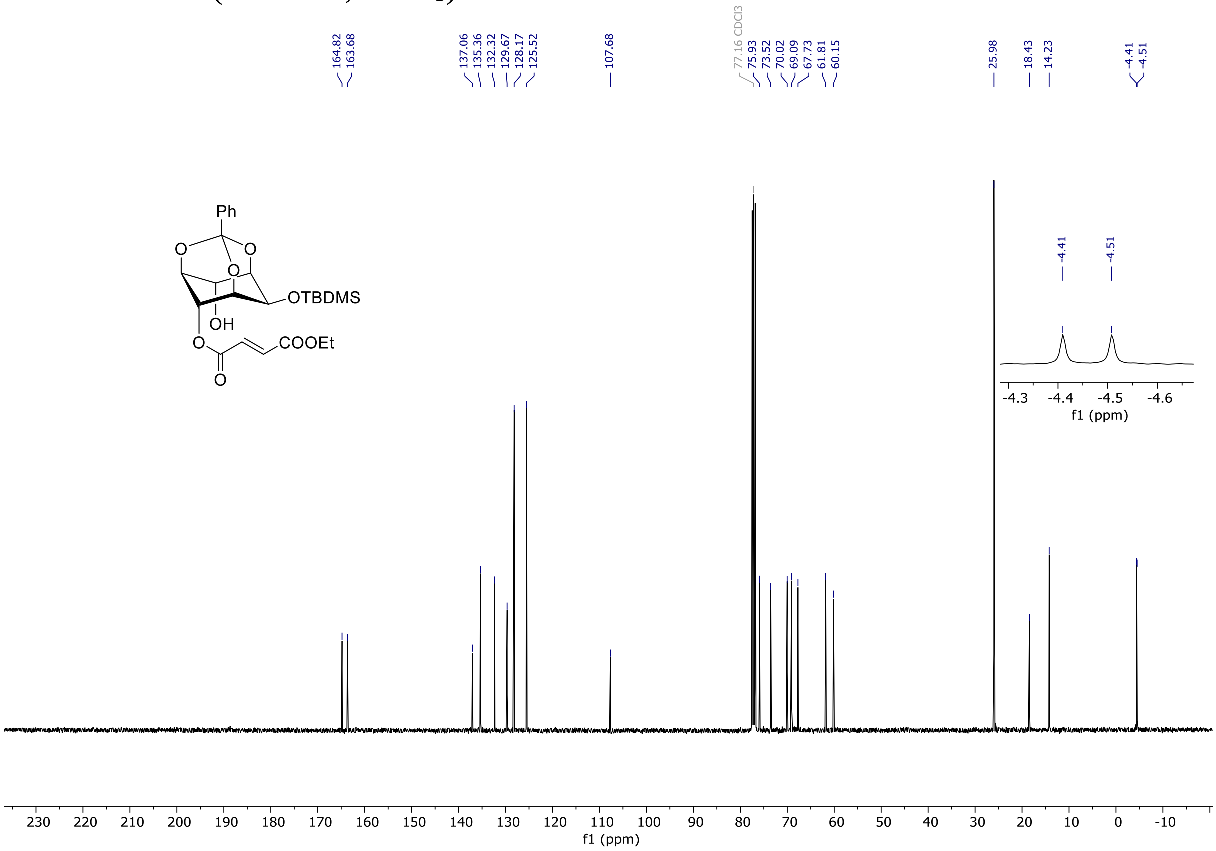

(1*R*,3*R*,5*S*,6*R*,7*S*,8*S*,9*R*)-8-acetoxy-9-((*tert*-Butyldimethylsilyl)oxy)-3-phenyl-2,4,10-trioxaadamantan-6-yl (*E*)-3-(4-methoxyphenyl)acrylate (4i)

^{1}H NMR of **4i** (400 MHz, $CDCl_3$)

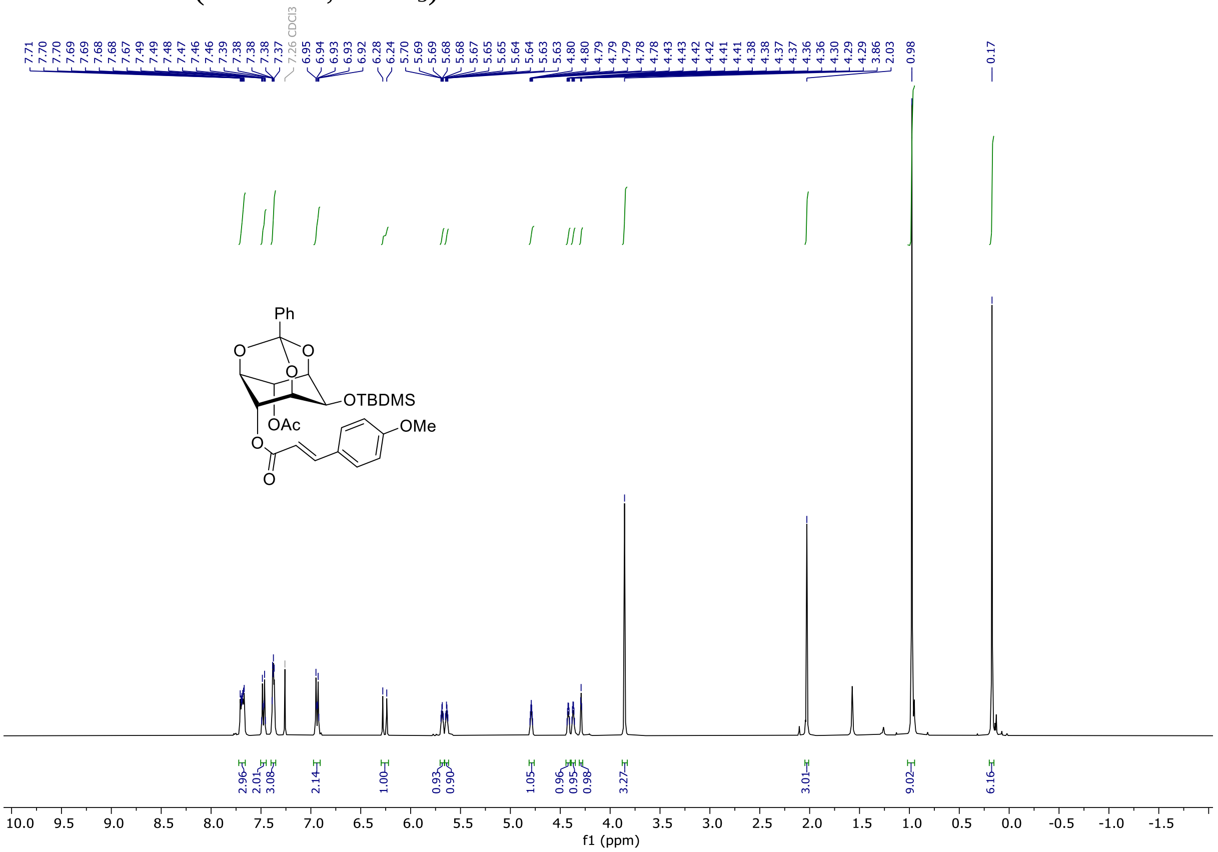

^{13}C NMR of **4i** (101 MHz, $CDCl_3$)

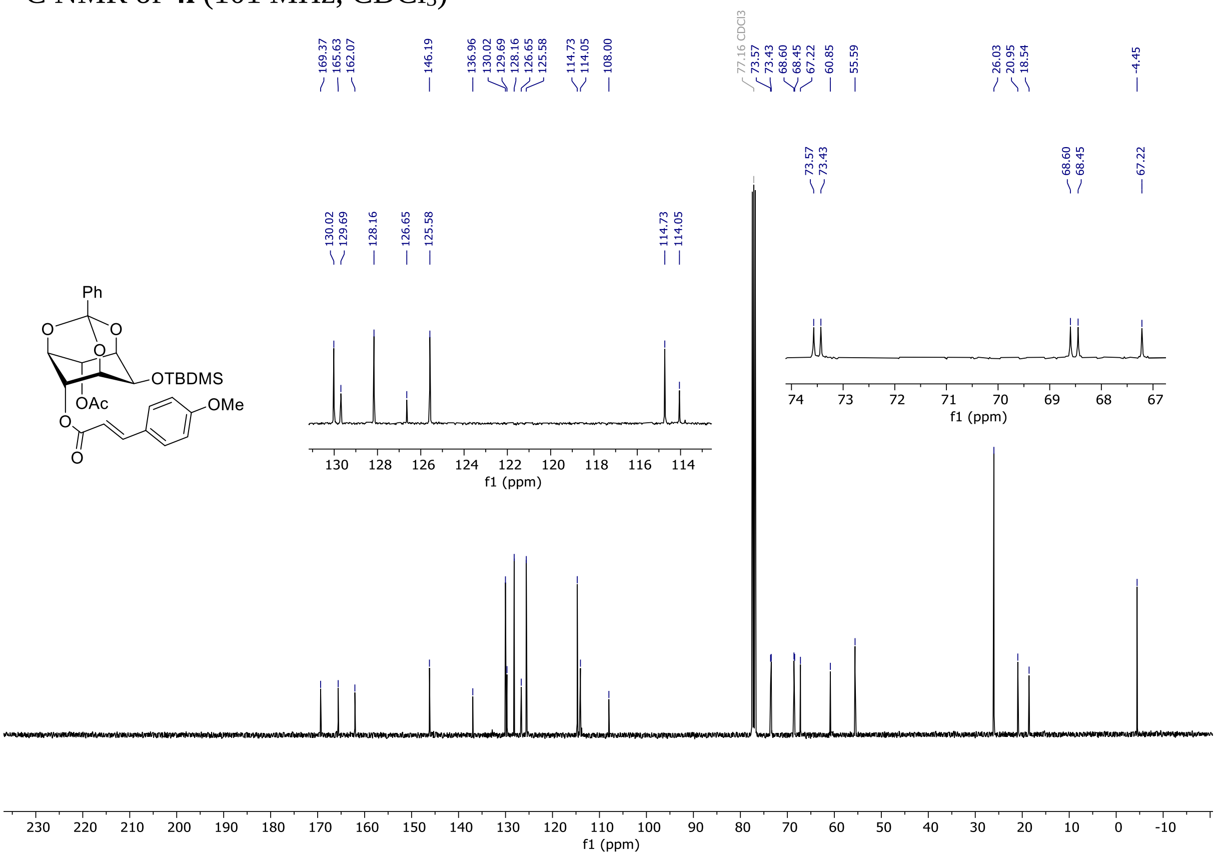

(1*R*,3*R*,5*S*,6*R*,7*S*,8*S*,9*R*)-8-acetoxy-9-((*tert*-Butyldimethylsilyl)oxy)-3-phenyl-2,4,10-trioxaadamantan-6-yl (*E*)-3-(4-nitrophenyl)acrylate (4k)

^{1}H NMR for **4k** (400 MHz, $CDCl_3$)

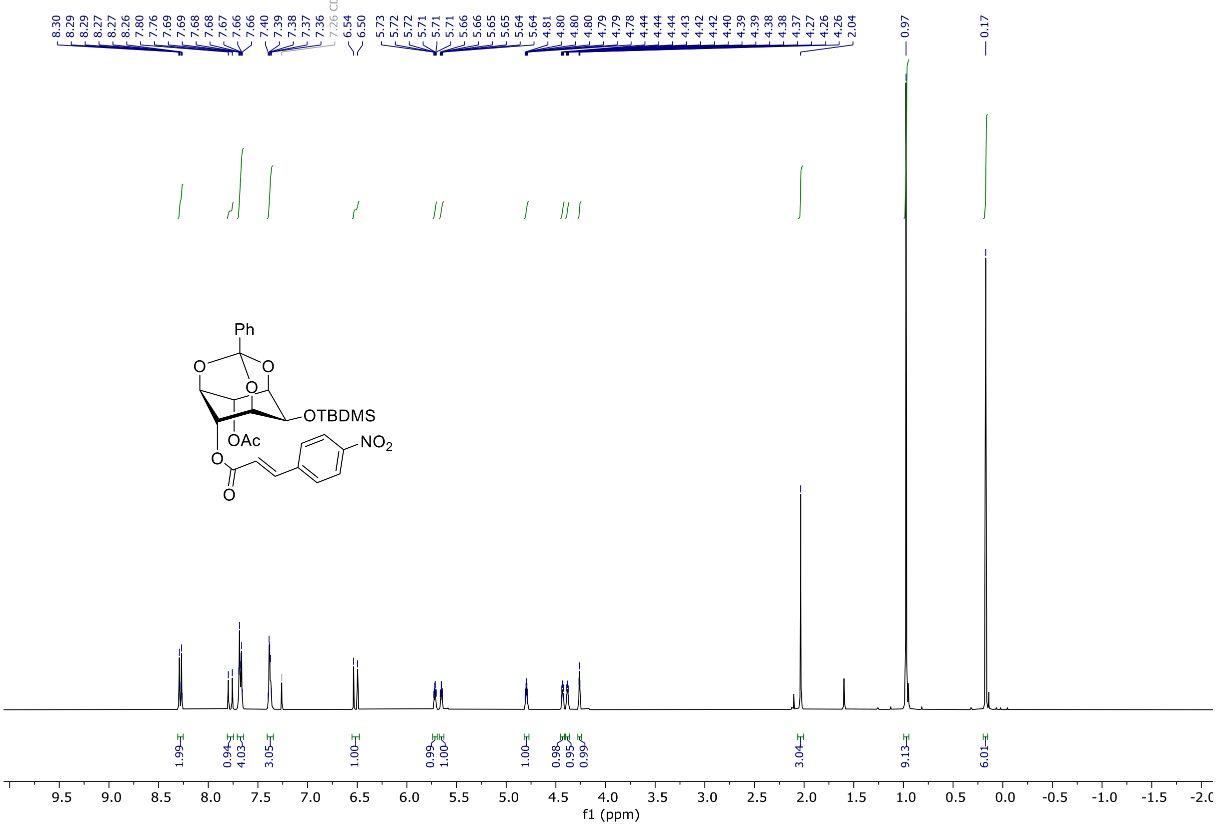

^{13}C{^{1}H} NMR for **4k** (101 MHz, $CDCl_3$)

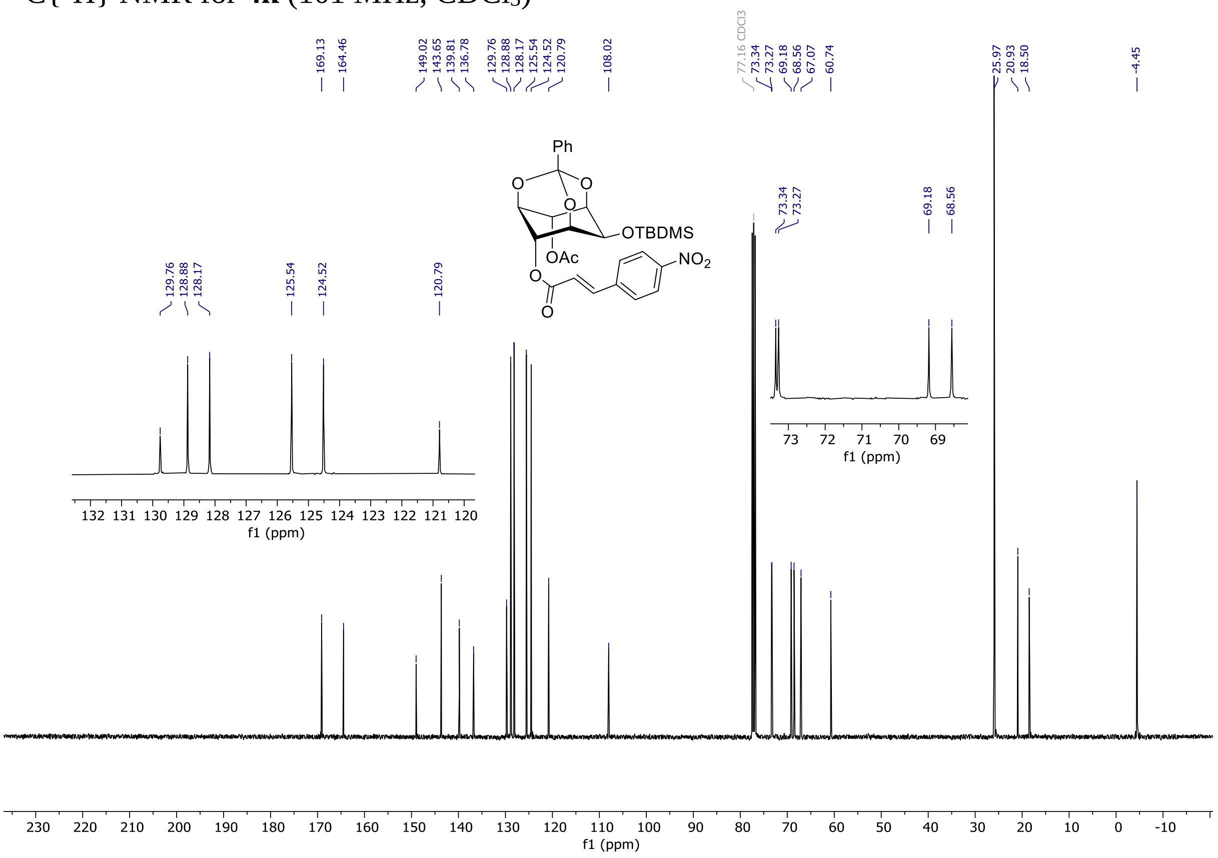

(1*R*,3*R*,5*S*,6*R*,7*S*,8*R*,9*S*)-8-((*tert*-Butyldimethylsilyl)oxy)-9-hydroxy-3-phenyl-2,4,10-trioxaadamantan-6-yl 2-(2-(*tert*-butyl)-6-methylphenoxy)-3-formylbenzoate (5b)

^{1}H NMR for **5b** (400 MHz, $CDCl_3$)

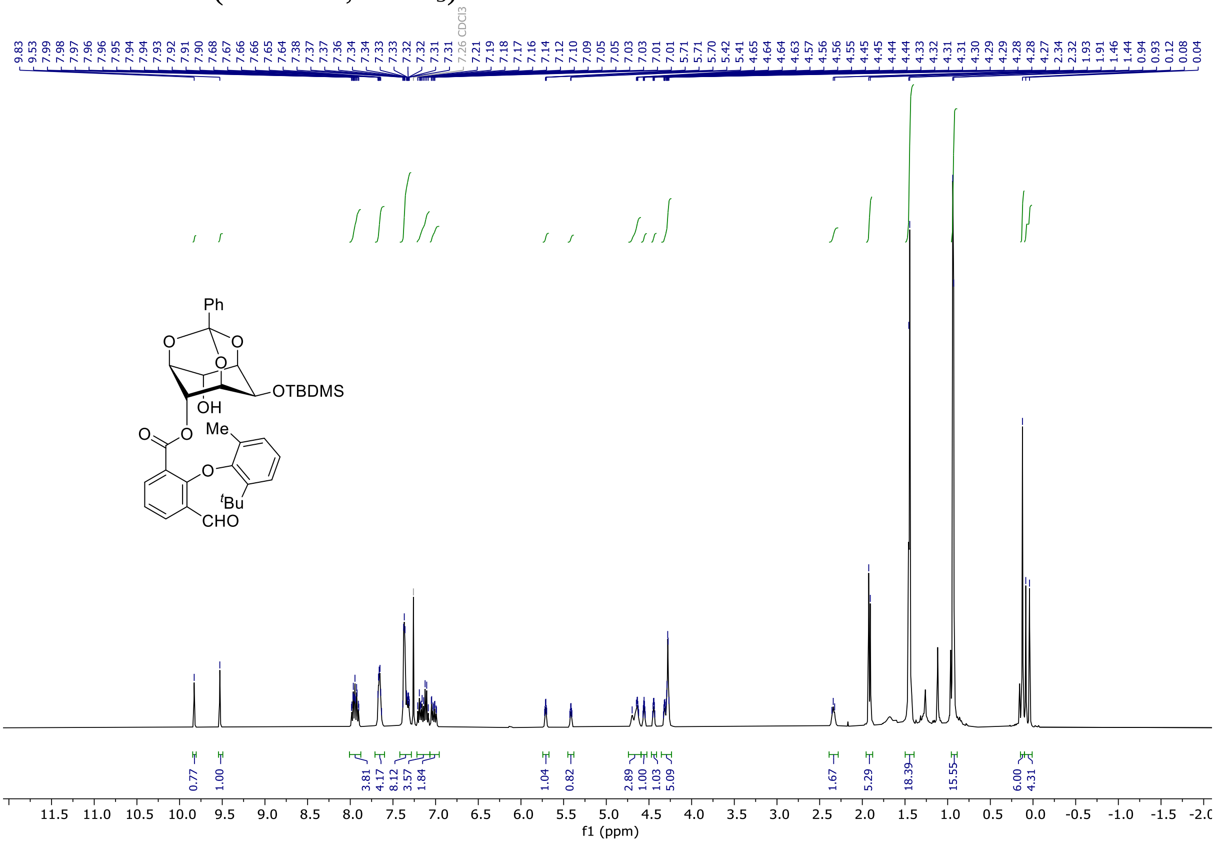

^{13}C{^{1}H} NMR for **5b** (101 MHz, $CDCl_3$)

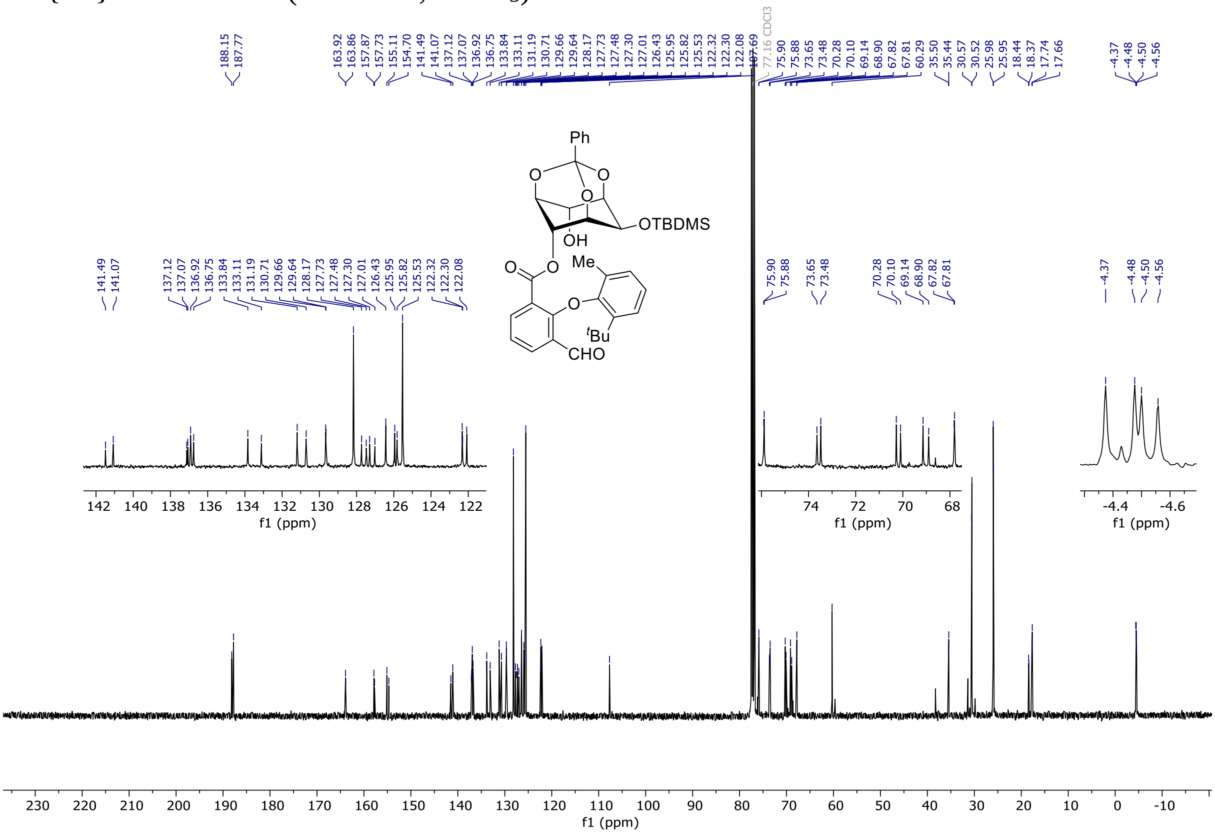

(1*R*,3*S*,5*S*,6*R*,7*R*,8*S*,9*S*)-8-((*tert*-Butoxycarbonyl)oxy)-9-((*tert*-butyldimethylsilyl)oxy)-3-phenyl-2,4,10-trioxaadamantan-6-yl cinnamate (6)

^{1}H NMR for **6** (400 MHz, $CDCl_3$)

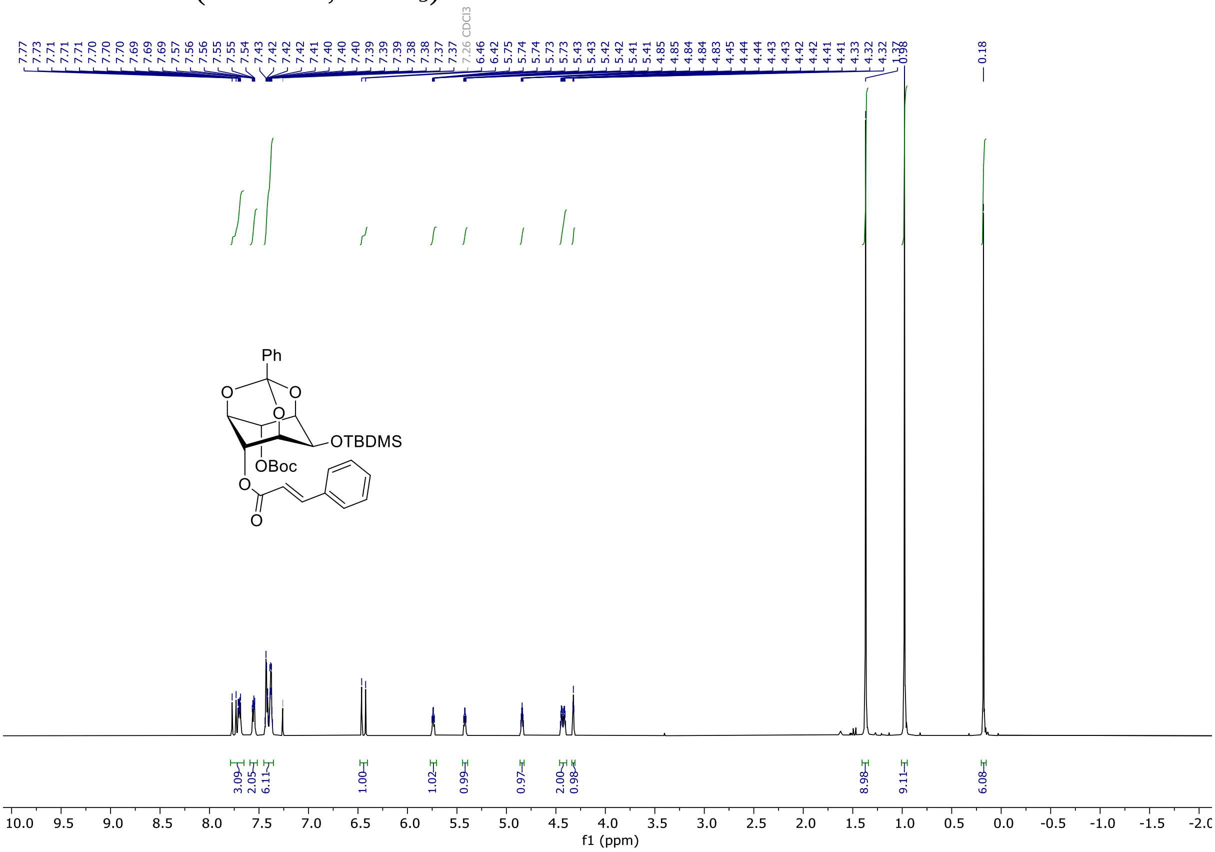

^{13}C{^{1}H} NMR for **6** (101 MHz, $CDCl_3$)

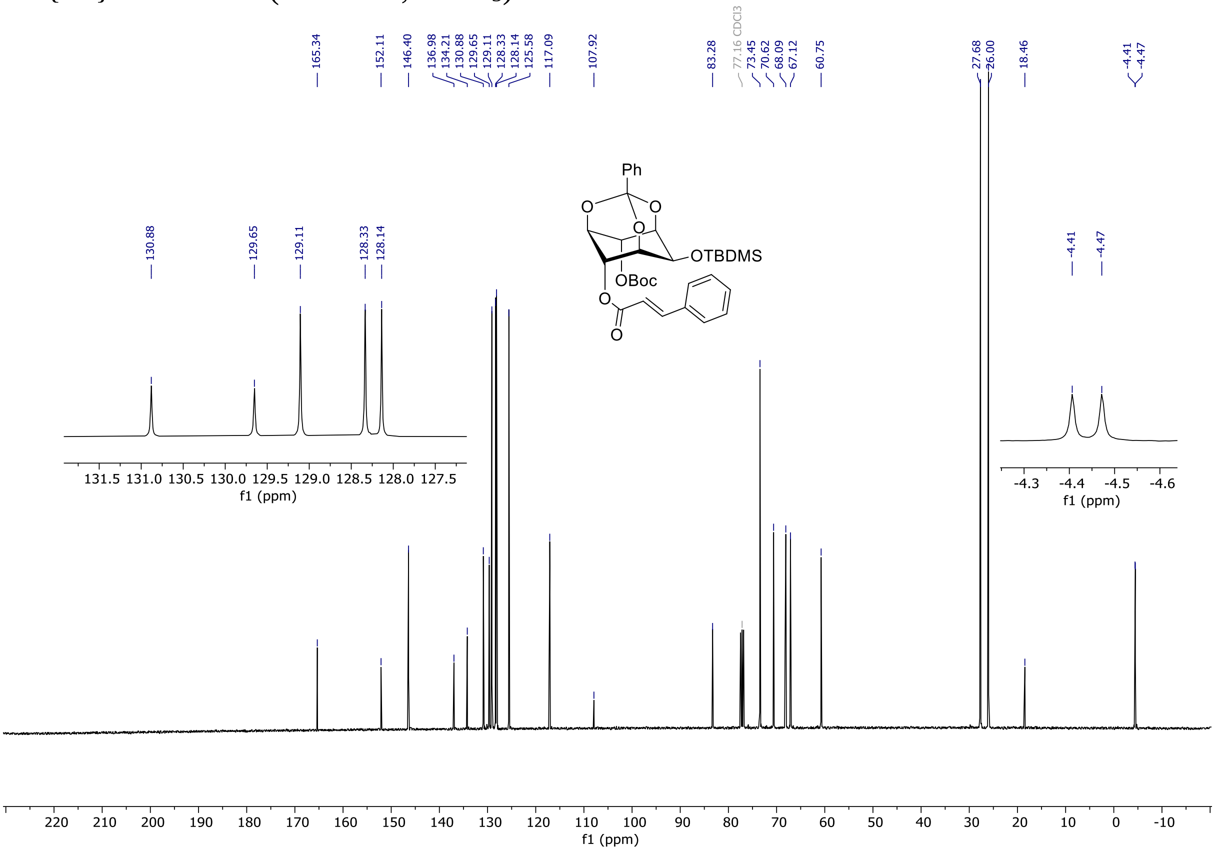

(1*R*,3*R*,5*S*,6*R*,7*S*,8*S*,9*R*)-8-Acetoxy-9-((*tert*-butyldimethylsilyl)oxy)-3-phenyl-2,4,10-trioxaadamantan-6-yl cinnamate (4a)

^{1}H NMR for **4a** (400 MHz, CDCl$_3$)

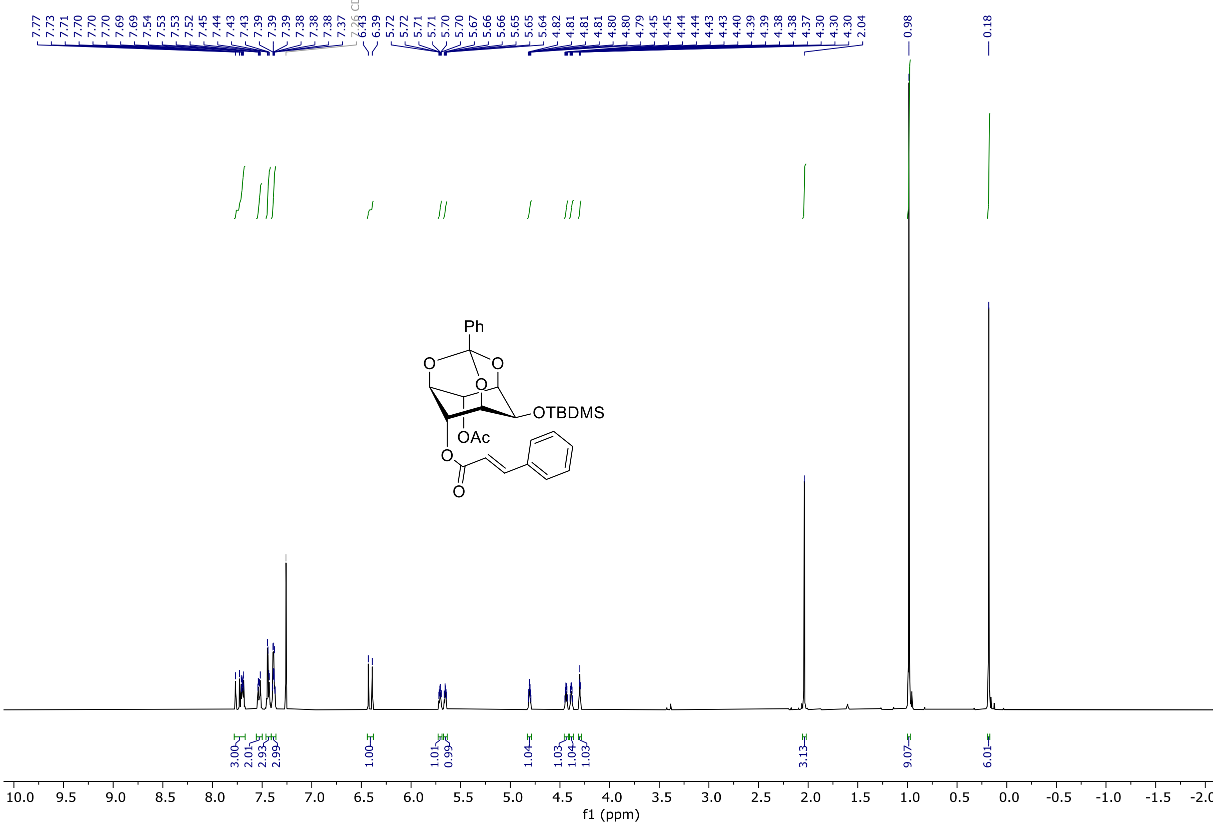

^{13}C{^{1}H} NMR **4a** (101 MHz, CDCl$_3$)

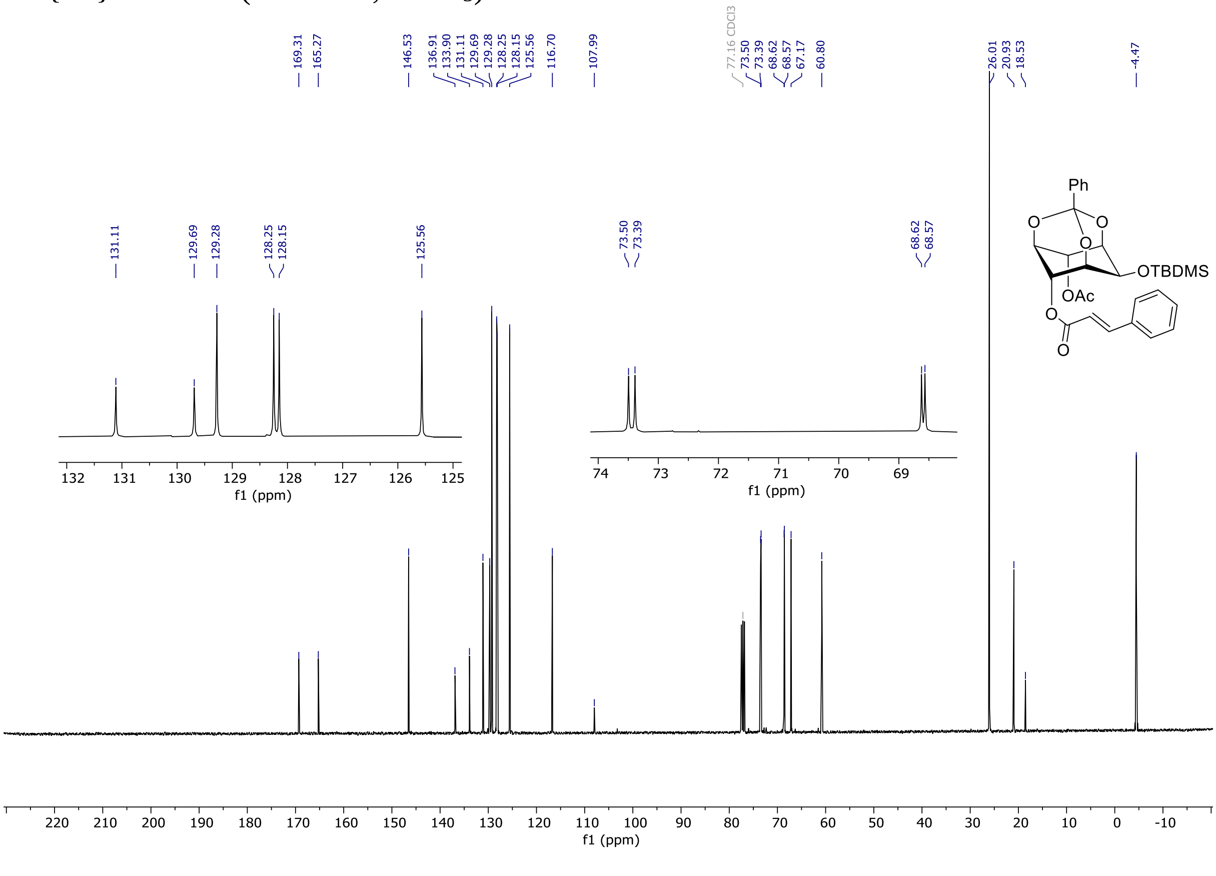

(1*S*,3*R*,5*R*,6*S*,7*S*,8*S*,9*S*)-8-((*tert*-butyldimethylsilyl)oxy)-9-(((2*S*,3a*S*,6*R*,7a*S*)-3a-methyl-6-(prop-1-en-2-yl)-2-sulfidohexahydrobenzo[*d*][1,3,2]oxathiaphosphol-2-yl)oxy)-3-phenyl-2,4,10-trioxaadamantan-6-yl cinnamate (7)

^{1}H NMR for **7** (400 MHz, $CDCl_3$)

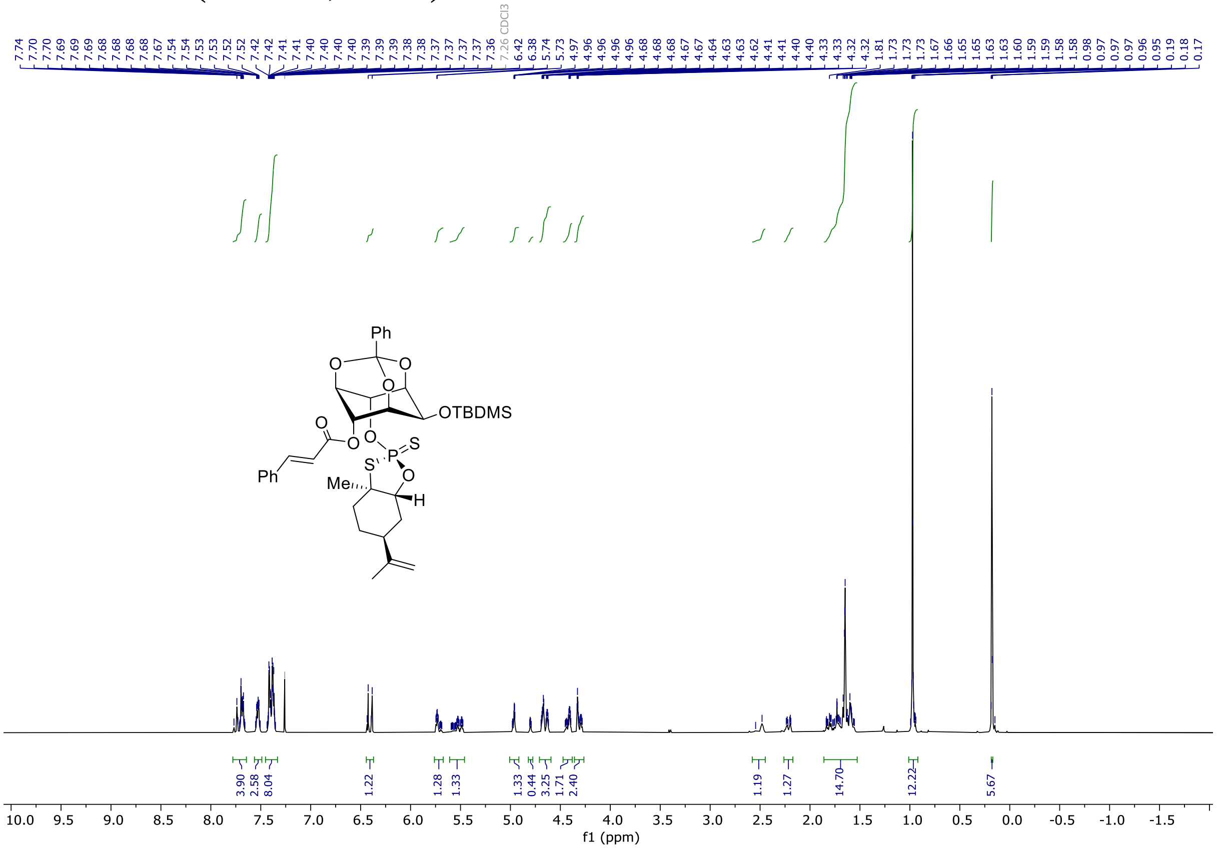

^{13}C{^{1}H} NMR for **7** (101 MHz, $CDCl_3$)

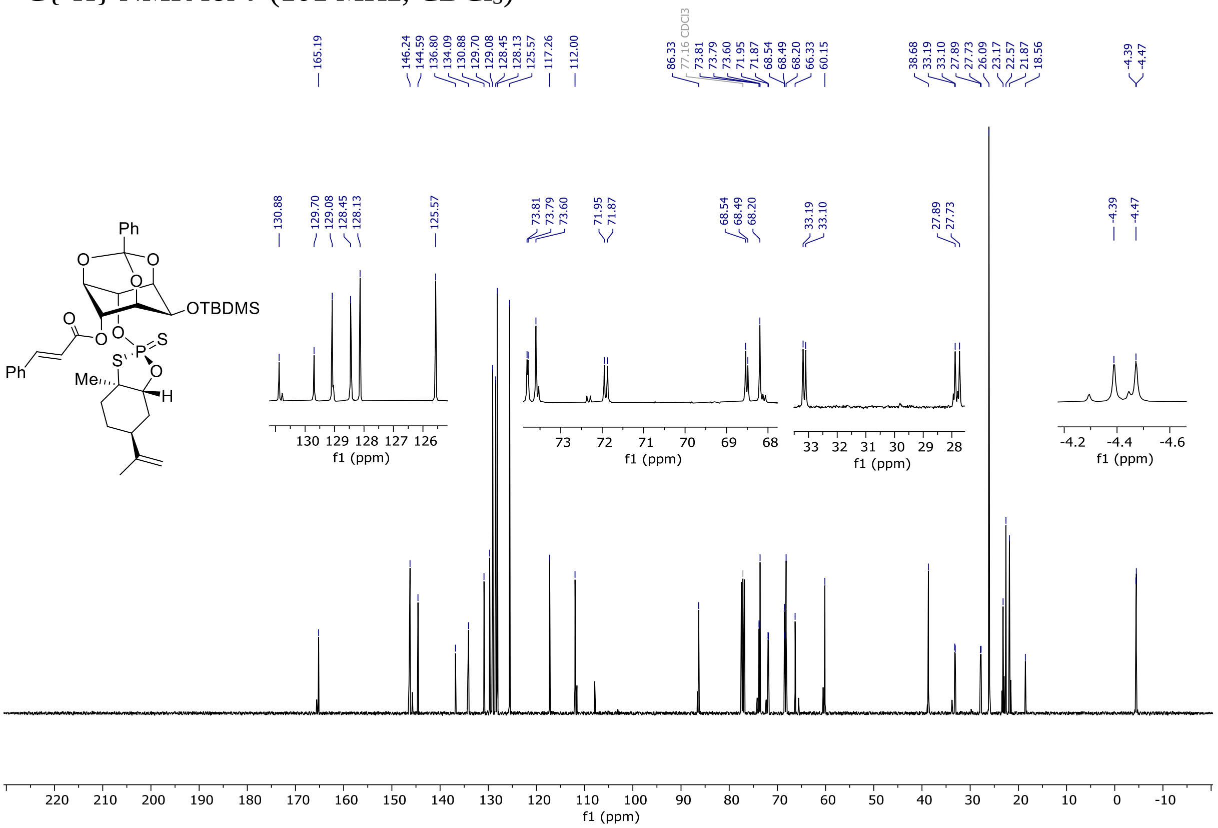

^{31}P NMR for **7** (162 MHz, $CDCl_3$)

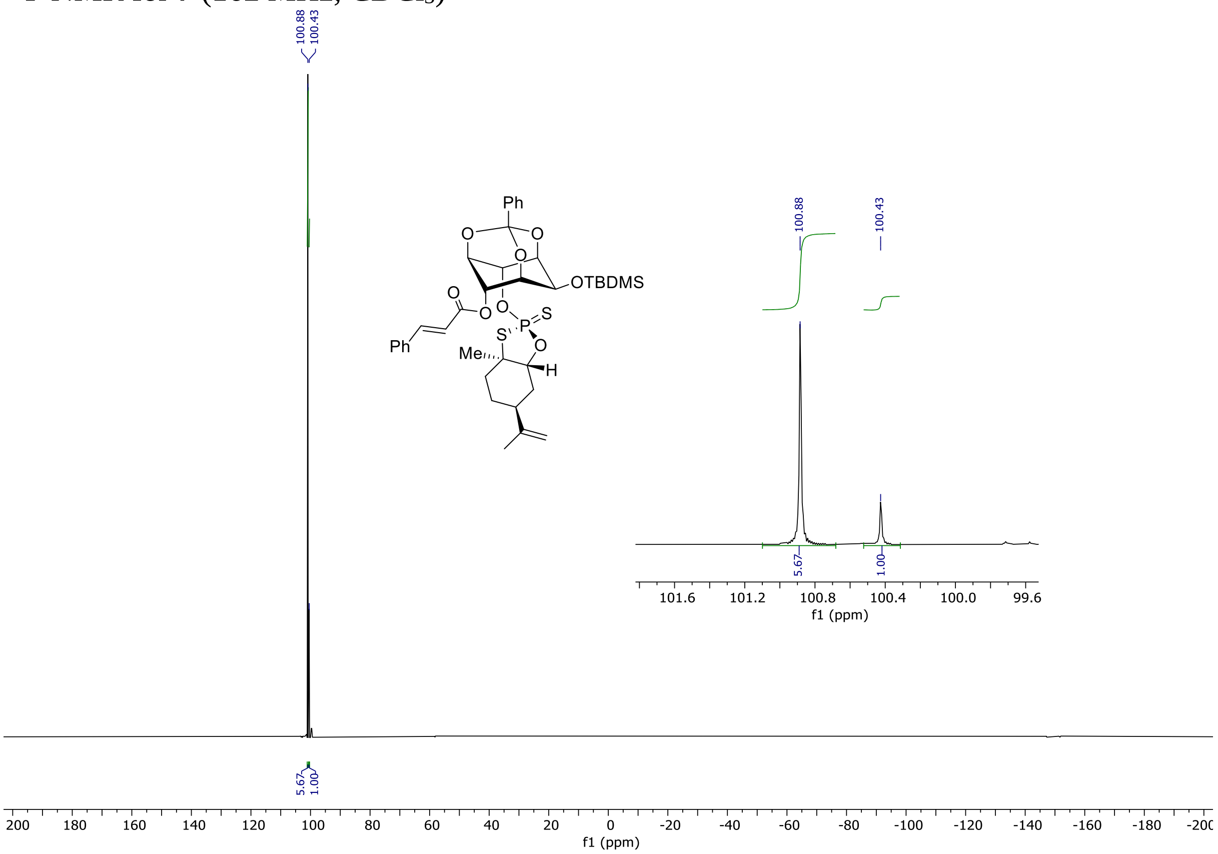

(1*S*,3*R*,5*S*,6*S*,7*R*,8*R*,9*R*)-8-((*tert*-Butyldimethylsilyl)oxy)-3-phenyl-9-(((*E*)-prop-1-en-1-yl)oxy)-2,4,10-trioxaadamantan-6-ol (11)

^{1}H NMR for **11** (400 MHz, $CDCl_3$)

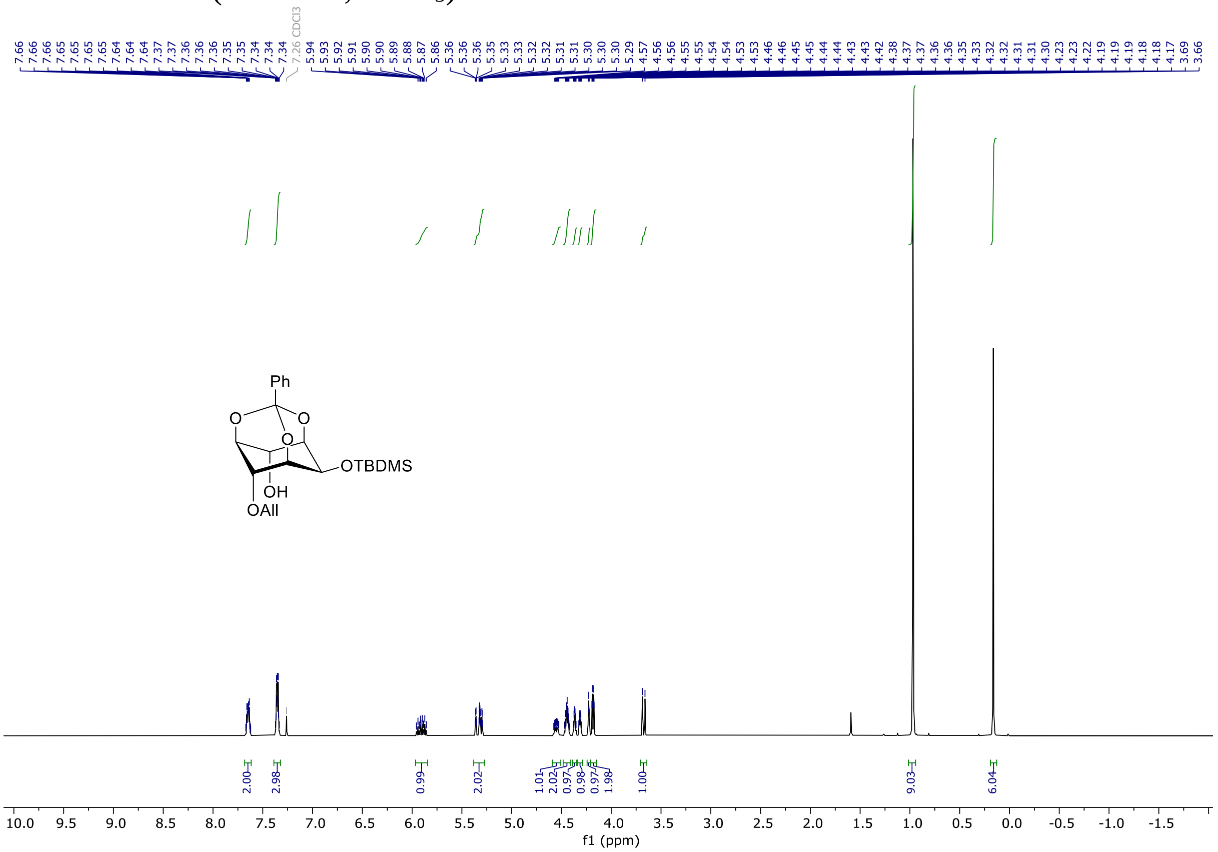

^{13}C{^{1}H} NMR for **11** (101 MHz, $CDCl_3$)

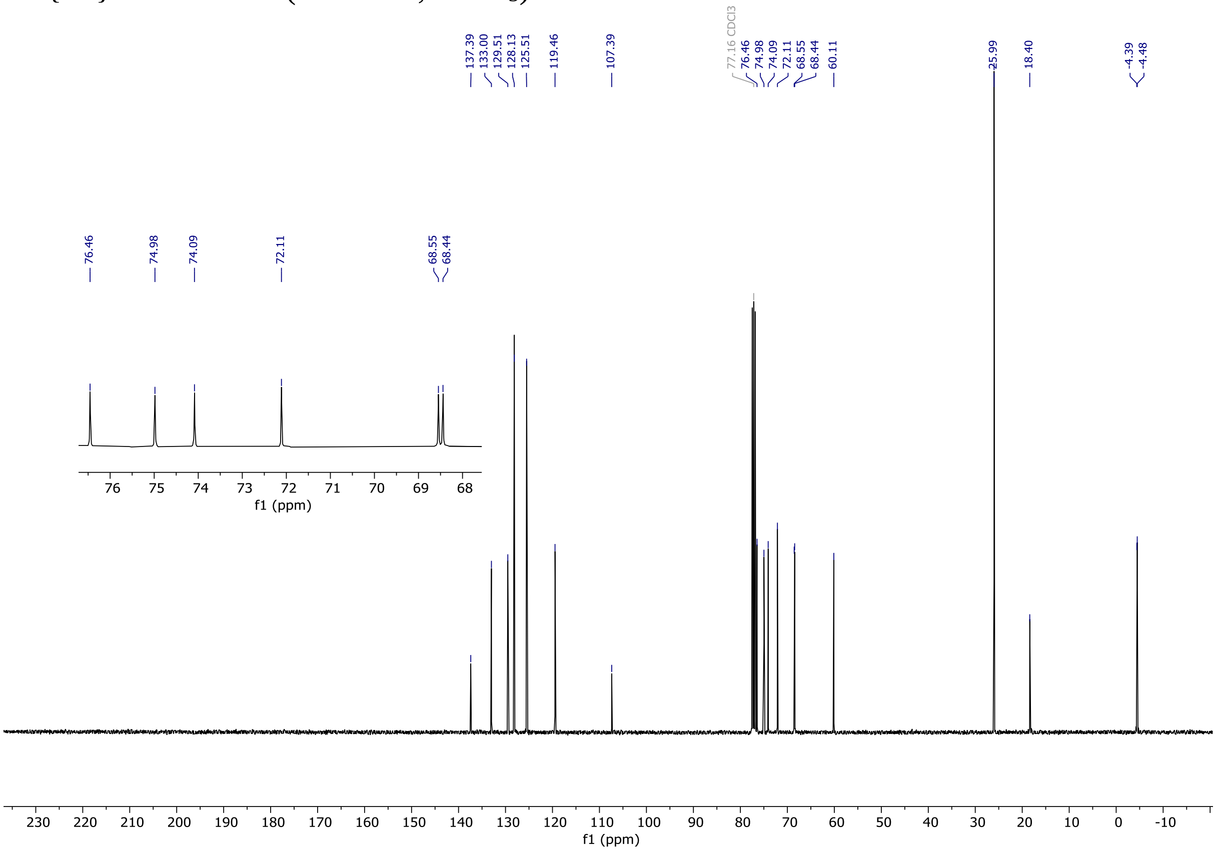

Chiral HPLC

(1*R*,3*R*,5*S*,6*R*,7*S*,8*R*,9*S*)-8-((*tert*-Butyldimethylsilyl)oxy)-9-hydroxy-3-phenyl-2,4,10-trioxaadamantan-6-yl cinnamate (3a)

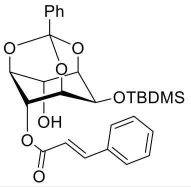

Conditions: IA column
mobile phase: *n*-heptane/*i*-PrOH – 95:5
λ = 275 nm, V = 1.0 ml/min, t = 25 °C
for **3a**: t_R = 13.4 min (minor), t_R = 17.5 min (major)

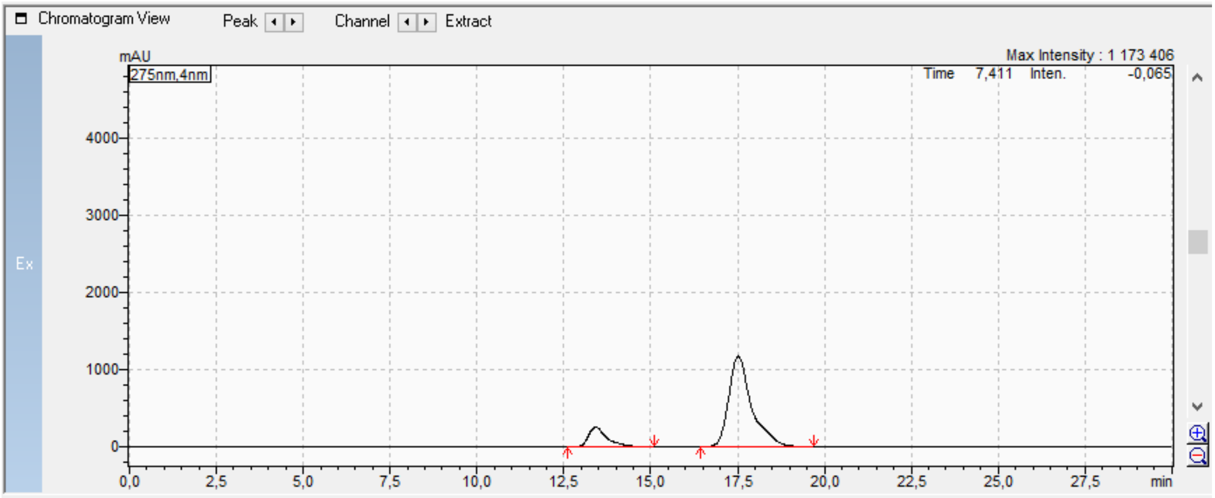

Results View - Peak Table

Peak Table | Compound | Group | Calibration Curve

Peak#	Ret. Time	Area	Height	Peak Start	Peak End	Mark	Conc.	Unit	Area%
1	13,418	9208133	254136	12,608	15,104	M	14,672		14,672
2	17,522	53550379	1172760	16,427	19,691	M	85,328		85,328
Total		62758512	1426896				100,000		100,000

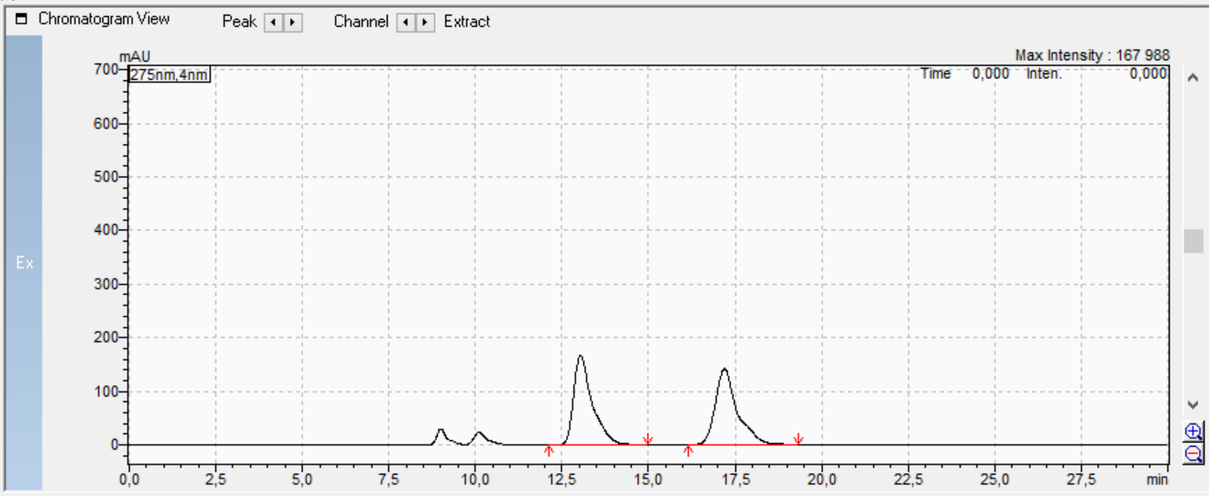

Results View - Peak Table

Peak Table | Compound | Group | Calibration Curve

Peak#	Ret. Time	Area	Height	Peak Start	Peak End	Mark	Conc.	Unit	Area%
1	13,036	6196902	167634	12,139	14,987	M	50,023		50,023
2	17,196	6191081	141266	16,139	19,328	M	49,977		49,977
Total		12387983	308901				100,000		100,000

for **3a**: *er* 85.3:14.7 (*ee* 70 %)

(1*S*,3*S*,5*R*,6*S*,7*R*,8*S*,9*R*)-8-((*tert*-butyldimethylsilyl)oxy)-9-hydroxy-3-phenyl-2,4,10-trioxaadamantan-6-yl cinnamate (*ent*-3a)

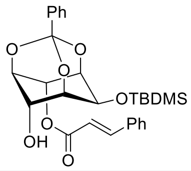

Conditions: IA column
mobile phase: *n*-heptane/*i*-PrOH – 95:5
λ = 275 nm, V = 1.0 ml/min, t = 25 °C
for ***ent*-3a**: t_R = 13.4 min (major), t_R = 17.5 min (minor)

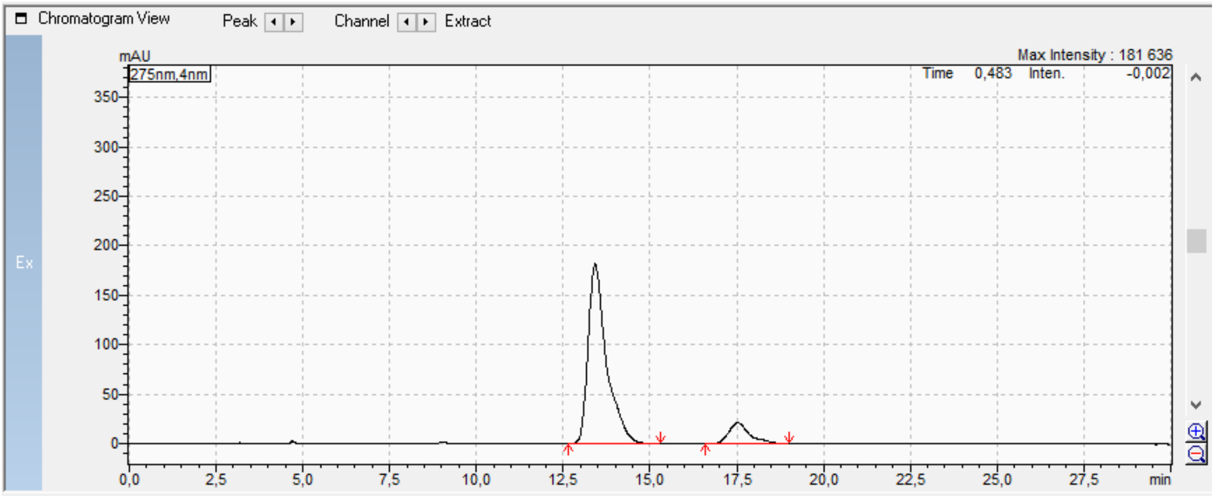

Results View - Peak Table

Peak Table | Compound | Group | Calibration Curve

Peak#	Ret. Time	Area	Height	Peak Start	Peak End	Mark	Conc.	Unit	Area%
1	13,424	6714565	181831	12,651	15,307	S	87,880		87,880
2	17,531	926034	21307	16,597	19,008		12,120		12,120
Total		7640599	203138				100,000		100,000

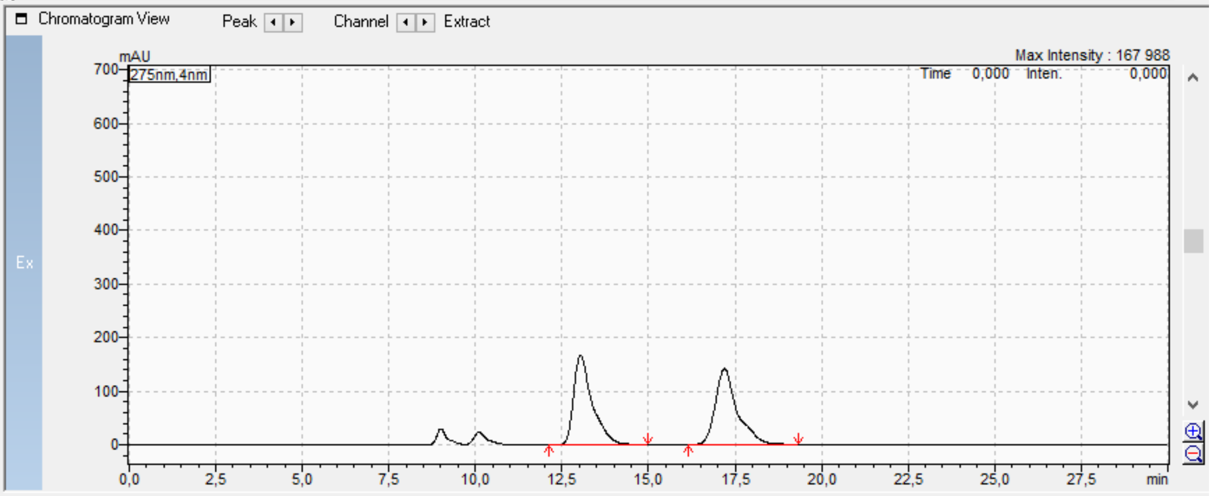

Results View - Peak Table

Peak Table | Compound | Group | Calibration Curve

Peak#	Ret. Time	Area	Height	Peak Start	Peak End	Mark	Conc.	Unit	Area%
1	13,036	6196902	167634	12,139	14,987	M	50,023		50,023
2	17,196	6191081	141266	16,139	19,328	M	49,977		49,977
Total		12387983	308901				100,000		100,000

for ***ent*-3a**: *er* = 87.9:12.1 (*ee* = 76 %)

(1*R*,3*R*,5*S*,6*R*,7*S*,8*R*,9*S*)-8-((*tert*-Butyldimethylsilyl)oxy)-9-hydroxy-2,4,10-trioxaadamantan-6-yl cinnamate (3b)

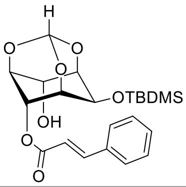

Conditions: IA column
mobile phase: *n*-heptane/*i*-PrOH – 95:5
λ = 277 nm, V = 1.0 ml/min, t = 25 °C
for **3b**: t_R = 15.8 min (minor), t_R = 19.7 min (major)

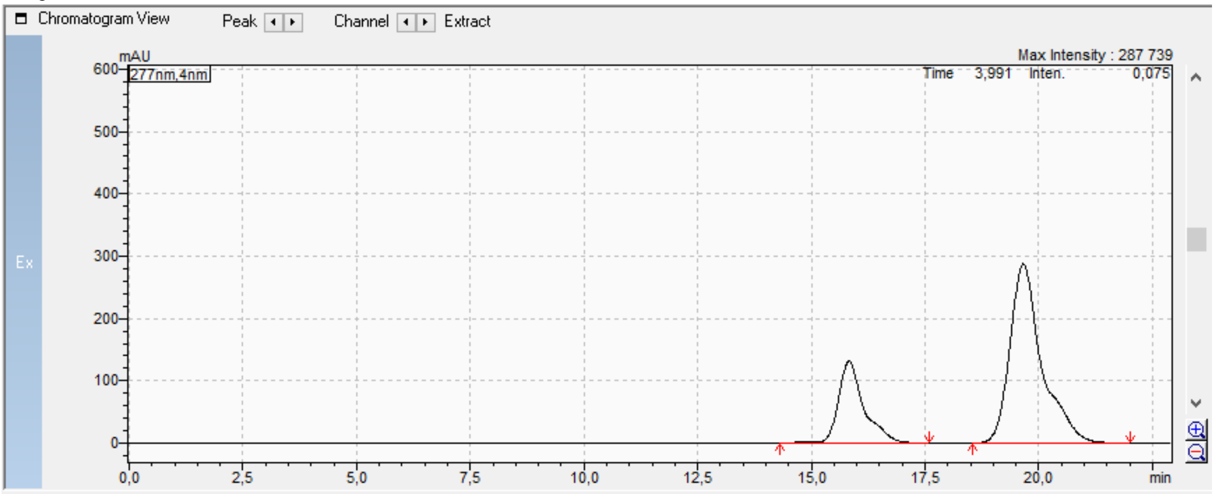

Results View - Peak Table

Peak Table | Compound | Group | Calibration Curve

Peak#	Ret. Time	Area	Height	Peak Start	Peak End	Mark	Conc.	Unit	Area%
1	15,829	5143280	131875	14,304	17,589	M	26,823		26,823
2	19,661	14031326	287461	18,539	22,016	M	73,177		73,177
Total		19174606	419336				100,000		100,000

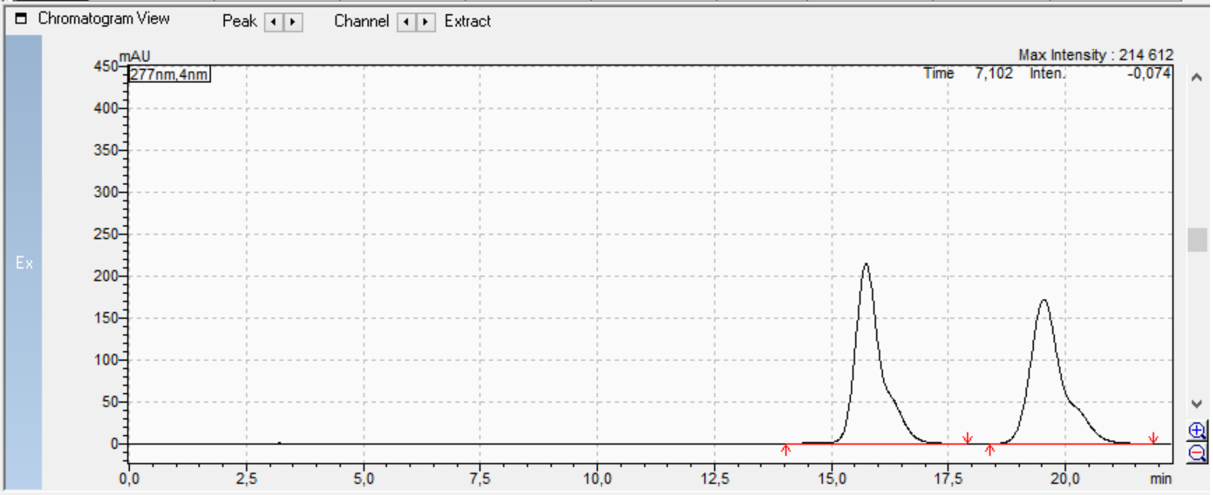

Results View - Peak Table

Peak Table | Compound | Group | Calibration Curve

Peak#	Ret. Time	Area	Height	Peak Start	Peak End	Mark	Conc.	Unit	Area%
1	15,738	8389307	214484	14,016	17,909	M	50,170		50,170
2	19,538	8332422	171988	18,368	21,888	M	49,830		49,830
Total		16721729	386472				100,000		100,000

for **3b**: *er* = 73.2:26.8 (*ee* = 46 %)

(1*R*,3*R*,5*S*,6*R*,7*S*,8*S*,9*R*)-8-Hydroxy-3-phenyl-9-((trimethylsilyl)oxy)-2,4,10-trioxaadamantan-6-yl cinnamate (3c)

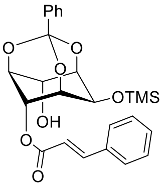

Conditions: IA column
mobile phase: *n*-heptane/*i*-PrOH – 95:5
λ = 277 nm, V = 1.0 ml/min, t = 25 °C
for **3c**: t_R = 15.8 min (minor), t_R = 23.3 min (major)

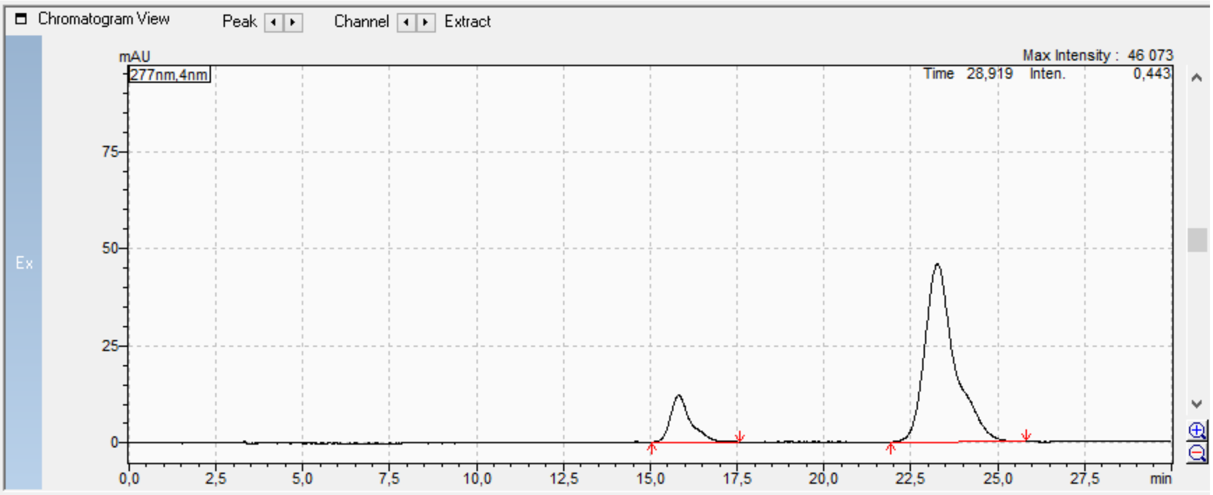

Results View - Peak Table

Peak Table | Compound | Group | Calibration Curve

Peak#	Ret. Time	Area	Height	Peak Start	Peak End	Mark	Conc.	Unit	Area%
1	15,813	481950	11980	15,051	17,579	M	14,943		14,943
2	23,263	2743288	45774	21,920	25,824	M	85,057		85,057
Total		3225237	57755				100,000		100,000

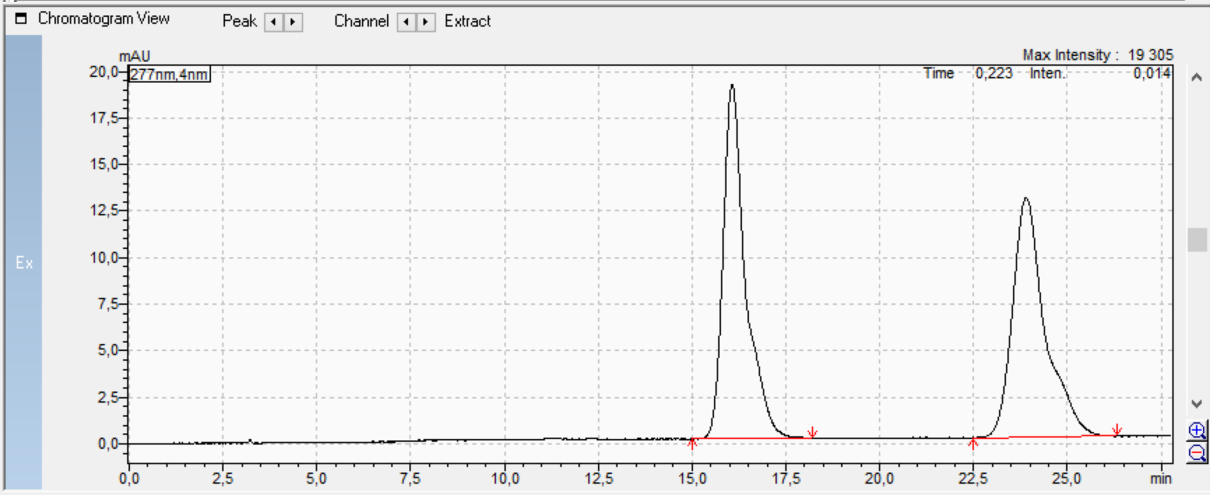

Results View - Peak Table

Peak Table | Compound | Group | Calibration Curve

Peak#	Ret. Time	Area	Height	Peak Start	Peak End	Mark	Conc.	Unit	Area%
1	16,070	785337	19011	14,987	18,219	M	50,359		50,359
2	23,891	774152	12830	22,496	26,336	M	49,641		49,641
Total		1559489	31841				100,000		100,000

for **3c**: *er* = 85.1:14.9 (*ee* = 70 %)

(1*R*,3*R*,5*S*,6*R*,7*S*,8*R*,9*S*)-8-((*tert*-Butyldiphenylsilyl)oxy)-9-hydroxy-3-phenyl-2,4,10-trioxaadamantan-6-yl cinnamate (3d)

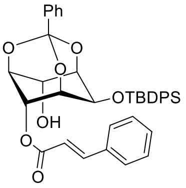

Conditions: IA column
mobile phase: *n*-heptane/*i*-PrOH – 97:3
λ = 190 nm, V = 1.0 ml/min, t = 25 °C
for **3d**: t_R = 13.4 min (minor), t_R = 16.1 min (major)

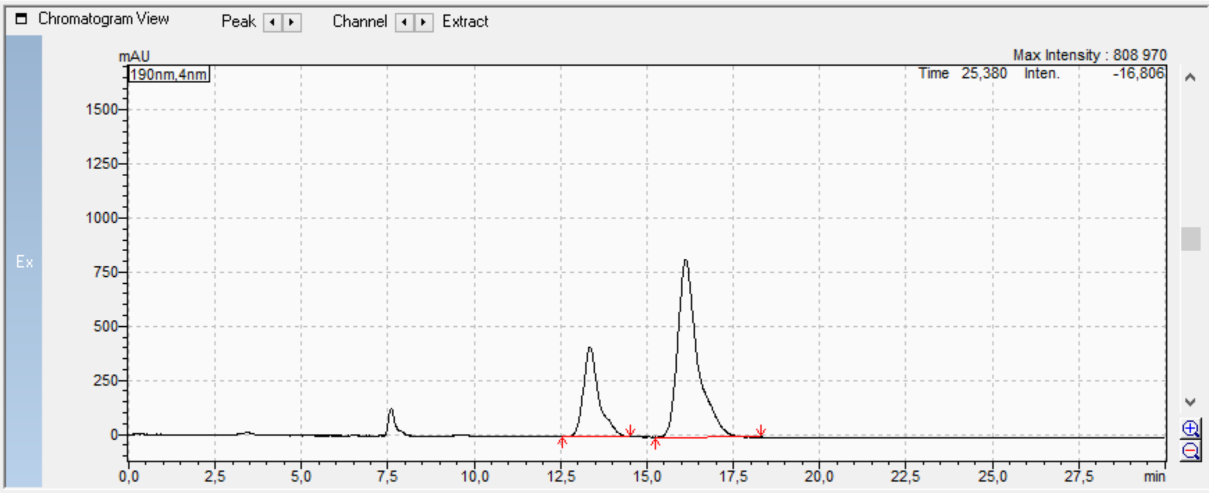

Results View - Peak Table

Peak Table | Compound | Group | Calibration Curve

Peak#	Ret. Time	Area	Height	Peak Start	Peak End	Mark	Conc.	Unit	Area%
1	13,352	12974104	413956	12,544	14,517	M	28,354		28,354
2	16,117	32783801	821922	15,243	18,304	M	71,646		71,646
Total		45757905	1235878				100,000		100,000

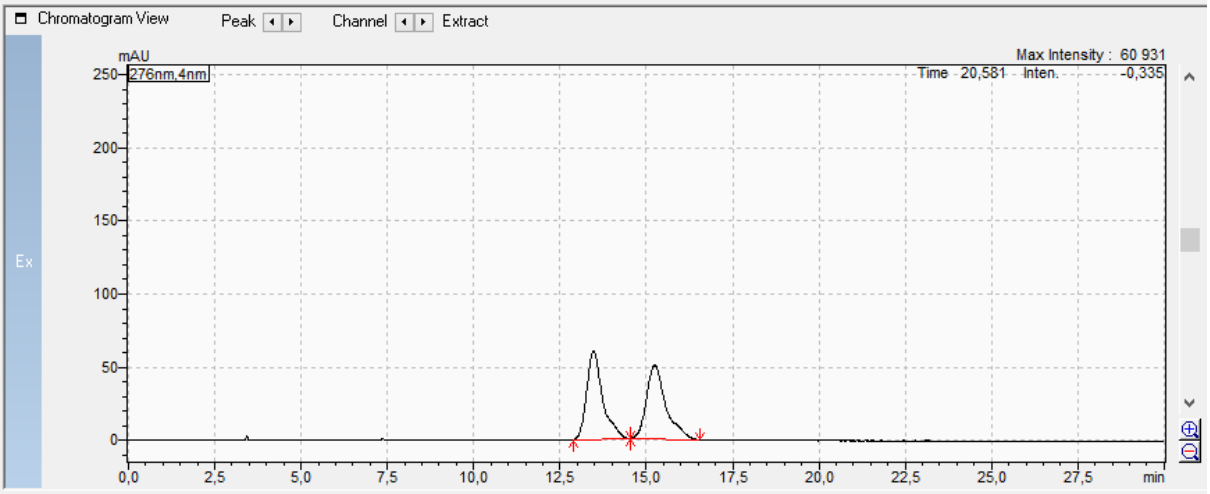

Results View - Peak Table

Peak Table | Compound | Group | Calibration Curve

Peak#	Ret. Time	Area	Height	Peak Start	Peak End	Mark	Conc.	Unit	Area%
1	13,473	1944770	60275	12,885	14,528	M	49,729		49,729
2	15,246	1965929	50406	14,528	16,555	M	50,271		50,271
Total		3910699	110681				100,000		100,000

for **3d**: *er* = 71.6:28.4 (*ee* = 43 %)

(1*S*,3*R*,5*R*,6*R*,7*S*,8*R*,9*S*)-8-(Cinnamoyloxy)-9-hydroxy-3-phenyl-2,4,10-trioxaadamantan-6-yl benzoate (3e)

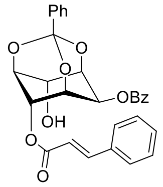

Conditions: IA column
mobile phase: *n*-heptane/*i*-PrOH – 90:10
λ = 276 nm, V = 1.0 ml/min, t = 25 °C
for **3e**: t_R = 16.9 min (major), t_R = 21.5 min (minor)

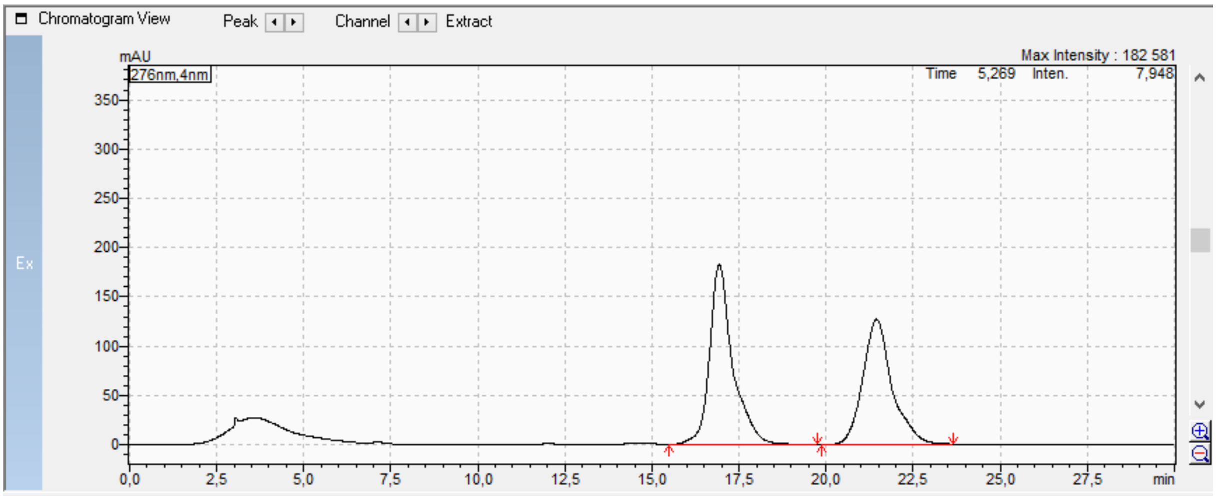

Results View - Peak Table

Peak Table | Compound | Group | Calibration Curve

Peak#	Ret. Time	Area	Height	Peak Start	Peak End	Mark	Conc.	Unit	Area%
1	16,934	8416339	182232	15,488	19,765	M	53,343		53,343
2	21,453	7361311	126711	19,883	23,648	M	46,657		46,657
Total		15777650	308943				100,000		100,000

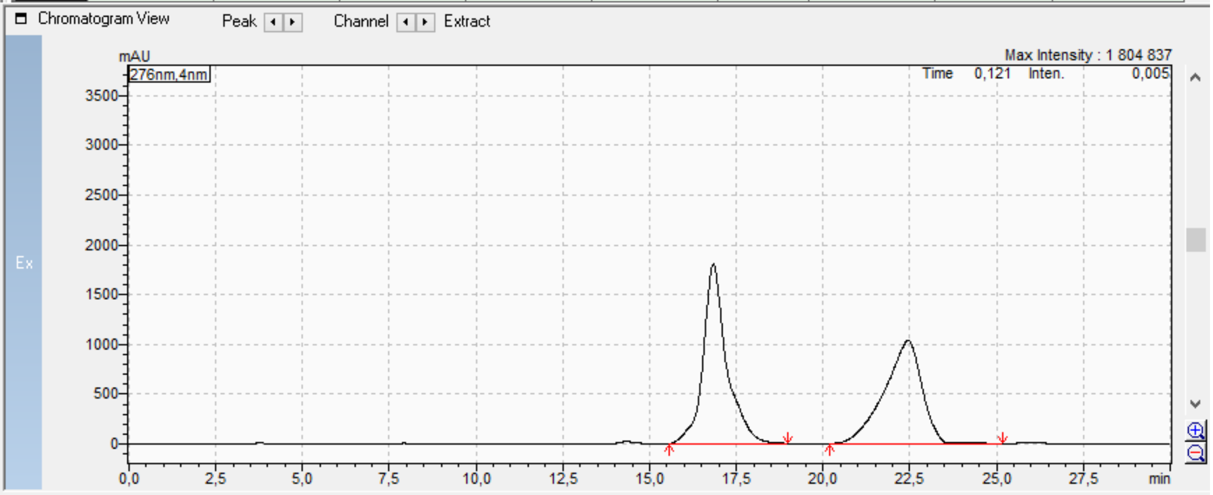

Results View - Peak Table

Peak Table | Compound | Group | Calibration Curve

Peak#	Ret. Time	Area	Height	Peak Start	Peak End	Mark	Conc.	Unit	Area%
1	16,840	84851372	1797895	15,573	18,987	M	52,136		52,136
2	22,455	77898037	1034522	20,181	25,163	M	47,864		47,864
Total		162749409	2832417				100,000		100,000

for **3e**: *er* = 53.3:46.7 (*ee* = 7 %)

(1*R*,3*R*,5*S*,6*R*,7*S*,8*R*,9*S*)-9-Hydroxy-3-phenyl-2,4,10-trioxaadamantane-6,8-diyl (2*E*,2'*E*)-bis(3-phenylacrylate) (3f)

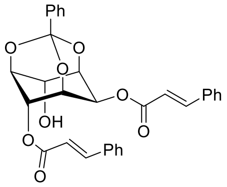

Conditions: IA column
mobile phase: *n*-heptane/*i*-PrOH – 90:10
λ = 276 nm, V = 1.0 ml/min, t = 25 °C
for **3f**: t_R = 23.3 min (major), t_R = 43.4 min (minor)

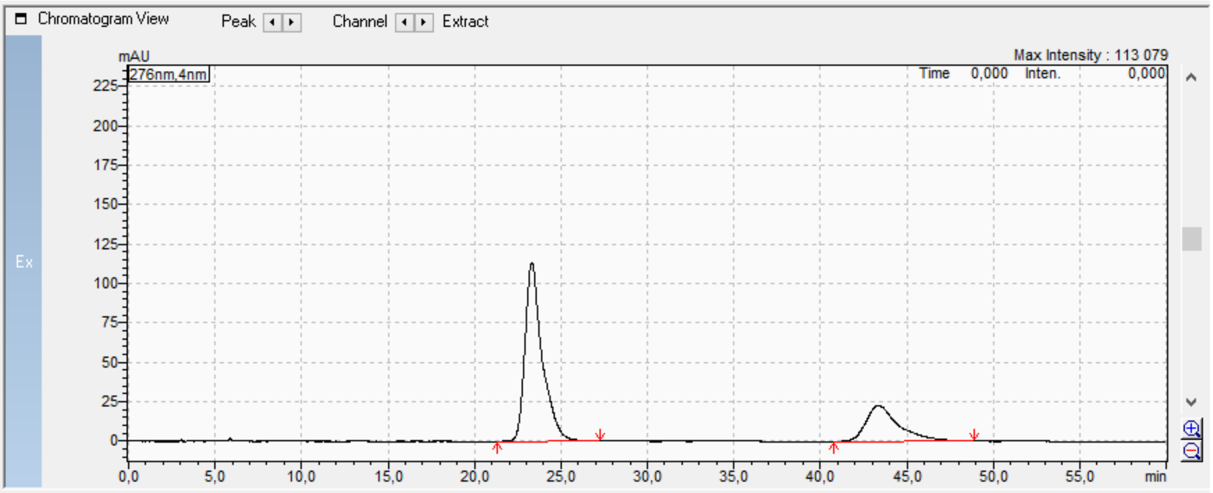

Results View - Peak Table

Peak Table | Compound | Group | Calibration Curve

Peak#	Ret. Time	Area	Height	Peak Start	Peak End	Mark	Conc.	Unit	Area%
1	23,318	7823044	113024	21,312	27,285	M	72,384		72,384
2	43,352	2984723	22623	40,800	48,885	M	27,616		27,616
Total		10807767	135647				100,000		100,000

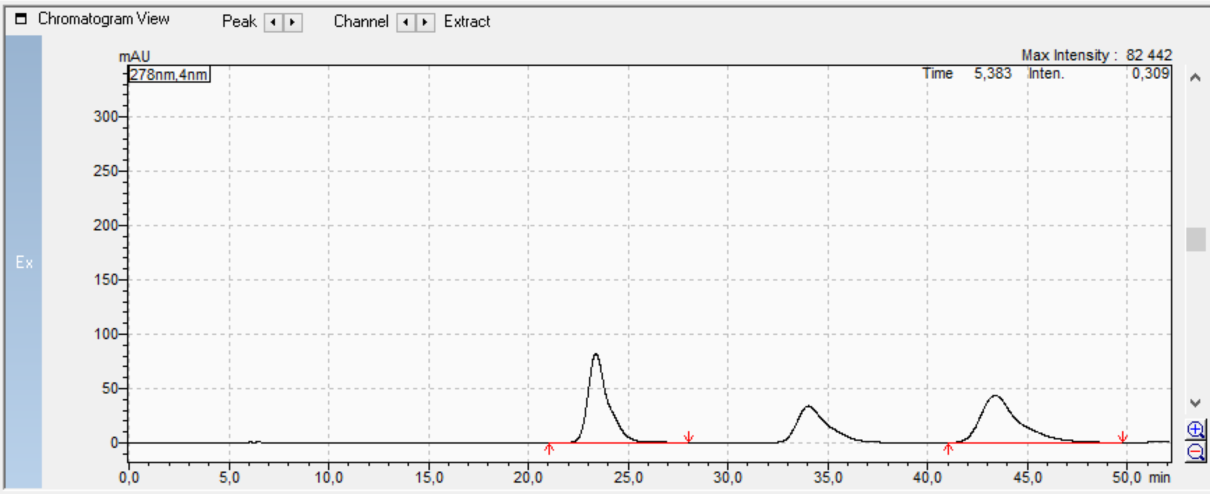

Results View - Peak Table

Peak Table | Compound | Group | Calibration Curve

Peak#	Ret. Time	Area	Height	Peak Start	Peak End	Mark	Conc.	Unit	Area%
1	23,381	5755583	82102	21,013	28,032	M	49,770		49,770
2	43,389	5808890	43470	41,003	49,781	M	50,230		50,230
Total		11564472	125572				100,000		100,000

for **3f**: *er* = 72.4:27.6 (*ee* = 45 %)

(1*R*,3*R*,5*S*,6*R*,7*S*,8*R*,9*S*)-8-((*tert*-Butyldimethylsilyl)oxy)-9-hydroxy-3-phenyl-2,4,10-trioxaadamantan-6-yl (*E*)-3-(naphthalen-2-yl)acrylate (3g)

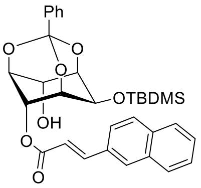

Conditions: IA column

mobile phase: *n*-heptane/*i*-PrOH – 95:5

λ = 271 nm, V = 1.0 ml/min, t = 25 °C

for **3g**: t_R = 17.2 min (minor), t_R = 24.5 min (major)

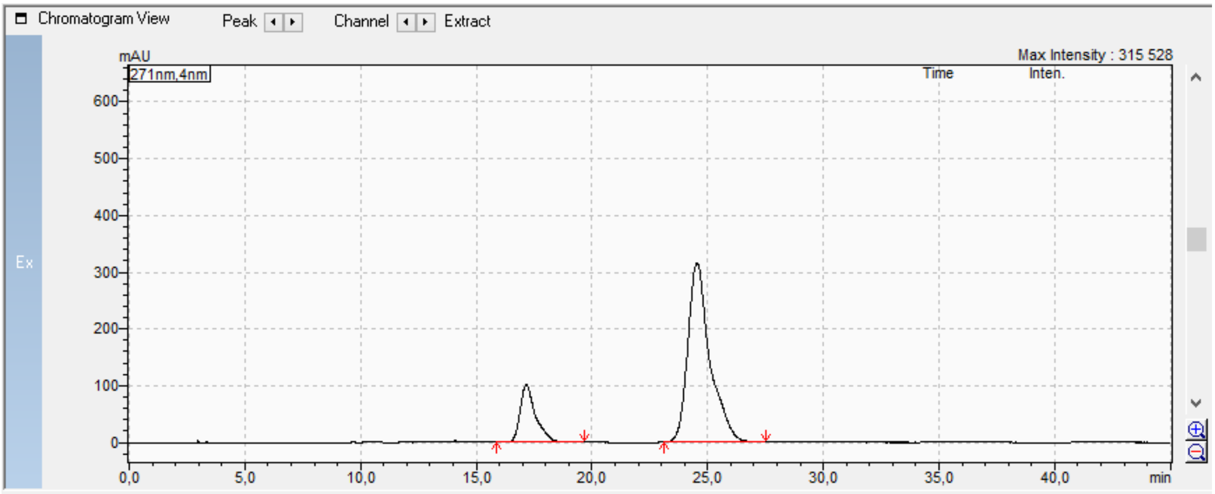

Results View - Peak Table

Peak Table | Compound | Group | Calibration Curve

Peak#	Ret. Time	Area	Height	Peak Start	Peak End	Mark	Conc.	Unit	Area%
1	17,166	4655730	100939	15,883	19,669	M	18,187		18,187
2	24,539	20943950	315020	23,125	27,541	M	81,813		81,813
Total		25599680	415959				100,000		100,000

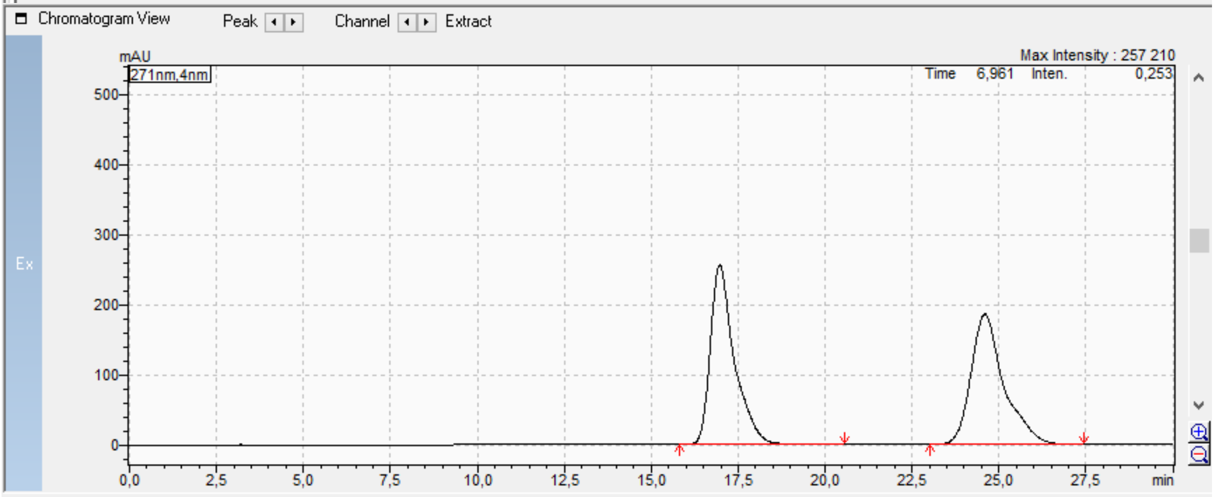

Results View - Peak Table

Peak Table | Compound | Group | Calibration Curve

Peak#	Ret. Time	Area	Height	Peak Start	Peak End	Mark	Conc.	Unit	Area%
1	16,973	12355020	256267	15,808	20,565	M	50,210		50,210
2	24,595	12251785	186482	23,029	27,424	M	49,790		49,790
Total		24606805	442749				100,000		100,000

for **3g**: *er* = 81.8:18.2 (*ee* = 64 %)

(1*R*,3*R*,5*S*,6*R*,7*S*,8*R*,9*S*)-8-((*tert*-Butyldimethylsilyl)oxy)-9-hydroxy-3-phenyl-2,4,10-trioxaadamantan-6-yl (*E*)-3-(*p*-tolyl)acrylate (3h)

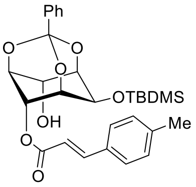

Conditions: IA column
mobile phase: *n*-heptane/*i*-PrOH – 95:5
λ = 284 nm, V = 1.0 ml/min, t = 25 °C
for **3h**: t_R = 17.1 min (minor), t_R = 20.6 min (major)

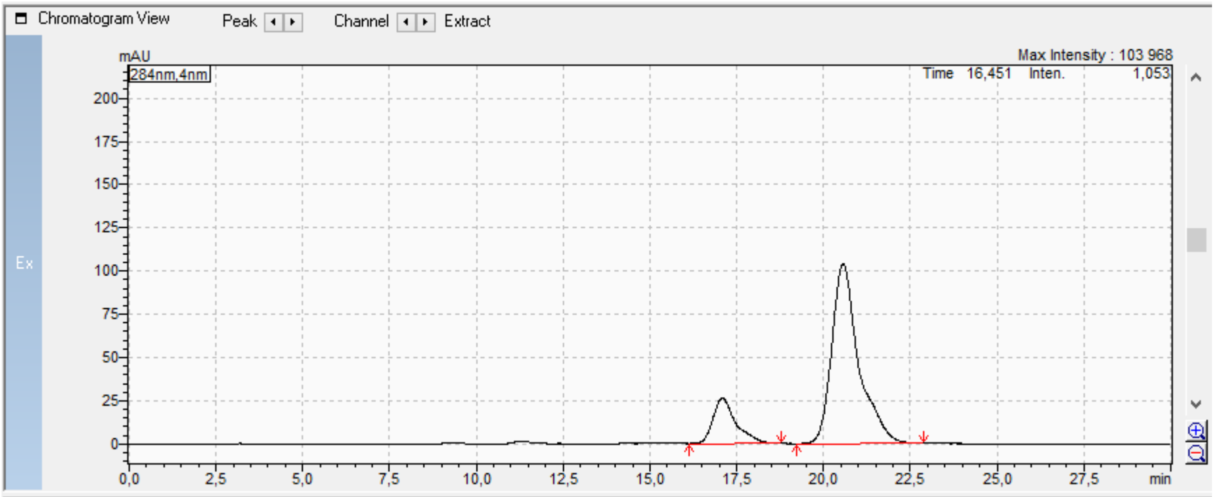

Results View - Peak Table

Peak Table | Compound | Group | Calibration Curve

Peak#	Ret. Time	Area	Height	Peak Start	Peak End	Mark	Conc.	Unit	Area%
1	17,094	1205568	26090	16,139	18,784	M	17,688		17,688
2	20,564	5610143	103705	19,221	22,880	M	82,312		82,312
Total		6815711	129795				100,000		100,000

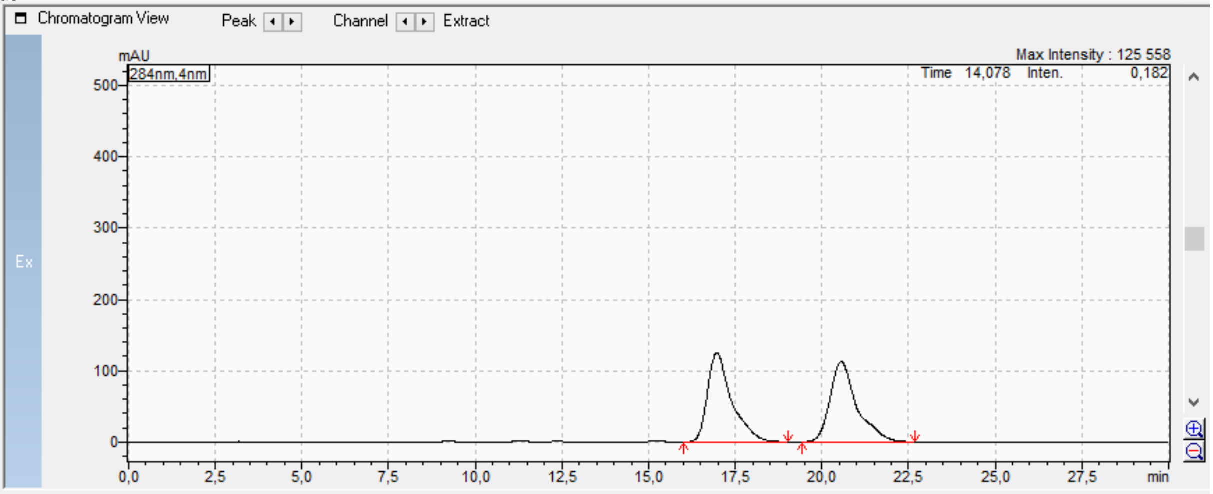

Results View - Peak Table

Peak Table | Compound | Group | Calibration Curve

Peak#	Ret. Time	Area	Height	Peak Start	Peak End	Mark	Conc.	Unit	Area%
1	16,964	6050852	125165	16,021	19,029	M	50,056		50,056
2	20,556	6037213	112247	19,424	22,677	M	49,944		49,944
Total		12088065	237412				100,000		100,000

for **3h**: *er* = 82.3:17.7 (*ee* = 65 %)

(1*R*,3*R*,5*S*,6*R*,7*S*,8*R*,9*S*)-8-((*tert*-Butyldimethylsilyl)oxy)-9-hydroxy-3-phenyl-2,4,10-trioxaadamantan-6-yl (*E*)-3-(4-methoxyphenyl)acrylate (3j)

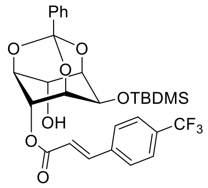

Conditions: IA column
mobile phase: *n*-heptane/*i*-PrOH – 95:5
λ = 271 nm, V = 1.0 ml/min, t = 25 °C
for **3j**: t_R = 10.6 min (minor), t_R = 15.1 min (major)

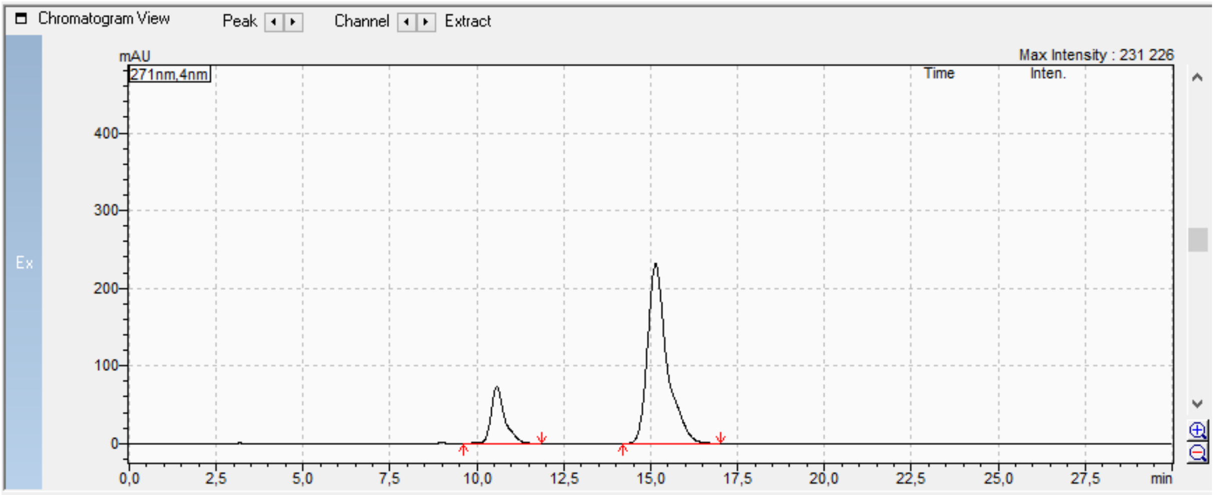

Results View - Peak Table

Peak Table | Compound | Group | Calibration Curve

Peak#	Ret. Time	Area	Height	Peak Start	Peak End	Mark	Conc.	Unit	Area%
1	10,575	1995034	73324	9,611	11,861	M	18,073		18,073
2	15,147	9043736	231244	14,197	17,003	M	81,927		81,927
Total		11038770	304568				100,000		100,000

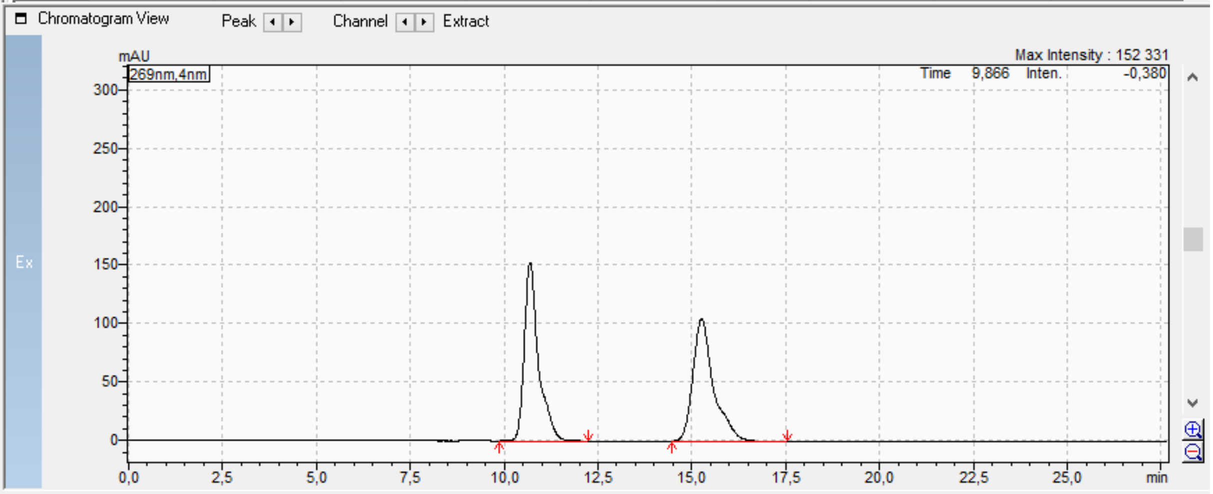

Results View - Peak Table

Peak Table | Compound | Group | Calibration Curve

Peak#	Ret. Time	Area	Height	Peak Start	Peak End	Mark	Conc.	Unit	Area%
1	10,692	4025675	152728	9,877	12,245	M	50,160		50,160
2	15,259	4000015	104762	14,475	17,547	M	49,840		49,840
Total		8025690	257490				100,000		100,000

for **3j**: *er* = 81.9:18.1 (*ee* = 64 %)

(1*R*,3*R*,5*S*,6*R*,7*S*,8*R*,9*S*)-8-((*tert*-Butyldimethylsilyl)oxy)-9-hydroxy-3-phenyl-2,4,10-trioxaadamantan-6-yl (*E*)-3-(4-fluorophenyl)acrylate (3l)

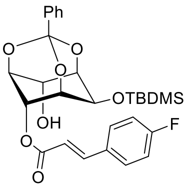

Conditions: IA column
mobile phase: *n*-heptane/*i*-PrOH – 95:5
λ = 190 nm, V = 1.0 ml/min, t = 25 °C
for **3l**: t_R = 12.5 min (minor), t_R = 16.3 min (major)

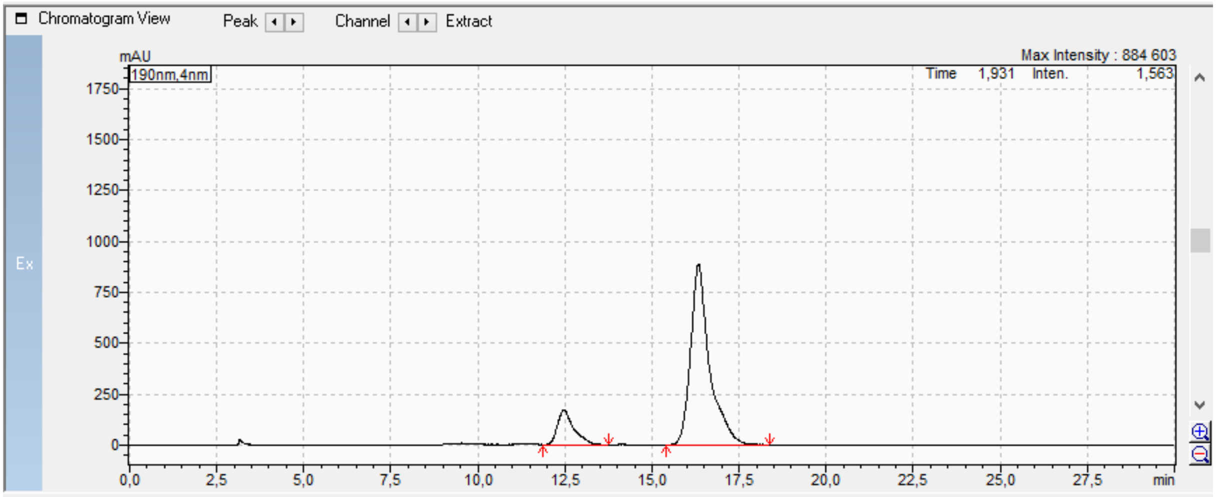

Results View - Peak Table

Peak Table | Compound | Group | Calibration Curve

Peak#	Ret. Time	Area	Height	Peak Start	Peak End	Mark	Conc.	Unit	Area%
1	12,464	5560339	168472	11,883	13,771	M	14,212		14,212
2	16,324	33563908	883897	15,413	18,400	M	85,788		85,788
Total		39124246	1052369				100,000		100,000

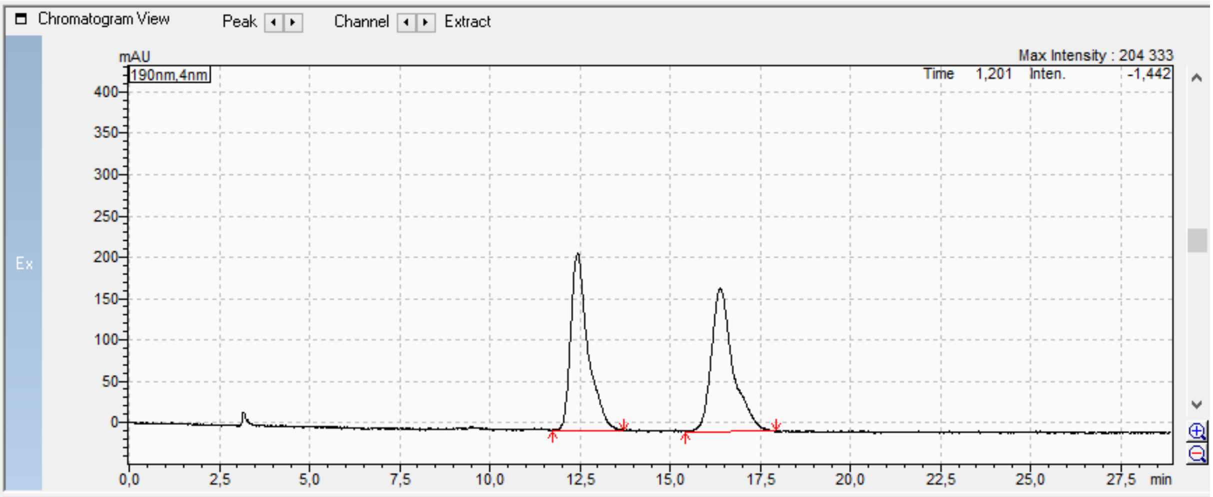

Results View - Peak Table

Peak Table | Compound | Group | Calibration Curve

Peak#	Ret. Time	Area	Height	Peak Start	Peak End	Mark	Conc.	Unit	Area%
1	12,436	7103539	213897	11,744	13,717	M	49,622		49,622
2	16,392	7211818	173496	15,424	17,941	M	50,378		50,378
Total		14315357	387393				100,000		100,000

for **3l**: *er* = 85.8:14.2 (*ee* = 72 %)

(1*R*,3*R*,5*S*,6*R*,7*S*,8*R*,9*S*)-8-((*tert*-Butyldimethylsilyl)oxy)-9-hydroxy-3-phenyl-2,4,10-trioxaadamantan-6-yl (*E*)-3-(4-chlorophenyl)acrylate (3m)

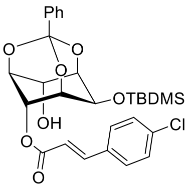

Conditions: IA column
mobile phase: *n*-heptane/*i*-PrOH – 95:5
λ = 283 nm, V = 1.0 ml/min, t = 25 °C
for **3m**: t_R = 12.7 min (minor), t_R = 17.1 min (major)

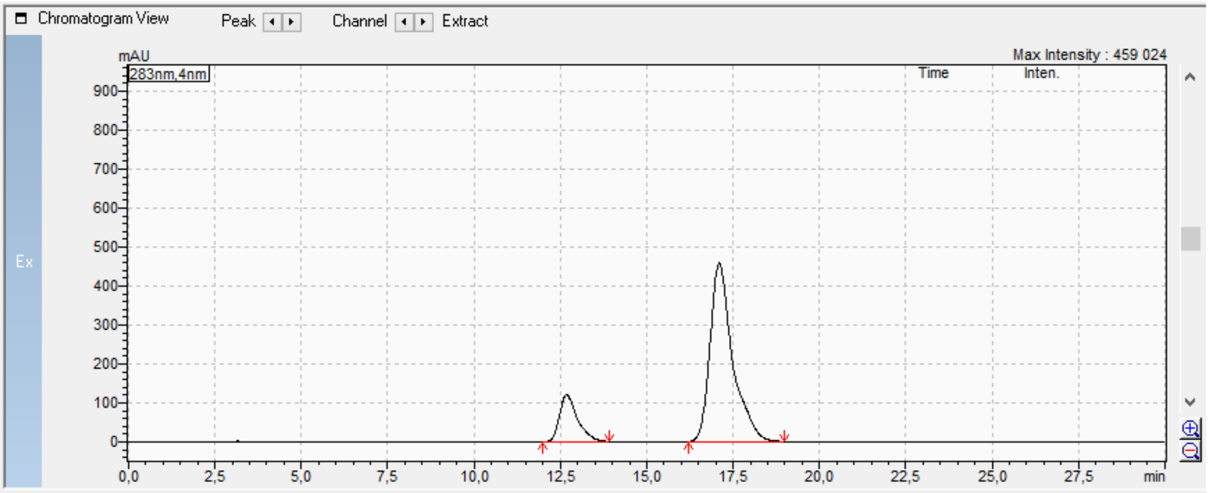

Results View - Peak Table

Peak Table | Compound | Group | Calibration Curve

Peak#	Ret. Time	Area	Height	Peak Start	Peak End	Mark	Conc.	Unit	Area%
1	12,679	4337796	120071	11,979	13,931	M	17,251		17,251
2	17,105	20807213	458295	16,192	18,987	M	82,749		82,749
Total		25145009	578366				100,000		100,000

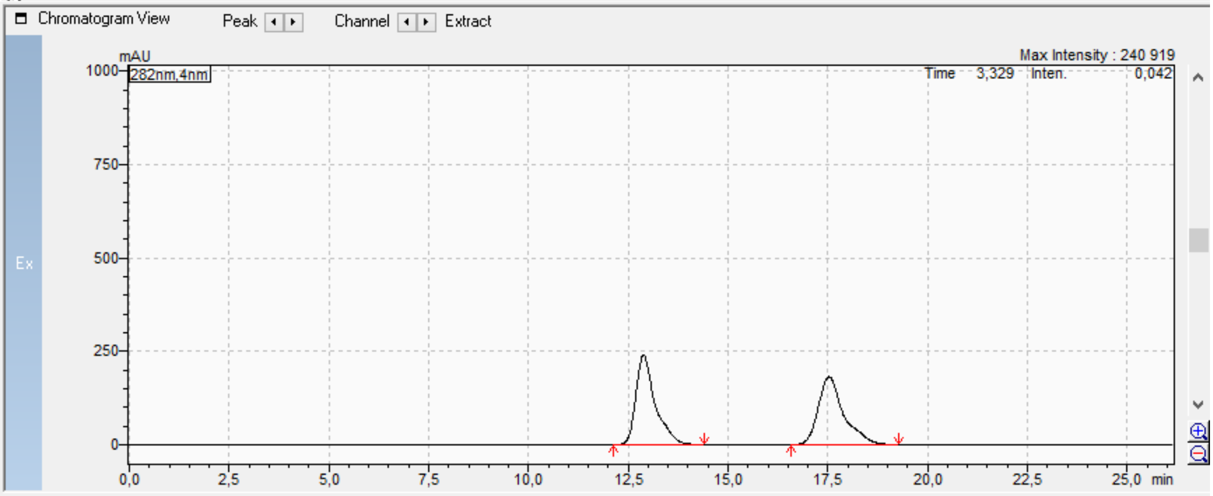

Results View - Peak Table

Peak Table | Compound | Group | Calibration Curve

Peak#	Ret. Time	Area	Height	Peak Start	Peak End	Mark	Conc.	Unit	Area%
1	12,882	8040379	239865	12,139	14,389	M	49,994		49,994
2	17,529	8042346	181569	16,576	19,275	M	50,006		50,006
Total		16082725	421434				100,000		100,000

for **3m**: *er* = 82.7:17.3 (*ee* = 65 %)

(1*R*,3*R*,5*S*,6*R*,7*S*,8*R*,9*S*)-8-((*tert*-Butyldimethylsilyl)oxy)-9-hydroxy-3-phenyl-2,4,10-trioxaadamantan-6-yl (*E*)-3-(4-bromophenyl)acrylate (3n)

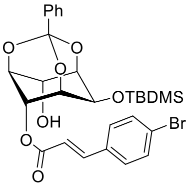

Conditions: IA column
mobile phase: *n*-heptane/*i*-PrOH – 95:5
λ = 286 nm, V = 1.0 ml/min, t = 25 °C
for **3n**: t_R = 13.0 min (minor), t_R = 18.1 min (major)

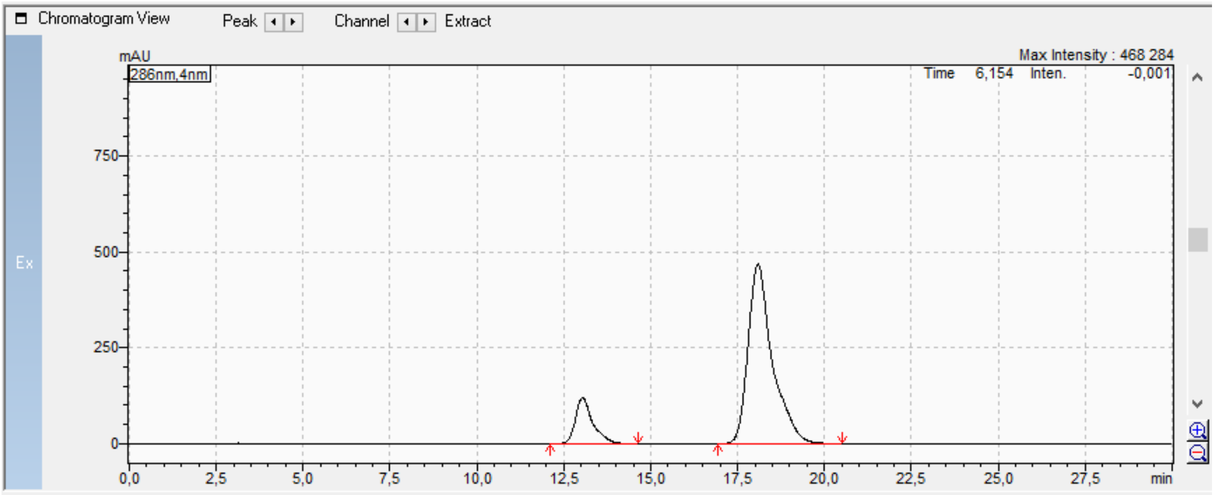

Results View - Peak Table

Peak Table | Compound | Group | Calibration Curve

Peak#	Ret. Time	Area	Height	Peak Start	Peak End	Mark	Conc.	Unit	Area%
1	13,041	4153904	119647	12,117	14,635	M	15,434		15,434
2	18,085	22760190	467908	16,949	20,512	M	84,566		84,566
Total		26914094	587555				100,000		100,000

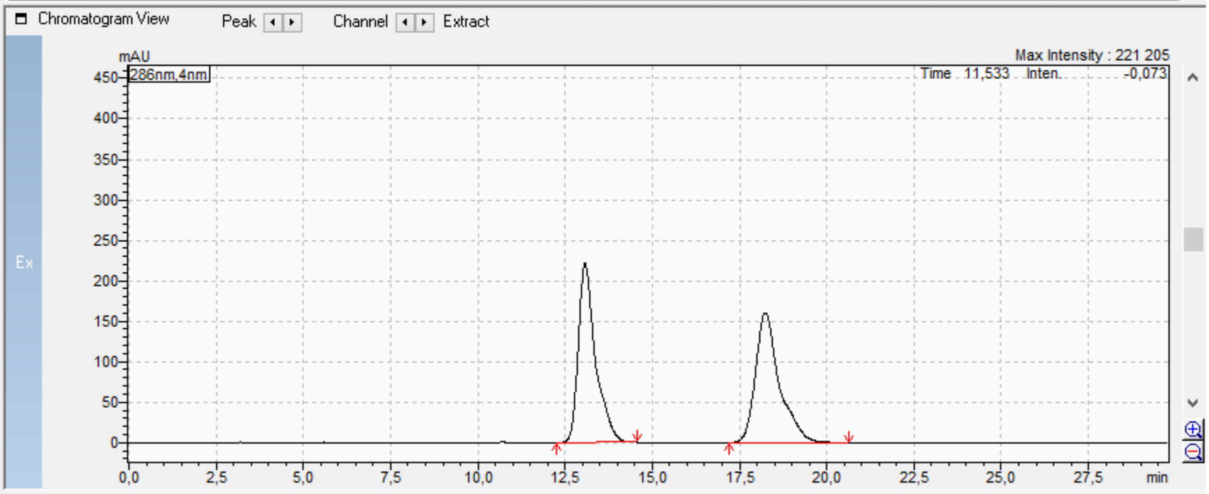

Results View - Peak Table

Peak Table | Compound | Group | Calibration Curve

Peak#	Ret. Time	Area	Height	Peak Start	Peak End	Mark	Conc.	Unit	Area%
1	13,073	7590209	221087	12,256	14,560	M	49,953		49,953
2	18,241	7604406	160212	17,205	20,629	M	50,047		50,047
Total		15194615	381299				100,000		100,000

for **3n**: *er* = 84.6:15.4 (*ee* = 69 %)

(1*R*,3*R*,5*S*,6*R*,7*S*,8*R*,9*S*)-8-((*tert*-Butyldimethylsilyl)oxy)-9-hydroxy-3-phenyl-2,4,10-trioxaadamantan-6-yl (*E*)-3-(3-chlorophenyl)acrylate (3o)

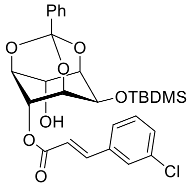

Conditions: IA column
mobile phase: *n*-heptane/*i*-PrOH – 95:5
λ = 209 nm, V = 1.0 ml/min, t = 25 °C
for **3o**: t_R = 10.1 min (minor), t_R = 13.7 min (major)

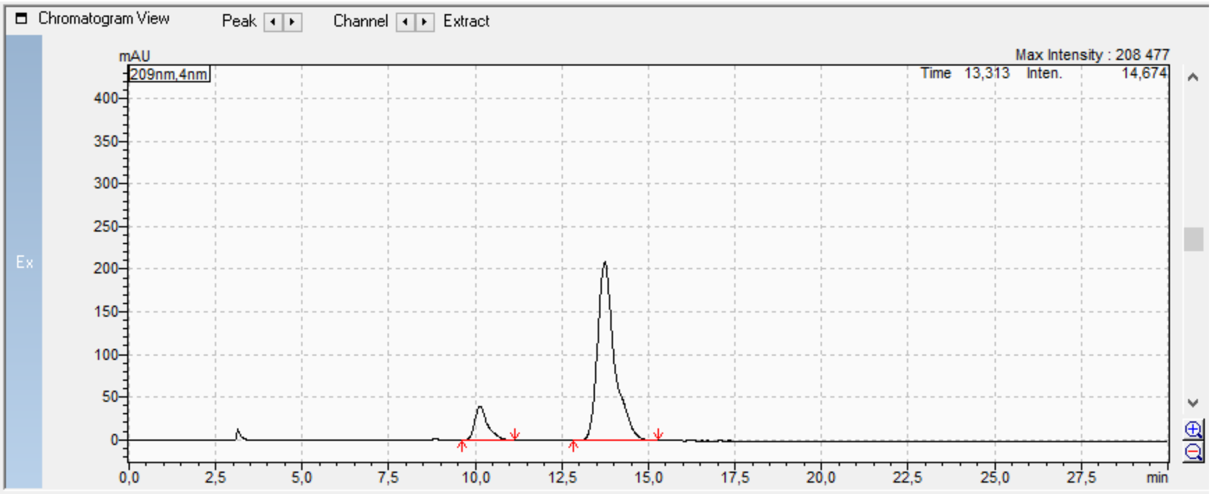

Results View - Peak Table

Peak Table | Compound | Group | Calibration Curve

Peak#	Ret. Time	Area	Height	Peak Start	Peak End	Mark	Conc.	Unit	Area%
1	10,133	1041513	40083	9,611	11,136	M	12,669		12,669
2	13,740	7179564	209570	12,832	15,285	M	87,331		87,331
Total		8221078	249653				100,000		100,000

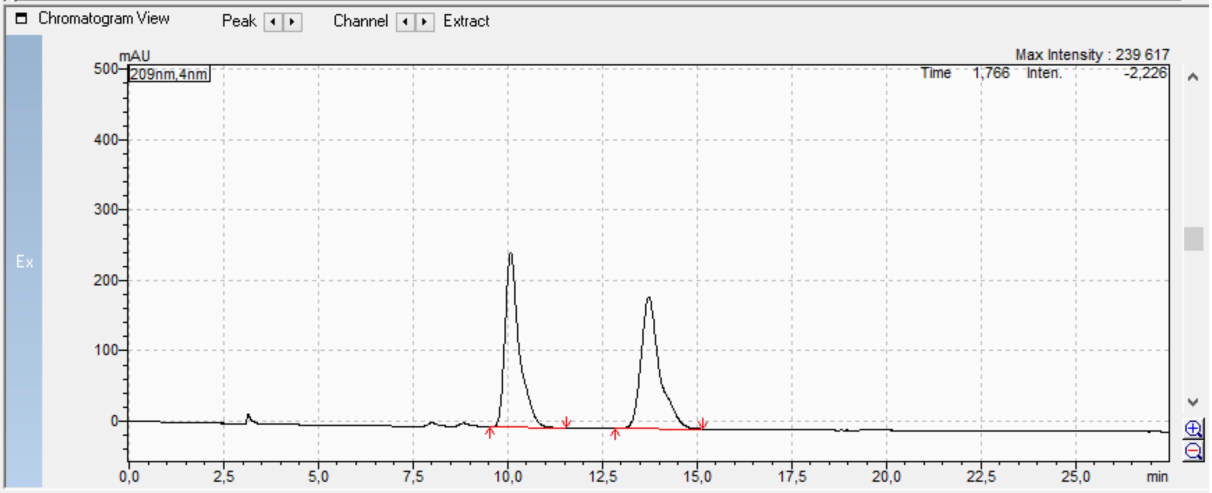

Results View - Peak Table

Peak Table | Compound | Group | Calibration Curve

Peak#	Ret. Time	Area	Height	Peak Start	Peak End	Mark	Conc.	Unit	Area%
1	10,080	6504337	248186	9,525	11,552	M	50,399		50,399
2	13,731	6401407	187783	12,843	15,157	M	49,601		49,601
Total		12905744	435970				100,000		100,000

for **3o**: *er* = 87.3:12.7 (*ee* = 75 %)

(1*R*,3*R*,5*S*,6*R*,7*S*,8*R*,9*S*)-8-((*tert*-Butyldimethylsilyl)oxy)-9-hydroxy-3-phenyl-2,4,10-trioxaadamantan-6-yl (*E*)-3-(2-chlorophenyl)acrylate (3p)

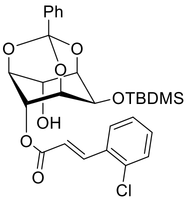

Conditions: IA column
mobile phase: *n*-heptane/*i*-PrOH – 95:5
λ = 276 nm, V = 1.0 ml/min, t = 25 °C
for **3p**: t_R = 13.4 min (minor), t_R = 15.9 min (major)

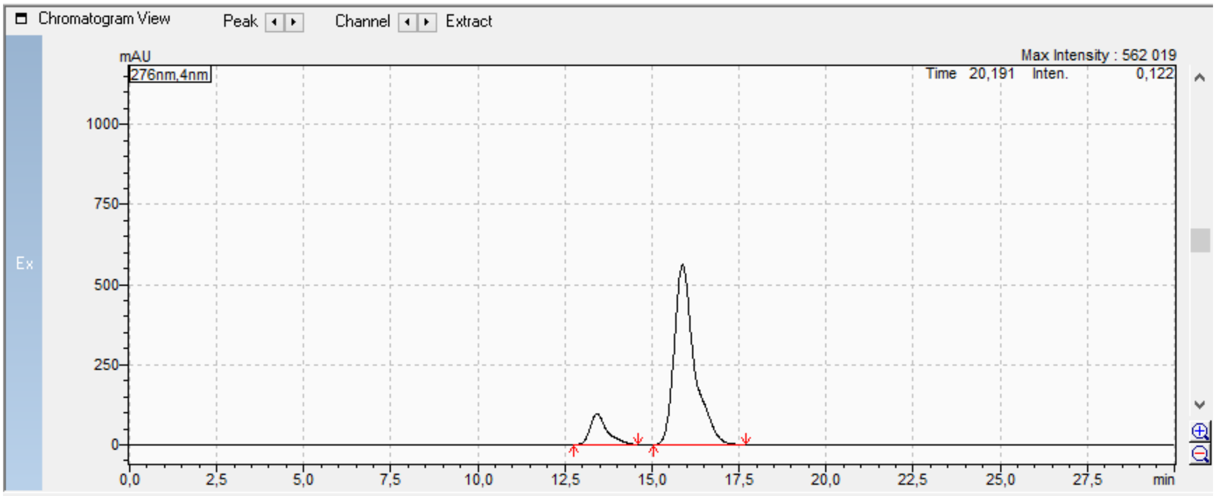

Results View - Peak Table

Peak Table | Compound | Group | Calibration Curve

Peak#	Ret. Time	Area	Height	Peak Start	Peak End	Mark	Conc.	Unit	Area%
1	13,421	3289965	96245	12,747	14,603	M	12,671		12,671
2	15,879	22674674	561251	15,040	17,696	M	87,329		87,329
Total		25964639	657496				100,000		100,000

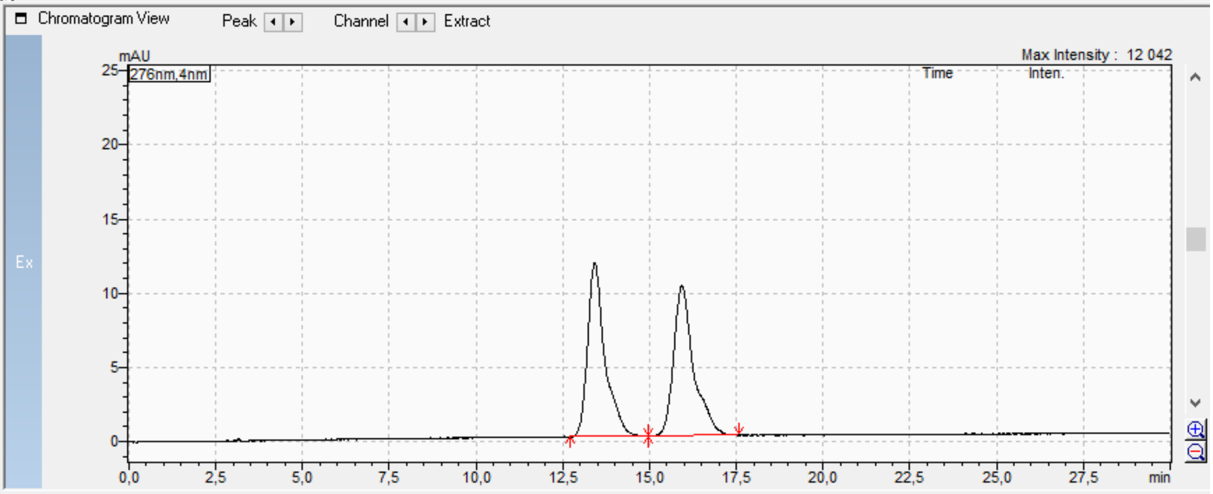

Results View - Peak Table

Peak Table | Compound | Group | Calibration Curve

Peak#	Ret. Time	Area	Height	Peak Start	Peak End	Mark	Conc.	Unit	Area%
1	13,422	402496	11678	12,704	14,944	M	49,864		49,864
2	15,933	404693	10082	14,944	17,568	M	50,136		50,136
Total		807190	21760				100,000		100,000

for **3p**: *er* = 87.3:12.7 (*ee* = 75 %)

(1*R*,3*R*,5*S*,6*R*,7*S*,8*R*,9*S*)-8-((*tert*-Butyldimethylsilyl)oxy)-9-hydroxy-3-phenyl-2,4,10-trioxaadamantan-6-yl (*E*)-3-(2-chlorophenyl)acrylate (3q)

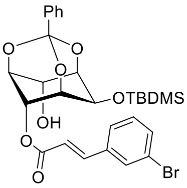

Conditions: IA column
mobile phase: *n*-heptane/*i*-PrOH – 95:5
λ = 275 nm, V = 1.0 ml/min, t = 25 °C
for **3q**: t_R = 13.2 min (minor), t_R = 15.9 min (major)

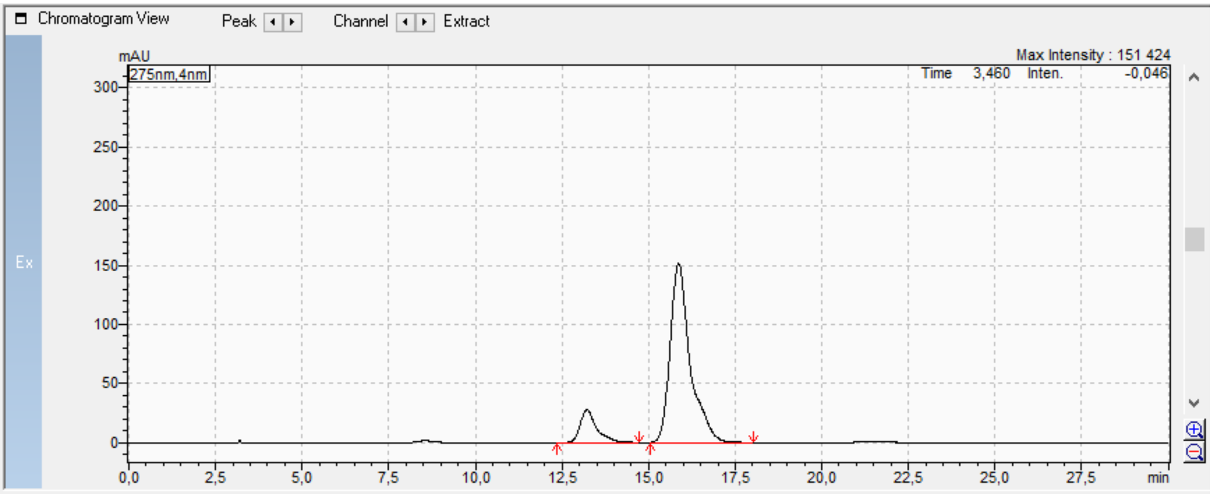

Results View - Peak Table

Peak Table | Compound | Group | Calibration Curve

Peak#	Ret. Time	Area	Height	Peak Start	Peak End	Mark	Conc.	Unit	Area%
1	13,220	925810	27777	12,341	14,731	M	13,254		13,254
2	15,867	6059234	151106	15,051	18,027	M	86,746		86,746
Total		6985043	178883				100,000		100,000

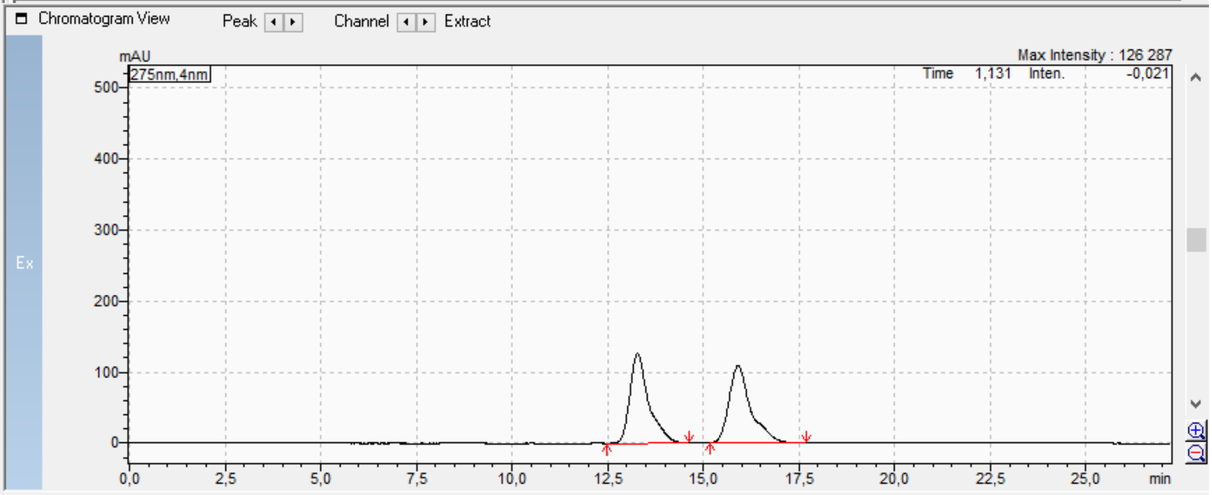

Results View - Peak Table

Peak Table | Compound | Group | Calibration Curve

Peak#	Ret. Time	Area	Height	Peak Start	Peak End	Mark	Conc.	Unit	Area%
1	13,281	4324538	126288	12,491	14,613	M	50,101		50,101
2	15,906	4307134	109092	15,157	17,685	M	49,899		49,899
Total		8631672	235380				100,000		100,000

for **3q**: *er* = 86.7:13.3 (*ee* = 73 %)

(1*R*,3*R*,5*S*,6*R*,7*S*,8*R*,9*S*)-8-((*tert*-Butyldimethylsilyl)oxy)-9-hydroxy-3-phenyl-2,4,10-trioxaadamantan-6-yl (*E*)-3-(2-bromophenyl)acrylate (3r)

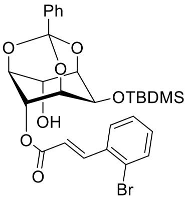

Conditions: IA column
mobile phase: *n*-heptane/*i*-PrOH – 95:5
λ = 271 nm, V = 1.0 ml/min, t = 25 °C
for **3r**: t_R = 10.6 min (minor), t_R = 14.3 min (major)

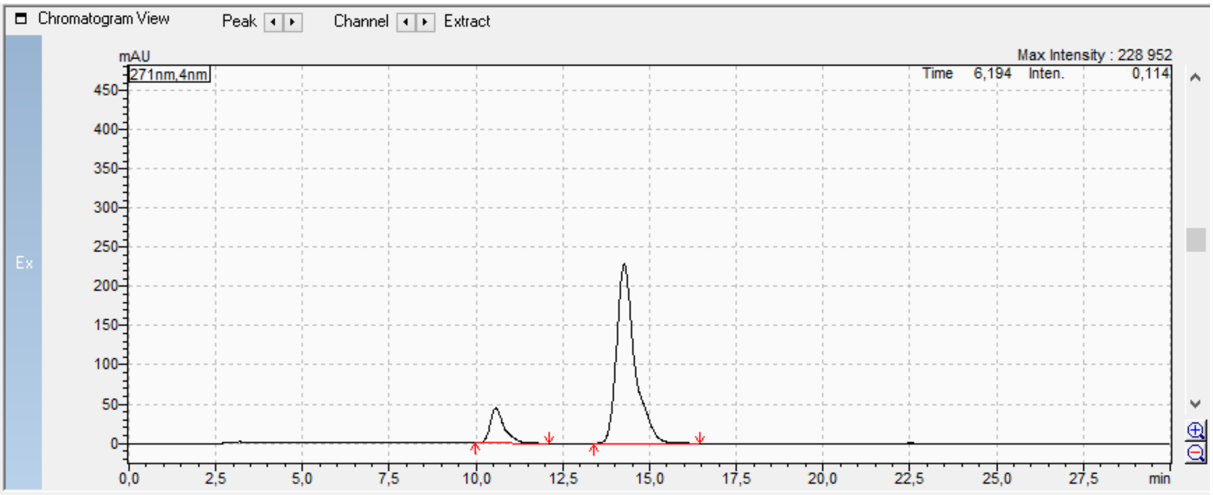

Results View - Peak Table

Peak Table | Compound | Group | Calibration Curve

Peak#	Ret. Time	Area	Height	Peak Start	Peak End	Mark	Conc.	Unit	Area%
1	10,573	1242223	44534	9,984	12,096	M	12,954		12,954
2	14,273	8347526	229026	13,376	16,437	M	87,046		87,046
Total		9589749	273560				100,000		100,000

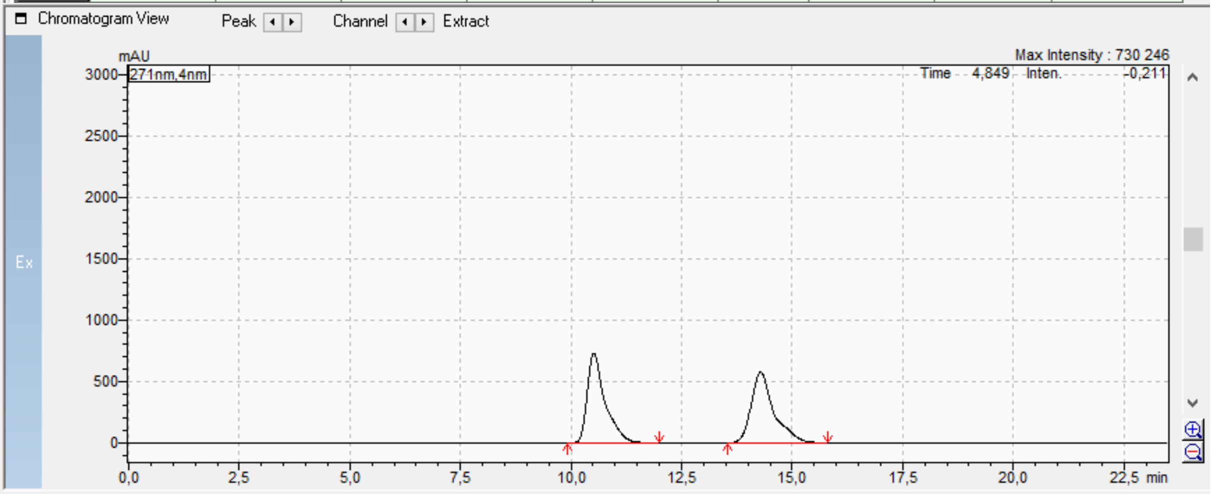

Results View - Peak Table

Peak Table | Compound | Group | Calibration Curve

Peak#	Ret. Time	Area	Height	Peak Start	Peak End	Mark	Conc.	Unit	Area%
1	10,518	20620153	730272	9,909	11,989	M	50,133		50,133
2	14,289	20510568	574766	13,547	15,797	M	49,867		49,867
Total		41130720	1305038				100,000		100,000

for **3r**: *er* = 87.0:13.0 (*ee* = 74 %)

(1*R*,3*R*,5*S*,6*R*,7*S*,8*R*,9*S*)-8-((*tert*-Butyldimethylsilyl)oxy)-9-hydroxy-3-phenyl-2,4,10-trioxaadamantan-6-yl (*E*)-3-(furan-2-yl)acrylate (3s)

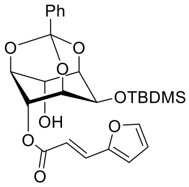

Conditions: IA column
mobile phase: *n*-heptane/*i*-PrOH – 95:5
λ = 303 nm, V = 1.0 ml/min, t = 25 °C
for **3s**: t_R = 13.2 min (minor), t_R = 20.5 min (major)

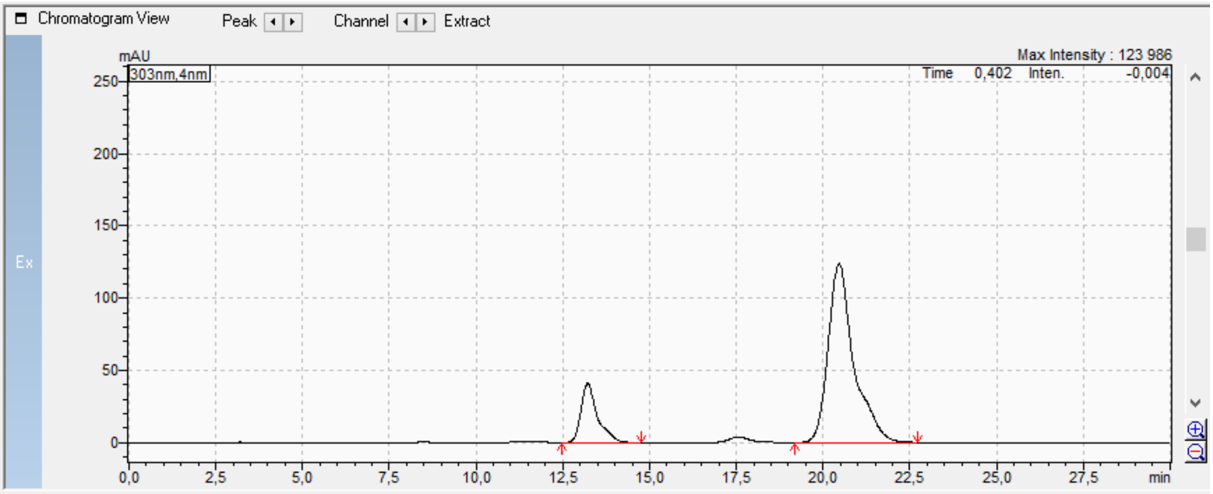

Results View - Peak Table

Peak Table | Compound | Group | Calibration Curve

Peak#	Ret. Time	Area	Height	Peak Start	Peak End	Mark	Conc.	Unit	Area%
1	13,220	1353241	41144	12,459	14,741	M	17,514		17,514
2	20,466	6373236	123849	19,200	22,709	M	82,486		82,486
Total		7726477	164993				100,000		100,000

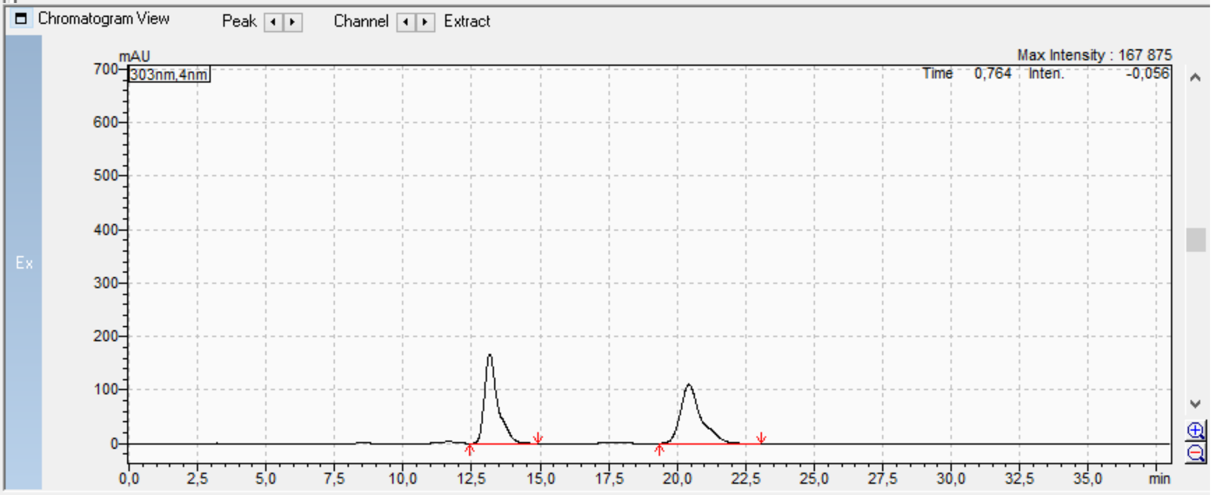

Results View - Peak Table

Peak Table | Compound | Group | Calibration Curve

Peak#	Ret. Time	Area	Height	Peak Start	Peak End	Mark	Conc.	Unit	Area%
1	13,160	5622962	167534	12,427	14,923	M	49,560		49,560
2	20,428	5722732	110237	19,371	23,061	M	50,440		50,440
Total		11345694	277771				100,000		100,000

for **3s**: *er* = 82.5:17.5 (*ee* = 65 %)

(1*R*,3*R*,5*S*,6*R*,7*S*,8*R*,9*S*)-8-((*tert*-Bbutyldimethylsilyl)oxy)-9-hydroxy-3-phenyl-2,4,10-trioxaadamantan-6-yl (*E*)-3-(thiophen-2-yl)acrylate (3t)

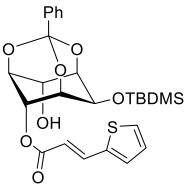

Conditions: IA column
mobile phase: *n*-heptane/*i*-PrOH – 95:5
λ = 309 nm, V = 1.0 ml/min, t = 25 °C
for **3t**: t_R = 20.0 min (minor), t_R = 24.2 min (major)

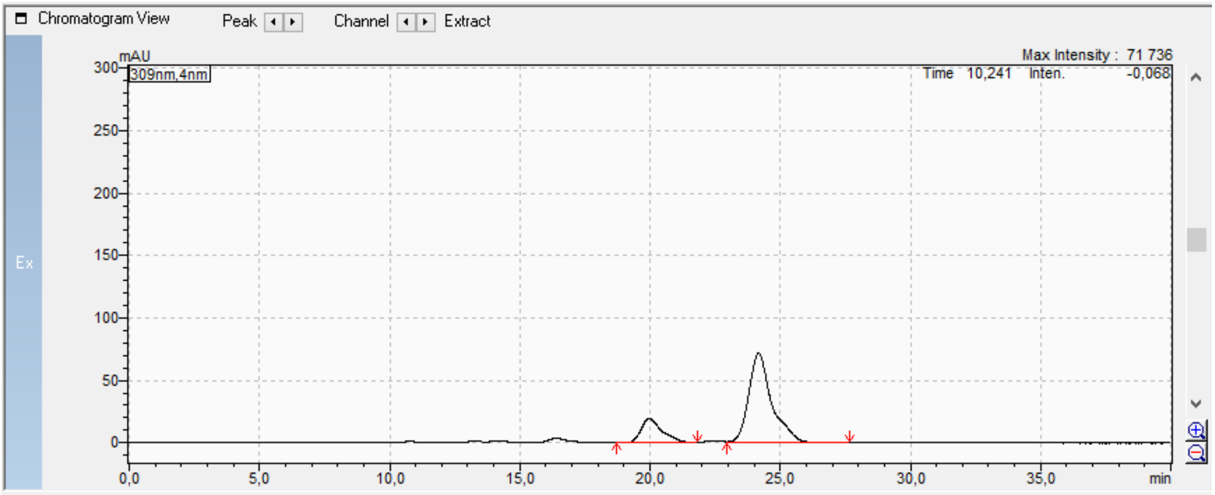

Results View - Peak Table

Peak Table | Compound | Group | Calibration Curve

Peak#	Ret. Time	Area	Height	Peak Start	Peak End	Mark	Conc.	Unit	Area%
1	19,976	1078751	19595	18,709	21,813	M	19,967		19,967
2	24,170	4323786	71139	22,944	27,669	M	80,033		80,033
Total		5402536	90735				100,000		100,000

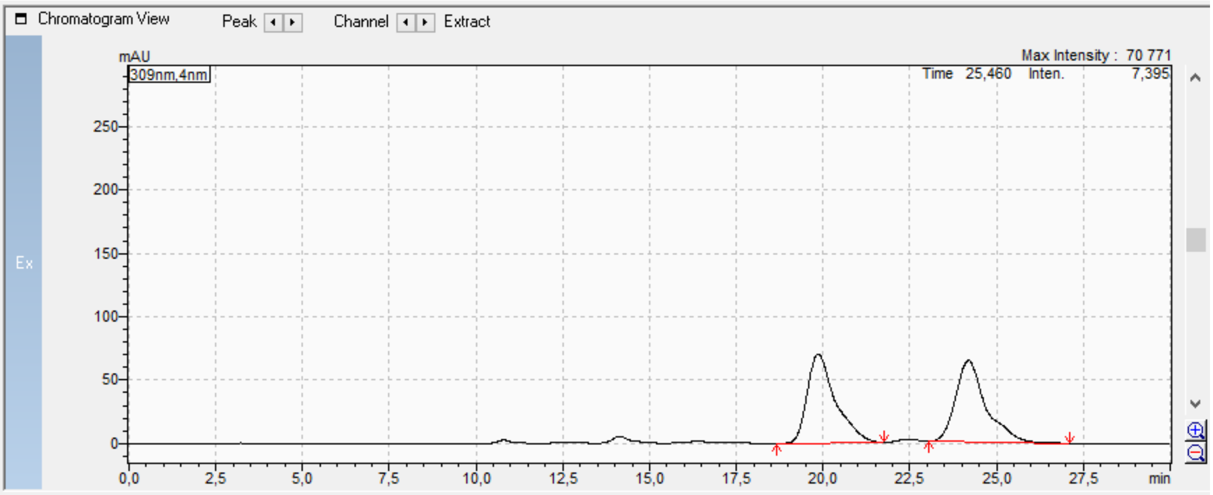

Results View - Peak Table

Peak Table | Compound | Group | Calibration Curve

Peak#	Ret. Time	Area	Height	Peak Start	Peak End	Mark	Conc.	Unit	Area%
1	19,863	3953417	70341	18,645	21,760	M	50,349		50,349
2	24,194	3898589	64450	23,061	27,093	M	49,651		49,651
Total		7852006	134791				100,000		100,000

for **3t**: *er* = 80.0:20.0 (*ee* = 60 %)

(1*R*,3*R*,5*S*,6*R*,7*S*,8*R*,9*S*)-8-((*tert*-butyldimethylsilyl)oxy)-9-hydroxy-3-phenyl-2,4,10-trioxaadamantan-6-yl ethyl fumarate (3u)

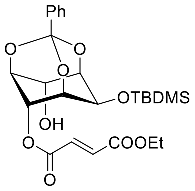

Conditions: IB column
mobile phase: *n*-heptane/*i*-PrOH – 80:20
λ = 208 nm, V = 1.0 ml/min, t = 25 °C
for **3u**: t_R = 5.2 min (minor), t_R = 6.3 min (major)

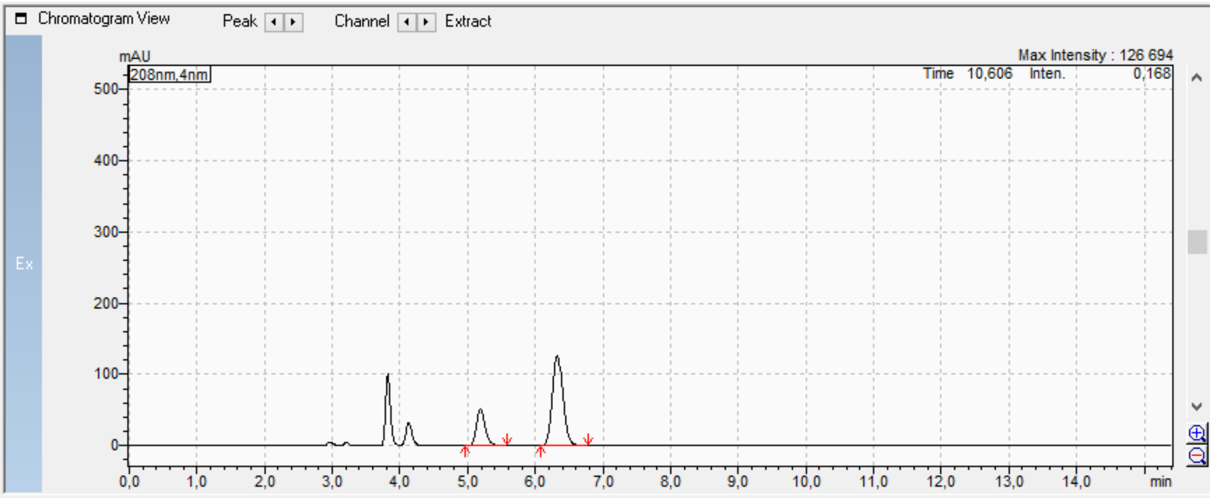

Results View - Peak Table

Peak Table | Compound | Group | Calibration Curve

Peak#	Ret. Time	Area	Height	Peak Start	Peak End	Mark	Conc.	Unit	Area%
1	5,190	477247	51887	4,960	5,579	M	25,891		25,891
2	6,327	1366036	126469	6,069	6,773	M	74,109		74,109
Total		1843283	178356				100,000		100,000

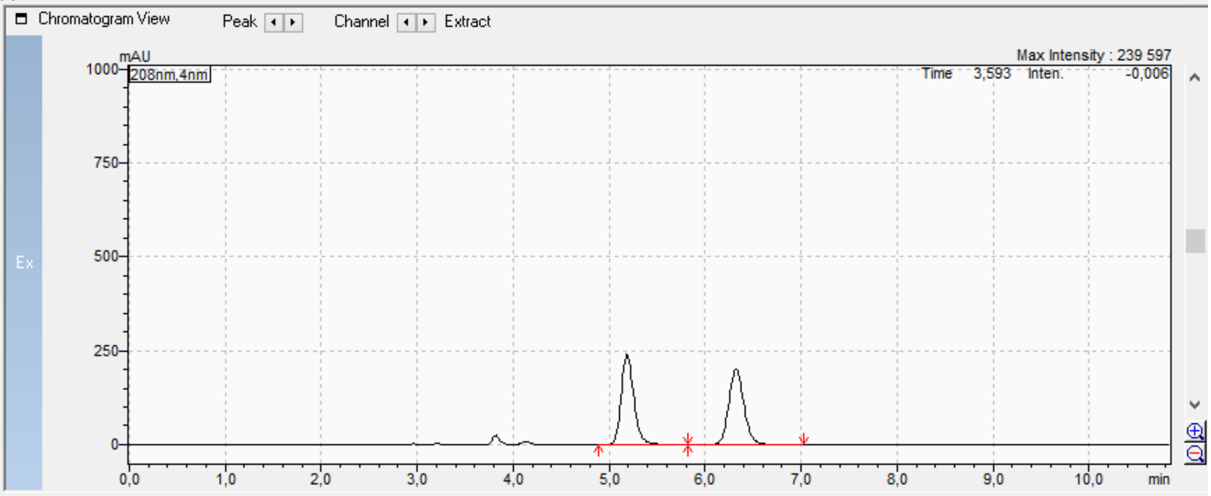

Results View - Peak Table

Peak Table | Compound | Group | Calibration Curve

Peak#	Ret. Time	Area	Height	Peak Start	Peak End	Mark	Conc.	Unit	Area%
1	5,184	2189582	239373	4,885	5,824	M	49,920		49,920
2	6,321	2196574	201460	5,824	7,029	M	50,080		50,080
Total		4386156	440833				100,000		100,000

for **3u**: *er* = 74.1:25.9 (*ee* = 48 %)

(1*R*,3*R*,5*S*,6*R*,7*S*,8*S*,9*R*)-8-Acetoxy-9-((*tert*-butyldimethylsilyl)oxy)-3-phenyl-2,4,10-trioxaadamantan-6-yl cinnamate (4a)

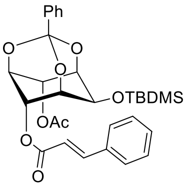

Conditions: IG column
mobile phase: *n*-heptane/*i*-PrOH – 95:5
λ = 276 nm, V = 1.0 ml/min, t = 25 °C
for **4a**: t_R = 19.8 min (minor), t_R = 22.1 min (major)

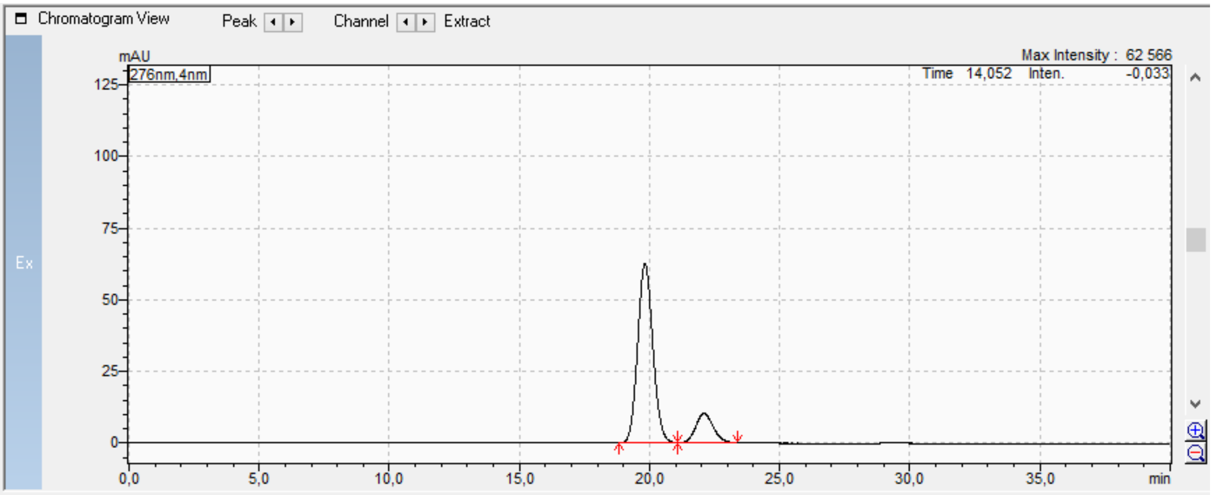

Results View - Peak Table

Peak Table | Compound | Group | Calibration Curve

Peak#	Ret. Time	Area	Height	Peak Start	Peak End	Mark	Conc.	Unit	Area%
1	19,826	2565888	62596	18,837	21,088		83,985		83,985
2	22,093	489291	10298	21,088	23,392	V	16,015		16,015
Total		3055179	72894				100,000		100,000

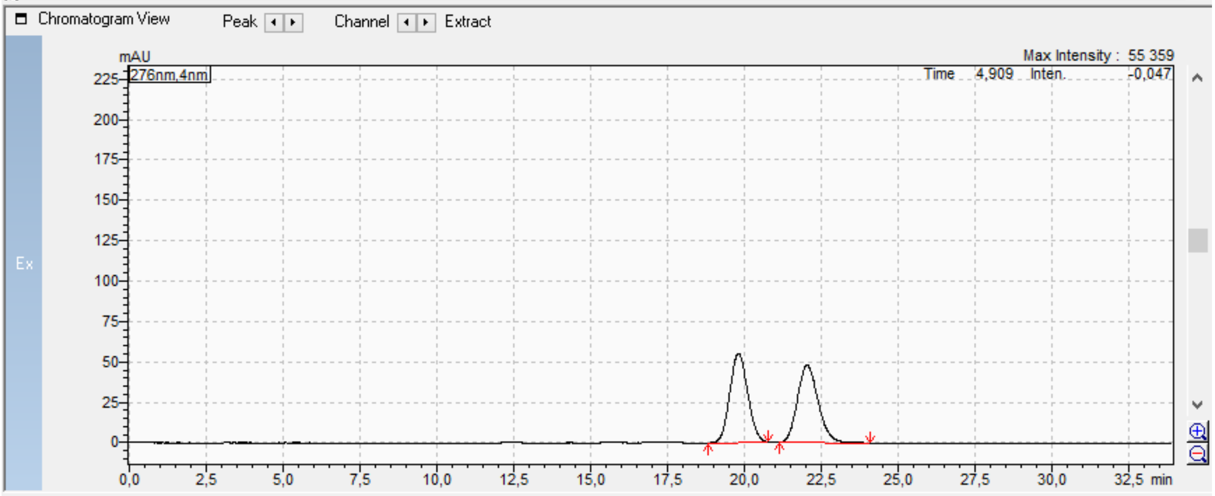

Results View - Peak Table

Peak Table | Compound | Group | Calibration Curve

Peak#	Ret. Time	Area	Height	Peak Start	Peak End	Mark	Conc.	Unit	Area%
1	19,808	2237797	55199	18,816	20,768	M	50,004		50,004
2	22,041	2237407	47849	21,141	24,096	M	49,996		49,996
Total		4475203	103048				100,000		100,000

for **4a**: *er* = 84.0:16.0 (*ee* = 68 %)

(1*R*,3*R*,5*S*,6*R*,7*S*,8*S*,9*R*)-8-Acetoxy-9-((*tert*-butyldimethylsilyl)oxy)-3-phenyl-2,4,10-trioxaadamantan-6-yl (*E*)-3-(4-methoxyphenyl)acrylate (4i)

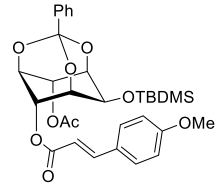

Conditions: IG column

mobile phase: *n*-heptane/*i*-PrOH – 80:20

λ = 310 nm, V = 1.0 ml/min, t = 25 °C

for **4i**: t_R = 14.3 min (minor), t_R = 16.5 min (major)

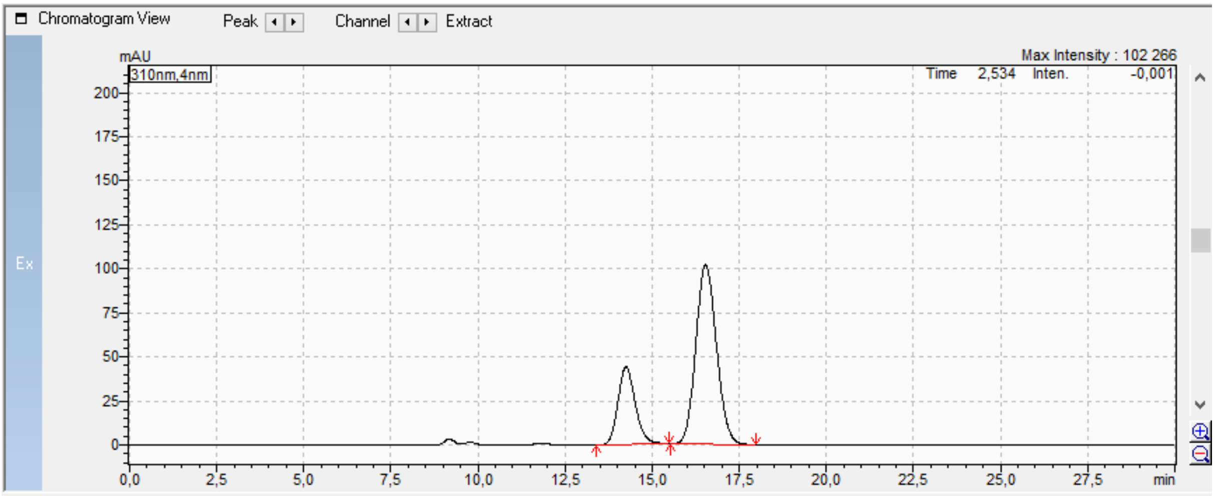

Results View - Peak Table

Peak Table | Compound | Group | Calibration Curve

Peak#	Ret. Time	Area	Height	Peak Start	Peak End	Mark	Conc.	Unit	Area%
1	14,250	1518605	44038	13,397	15,488	M	26,532		26,532
2	16,536	4205098	101820	15,531	17,984	M	73,468		73,468
Total		5723703	145858				100,000		100,000

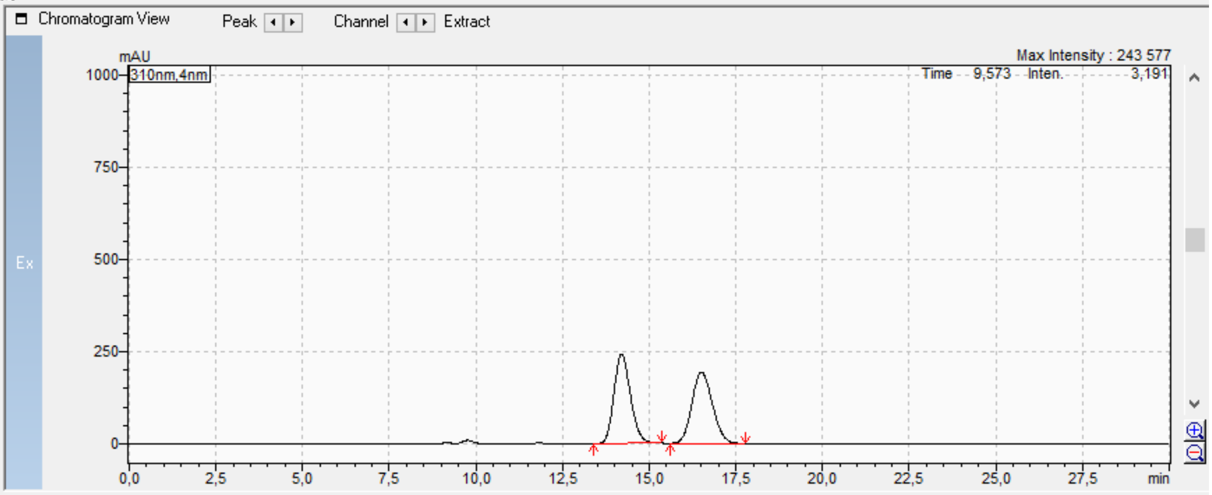

Results View - Peak Table

Peak Table | Compound | Group | Calibration Curve

Peak#	Ret. Time	Area	Height	Peak Start	Peak End	Mark	Conc.	Unit	Area%
1	14,203	8078191	242919	13,397	15,360	M	50,350		50,350
2	16,509	7965902	193436	15,605	17,781	M	49,650		49,650
Total		16044093	436356				100,000		100,000

for **4i**: *er* = 73.5:26.5 (*ee* = 47 %)

(1*R*,3*R*,5*S*,6*R*,7*S*,8*S*,9*R*)-8-Acetoxy-9-((*tert*-butyldimethylsilyl)oxy)-3-phenyl-2,4,10-trioxaadamantan-6-yl (*E*)-3-(4-nitrophenyl)acrylate (4k)

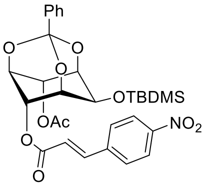

Conditions: IB column
mobile phase: *n*-heptane/*i*-PrOH – 80:20
λ = 294 nm, V = 1.0 ml/min, t = 25 °C
for **4k**: t_R = 18.9 min (minor), t_R = 20.9 min (major)

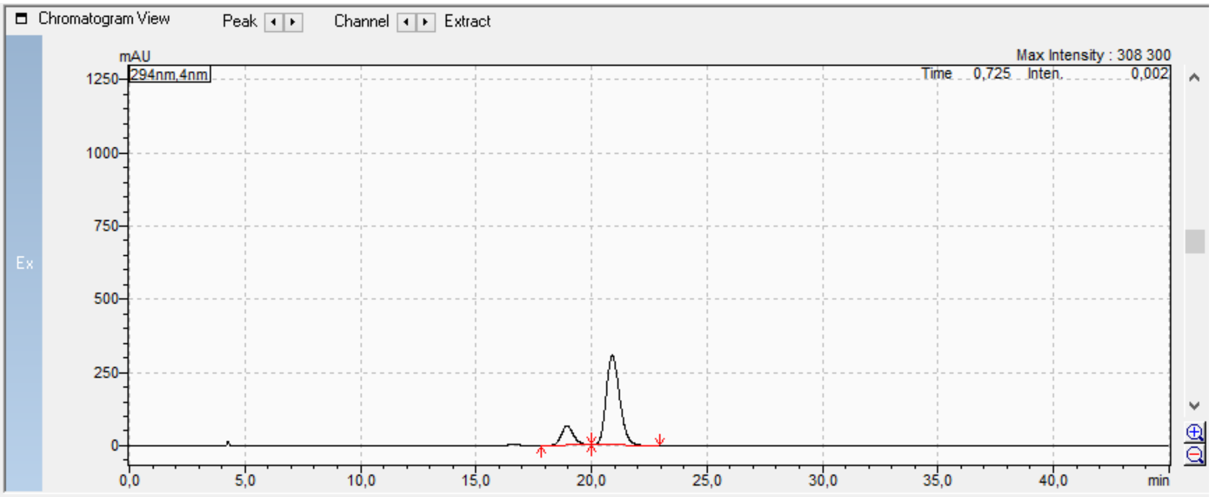

Results View - Peak Table

Peak Table | Compound | Group | Calibration Curve

Peak#	Ret. Time	Area	Height	Peak Start	Peak End	Mark	Conc.	Unit	Area%
1	18,944	2468479	66325	17,824	20,000	M	16,587		16,587
2	20,904	12413673	306658	20,000	22,965	M	83,413		83,413
Total		14882152	372983				100,000		100,000

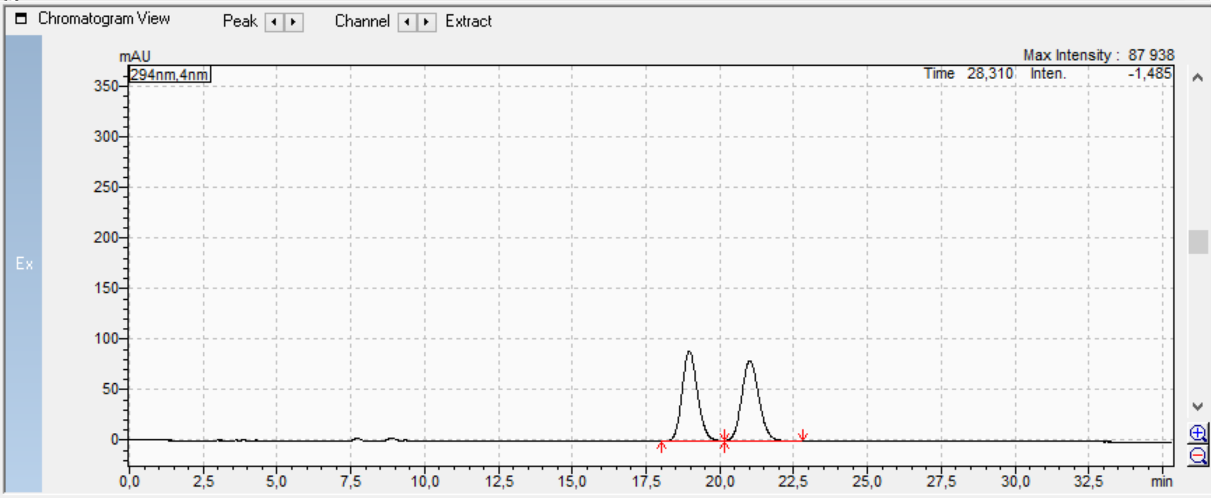

Results View - Peak Table

Peak Table | Compound | Group | Calibration Curve

Peak#	Ret. Time	Area	Height	Peak Start	Peak End	Mark	Conc.	Unit	Area%
1	18,963	3230735	88868	18,016	20,149	M	50,020		50,020
2	21,010	3228124	79613	20,149	22,805	M	49,980		49,980
Total		6458860	168481				100,000		100,000

for **4k**: *er* = 83.4:16.6 (*ee* = 67 %)

(1*R*,3*R*,5*S*,6*R*,7*S*,8*R*,9*S*)-8-((*tert*-Butyldimethylsilyl)oxy)-9-hydroxy-3-phenyl-2,4,10-trioxaadamantan-6-yl 2-(2-(*tert*-butyl)-6-methylphenoxy)-3-formylbenzoate (5b)

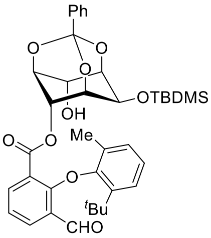

Prepared with optimized reaction conditions

Conditions: IB column

mobile phase: *n*-heptane/*i*-PrOH – 97:3

λ = 206 nm, V = 1.0 ml/min, t = 25 °C

for **5b**: t_R = 16.1 min (major), t_R = 17.6 min (minor), t_R = 20.2 min (minor'), t_R = 24.9 min (major')

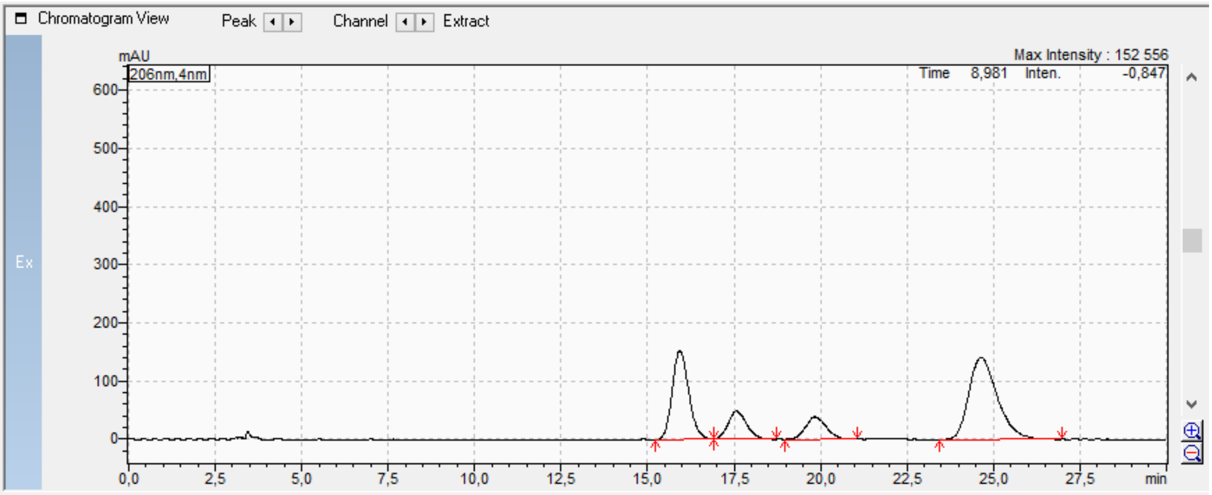

Results View - Peak Table

Peak Table | Compound | Group | Calibration Curve

Peak#	Ret. Time	Area	Height	Peak Start	Peak End	Mark	Conc.	Unit	Area%
1	15,931	4975358	152412	15,221	16,896	M	30,301		30,301
2	17,566	1742737	47200	16,896	18,731	M	10,614		10,614
3	19,848	1621486	38414	18,965	21,067	M	9,875		9,875
4	24,657	8080349	141527	23,445	26,987	M	49,211		49,211
Total		16419930	379552				100,000		100,000

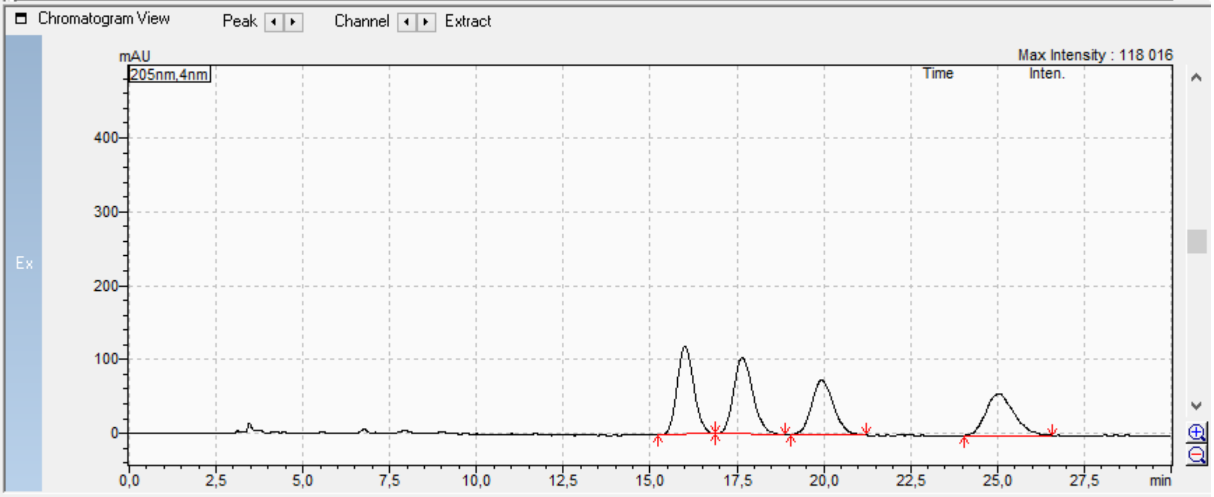

Results View - Peak Table

Peak Table | Compound | Group | Calibration Curve

Peak#	Ret. Time	Area	Height	Peak Start	Peak End	Mark	Conc.	Unit	Area%
1	16,000	3945788	119046	15,221	16,885	M	27,410		27,410
2	17,650	3928634	103426	16,885	18,901	M	27,290		27,290
3	19,931	3273777	74162	19,061	21,237	M	22,741		22,741
4	25,059	3247450	56778	24,032	26,592	M	22,559		22,559
Total		14395649	353411				100,000		100,000

Not: Prepared with optimized reaction conditions,

For **5b**: *dr* = 1:1.4, *er* = 74:26 / 83:17

(1*R*,3*R*,5*S*,6*R*,7*S*,8*R*,9*S*)-8-((*tert*-butyldimethylsilyl)oxy)-9-hydroxy-3-phenyl-2,4,10-trioxaadamantan-6-yl 2-(2-(*tert*-butyl)-6-methylphenoxy)-3-formylbenzoate (5b)

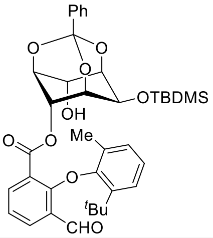

Conditions: IB column
mobile phase: *n*-heptane/*i*-PrOH – 97:3
λ = 208 nm, V = 1.0 ml/min, t = 25 °C
for **5b**: t_R = 16.1 min (minor), t_R = 17.6 min (major), t_R = 20.2 min (minor'), t_R = 24.9 min (major')

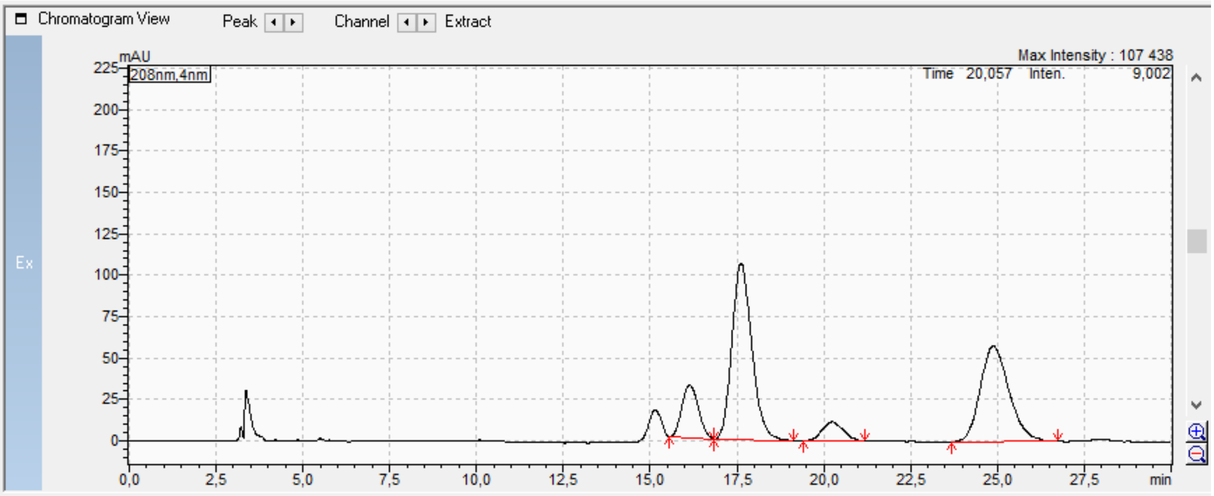

Results View - Peak Table

Peak Table | Compound | Group | Calibration Curve

Peak#	Ret. Time	Area	Height	Peak Start	Peak End	Mark	Conc.	Unit	Area%
1	16,133	1061134	31888	15,563	16,843	M	11,565		11,565
2	17,620	4270508	106762	16,843	19,136	M	46,544		46,544
3	20,248	495318	11629	19,413	21,184	M	5,398		5,398
4	24,880	3348185	57962	23,680	26,741	M	36,492		36,492
Total		9175145	208242				100,000		100,000

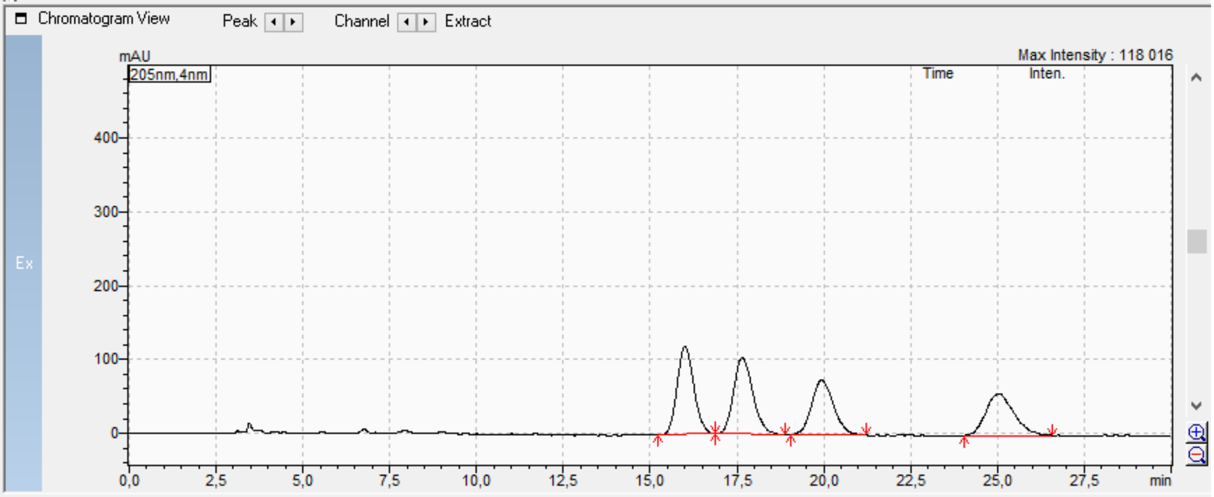

Results View - Peak Table

Peak Table | Compound | Group | Calibration Curve

Peak#	Ret. Time	Area	Height	Peak Start	Peak End	Mark	Conc.	Unit	Area%
1	16,000	3945788	119046	15,221	16,885	M	27,410		27,410
2	17,650	3928634	103426	16,885	18,901	M	27,290		27,290
3	19,931	3273777	74162	19,061	21,237	M	22,741		22,741
4	25,059	3247450	56778	24,032	26,592	M	22,559		22,559
Total		14395649	353411				100,000		100,000

Not: Prepared with previously reported reaction conditions.[8]
For **5b**: *dr* = 1.4:1, *er* = 20:80 / 87:13

(1*R*,3*S*,5*S*,6*R*,7*R*,8*S*,9*S*)-8-((*tert*-Butoxycarbonyl)oxy)-9-((*tert*-butyldimethylsilyl)oxy)-3-phenyl-2,4,10-trioxaadamantan-6-yl cinnamate (6)

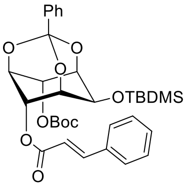

Conditions: ODH column
mobile phase: *n*-heptane/*i*-PrOH – 99:1
λ = 275 nm, V = 1.0 ml/min, t = 25 °C
for **6**: t_R = 17.4 min (major), t_R = 31.3 min (minor)

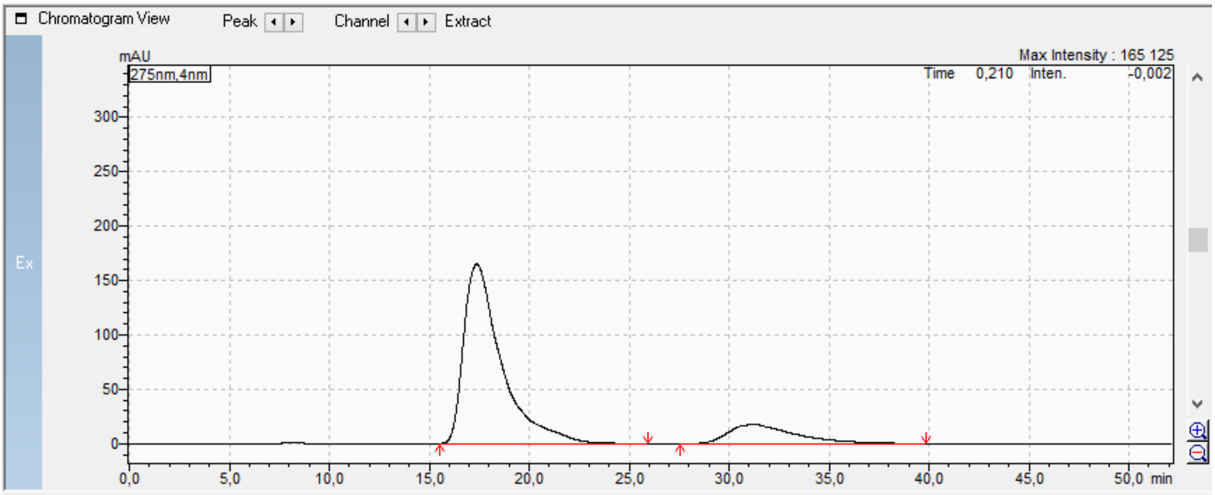

Results View - Peak Table

Peak Table | Compound | Group | Calibration Curve

Peak#	Ret. Time	Area	Height	Peak Start	Peak End	Mark	Conc.	Unit	Area%
1	17,380	21755953	165044	15,509	25,941	M	84,287		84,287
2	31,259	4055709	17499	27,552	39,851	S M	15,713		15,713
Total		25811663	182543				100,000		100,000

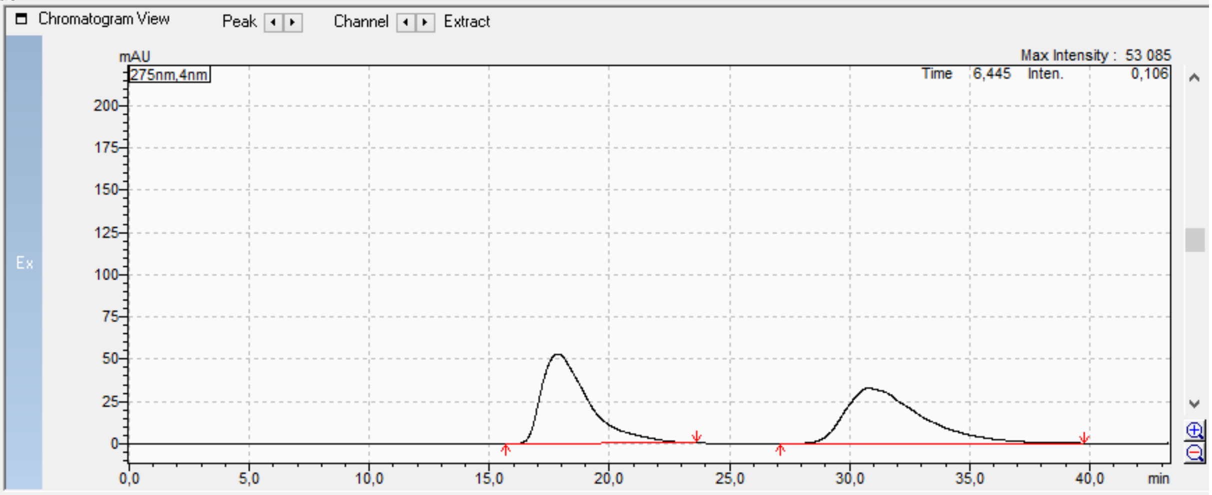

Results View - Peak Table

Peak Table | Compound | Group | Calibration Curve

Peak#	Ret. Time	Area	Height	Peak Start	Peak End	Mark	Conc.	Unit	Area%
1	17,854	7196131	52932	15,680	23,637	M	50,150		50,150
2	30,805	7153155	32702	27,115	39,776	M	49,850		49,850
Total		14349286	85634				100,000		100,000

for **6**: *er* = 84.3:15.7 (*ee* = 69 %)

(1*S*,3*R*,5*S*,6*S*,7*R*,8*R*,9*R*)-8-((*tert*-Butyldimethylsilyl)oxy)-3-phenyl-9-(((*E*)-prop-1-en-1-yl)oxy)-2,4,10-trioxaadamantan-6-ol (11)

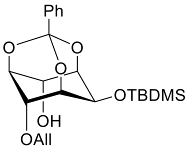

Conditions: IH column
mobile phase: *n*-heptane/*i*-PrOH – 98:2
λ = 207 nm, V = 1.0 ml/min, t = 25 °C
for **11**: t_R = 6.4 min (minor), t_R = 7.2 min (major)

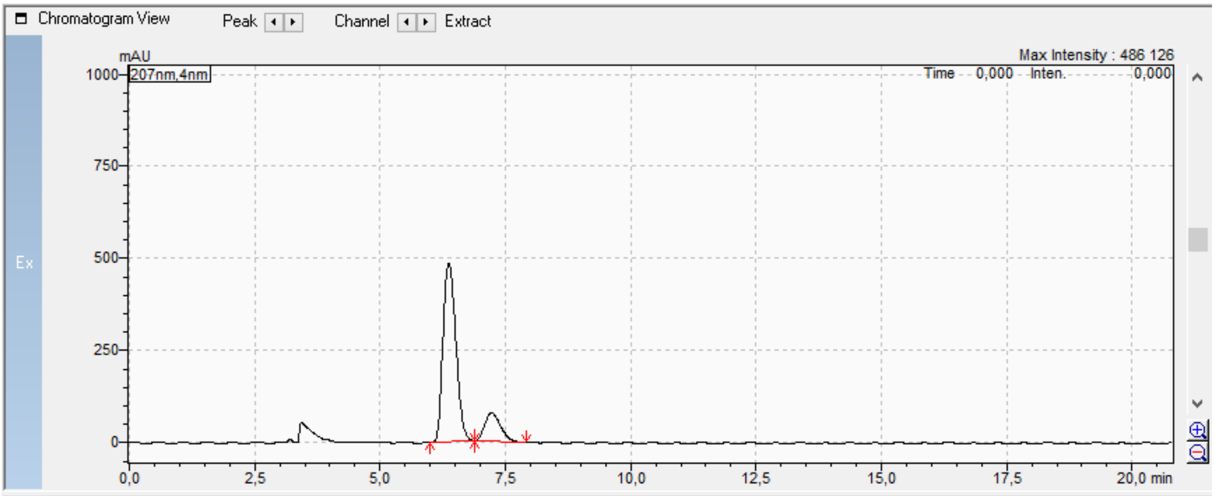

Results View - Peak Table

Peak Table | Compound | Group | Calibration Curve

Peak#	Ret. Time	Area	Height	Peak Start	Peak End	Mark	Conc.	Unit	Area%
1	6,373	8683175	483825	5,995	6,891	M	83,700		83,700
2	7,224	1690955	76569	6,891	7,915	M	16,300		16,300
Total		10374130	560394				100,000		100,000

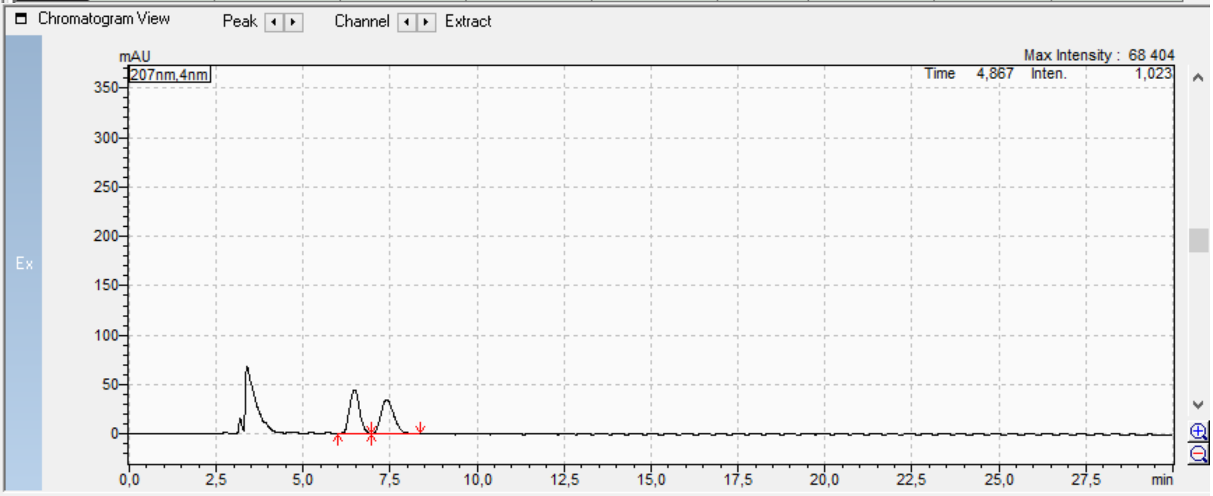

Results View - Peak Table

Peak Table | Compound | Group | Calibration Curve

Peak#	Ret. Time	Area	Height	Peak Start	Peak End	Mark	Conc.	Unit	Area%
1	6,475	886261	43749	5,973	6,955	M	49,511		49,511
2	7,402	903775	34249	6,955	8,384	M	50,489		50,489
Total		1790037	77998				100,000		100,000

for **11**: *er* = 83.7:16.3 (*ee* = 67 %)

References

(1) Sheldrick, G. M. SHELXT – Integrated Space-Group and Crystal-Structure Determination. *Acta Crystallogr. Sect. Found. Adv.* **2015**, *71* (1), 3–8. https://doi.org/10.1107/S2053273314026370.

(2) Sheldrick, G. M. Crystal Structure Refinement with SHELXL. *Acta Crystallogr. Sect. C Struct. Chem.* **2015**, *71* (1), 3–8. https://doi.org/10.1107/S2053229614024218.

(3) Parsons, S.; Flack, H. D.; Wagner, T. Use of Intensity Quotients and Differences in Absolute Structure Refinement. *Acta Crystallogr. Sect. B Struct. Sci. Cryst. Eng. Mater.* **2013**, *69* (3), 249–259. https://doi.org/10.1107/S2052519213010014.
